# Catalytic direct hydrocarboxylation of styrenes with CO_2_ and H_2_

**DOI:** 10.1038/s41467-022-35293-3

**Published:** 2022-12-08

**Authors:** Yushu Jin, Joaquim Caner, Shintaro Nishikawa, Naoyuki Toriumi, Nobuharu Iwasawa

**Affiliations:** Department of Chemistry, Tokyo Institute of Technology, O-okayama, Meguro-ku, Tokyo, 152-8551 Japan

**Keywords:** Homogeneous catalysis, Synthetic chemistry methodology

## Abstract

A three-component hydrocarboxylation of an olefin with CO_2_ and H_2_ could be regarded as a dream reaction, since it would provide a straightforward approach for the synthesis of aliphatic carboxylic acids in perfect atom economy. However, this transformation has not been realized in a direct manner under mild conditions, because boosting the carboxylation with thermodynamically stable CO_2_ while suppressing the rapid hydrogenation of olefin remains a challenging task. Here, we report a rhodium-catalysed reductive hydrocarboxylation of styrene derivatives with CO_2_ and H_2_ under mild conditions, in which H_2_ served as the terminal reductant. In this approach, the carboxylation process was largely accelerated by visible light irradiation, which was proved both experimentally and by computational studies. Hydrocarboxylation of various kinds of styrene derivatives was achieved in good yields without additional base under ambient pressure of CO_2_/H_2_ at room temperature. Mechanistic investigations revealed that use of a cationic rhodium complex was critical to achieve high hydrocarboxylation selectivity.

## Abstract (web summary)

Hydrocarboxylation of olefins with CO_2_ provides a useful approach for the synthesis of aliphatic carboxylic acids. Here, the authors report a rhodium-catalysed hydrocarboxylation of styrenes with CO_2_, using hydrogen as the terminal reductant.

## Introduction

**Fig. 1:**
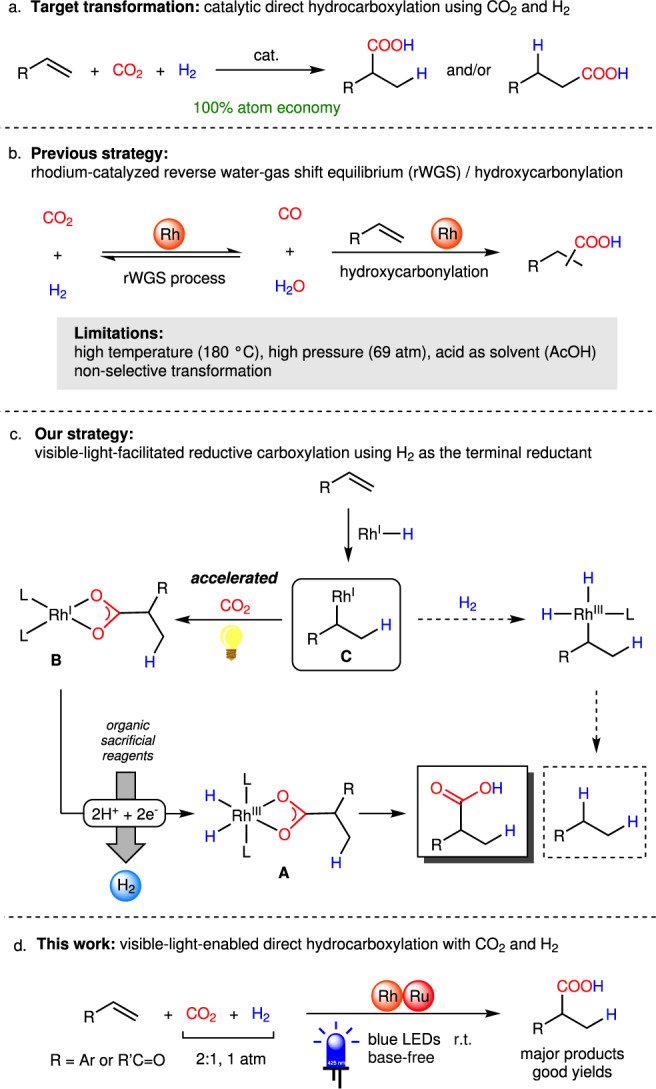
Carboxylation of olefinic compounds using CO_2_ and H_2_. **a** Target transformation: catalytic direct hydrocarboxylation of olefins using CO_2_ and H_2_, a 100% atom economical reaction. **b** Previous strategy: rhodium-catalysed reverse water-gas shift equilibrium (rWGS)/hydroxycarbonylation. **c** Our strategy: visible-light-facilitated reductive carboxylation using H_2_ as the terminal reductant. **d** This work: visible-light-enabled direct hydrocarboxylation with CO_2_ and H_2_. LED, light-emitting diode.

The increasing demands for environmental friendliness and sustainable development inspired scientists to search new applications of utilizing renewable resources to industrial chemical feedstock^1^. During the past decades, great efforts have been made on transforming carbon dioxide (CO_2_), which is the critical compound for the greenhouse effect, into useful chemicals in the field of synthetic chemistry^2–5^. Dihydrogen (H_2_), regarded as one of the best and cleanest energy sources, has long been expected to be a potential partner with CO_2_ in organic synthesis^6^, and extensive studies have been carried out for the reduction of CO_2_ with H_2_ to give formic acid, methanol, etc^7,8^. Catalytic production of carboxylic acids, alcohols, and alkanes with two or more carbons from CO_2_ and H_2_ has also been explored in recent years^9^. As a one-carbon (C_1_) source, another straightforward exit for CO_2_ is converting it into carboxylic acid derivatives by the reaction with organic substrates^10–17^, however, the reaction of organic compounds in combination with CO_2_ and H_2_ has rarely been studied. Among these transformations, a three-component hydrocarboxylation, which is the addition of CO_2_ and H_2_ to an olefin, could be regarded as a dream reaction, because it would provide the corresponding aliphatic carboxylic acid with 100% atom economy (Fig. 1a), while the existing examples of olefin hydrocarboxylations with CO_2_ using Cu^18–21^, Rh^22–24^, Ti^25^, Ni^26–28^, Ru^29^, Fe^30^, Co^31^, Zr^32^, and Pd^33,34^ catalysts or light energy^35–38^ could hardly get rid of using a stoichiometric amount of base or metallic/organic reductants, which are out of the concept of green chemistry and sustainability. A pioneering work of Rh-catalysed formal hydrocarboxylation of olefins with CO_2_ and H_2_ has been described by Leitner’s group in 2013, but the reaction was proved to proceed through a hydroxycarbonylation pathway with in situ generation of carbon monoxide (CO) and H_2_O via reverse water-gas shift equilibrium (rWGS) under high temperature (180 ˚C) and high pressure of CO_2_/H_2_ (6:1, 69 atm) in strongly acidic conditions (Fig. 1b)^39^. Although computational calculation indicated that catalytic direct hydrocarboxylation of olefins with CO_2_ and H_2_ was thermodynamically feasible, the competing hydrogenation reaction, which usually proceeds rapidly, and complexity of the desired reaction pathway have made this dream reaction challenging^40^. To the best of our knowledge, hydrocarboxylation of olefins with CO_2_ and H_2_ via direct insertion of CO_2_ has not yet been realized to date.

In our goal of developing novel and sustainable catalytic systems for the utilization of CO_2_, the potential of rhodium hydride as a catalyst for C–C bond formation^41^, as well as our recent studies on rhodium-catalysed reductive hydrocarboxylation of activated olefins^23,24^ inspired us to tackle this reaction not only under mild conditions but also with high efficiency. In our previous research, we have found that rhodium(III)-dihydride-carboxylate species **A** could be generated from rhodium(I) carboxylate **B**, which was formed by CO_2_ insertion into alkylrhodium(I) species **C** with the support of light energy, using organic sacrificial reagents (i.e. diisopropylethylamine^23^ and BI(OH)H (1,3-dimethyl-2-(*o*-hydroxyphenyl)-2,3-dihydro-1*H*-benzo[*d*]imidazole)^24^) as both proton and electron donors (Fig. 1c). From the mechanistic point of view, in principle, two protons and two electrons (2H^+^ + 2e^–^) can formally be replaced by molecular H_2_, thus providing the opportunity to employ H_2_ as the terminal reductant in our catalytic system. Furthermore, we also envisioned that selective hydrocarboxylation over the undesired hydrogenation may become possible, because the carboxylation process would be boosted under light irradiation.

Here we describe an example of base-free catalytic hydrocarboxylation of styrene derivatives with CO_2_ and H_2_ under mild conditions (Fig. 1d). Our catalytic system allowed the synthesis of aliphatic carboxylic acids under ambient pressure of a CO_2_/H_2_ gas at room temperature with visible light irradiation. Furthermore, it is worth mentioning that base is no longer required in our catalytic system, while conventional reductive carboxylations under base-free conditions have been elusive^42,43^.

## Results and discussion

### Optimization of reaction conditions

We started our investigation with modified conditions based on our previous research^23^. Methyl 4-vinylbenzoate (**1a**) was selected as the model substrate, and our first trial was conducted under an ambient pressure of CO_2_/H_2_ (2:1 ratio) at room temperature in DMA (*N*,*N*-dimethylacetamide) solvent in the presence of 2.0 mol% of [Rh(OAc)(C_2_H_4_)_2_]_2_ (4.0 mol% of Rh atom) as the rhodium precursor, 8.0 mol% of P(4-CF_3_C_6_H_4_)_3_ as the ligand, and 2.0 mol% of [Ru(bpy)_3_](PF_6_)_2_ (tris(2,2’-bipyridine)ruthenium(II) hexafluorophosphate) as the photosensitizer with blue LEDs (425 nm) irradiation. After 5 h, the hydrocarboxylation product **2a** was actually observed in 5% yield, although hydrogenation (**3a**, 74% yield) dominated the reaction (Table 1, entry 1).

**Table 1:** Optimization of reaction conditions and control experiments


Entry	Rh catalyst	Ligand	Yield of 2a (%)	Yield of 3a (%)
1	[Rh(OAc)(C_2_H_4_)_2_]_2_	P(4-CF_3_-C_6_H_4_)_3_	5	74
2	[Rh(OAc)(C_2_H_4_)_2_]_2_	JohnPhos	7	6
3	[Rh(OAc)(C_2_H_4_)_2_]_2_	CyJohnPhos	16	61
4	[Rh(OAc)(C_2_H_4_)_2_]_2_	DavePhos	35	48
5	[Rh(OAc)(C_2_H_4_)_2_]_2_	PhDavePhos	3	30
6	[Rh(OAc)(C_2_H_4_)_2_]_2_	CPhos	17	38
7	[RhCl(C_2_H_4_)_2_]_2_	DavePhos	4	>95
8	[Rh(cod)_2_](OTf)	DavePhos	18	80
9	[Rh(OAc)(DavePhos)]	DavePhos^a^	38	27
10	[Rh(C_2_H_4_)(DavePhos)](OTf) (**4**)	DavePhos^a^	75 (70)	23
11	[Rh(C_2_H_4_)(DavePhos)](OTf) (**4**)	–	10	9
12^b^	[Rh(C_2_H_4_)(DavePhos)](OTf) (**4**)	DavePhos^a^	84 (81)	16
13	–	DavePhos^a^	n.d.	n.d.
14^c^	[Rh(C_2_H_4_)(DavePhos)](OTf) (**4**)	DavePhos^a^	n.d.	13
15^d^	[Rh(C_2_H_4_)(DavePhos)](OTf) (**4**)	DavePhos^a^	n.d.	10
16^e^	[Rh(C_2_H_4_)(DavePhos)](OTf) (**4**)	DavePhos^a^	n.d.	n.d.
17^f^	[Rh(C_2_H_4_)(DavePhos)](OTf) (**4**)	DavePhos^a^	n.d.	n.d.

Reaction conditions: **1a** (50 μmol), CO_2_/H_2_ (2:1 mix gas, 1 atm, closed), Rh catalyst (2.0 μmol of Rh), ligand (4.0 μmol), [Ru(bpy)_3_](PF_6_)_2_ (1.0 μmol), DMA (1.0 mL), blue LEDs (425 nm), room temperature, 5 h. Yields were determined by ^1^H NMR spectroscopy with 1,1,2,2-tetrachloroethane as the internal standard; isolated yields in parenthesis.

*Me* methyl, bpy bipyridine, *Ac* acetyl, *Cy* cyclohexyl, *cod* 1,5-cyclooctadiene, *OTf* trifluoromethansulfonate.

^a^DavePhos (4.0 mol%) was used.

^b^With LiOTf (100 mol%).

^c^Under dark.

^d^Without Ru(bpy)_3_(PF_6_)_2_.

^e^Under CO_2_ (1 atm, closed) atmosphere.

^f^Under CO (1 atm, closed) atmosphere with H_2_O (500 mol%).

After an intensive screening of various kinds of ligands (Supplementary Table 1), we turned our eyes on testing various kinds of Buchwald-type ligands for hydrocarboxylation (Table 1, entries 2–6). It was found that when using DavePhos (2-dicyclohexylphosphino-2’-(*N*,*N*-dimethylamino)biphenyl) as the ligand, carboxylic acid **2a** was obtained in 35% yield with reduced amount of hydrogenation product **3a** (Table 1, entry 4). The choice of rhodium precursor also appeared to play a pivotal role in the selectivity between carboxylation and hydrogenation (Table 1, entries 7–10, see Supplementary Table 2 for details). Considering the fact that formation of rhodium-dihydride species from a Buchwald-type ligand coordinated cationic Rh-complex was very slow even under high pressure of H_2_^44^, consequently, a DavePhos ligated cationic rhodium complex, [Rh(C_2_H_4_)(DavePhos)](OTf) (Supplementary Data 1), was found to be not only highly reactive, but also selective for the hydrocarboxylation, providing the desired branched carboxylic acid **2a** in 75% yield, while only 23% yield of **3a** was generated (Table 1, entry 10). Additional amount of DavePhos ligand was critical to achieve high yield (Table 1, entry 11). A slightly higher yield of **2a** (84%) was achieved when the reaction was conducted in the presence of LiOTf (100 mol%, lithium trifluoromethanesulfonate) as an additive (Table 1, entry 12, see Supplementary Table 3 for details). We have confirmed that rhodium catalyst, light irradiation, photocatalyst, and H_2_ atmosphere were all essential to this transformation (Table 1, entries 13–16). Finally, the reaction didn’t proceed in the presence of H_2_O (500 mol%) under CO atmosphere (Table 1, entry 17), providing strong evidence that our reaction is not occurring through the rWGS pathway^39^. Although catalytic hydroxycarbonylations using CO/H_2_O are useful to obtain this type of carboxylic acids from vinyl arenes^45–47^, use of non-toxic CO_2_ gas would make this type of reaction more sustainable and environmentally friendly.

### Scope of substrates

**Fig. 2:**
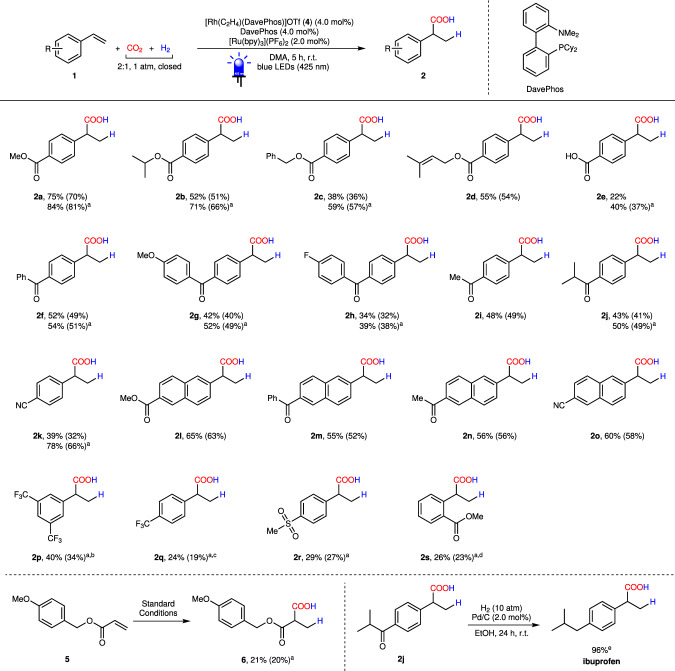
Scope of substrates. Reaction conditions: **1** or **5** (50 μmol), CO_2_/H_2_ (2:1, 1 atm, closed), [Rh(C_2_H_4_)(DavePhos)](OTf) (2.0 μmol), DavePhos (4.0 μmol), [Ru(bpy)_3_](PF_6_)_2_ (1.0 μmol), DMA (1.0 mL), blue LEDs (425 nm), room temperature, 5 h. NMR yields were shown, which were estimated by ^1^H NMR spectroscopy with 1,1,2,2-tetrachloroethane as the internal standard; isolated yields in parenthesis. ^a^With LiOTf (100 mol%). ^b^With TEMPO (10 mol%). ^c^With phenothiazine (10 mol%). ^d^10 h. ^e^Reaction conditions: **2j** (49 μmol), H_2_ (10 atm), Pd/C (10 wt% Pd, 5 wt% H_2_O) (1.0 μmol), EtOH (1.0 mL), room temperature, 24 h.

With the optimized conditions in hand (Table 1, entries 10 and 12), we next investigated the substrate scope for the hydrocarboxylation using CO_2_ and H_2_ (Fig. 2). Generally speaking, although the hydrocarboxylation proceeded smoothly under standard conditions for most electron-deficient styrene derivatives (Fig. 2, **1a**–**o**), it was found that slightly higher yields could be obtained by employing LiOTf (100 mol%) as the additive for many of the substrates. We assumed that either the carbonyl group in substrates or CO_2_ were activated by LiOTf due to its Lewis acidity^48^. Substrates bearing esters (**1a**–**d**, **1l**), ketones (**1f**–**j**, **1m**, **1n**), methoxy (**1g**), fluoride (**1h**), and nitrile (**1k**) moieties provided the target aliphatic carboxylic acids in moderate to good yields. The benzylic (**2c**) and prenylic ester (**2d**) scaffolds tolerated from potential ester hydrogenolysis under both acidic and H_2_ conditions. Owing to the base-free feature of our method, dicarboxylic acid **2e** could be prepared from 4-vinylbenzoic acid (**1e**) under CO_2_/H_2_ in synthetically useful yield (37%, with LiOTf) without protection. The hydrocarboxylation proceeded smoothly with 4-vinylbenzonitrile (**1k**) as the starting material, providing the benzylic carboxylic acid **2k** in good yield (66%), while the cyano group remained intact. As expected, hydrogenation products **3** were found to be the major side products (see Supplementary Table 6 for details). On the other hand, we also observed polymerization competing with the hydrocarboxylation and hydrogenation for some of the styrene substrates. For example, the hydrocarboxylation of trifluoromethyl substituted styrenes (**1p** and **1q**) suffered from serious polymerization under light irradiation and only low yields were obtained even with the addition of polymerization inhibitors (see Supplementary Table 5 for details). An acrylate substrate **5** was also examined under standard conditions. Unfortunately, only 20% yield of product **6** was obtained due to undesired hydrogenation and polymerization. Considering the importance of 2-arylpropionic acids in pharmaceuticals^49^, product **2j** was treated with Pd/C under a H_2_ atmosphere to provide the nonsteroidal anti-inflammatory drug ibuprofen in 96% yield.

### Mechanistic investigations

To get mechanistic insight for this reaction, some control experiments were firstly performed. Hydrocarboxylation product **2a** was not observed at all when the reaction of styrene **1a** was conducted at high temperature (110 °C) in dark in the absence of photocatalyst (see Supplementary Information, Section 6.1), proving that there is no thermal background reaction. Benzylic C–H bond carboxylation didn’t proceed at all when ethylbenzene **3a** was used as the substrate under the standard conditions (see Supplementary Information, Section 6.2), indicating that the hydrogenation-benzylic C–H bond photocarboxylation^42^ pathway is not likely.

**Fig. 3:**
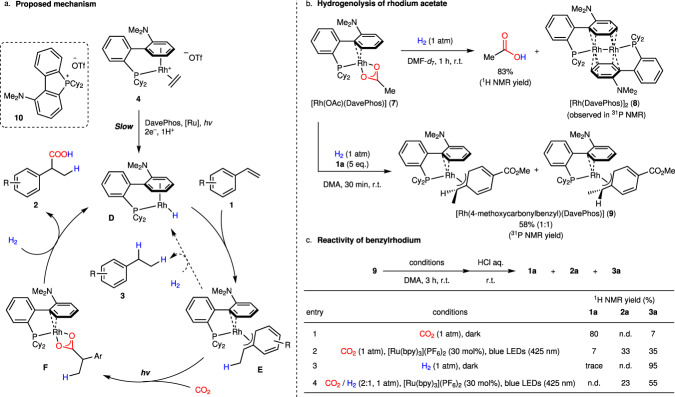
Experimental mechanistic studies. **a** Proposed mechanism. **b** Hydrogenolysis of rhodium acetate **7** with H_2_. **c** Reactivity of benzylrhodium **9**.

Then, the mechanism of this reaction is assumed based on our previous report on rhodium-catalysed hydrocarboxylation using amine as a reductant^23,24^. The postulated mechanism is shown in Fig. 3a, which consists of conversion of [Rh(C_2_H_4_)(DavePhos)](OTf) (**4**) to rhodium(I) hydride **D**, insertion of styrene to **D** to give benzylrhodium(I) **E**, carboxylation of **E** to yield rhodium(I) carboxylate **F**, and reduction of **F** to regenerate rhodium(I) hydride **D**. Under the catalytic reaction conditions, the initial generation of the active catalyst **D** from the cationic rhodium precursor **4** would involve the additional amount of DavePhos, since the hydrocarboxylation did not proceed efficiently in the absence of DavePhos (Table 1, entry 11). The kinetic time course showed the presence of an induction period (~1 h) on the generation of carboxylic acid (see Supplementary Fig. 5), suggesting slow conversion of **4** to **D**. We have observed a phosphonium salt **10** generated from DavePhos in the reaction conditions, and diisopropylethylamine or BI(OH)H as a catalytic additive instead of DavePhos was also effective for the hydrocarboxylation (see Supplementary Table 3, entries 5 and 6). These results indicated that the additional DavePhos may act as a sacrificial electron donor to reduce rhodium cation **4** to rhodium hydride **D**. On the other hand, **D** could also be generated by oxidative addition of H_2_ to Rh(I) species, followed by a base-induced deprotonation, in which DavePhos may serve as a base. To further confirm the function of DavePhos in this step, **4** and DavePhos were mixed in a H_2_ atmosphere under dark. As a result, both cationic rhodium complex **4** and DavePhos remained intact for long time (~9 h) (see Supplementary Information, Section 6.4 for details), suggesting that DavePhos is more likely to work as a sacrificial electron donor than as a base. Nevertheless, based on the facts that using a catalytic amount of Cs_2_CO_3_ instead of DavePhos also provided **2a** in 38% yield (Supplementary Table 3, entry 2), the possibility that DavePhos works as a base could not be completely ruled out (see also Supplementary Information, Section 6.5).

Next, we investigated the reactivities of the possible rhodium intermediates **D**–**F** to clarify the catalytic cycle. To confirm hydrogenolysis of rhodium carboxylate **F**, which is one of the key steps in this reaction (Fig. 1c), [Rh(OAc)(DavePhos)] (**7**) was synthesized as a model complex (see Supplementary Information, Section 6.3 for details). Treatment of **7** with H_2_ gas gave AcOH in high yield, simultaneously generating [Rh(DavePhos)]_2_ (**8**) (Supplementary Data 2), which would be produced by dehydrogenative dimerization of unstable rhodium hydride **D** (Fig. 3b). The release of carboxylic acid from rhodium carboxylate with H_2_ in the absence of base is known for a rhodium(III) acetate complex^50^, but has not been reported for rhodium(I) carboxylate complexes to the best of our knowledge. The hydrogenolysis of **7** in the presence of styrene **1a** afforded [Rh(4-methoxycarbonylbenzyl)(DavePhos)] **9**, which indicated rapid insertion of **1a** to rhodium hydride **D** before the dimerization of **D** proceeded.

The reactivity of benzylrhodium **9** towards CO_2_ and H_2_ is critical to realize selective hydrocarboxylation over hydrogenation (Fig. 1c). Similarly to our previous report^24^, **9** reacted with CO_2_ in the presence of [Ru(bpy)_3_](PF_6_)_2_ under irradiation to give carboxylic acid **2a** (Fig. 3c, entry 2), while **2a** was not observed at all without irradiation (Fig. 3c, entry 1). We have found that **9** underwent complete hydrogenolysis under a H_2_ atmosphere without irradiation (Fig. 3c, entry 3), indicating one plausible pathway to yield hydrogenated byproduct **3** in this reaction system. On the other hand, carboxylation did proceed under a CO_2_/H_2_ atmosphere upon irradiation although hydrogenolysis was faster (Fig. 3c, entry 4), which clearly demonstrates the photoenhanced carboxylation reaction in the presence of H_2_. The modest selectivity towards carboxylation observed here seems to be contradictory to the result of the catalytic reactions, where hydrocarboxylation of styrenes was preferred compared with hydrogenation. This discrepancy could be ascribed to the difference in excitation efficiency of benzylrhodium **9**. The relatively high concentration of **9** would hamper its excitation by [Ru(bpy)_3_](PF_6_)_2_ and cause hydrogenation in the investigation of the reactivity of **9** (Fig. 3c, entry 4). However, the existence of the induction period in the catalytic conditions would lower the concentration of active species **9**, making the photocarboxylation step much more selective.

In order to verify the existence of these possible intermediates in the hydrocarboxylation, we used rhodium acetate **7** or benzylrhodium **9** as a catalyst instead of cationic rhodium complex **4** (see Supplementary Information, Section 6.10 for details). Although the ratio of hydrogenation was somewhat increased, hydrocarboxylation proceeded smoothly without the addition of DavePhos, further supporting the role of these complexes as the reaction intermediates.

### Computational analysis

**Fig. 4:**
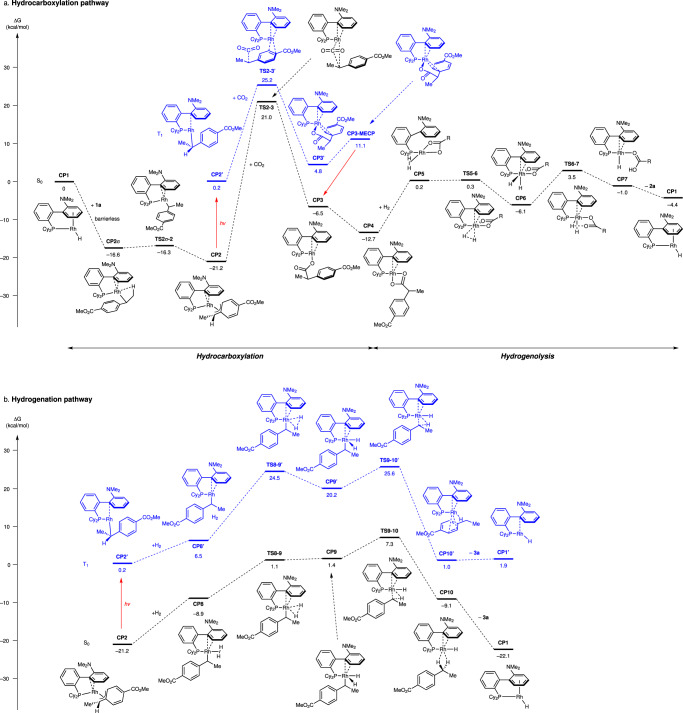
Computational studies. Free-energy diagram of **a** hydrocarboxylation and **b** hydrogenation of **1a** with RhH(Davephos) **7** (**CP1**) as a catalyst computed at the M06/SDD(Rh)&6-311 + G(d,p)(C, H, N, O, P), SMD(DMA)//LANL2DZ(Rh)&6-31 G(d,p)(C, H, N, O, P) level. Potential energy values relative to **CP1** (kcal/mol) are shown in the diagram.

To investigate the energy profile of this hydrocarboxylation, density functional theory (DFT) calculation of the proposed cycle was conducted starting from RhH(DavePhos) **CP1** and styrene **1a** (Fig. 4). The insertion of **1a** to **CP1** proceeds barrierlessly to afford σ-benzylrhodium **CP2σ** (–16.6 kcal/mol), which isomerize to give more stable π-benzylrhodium **CP2** (–21.2 kcal/mol) (Fig. 4a and Supplementary Fig. 6). The CO_2_ insertion to **CP2** needs quite high activation energy in the ground state (S_0_) (**CP2** to **TS2-3**: 42.2 kcal/mol)^51^, but proceeds smoothly in the lowest triplet excited state (T_1_) (**CP2’** to **TS2-3’**: 25.0 kcal/mol) to yield rhodium carboxylate **CP4** (–12.7 kcal/mol) after deactivation to the ground state. **CP4** undergoes hydrogenolysis via oxidative addition of H_2_ and reductive elimination of carboxylic acid **2a** with the low activation barrier (**CP4** to **TS6-7**: 16.2 kcal/mol) to regenerate **CP1** (–4.4 kcal/mol), which is endothermic by 8.3 kcal/mol. This increase of the energy is compensated in the presence of styrene **1a** by the generation of highly stable benzylrhodium **CP2**, realizing the base-free conditions. We also calculated on hydrogenation of **1a** to give alkane **3a** (Fig. 4b). **CP2** undergoes hydrogenolysis via oxidative addition of H_2_ and reductive elimination of **3a** with a slightly higher activation energy (**CP2** to **TS9-10**: 28.5 kcal/mol) than that of hydrocarboxylation in T_1_ (**CP2’** to **TS2-3’**: 25.0 kcal/mol). Notably, hydrogenation is not significantly accelerated in T_1_, where the activation barrier (**CP2’** to **TS9-10’**: 25.4 kcal/mol) is slightly higher than that of hydrocarboxylation. Although the difference of the activation energy between hydrocarboxylation and hydrogenation in T_1_ is relatively small (0.4 kcal/mol), these computational results are consistent with the experimental results, supporting the validity of the proposed catalytic cycle. The energy of product **2a** is 4.4 kcal/mol more stable than that of the starting materials (**1a** + H_2_ + CO_2_), which is consistent with the previous calculation^40^.

In summary, we have demonstrated an example of catalytic hydrocarboxylation of styrenes with CO_2_ and H_2_ under mild conditions using light energy. In the presence of rhodium/ruthenium dual catalysts and DavePhos, our catalytic system allowed the synthesis of various kinds of aliphatic carboxylic acids under ambient pressure of a CO_2_/H_2_ atmosphere at room temperature with visible light irradiation. Furthermore, we also discovered that base is no longer required under current reaction conditions. Mechanistic studies revealed that DavePhos has multiple functions in this reaction system. It not only played a role as a ligand, but also served as an electron donor, which supported the generation of Rh–H species for entering the catalytic cycle. The result of DFT calculations indicated that the activation barrier of the hydrogenation pathway is higher than that of the carboxylation, which rationalized the reason for the selectivity of hydrocarboxylation over hydrogenation under our catalytic system. We anticipate that our method would extend the toolbox for developing more sustainable catalytic methodologies in the future.

## Methods

### Procedure for the preparation of [Rh(C_2_H_4_)(DavePhos)](OTf) complex (4)

In an argon-filled glovebox, a THF solution (5 mL) of [RhCl(C_2_H_4_)_2_]_2_ (78 mg, 0.20 mmol) was stirred in a 30 mL Schlenk flask at room temperature. AgOTf (103 mg, 0.40 mmol) was added to the solution in small portions. White precipitate appeared immediately, and the mixture was kept stirring for 1 h. After all the volatiles were removed in vacuo, CH_2_Cl_2_ (5 mL) was added to the residue and the mixture was filtered with celite. The organic filtrate was evaporated again under reduced pressure to remove all the volatiles. DavePhos (157 mg, 0.40 mmol) was added to the residue and the flask was charged with CH_2_Cl_2_ (5 mL). The solution was kept stirring at room temperature for overnight to ensure complete complexation. The resulting mixture was filtered through celite again, and the filtrate was evaporated under reduced pressure. The product was recrystallized from slowly adding Et_2_O to CH_2_Cl_2_ solution to provide the target complex [Rh(C_2_H_4_)(DavePhos)](OTf) (**4**, 177 mg, 0.26 mmol, 66%) as orange yellow crystals.

### General procedure for the catalytic hydrocarboxylation of styrenes with CO_2_ and H_2_

In an argon-filled glove box, [Rh(C_2_H_4_)(DavePhos)](OTf) (**4**, 1.4 mg, 2.0 μmol), DavePhos (0.8 mg, 2.0 μmol), [Ru(bpy)_3_](PF_6_)_2_ (0.9 mg, 1.0 μmol), additive if any (0.05 mmol), and styrene substrate **1** (0.05 mmol) were placed in an oven-dried glass tube (ϕ = 1.7 cm, 18 cm). To the mixture was added anhydrous *N*,*N*-dimethylacetamide (DMA, 1.0 mL). The tube was sealed with a three-way cock and removed from the glove box. A mix gas of CO_2_/H_2_ (1 atm) was charged into the glass tube through the three-way cock. The mixture was then subjected to a blue LED (425 nm) irradiation (two sockets) with vigorous stirring. After 5 h, 1 N HCl aq. (1.0 mL) was added to the mixture, and was extracted with Et_2_O for three times. The crude mixture was analysed by ^1^H NMR to determine the yields of **2** and **3** using 1,1,2,2-tetrachloroethane as internal standard. Then the crude was diluted in Et_2_O and extracted with 1 N NaOH aq. (2.0 mL). The basic water phase was then acidified with 1 N HCl aq. until pH ≈ 1, and was extracted again with Et_2_O twice. The combined organic layer was dried over MgSO_4_. After removal of the solvent under reduced pressure, the residue was purified with preparative thin layer chromatography (PTLC) on silica gel (elution: hexane/EtOAc + 1% AcOH) to give the desired carboxylic acid product.

## Supplementary information

Supplementary Information

Description of Additional Supplementary Files

Dataset 1

Dataset 2

Dataset 3

## Data Availability

The data supporting the findings of this study are available within the paper and its Supplementary Information. Crystallographic data for the structures reported in this Article have been deposited at the Cambridge Crystallographic Data Centre, under accession code CCDC 2160650 (**4**) and CCDC 2160651 (**8**). Copies of the data can be obtained free of charge on application to CCDC, 12 Union Road, Cambridge CB2 1EZ, UK; (fax: +44(0) 1223 336 033; or e-mail: deposit@ccdc.cam.ac.uk). Cartesian coordinates of the optimized structure are available in Supplementary Data 3.
