# Supplementary material for: Catalytic direct hydrocarboxylation of styrenes with CO2 and H2
Source: Nat Commun. 2022 Dec 8;13:7584. doi: 10.1038/s41467-022-35293-3 (PMC9732006; doi:10.1038/s41467-022-35293-3)
Supplement: Supplementary file 1 — Supplementary Information [file 41467_2022_35293_MOESM1_ESM.pdf]

Supplementary Information

Catalytic Direct Hydrocarboxylation of Styrenes with CO_2 and H_2

Yushu Jin[1], Joaquim Caner[1], Shintaro Nishikawa[1], Naoyuki Toriumi[1] and Nobuharu Iwasawa[1]*

[1]*Department of Chemistry, Tokyo Institute of Technology*
O-okayama, Meguro-ku, Tokyo 152-8551, Japan
*e-mail: niwasawa@chem.titech.ac.jp

Table of Contents

1. General Information S2
2. Preparation of Starting Materials S3
 2.1 Synthesis of 3-Methylbut-2-en-1-yl 4-vinylbenzoate (**1d**) S3
 2.2 Synthesis of 4-Vinyl Ketones **1g** and **1h** S4
 2.3 Synthesis of Naphthalene Derivatives S5
3. Preparation of [Rh(C_2H_4)(DavePhos)](OTf) (**4**) S9
4. Optimization of Reaction Conditions S11
 4.1 Screening of Phosphine Ligands S11
 4.2 Screening of Rhodium Precursors S12
 4.3 Examination of Additives Using [Rh(C_2H_4)(DavePhos)](OTf) S13
 4.4 Examination of Photocatalysts S14
 4.5 Examination of Polymerization Inhibitors Using **1q** as Substrate S15
5. Rhodium-Catalyzed Hydrocarboxylation of Olefins Using CO_2 and H_2 S16
6. Experimental Mechanistic Studies S27
 6.1 Thermal Background Reaction S27
 6.2 Benzylic C–H Bond Photocarboxylation S27
 6.3 Synthesis of Rh Complexes **7–9** and Phosphonium Salt **10** S28
 6.4 Reactivity of Rh complex **4** and DavePhos under H_2 Atmosphere S32
 6.5 Generation of Phosphonium Salt **10** from DavePhos•HOTf Salt S33
 6.6 Hydrogenolysis of [Rh(OAc)(DavePhos)] (**7**) (Fig. 3b) S35
 6.7 Reactivity of Benzylrhodium Complex **9** (Fig. 3c) S36
 6.8 Time Course Analysis of Hydrocarboxylation and Hydrogenation of **1a** S37
 6.9 Observation of Phosphonium Salt **10** Under the Catalytic Reaction Conditions S38
 6.10 Catalytic Reactivity of Rhodium Acetate **7** and Benzylrhodium **8** S39
 6.11 Protonation of benzylrhodium **9** S40
7. X-ray Diffraction Analysis S41
8. Computational Details S42
9. Spectral Data S43
10. Supplementary References S83

Supplementary Methods

1. General Information

All operations except for carboxylation reactions were performed under an argon or a nitrogen atmosphere. All of the ^{1}H, ^{13}C, ^{31}P NMR and ^{19}F NMR spectra were recorded on Bruker DRX-500, Bruker Ascend-600, ECZ-500, and ECX-500 spectrometers in $CDCl_3$, CD_2Cl_2, C_6D_6, THF-d_8, CD_3CN, and acetone-d_6. Chemical shifts are expressed in parts per million (ppm) downfield from tetramethylsilane (^{1}H, $\delta = 0.00$) and are referenced to residual solvents (δ_H 7.26 and δ_C 77.0 for chloroform, δ_H 5.30 and δ_C 53.8 for dichloromethane, δ_H 7.16 and δ_C 128.06 for benzene, δ_H 3.58 and δ_C 67.21 for THF, δ_H 1.94 and δ_C 1.32 for acetonitrile, δ_H 2.05 and δ_C 206.17 for acetone). MULTIPLICITY: (s = singlet, d = doublet, dd = doublet of doublets, t = triplet, q = quartet, td = apparent triplet of doublets, sept = septet, br = broad peak). IR spectra were recorded on an Agilent Cary 630 FTIR spectrometer. Ultraviolet-visible absorption spectra were recorded with a JASCO V-650 spectrophotometer. ESI mass spectra were measured on a Bruker micrOTOF-II spectrometer. Silica Gel 60 (Kanto Chemical Co., Inc.) was used for flash column chromatography. Merck Kieselgel 60 F_{254} (0.25 mm thickness, coated on glass 20×20 cm^2) plate was used for analytical thin layer chromatography (TLC), and Wakogel B-5F coated on glass in a thickness of 1.5 mm was used for preparative TLC. CO_2 and CO_2/H_2 (33.8% H_2) gas were purchased from Taiyo Nippon Sanso Corporation. THF, Et_2O, and toluene were purified by solvent purification system of Glass-Contour. Dehydrated DMA was purchased from Wako or Sigma-Aldrich Corporation. Other materials were purchased from Sigma-Aldrich Corporation, Tokyo Chemical Industry Co. Ltd., Kanto Chemical Co., Inc., or Wako Pure Chemical Industries, Ltd. and used as received unless otherwise noted.

Photoreactions were performed in a test tube or a J-Young NMR tube in a water bath at room temperature using a Relyon Twin LED Light (3W×2, λirr. = 425 ± 15 nm).

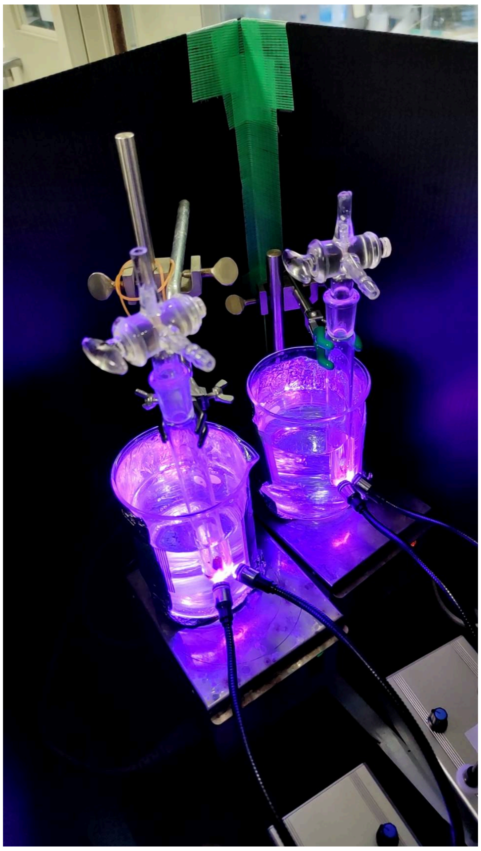

Supplementary Figure 1. Experimental Setup for the photocatalytic reactions.

2. Preparation of Starting Materials.

Compounds **1e**, **1k**, **1p**, **1q, 1r** were commercially available. **1a-c**[1], **1f**[1], **1i**[1], and **1s**[2], **1j**[3], **1l**[4], **1t**[5], and **5**[6] were prepared according to the reported procedures. Other starting materials were prepared using modified methods as described below.

2.1 Synthesis of 3-Methylbut-2-en-1-yl 4-vinylbenzoate (1d):

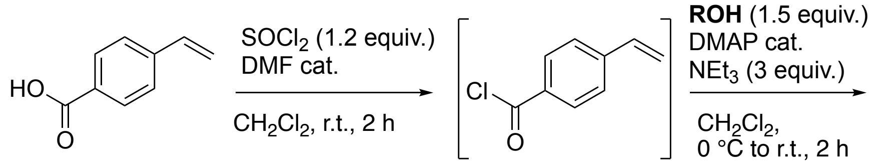

4-VBA stock solution **1d**

STEP 1. Preparation of 4-vinylbenzoyl chloride stock solution: To a flame-dried 30 mL two-necked round bottom flask were added 4-vinylbenzoic acid (1.48 g, 10 mmol), DCM (20 mL), and DMF (50 μL) under a N_2 atmosphere. Then, thionyl chloride (0.9 mL, 12 mmol, 1.2 equiv.) was added dropwise at room temperature. The resulting mixture was stirred for 2 h and concentrated under reduced pressure to remove excess thionyl chloride. The residue was dissolved in DCM (15 mL) and used as stock solution (0.67 M) for the synthesis of **1d**.

STEP 2 (1d): In a flame-dried 30 mL two-necked round bottom flask, DMAP (a spatula tip, ca. 5 mg, cat.) and 3-methyl-2-buten-1-ol (0.5 mL, 5.0 mmol, 1.5 equiv.) were dissolved in 10 mL of DCM. The mixture was cooled at 0 °C and a freshly prepared 4-vinylbenzoyl chloride stock solution (0.67 M in DCM, 5 ml, 3.3 mmol, 1 equiv.) was added. Then NEt_3 (1.4 mL, 10.1 mmol, 3 equiv.) was added dropwise. After 15 min, the reaction mixture was allowed to stir at room temperature. After 2 h, the solution was diluted with EtOAc and quenched with 1N HCl aq. After separation, the aqueous layer was extracted with EtOAc twice. The combined organic layer was washed with water and dried over Na_2SO_4, and the solvent was removed under reduced pressure. The resulting crude mixture was purified by silica gel column chromatography (hexane/AcOEt = 20/1) to furnish the desired product **1d** as dense colorless oil (613 mg, 2.8 mmol, 85%).

	**1d**
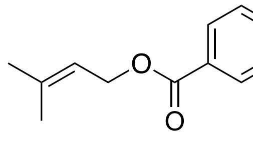 **1d**	^{1}H NMR (600 MHz, $CDCl_3$) δ 1.77 (s, 3H), 1.79 (s, 3H), 4.81 (d, J = 7.2 Hz, 2H), 5.37 (dd, J = 10.9, 0.5 Hz, 1H), 5.45-5.49 (m, 1H), 5.85 (dd, J = 17.6, 0.6 Hz, 1H), 6.74 (dd, J = 17.6, 10.9 Hz, 1H), 7.45 (d, J = 8.3 Hz, 2H). 8.00 (d, J = 8.3 Hz, 2H).<br>^{13}C NMR (151 MHz, $CDCl_3$) δ 18.1, 25.8, 61.9, 116.4, 118.7, 126.0, 129.3, 129.9, 136.1, 139.1, 141.8, 166.4.<br>HRMS (ESI-TOF): m/z calcd for $C_{14}H_{16}O_2Na^+$ $[M+Na]^+$: 239.1043, found: 239.1053.<br>FT-IR (ATR): 2973, 2932, 2857, 1711, 1607, 1444, 1403, 1374, 1332, 1310, 1170, 1098, 1068, 1015, 990, 913, 859, 751, 714 cm^{-1}.

2.2 Synthesis of 4-Vinyl Ketones 1g and 1h:

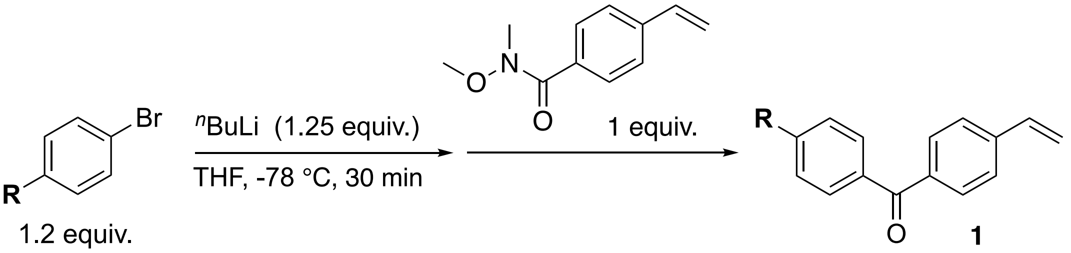

Stock solution of *N*-methoxy-*N*-methyl-4-vinylbenzamide:[1] A 0.4 M solution of *N*-methoxy-*N*-methyl-4-vinylbenzamide was prepared by dissolving 773 mg of *N*-methoxy-*N*-methyl-4-vinylbenzamide in 10 mL of anhydrous THF under argon.

2.2.1 Synthesis of 4-methoxyphenyl-(4-vinylphenyl)methanone (1g) [24992-90-6]: To a flame-dried 30 mL two-necked round bottom flask were added 4-bromoanisole (300 µL, 2.4 mmol, 1.2 equiv.) and THF (5 mL) under an Ar atmosphere and the solution was cooled to -78 °C. Then, ^{n}BuLi (1.56 M in hexane, 1.6 mL, 2.5 mmol, 1.25 equiv.) was added dropwise to the mixture and stirred for 30 min at -78 °C. To this freshly prepared organolithium compound, stock solution of *N*-methoxy-*N*-methyl-4-vinylbenzamide (0.4 M in THF, 5 mL, 2.0 mmol, 1 equiv.) was added dropwise over 10 min. After 30 min at -78 °C the reaction was quenched by addition of 2 mL of 0.5 N HCl aqueous solution and allowed to warm up to room temperature. Then, water was added to the reaction mixture and extracted three times with AcOEt. The combined organic layer was dried over Na_2SO_4 and concentrated in vacuo. The product was purified by silica gel column chromatography (hexane/AcOEt 93:7 to 90:10) to furnish the desired compound **1g** (398 mg, 1.67 mmol, 84%) as white solid.

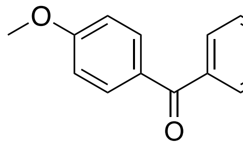 **1g**	**1g** was a known compound in literature, and its spectral data were in good agreement with literature values.[7]
	^{1}H NMR (600 MHz, $CDCl_3$) δ 3.89 (s, 3H), 5.39 (dd, *J* = 10.8, 0.6 Hz, 1H), 5.88 (dd, *J* = 18.1, 0.6 Hz, 1H), 6.78 (dd, *J* = 18.1, 10.8 Hz, 1H), 6.96 (d, *J* = 9.0 Hz, 2H), 7.50 (d, *J* = 8.4 Hz, 2H), 7.74 (d, *J* = 8.4 Hz, 2H), 7.82 (d, *J* = 9.0 Hz, 2H). ^{13}C NMR (151 MHz, $CDCl_3$) δ 55.5, 113.5, 116.3, 125.9, 130.2, 130.3, 132.4, 136.1, 137.4, 141.0, 163.2, 195.0.

2.2.2 4-Fluorophenyl-(4-vinylphenyl)methanone (1h) [848466-66-0]:
To a flame-dried 30 mL two-necked round bottom flask were added 4-bromofluorobenzene (270 µL, 2.4 mmol, 1.2 equiv.) and THF (5 mL) under an Ar atmosphere and the solution was cooled to -78 °C. Then, ^{n}BuLi (1.56 M in hexane, 1.6 mL, 2.5 mmol, 1.25 equiv.) was added dropwise to the mixture and stirred for 30 min at -78 °C. To this freshly prepared organolithium compound, stock solution of *N*-methoxy-*N*-methyl-4-vinylbenzamide (0.4 M in THF, 5 mL, 2.0 mmol, 1 equiv.) was added dropwise over 10 min. After 30 min at -78 °C the reaction was quenched by addition of 2 mL of 0.5 N HCl aqueous solution and allowed to warm up to room temperature. Then, water was added to the reaction mixture and extracted three times with AcOEt. The combined organic layer was dried over Na_2SO_4 and concentrated in vacuo. The product was purified by silica gel column chromatography (hexane/AcOEt 20:1) to furnish the desired compound **1h** (394 mg, 1.73 mmol, 87%) as white solid.

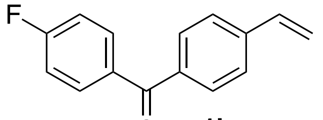 O 1h	**1h**
	^{1}H NMR (600 MHz, CDCl$_3$) δ 5.42 (dd, J = 10.8, 0.6 Hz, 1H), 5.90 (dd, J = 17.4, 0.6 Hz, 1H), 6.79 (dd, 17.4, 10.8 Hz, 1H), 7.16-7.18 (m, 2H), 7.51 (d, J = 8.3 Hz, 2H), 7.75 (d, J = 8.3 Hz, 2H), 7.82-7.86 (m, 2H).<br>^{13}C NMR (151 MHz, CDCl$_3$) δ 115.4 (d, $J_{C\text{-}F}$ = 21.9 Hz), 116.7, 126.1, 130.4, 132.5 (d, $J_{C\text{-}F}$ = 8.9 Hz), 133.9 (d, $J_{C\text{-}F}$ = 3.0 Hz), 135.9, 136.5, 141.6, 165.3 (d, $J_{C\text{-}F}$ = 254.1 Hz), 194.7.<br>^{19}F NMR (466 MHz, CDCl$_3$) δ -105.8.<br>HRMS (ESI-TOF): *m/z* calcd for $C_{15}H_{11}FONa^+$ $[M+Na]^+$: 249.0686, found: 249.0690.<br>FT-IR (ATR): 3094, 3066, 3032, 3012, 2991, 1646, 1592, 1498, 1400, 1297, 1277, 1224, 1183, 1144, 1116, 1100, 1014, 991, 967, 923, 859, 842, 814, 773, 734, 687 cm^{-1}.

2.3 Synthesis of Naphthalene Derivatives:

1-(6-Vinylnaphthalen-2-yl)ethan-1-one (1n) [259199-33-2]:

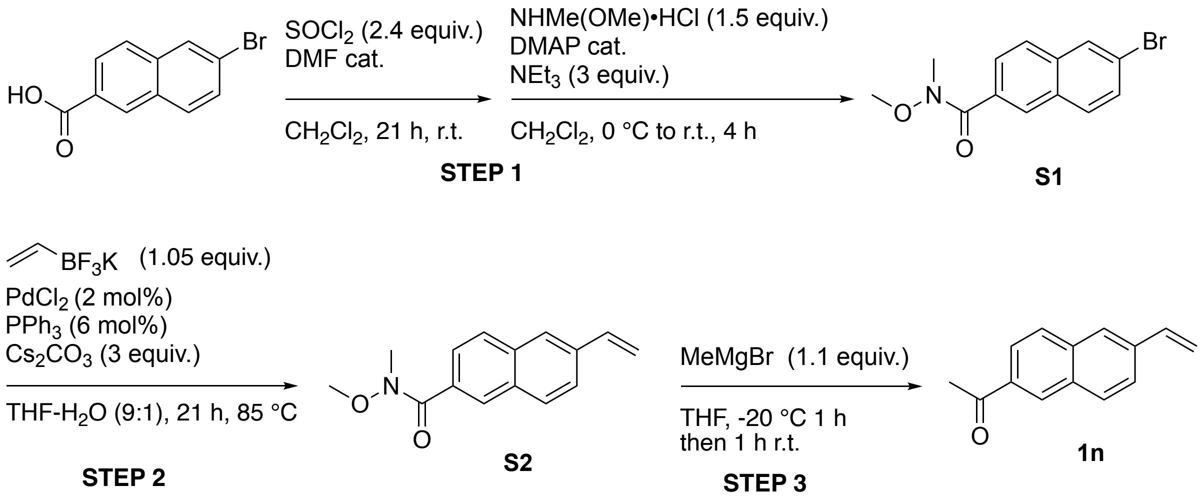

STEP 1 (S1, [861880-64-0]):

To a flame-dried 100 mL two-necked round bottom flask were added 6-bromo-2-naphthoic acid (2.51 g, 10.0 mmol), DCM (60 mL), and DMF (100 µL, cat.) under a N_2 atmosphere. Then, thionyl chloride (1.8 mL, 24.8 mmol, 2.5 equiv.) was added dropwise at room temperature. The resulting slurry was stirred for 21 h and concentrated under reduced pressure to remove the excess thionyl chloride. The above acyl chloride stock solution was added *via* cannula to a solution of NHMe(OMe)•HCl (1.46 g, 15.0 mmol, 1.5 equiv.) and DMAP (10 mg, cat.) in DCM (20 mL) prepared in a 100 mL two-necked round bottom flask under a N_2 atmosphere. Then, NEt_3 (4.2 mL, 30 mmol, 3 equiv.) was added dropwise at 0 °C. The reaction mixture was allowed to stir at room temperature. After 4 h, the solution was quenched with 1 N HCl aq. (20 mL) and diluted with CH_2Cl_2 and H_2O. After separation, the aqueous layer was extracted with CH_2Cl_2 (2 × 20 mL). The combined organic layer was dried over Na_2SO_4 and evaporated under reduced pressure. The resulting crude product was purified by silica gel column chromatography (hexane/AcOEt = 3:1 to 2:1) to afford **S1** (2.60 g; 8.8 mmol, 88%) as pale brownish dense oil.

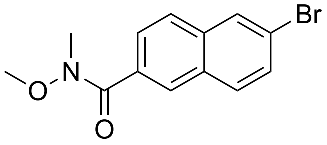<br>**S1**	**S1**
	^{1}H NMR (600 MHz, $CDCl_3$) δ 3.42 (s, 3H), 3.55 (s, 3H), 7.60 (dd, J = 9.0, 1.8 Hz, 1H), 7.74-7.80 (m, 3H), 8.03 (d, J = 1.8 Hz, 1H), 8.19 (s, 1H).<br>^{13}C NMR (151 MHz, $CDCl_3$) δ 33.7, 61.2, 121.6, 126.2, 126.7, 128.6, 129.8, 130.0, 130.4, 130.9, 131.8, 135.2, 169.4.<br>HRMS (ESI-TOF): *m/z* calcd for $C_{13}H_{12}BrNO_2Na^+$ $[M+Na]^+$: 315.9944, found: 315.9951.<br>FT-IR (ATR): 2958, 2933, 2902, 2816, 1638, 1588, 1459, 1414, 1374, 1349, 1267, 1202, 1174, 1127, 1062, 988, 952, 889, 807, 732, 697 cm^{-1}.

STEP 2 (S2):

To a 30 mL Schlenk tube, 6-bromo-*N*-methoxy-*N*-methyl-2-naphthamide (**S1**, 1.32 g, 4.5 mmol, 1 equiv.), $PdCl_2$ (16 mg, 0.09 mmol, 2 mol%), PPh_3 (71 mg, 0.27 mmol, 6 mol%), Cs_2CO_3 (4.4 g, 13.5 mmol), and potassium vinyltrifluoroborate (633 mg, 4.7 mmol, 1.05 equiv.) were added sequentially. The headspace was replaced twice with N_2, and 9 mL of degassed THF-H_2O (9:1) was added. The reaction mixture was heated at 85 °C for 21 h, cooled to r.t., and diluted with H_2O and Et_2O. The aqueous phase was extracted with ether twice, the combined organic layers were dried over Na_2SO_4 and the solvent removed under reduced pressure. The crude product was purified by silica gel chromatography (hexane/AcOEt 20:1) to yield the title compound **S2** as a pale-yellow waxy solid (712 mg, 2.95 mmol, 65%).

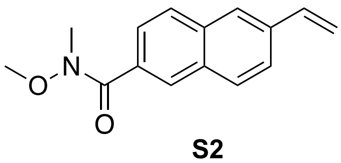<br>**S2**	**S2**
	^{1}H NMR (600 MHz, $CDCl_3$) δ 3.41 (s, 3H), 3.56 (s, 3H), 5.38 (d, J = 10.8 Hz), 5.90 (d, J = 17.4 Hz, 1H), 6.89 (dd, J = 17.4, 10.8 Hz, 1H), 7.67 (d, J = 8.4 Hz, 1H), 7.72-7.77 (m, 2H), 7.80-7.87 (m, 2H), 8.19 (s, 1H).<br>^{13}C NMR (151 MHz, $CDCl_3$) δ 33.8, 61.1, 115.1, 123.8, 125.5, 126.0, 127.7, 128.4, 129.0, 131.3, 132.1, 134.4, 136.5, 136.6, 169.8.<br>HRMS (ESI-TOF): *m/z* calcd for $C_{15}H_{16}NO_2^+$ $[M+H]^+$: 242.1176, found: 242.1169.<br>FT-IR (ATR): 3004, 2974, 2913, 1623, 1593, 1482, 1414, 1385, 1185, 1116, 1020, 993, 915, 896, 818, 744, 667 cm^{-1}.

STEP 3 (1n, [442850-90-0]):

To a flame-dried 30 mL two-necked round bottom flask were added *N*-methoxy-*N*-methyl-6-vinyl-2-naphthamide (**S2**, 320 mg, 1.33 mmol, 1 equiv.) and THF (6 mL) under a N_2 atmosphere and the solution was cooled to -20 °C. Then, MeMgBr (3.0 M in Et_2O, 0.5 mL, 1.5 mmol, 1.2 equiv.) was added dropwise to the mixture. After 1 h at -20 °C, the reaction was stirred at room temperature for another 1 h. The reaction was quenched with saturated NH_4Cl aq., diluted with water, and extracted with AcOEt three times. The combined organic layer was dried over $MgSO_4$ and concentrated in vacuo. The crude product was purified by silica gel column chromatography (hexane/AcOEt 9:1) to furnish the desired compound **1n** (240 mg, 1.22 mmol, 92%) as off-white waxy solid.

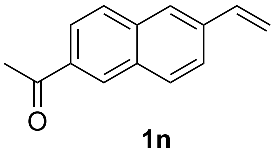<br>**1n**	**1n**<br>^{1}H NMR (600 MHz, $CDCl_3$) δ 2.72 (s, 3H), 5.42 (d, J = 10.8 Hz, 1H), 5.94 (d, J = 17.4 Hz, 1H), 6.89 (dd, J = 17.4 Hz, 10.8 Hz, 1H), 7.70 (dd, J = 7.8 Hz, 1.8 Hz, 1H), 7.77 (s, 1H), 7.85 (d, 1H, J = 8.4 Hz), 7.91 (d, J = 8.4 Hz, 1H), 8.01 (dd, J = 8.4, 1.8 Hz, 1H), 8.42 (s, 1H).<br>^{13}C NMR (151 MHz, $CDCl_3$) δ 26.7, 115.7, 124.2, 124.4, 126.0, 128.5, 129.7, 129.8, 132.1, 134.4, 135.8, 136.5, 137.6, 197.9.<br>HRMS (ESI-TOF): m/z calcd for $C_{14}H_{12}ONa^+$ $[M+Na]^+$: 219.0780, found: 219.0800.<br>FT-IR (ATR): 2954, 2917, 2850, 1675, 1625, 1599, 1465, 1407, 1377, 1358, 1262, 1221, 1192, 1128, 1064, 1017, 962, 934, 908, 895, 857, 829, 794, 726, 676 cm^{-1}.

Phenyl(6-vinylnaphthalen-2-yl)methanone (1m):

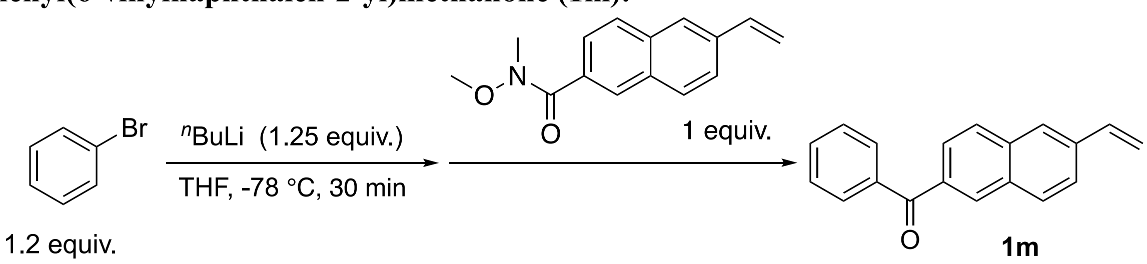

To an oven-dried 20 mL two-necked round bottom flask were added bromobenzene (25 µL, 0.24 mmol, 1.2 equiv.) and THF (3 mL) under a N_2 atmosphere and the solution was cooled to -78 °C. Then, ^{n}BuLi (1.57 M in hexanes; 0.16 mL, 0.25 mmol, 1.25 equiv.) was added dropwise to the mixture, and the mixture was stirred for 30 min at -78 °C. To this freshly prepared organolithium compound, a solution of *N*-methoxy-*N*-methyl-6-vinyl-2-naphthamide (**S2**) (48 mg, 0.1 M in THF, 2 mL, 0.2 mmol, 1 equiv.) was added dropwise over 5 min. After 30 min at -78 °C the reaction was quenched by the addition of 1 mL of 0.5 N HCl aqueous solution. After warming up to room temperature, water was added to the reaction mixture, and the mixture was extracted three times with AcOEt. The combined organic layer was dried over $MgSO_4$ and concentrated in vacuo. The crude was purified by silica gel column chromatography (hexane/EtOAc 20:1) to furnish the desired compound **1m** (41 mg, 0.16 mmol, 80%) as off-white solid.

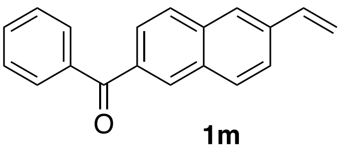<br>**1m**	**1m**<br>^{1}H NMR (600 MHz, $CDCl_3$) δ 5.42 (d, J = 11.4 Hz, 1H), 5.95 (d, J = 17.4 Hz, 1H), 6.91 (dd, J = 17.4, 11.4 Hz, 1H), 7.49-7.55 (m, 2H), 7.59-7.64 (m, 1H), 7.70 (dd, J = 8.4, 1.2 Hz, 1H), 7.82 (s, 1H), 7.84-7.96 (m, 5H), 8.23 (s, 1H).<br>^{13}C NMR (151 MHz, $CDCl_3$) δ 115.7, 124.2, 126.1, 126.3, 128.35, 128.38, 129.7, 130.1, 131.6, 131.9, 132.4, 134.8, 135.6, 136.6, 137.5, 137.9, 196.6.<br>HRMS (ESI-TOF): m/z calcd for $C_{19}H_{14}ONa^+$ $[M+Na]^+$: 281.0937, found: 281.0953.<br>FT-IR (ATR): 3081, 3058, 3012, 2919, 2849, 1653, 1617, 1597, 1575, 1497, 1476, 1444, 1424, 1379, 1340, 1308, 1271, 1247, 1213, 1176, 1161, 1116, 1073, 992, 971, 948, 915, 895, 824, 796, 723, 695, 673 cm^{-1}.

6-Vinyl-2-naphthalonitrile (1o) [259199-33-2]:

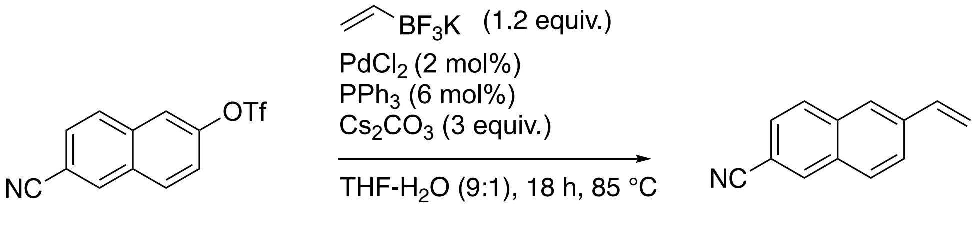

1o

To a 10 mL screw-cap test tube, 6-cyanonaphthalen-2-yl trifluoromethanesulfonate[8] (301 mg, 1.0 mmol, 1 equiv.), $PdCl_2$ (3.5 mg, 0.02 mmol, 2 mol%), PPh_3 (16 mg, 0.06 mmol, 6 mol%), Cs_2CO_3 (990 mg, 3.0 mmol, 3 equiv.), and potassium vinyltrifluoroborate (160 mg, 1.2 mmol, 1 equiv.) were added sequentially. The headspace was replaced twice with N_2, and 2 mL of degassed THF-H_2O (9:1) was added. The reaction mixture was heated at 85 °C for 18 h, cooled to room temperature, and diluted with H_2O and Et_2O. The aqueous phase was extracted with ether twice. The combined organic layer was dried over Na_2SO_4, and the solvent was removed under reduced pressure. The crude product was purified by silica gel chromatography (hexane/AcOEt 20:1) to yield the title compound **1o** as white solid (118 mg, 0.66 mmol, 66%).

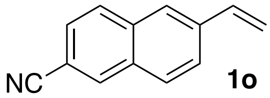 **1o**	**1o** was a known compound in literature, and its spectral data were in good agreement with literature values.[9]
	^{1}H NMR (600 MHz, $CDCl_3$) δ 5.46 (d, J = 10.8 Hz, 1H), 5.96 (d, 18.0 Hz, 1H), 6.89 (dd, J = 18.0, 10.8 Hz, 1H), 7.59 (dd, J = 8.4, 1.2 Hz, 1H), 7.75 (dd, J = 9.0, 1.2 Hz, 1H), 7.78 (s, 1H), 7.86 (d, J = 9.0 Hz, 1H), 7.88 (d, J = 8.4 Hz, 1H), 8.19 (s, 1H).

3. Preparation of [Rh(C_2H_4)(DavePhos)](OTf) (4)

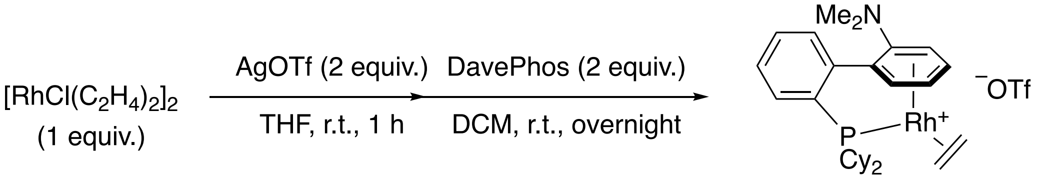

[Rh(C_2H_4)(DavePhos)](OTf) **4**
66%

The titled rhodium complex was prepared following the reference procedure.[10]
In an argon filled glovebox, a THF solution (5 mL) of [RhCl(C_2H_4)$_2$]$_2$ (78 mg, 0.20 mmol) was stirred in a 30 mL Schlenk flask at room temperature. AgOTf (103 mg, 0.40 mmol) was added to the solution in small portions. White precipitate appeared immediately, and the mixture was kept stirring for 1 h. After all the volatiles were removed in vacuo, CH_2Cl_2 (5 mL) was added to the residue and the mixture was filtered with Celite. The organic filtrate was evaporated again under reduced pressure to remove all the volatiles. DavePhos (157 mg, 0.40 mmol) was added to the residue and the flask was charged with CH_2Cl_2 (5 mL). The solution was kept stirring at room temperature overnight to ensure complete complexation. The resulting mixture was filtered through Celite again, and the filtrate was evaporated under reduced pressure. The product was recrystallized by slowly adding Et_2O to DCM solution to provide the target complex [Rh(C_2H_4)(DavePhos)](OTf) (**4**, 177 mg, 0.26 mmol, 66%) as orange yellow crystals. Block shaped yellow crystals suitable for X-ray single crystal analysis were grown by vapor diffusion of Et_2O into a CH_2Cl_2 solution of **4** under an Ar atmosphere.

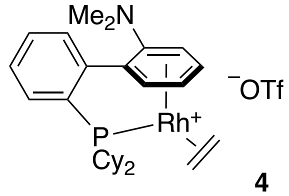 **4**	**4**
	^{1}H NMR (600 MHz, CD_2Cl_2): δ 0.60-0.70 (m, 1H), 0.91-1.00 (m, 1H), 1.02-1.36 (m, 8H), 1.43-1.51 (m, 1H), 1.57-1.85 (m, 8H), 1.90-1.99 (m, 1H), 2.03-2.11 (m, 2H), 2.72 (s, 6H), 3.08 (s, 2H), 3.17 (s, 2H), 5.35-5.40 (m, 1H), 6.52 (d, J = 7.2 Hz, 2H), 6.66 (d, J = 2.4 Hz, 1H), 7.45-7.55 (m, 2H), 7.60-7.65 (m, 1H), 7.78 (d, J = 7.2 Hz, 1H).<br>^{13}C NMR (151 MHz, CD_2Cl_2): δ 26.3 (d, J = 21.1 Hz), 26.7 (d, J = 10.6 Hz), 27.27 (d, J = 10.6 Hz), 27.32 (d, J = 13.6 Hz), 27.6 (d, J = 13.6 Hz), 28.1, 28.6, 29.0, 35.0 (d, J = 25.7 Hz), 36.1 (d, J = 24.2 Hz), 43.2, 43.3, 89.6, 93.9 (d, J = 10.6 Hz), 101.3, 105.6, 109.1, 120.4, 121.5 (q, J = 320.1 Hz), 129.0 (d, J = 6.0 Hz), 131.1 (d, J = 12.1 Hz), 131.5, 131.9, 141.6, 142.9, 143.1, 146.1 (d, J = 18.1 Hz).<br>^{31}P{^{1}H} NMR (202 MHz, $CDCl_3$): δ 71.1 (d, J = 190.7 Hz).<br>^{19}F NMR (471 MHz, THF-d_8): δ -80.7 (s).<br>HRMS (ESI): m/z calcd for $C_{26}H_{36}NPRh^+$ [M–C_2H_4–OTf]$^+$: 496.1635, found 496.1642.<br>IR(ATR): 3077, 2935, 2854, 1541, 1499, 1426, 1261, 1223, 1203, 1146, 1027 cm^{-1}.

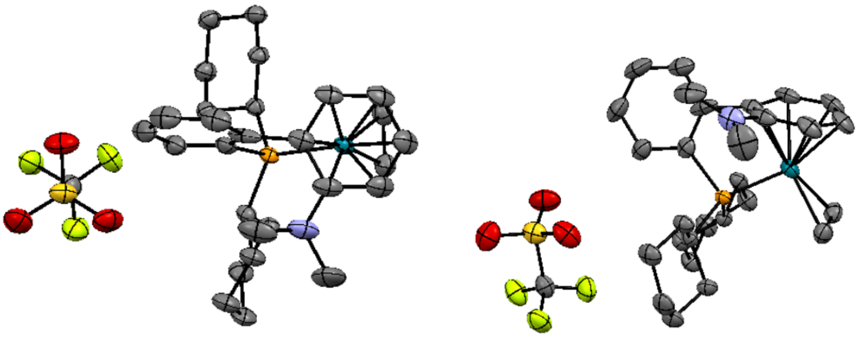

Supplementary Figure 2. X-ray structure of **4** (left: top view, right: side view). Thermal ellipsoids are set at 50% probability. H atoms are omitted for clarity.

4. Optimization of Reaction Conditions

4.1 Screening of Phosphine Ligands

General procedure: In an argon filled glovebox, $[Rh(OAc)(C_2H_4)_2]_2$ (0.4 mg, 1 μmol, 2 μmol Rh), and phosphine ligand (4 μmol of P) were premixed in 0.5 mL of DMA for about 10 min in an oven dried glass tube (ϕ = 1.7 cm, 18 cm). $[Ru(bpy)_3](PF_6)_2$ (0.9 mg, 1 μmol) and methyl 4-vinylbenzoate (**1a**, 8.1 mg, 0.05 mmol) were added to the mixture with extra 0.5 mL of DMA. The tube was sealed with a three-way cock and removed from the glovebox. A mix gas of CO_2/H_2 (2:1, 1 atm) was charged into the glass tube through the three-way cock. The mixture was then subjected to a blue LED (425 nm) irradiation (two sockets) with vigorous stirring. After 5 h, the reaction was diluted with Et_2O (2.0 mL) and 1 N HCl aq. (2.0 mL) was added. The water phase was extracted with Et_2O three times. The combined organic layer was dried over $MgSO_4$. After removal of the solvent under reduced pressure, 1,1,2,2-tetrachloroethane (10 μL) was added to the crude product as an internal standard. The yields were calculated based on the integration of ^1H NMR spectra.

Supplementary Table 1. Screening of Phosphine Ligands

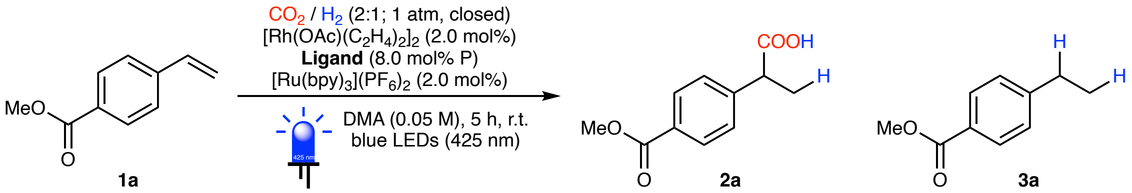

entry	ligand	yield of **2a** (%)[a]	yield of **3a** (%)[a]
1[b]	no ligand	n.d.	65
2	$P(4\text{-}CF_3\text{-}C_6H_4)_3$	5	74
3	$P(4\text{-}CF_3\text{-}C_6H_4)_3$ (12 mol%P)	5	85
4	PPh_3	9	70
5	$P(4\text{-}MeO\text{-}C_6H_4)_3$	trace	86
6	PCy_3	n.d.	12
7	Xantphos	13	79
8	DPEphos	1	65
9	DPPE	trace	90
10	JohnPhos	7	6
11	SPhos	9	36
12	XPhos	4	67
13	RuPhos	11	80
14	CyJohnPhos	16	61
**15**	**DavePhos**	**35**	**48**
16	DavePhos (4 mol% P)	22	35
17	^{t}BuDavePhos	trace	99
18	PhDavePhos	3	30
19	*p*-DavePhos	16	-[c]
20	*p*-DavePhos	15	77
21	CPhos	17	38

[a] ^1H NMR yield. [b] 100 mol% of LiOTf was added. [c] Not determined.
n.d. = not detected by ^1H NMR.

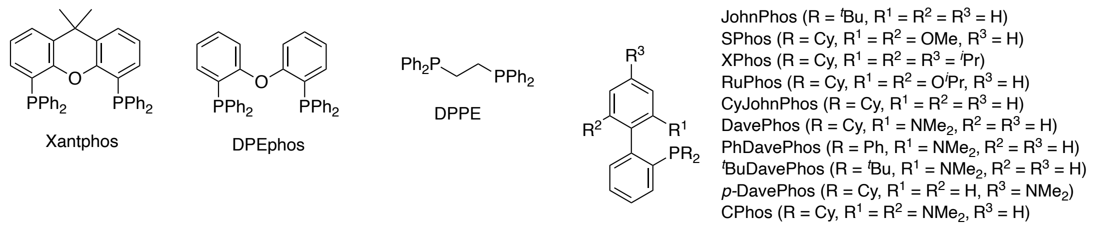

4.2 Screening of Rhodium Precursors

General procedure: In an argon filled glovebox, rhodium precursor (2 μmol Rh), DavePhos (1.6 mg (4 μmol) or 0.8 mg (2 μmol); total amount of P 4 μmol) were premixed in 0.5 mL of DMA for about 10 min in an oven dried glass tube (ϕ = 1.7 cm, 18 cm). $[Ru(bpy)_3](PF_6)_2$ (0.9 mg, 1 μmol) and methyl 4-vinylbenzoate (**1a**, 8.1 mg, 0.05 mmol) were added with extra 0.5 mL of DMA. The tube was sealed with a three-way cock and removed from the glovebox. A mix gas of CO_2/H_2 (2:1, 1 atm) was charged into the glass tube through the three-way cock. The mixture was then subjected to a blue LED (425 nm) irradiation (two sockets) with vigorous stirring. After 5 h, the reaction was diluted with Et_2O (2.0 mL) and 1 N HCl aq. (2.0 mL) was added. The water phase was extracted with Et_2O three times. The combined organic layer was dried over $MgSO_4$. After removal of the solvent under reduced pressure, 1,1,2,2-tetrachloroethane (10 μL) was added to the crude product as an internal standard. The yields were calculated based on the integration of ^1H NMR spectra.

Supplementary Table 2. Screening of Rhodium Precursors

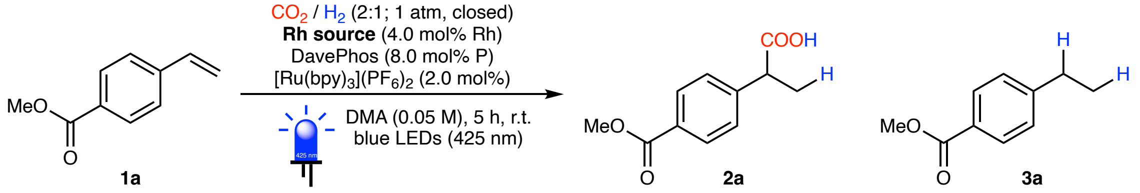

entry	Rh source	yield of **2a** (%)[a]	yield of **3a** (%)[a]
1	$[RhCl(C_2H_4)_2]_2$	4	97
2	$[Rh(cod)_2]OTf$	18	80
3	$[Rh(nbd)_2]BF_4$	25	42
**4**	**$[Rh(C_2H_4)(DavePhos)]OTf$**	**75 (70)**	**23**
5[b]	$[Rh(C_2H_4)(DavePhos)]OTf$	10	9
6	[Rh(OAc)(DavePhos)]	38	27
7[b]	[Rh(OAc)(DavePhos)]	10	38

[a] ^1H NMR yield. Isolated yield in parenthesis. [b]No extra DavePhos was added (total P amount 4 mol%; Rh:P 1:1).

4.3 Examination of Additives Using $[Rh(C_2H_4)(DavePhos)](OTf)$

General procedure: In an argon filled glove box, $[Rh(C_2H_4)(DavePhos)](OTf)$ (1.4 mg, 2 µmol), DavePhos (0.8 mg, 2 µmol), $[Ru(bpy)_3](PF_6)_2$ (0.9 mg, 1 µmol), additive (X mol%), and methyl 4-vinylbenzoate (**1a**, 8.1 mg, 0.05 mmol) were placed in an oven dried glass tube (ϕ = 1.7 cm, 18 cm). To the mixture was added anhydrous *N*,*N*-dimethylacetamide (DMA, 1.0 mL). The tube was sealed with a three-way cock and removed from the glove box. A mix gas of CO_2/H_2 (2:1, 1 atm) was charged into the glass tube through the three-way cock. The mixture was then subjected to a blue LED (425 nm) irradiation (two sockets) with vigorous stirring. After 5 h, the reaction mixture was diluted with Et_2O and 1 N HCl aq. was added. The water phase was extracted with Et_2O three times. The combined organic layer was dried over $MgSO_4$. After removal of the solvent under reduced pressure, 1,1,2,2-tetrachloroethane was added to the crude product as an internal standard. The yields were calculated based on the integration of ^1H NMR spectra.

Supplementary Table 3. Examination of Additives Using $[Rh(C_2H_4)(DavePhos)](OTf)$

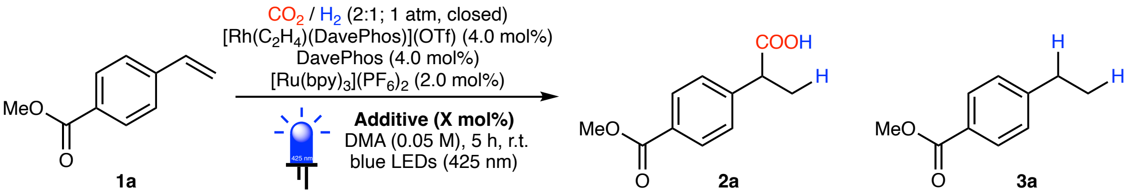

entry	additive	yield of **2a** (%)[a]	yield of **3a** (%)[a]
1	Cs_2CO_3 (4.5 mol%)	32	25
2[b]	Cs_2CO_3 (4 mol%)	38	49
3	Cs_2CO_3 (20 mol%)	47	61
4	Cs_2CO_3 (110 mol%)	28	22
5[b]	$^iPr_2NEt$ (4 mol%)	47	30
6[b]	BI(OH)H (4 mol%)	35	54
7	KH_2PO_4 (20 mol%)	48	63
8	K_3PO_4 (110 mol%)	23	18
9	PhCOOH (10 mol%)	69	26
10	NaOTf (100 mol%)	65	20
11	$La(OTf)_3$ (20 mol%)	n.d.	trace
12	LiOTf (20 mol%)	69	31
13	LiOTf (50 mol%)	83	24
**14**	**LiOTf (100 mol%)**	**84(81)**	**16**
15	LiOTf (200 mol%)	65	27
16	KOTf (100 mol%)	80	19

[a] ^1H NMR yield. Isolated yield in parenthesis. [b] Without extra DavePhos. n.d. = not detected by ^1H NMR.

4.4 Examination of Photocatalysts

General procedure: In an argon filled glove box, [Rh(C_2H_4)(DavePhos)](OTf) (1.4 mg, 2 μmol), DavePhos (0.8 mg, 2 μmol), photocatalyst (1 μmol), LiOTf (7.8 mg, 0.05 mmol), and methyl 4-vinylbenzoate (**1a**, 8.1 mg, 0.05 mmol) were placed in an oven dried glass tube (ϕ = 1.7 cm, 18 cm). To the mixture was added anhydrous *N*,*N*-dimethylacetamide (DMA, 1.0 mL). The tube was sealed with a three-way cock and removed from the glove box. A mix gas of CO_2/H_2 (2:1, 1 atm) was charged into the glass tube through the three-way cock. The mixture was then subjected to a blue LED (425 nm) irradiation (two sockets) with vigorous stirring. After 5 h, the reaction mixture was diluted with Et_2O and 1 N HCl aq. was added. The water phase was extracted with Et_2O three times. The combined organic layer was dried over $MgSO_4$. After removal of the solvent under reduced pressure, 1,1,2,2-tetrachloroethane was added to the crude product as an internal standard. The yields were calculated based on the integration of ^1H NMR spectra.

Supplementary Table 4. Examination of Photocatalysts

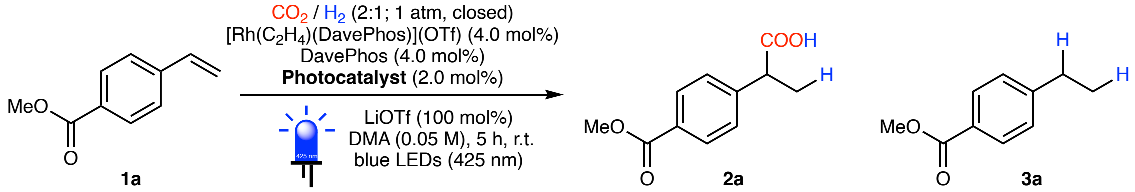

entry	photocatalyst	triplet *E* (kcal/mol)	E^*_{red} (V)[a]	E^*_{ox} (V)[a]	yield of **2a** (%)[b]	yield of **3a** (%)[b]
**1**	**[Ru(bpy)$_3$](PF$_6$)$_2$**	49.0	+0.77	–0.81	**84 (81)**	**16**
2	*fac*-Ir(ppy)$_3$	58.1	+0.31	–1.73	21	19
3	[Ir(ppy)$_2$(dtbpy)](PF$_6$)	49.2	+0.66	–0.96	65	14
4	[Ir(dF(CF$_3$)ppy)$_2$(dtbpy)](PF$_6$)	61.8	+1.21	–0.89	37	26
5	[Ir(ppy)$_2$(phen)](PF$_6$)	51.6	+1.06	–1.05	48	n.d.
6	4CzIPN	54.5	+1.35	–1.14	50	7

[a] vs SCE. [b] ^1H NMR yield. Isolated yield in parenthesis.

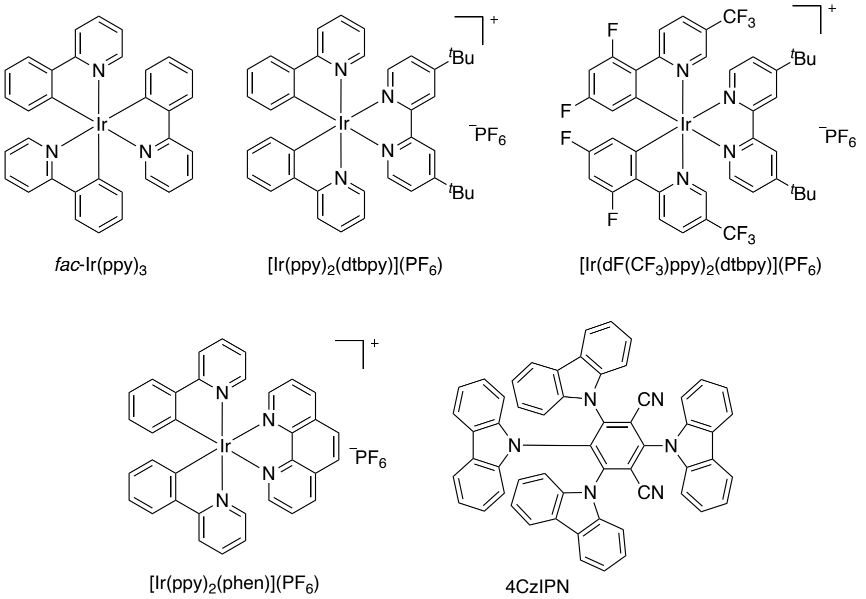

4.5 Examination of Polymerization Inhibitors Using 1q as Substrate

General procedure: In an argon filled glove box, [Rh(C_2H_4)(DavePhos)](OTf) (1.4 mg, 2 μmol), DavePhos (0.8 mg, 2 μmol), [Ru(bpy)$_3$](PF_6)$_2$ (0.9 mg, 1 μmol), LiOTf (7.8 mg, 0.05 mmol), polymerization inhibitor (10 mol%), and 1-(trifluoromethyl)-4-vinylbenzene **1q** (8.6 mg, 0.05 mmol) were placed in an oven dried glass tube (ϕ = 1.7 cm, 18 cm). To the mixture was added anhydrous *N*,*N*-dimethylacetamide (DMA, 1.0 mL). The tube was sealed with a three-way cock and removed from the glove box. A mix gas of CO_2/H_2 (2:1, 1 atm) was charged into the glass tube through the three-way cock. The mixture was then subjected to a blue LED (425 nm) irradiation (two sockets) with vigorous stirring. After 5 h, the reaction mixture was diluted with Et_2O and 1 N HCl aq. was added. The water phase was extracted with Et_2O three times. The combined organic layer was dried over $MgSO_4$. After removal of the solvent under reduced pressure, 1,1,2,2-tetrachloroethane was added to the crude product as an internal standard. The yields were calculated based on the integration of ^{1}H NMR spectra.

Supplementary Table 5. Examination of Polymerization Inhibitors Using **1q** as Substrate

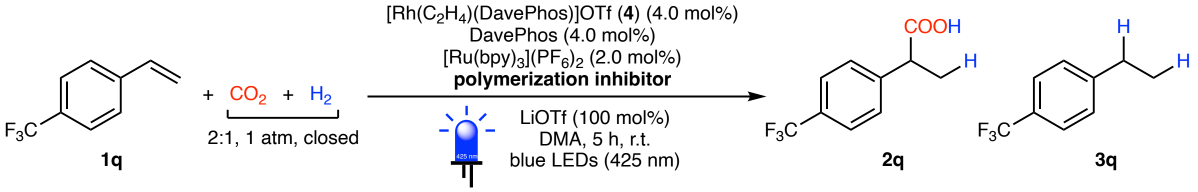

entry	polymerization inhibitor	yield of **2q** (%)[a]	yield of **3q** (%)[a]
1	none	14	n.d.
2	4-*tert*-butylcatechol (10 mol%)	n.d.	n.d.
3	TEMPO (10 mol%)	20	n.d.
4	4-OH-TEMPO (10 mol%)	11	n.d.
5	1,4-naphthoquinone (10 mol%)	15	n.d.
6	1,4-dihydroxynaphthalene (10 mol%)	19	n.d.
7	phenothiazine (10 mol%)	24	n.d.

5. Rhodium-Catalyzed Hydrocarboxylation of Olefins Using CO_2 and H_2

General Procedure: In an argon filled glove box, [Rh(C_2H_4)(DavePhos)](OTf) (1.4 mg, 2 μmol), DavePhos (0.8 mg, 2 μmol), [Ru(bpy)$_3$](PF_6)$_2$ (0.9 mg, 1 μmol), additive if any (0.05 mmol), and olefin substrate (**1**, 0.05 mmol) were placed in an oven dried glass tube (ϕ = 1.7 cm, 18 cm). To the mixture was added anhydrous *N*,*N*-dimethylacetamide (DMA, 1.0 mL). The tube was sealed with a three-way cock and removed from the glove box. CO_2/H_2 (2:1, 1 atm) was charged into the glass tube through the three-way cock. The mixture was then subjected to a blue LED (425 nm) irradiation (two sockets) with vigorous stirring. After 5 h, 1 N HCl aq. (1.0 mL) was added to the mixture, and the mixture was extracted with Et_2O three times. The crude mixture was analyzed by ^{1}H NMR to determine the yields of **2** and **3** using 1,1,2,2-tetrachloroethane as internal standard. Then the crude was diluted in Et_2O and extracted with 1 N NaOH aq. (2.0 mL). The basic water phase was then acidified with 1 N HCl aq. until pH ≈ 1, and was extracted again with Et_2O twice. The combined organic layer was dried over $MgSO_4$. After removal of the solvent under reduced pressure, the residue was purified with preparative thin layer chromatography (PTLC) on silica gel (elution: hexane/EtOAc + 1% AcOH) to give the desired carboxylic acid product.

2-(4-(Methoxycarbonyl)phenyl)propanoic acid (2a) [207455-46-7]:

Without additives: The reaction was conducted following the general procedure using methyl 4-vinylbenzoate **1a** (8.1 mg, 0.05 mmol). The crude mixture was purified directly by PTLC without reverse phase extraction to give 2-(4-(methoxycarbonyl)phenyl)propanoic acid **2a** (7.3 mg, 35.1 μmol, 70%) as white solid.

With LiOTf: The reaction was conducted following the general procedure using methyl 4-vinylbenzoate **1a** (8.1 mg, 0.05 mmol) and LiOTf as an additive (7.8 mg, 0.05 mmol). The crude mixture was purified by PTLC to give 2-(4-(methoxycarbonyl)phenyl)propanoic acid **2a** (8.4 mg, 40.3 μmol, 81%) as white solid.

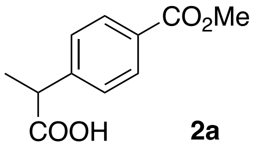 **2a**	The spectral data were in accordance with reference.[1]
	^{1}H NMR (500 MHz, $CDCl_3$) δ 1.54 (d, *J* = 7.2 Hz, 3H), 3.81 (q, *J* = 7.2 Hz, 1H), 3.91 (s, 3H), 7.39 (d, *J* = 8.2 Hz, 2H), 8.00 (d, *J* = 8.3 Hz, 2H), 11.02 (br, 1H).<br>^{13}C NMR (126 MHz, $CDCl_3$) δ 18.0, 45.3, 52.1, 127.7, 129.3, 130.0, 144.7, 166.8, 179.8.

2-[4-(Isopropoxycarbonyl)phenyl]propanoic acid (2b) [1924669-31-7]:

Without additives: The reaction was conducted following the general procedure using isopropyl 4-vinylbenzoate **1b** (9.6 mg, 0.05 mmol). The crude mixture was purified directly by PTLC without reverse phase extraction to give the title compound **2b** (6.0 mg, 25.4 μmol, 51%) as off-white solid.

With LiOTf: The reaction was conducted following the general procedure using isopropyl 4-vinylbenzoate **1b** (9.6 mg, 0.05 mmol) and LiOTf as additive (7.8 mg, 0.05 mmol). The crude mixture was purified directly by PTLC without reverse phase extraction to give the title compound **2b** (7.8 mg, 33.0 μmol, 66%) as off-white solid.

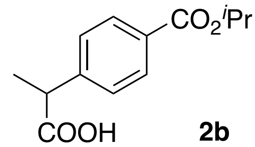 **2b**	The spectral data were in accordance with reference.[1]
	^{1}H NMR (600 MHz, $CDCl_3$) δ 1.35 (d, *J* = 6.0 Hz, 6H), 1.53 (d, *J* = 7.2 Hz, 3H), 3.81 (q, *J* = 7.2 Hz, 1H), 5.24 (sept, *J* = 6.0 Hz, 1H), 7.38 (d, *J* = 7.8 Hz, 2H), 8.00 (d, *J* = 7.8 Hz, 2H).

	^{13}C NMR (151 MHz, $CDCl_3$) δ 18.1, 21.9, 45.1, 68.4, 127.6, 130.0, 130.1, 144.6, 165.8, 178.2.

2-[4-(Benzyloxy)carbonyl]phenylpropanoic acid (2c) [1924669-35-1]:
Without additives: The reaction was conducted following the general procedure using benzyl 4-vinylbenzoate **1c** (11.9 mg, 0.05 mmol). The crude mixture was purified directly by PTLC without reverse phase extraction to give the title compound **2c** (5.1 mg, 17.9 µmol, 36%) as an off-white solid.
With LiOTf: The reaction was conducted following the general procedure using benzyl 4-vinylbenzoate **1c** (12.0 mg, 0.05 mmol) and LiOTf as an additive (7.8 mg, 0.05 mmol). The crude mixture was purified directly by PTLC without reverse phase extraction to give the title compound **2c** (8.1 mg, 28.5 µmol, 57%) as off-white solid.

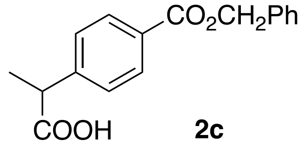 **2c**	The spectral data were in accordance with reference.[1]
	^{1}H NMR (600 MHz, $CDCl_3$) δ 1.53 (d, J = 7.2 Hz, 3H), 3.80 (q, J = 7.2 Hz, 1H), 5.36 (s, 2H), 7.32-7.36 (m, 1H), 7.36-7.41 (m, 4H), 7.43 (d, J = 7.2 Hz, 2H), 8.04 (d, J = 8.4 Hz, 2H).
	^{13}C NMR (151 MHz, $CDCl_3$) δ 18.1, 45.2, 66.7, 127.7, 128.1, 128.3, 128.6, 129.3, 130.2, 136.0, 145.0, 166.1, 178.8.

2-[4-(((3-Methylbut-2-en-1-yl)oxy)carbonyl)phenyl]propanoic acid (2d):
Without additives: The reaction was conducted following the general procedure using 3-methylbut-2-en-1-yl 4-vinylbenzoate **1d** (10.9 mg, 0.05 mmol). The crude mixture was purified directly by PTLC without reverse phase extraction to give the title compound **2d** (7.1 mg, 27.0 µmol, 54%) as off-white solid.
With LiOTf: The reaction was conducted following the general procedure using 3-methylbut-2-en-1-yl 4-vinylbenzoate **1d** (10.9 mg, 0.05 mmol) and LiOTf as an additive (7.8 mg, 0.05 mmol). The analysis of the crude mixture by ^{1}H NMR showed the presence of 21.5 µmol (43%) of the title compound **2d** (1,1,2,2-tetrachloroethane was used as an internal standard).

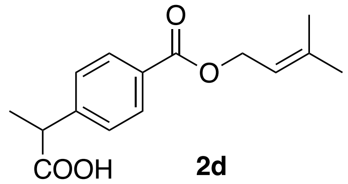 **2d**	**2d** New compound.
	^{1}H NMR (600 MHz, $CDCl_3$) δ 1.54 (d, J = 7.2 Hz, 3H), 1.76 (s, 3H), 1.78 (s, 3H), 3.80 (q, J = 7.2 Hz, 1H), 4.81 (d, J = 7.2 Hz, 2H), 5.44-5.47 (m, 1H), 7.38 (d, J = 8.4 Hz, 2H), 8.01 (d, J = 8.4 Hz, 2H). ^{13}C NMR (151 MHz, $CDCl_3$) δ 18.08, 18.12, 25.8, 45.2, 61.9, 118.6, 127.6, 129.7, 130.0, 139.2, 144.7, 166.4, 178.4. HRMS (ESI-TOF): m/z calcd for $C_{15}H_{18}O_4Na^+$ $[M+Na]^+$: 285.1097, found: 285.1125. FT-IR (ATR): 2973, 2930, 2873, 1709, 1608, 1448, 1416, 1375, 1267, 1180, 1100, 1019, 978, 930, 863, 768, 703 cm^{-1}.

2-(4-Carboxyphenyl)propionic acid (2e) [67381-50-4]:
Without additives: The reaction was conducted following the general procedure using 4-vinylbenzoic acid **1e** (7.4 mg, 0.05 mmol). The analysis of the crude mixture by ^{1}H NMR showed the presence of 11 µmol (22%) of the title compound 2-(4-carboxyphenyl)propionic acid **2e** (1,1,2,2-tetrachloroethane was used as an internal standard).
With LiOTf: The reaction was conducted following the general procedure using 4-vinylbenzoic acid **1e** (7.4 mg, 0.05 mmol) and LiOTf as an additive (7.8 mg, 0.05 mmol). The

crude mixture was purified directly by PTLC without reverse phase extraction to give 2-(4-carboxyphenyl)propionic acid **2e** (3.6 mg, 18.5 μmol, 37%) as white solid.

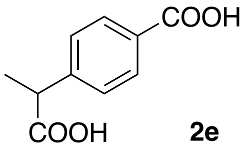 2e	The spectral data were in accordance with reference.[11]
	^{1}H NMR (500 MHz, acetone-d_6) δ 1.48 (d, J = 7.2 Hz, 3H), 3.88 (q, J = 7.0 Hz, 1H), 7.49 (d, J = 8.3 Hz, 2H), 8.01 (d, J = 8.2 Hz, 2H), 10.95 (br, 2H).<br>^{13}C NMR (126 MHz, acetone-d_6) δ 18.9, 45.8, 128.6, 130.2, 130.7, 147.4, 167.4, 175.0.

2-(4-Benzoylphenyl)propanoic acid (2f) [22410-97-5]:

<u>Without additives</u>: The reaction was conducted following the general procedure using phenyl(4-vinylphenyl)methanone **1f** (10.6 mg, 0.05 mmol). The crude mixture was purified with PTLC to give 2-(4-benzoylphenyl)propanoic acid **2f** (6.3 mg, 25.0 μmol, 49%) as white solid.

<u>With LiOTf</u>: The reaction was conducted following the general procedure using phenyl(4-vinylphenyl)methanone **1f** (10.4 mg, 0.05 mmol) and LiOTf as an additive (7.8 mg, 0.05 mmol). The crude mixture was purified with PTLC to give 2-(4-benzoylphenyl)propanoic acid **2f** (6.5 mg, 25.6 μmol, 51%) as a white solid.

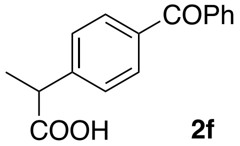 2f	The spectral data were in accordance with reference.[1]
	^{1}H NMR (500 MHz, $CDCl_3$) δ 1.58 (d, J = 7.2 Hz, 3H), 3.86 (q, J = 7.2 Hz, 1H), 7.45 (d, J = 8.2 Hz, 2H), 7.49 (t, J = 7.8 Hz, 2H), 7.60 (t, J = 7.5 Hz, 1H), 7.78-7.81 (m, 4H), 9.61 (br, 1H).<br>^{13}C NMR (151 MHz, $CDCl_3$) δ 18.1, 45.2, 127.6, 128.3, 130.0, 130.5, 132.5, 136.7, 137.5, 144.3, 178.6, 196.2.

2-[4-(4-Methoxybenzoyl)phenyl]propanoic acid (2g) [1159712-39-6]:

<u>Without additives:</u> The reaction was conducted following the general procedure using 4-methoxyphenyl(4-vinylphenyl)methanone **1g** (12.0 mg, 0.05 mmol). The crude mixture was purified by PTLC to give the title compound **2g** (5.7 mg, 20.0 μmol, 40%) as off-white solid.

<u>With LiOTf:</u> The reaction was conducted following the general procedure using 4-methoxyphenyl(4-vinylphenyl)methanone **1g** (11.9 mg, 0.05 mmol) and LiOTf as additive (7.9 mg, 0.05 mmol). The crude mixture was purified by PTLC to give the title compound **2g** (6.9 mg, 24.3 μmol, 49%) as white solid.

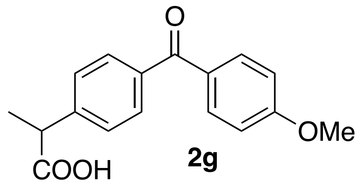 2g	**2g** New compound.
	^{1}H NMR (600 MHz, $CDCl_3$) δ 1.57 (d, J = 7.2 Hz, 3H), 3.84 (q, J = 7.2 Hz, 1H), 3.89 (s, 3H), 6.96 (d, J = 9.0 Hz, 2H), 7.43 (d, J = 8.4 Hz, 2H), 7.73 (d, J = 7.8 Hz, 2H), 7.82 (d, J = 8.4 Hz, 2H).<br>^{13}C NMR (151 MHz, $CDCl_3$) δ 18.1, 45.2, 55.5, 113.6, 127.5, 130.1, 130.2, 132.6, 137.4, 143.8, 163.3, 178.6, 195.1.<br>HRMS (ESI-TOF): m/z calcd for $C_{17}H_{16}O_3Na^+$ $[M+Na]^+$: 307.0941, found: 307.0938.<br>FT-IR (ATR): 2971, 2926, 2842, 1733, 1705, 1646, 1595, 1508, 1457, 1416, 1377, 1305, 1280, 1254, 1170, 1148, 1116, 1072, 1025, 925, 928, 840, 770, 687 cm^{-1}.

2-[4-(4-Fluorobenzoyl)phenyl]propanoic acid (2h):

Without additives: The reaction was conducted following the general procedure using 4-fluorophenyl(4-vinylphenyl)methanone **1h** (11.3 mg, 0.05 mmol). The crude mixture was purified by PTLC to give the title compound **2h** (4.4 mg, 16.2 μmol, 32%) as off-white solid.

With LiOTf: The reaction was conducted following the general procedure using 4-fluorophenyl(4-vinylphenyl)methanone **1h** (11.3 mg, 0.05 mmol) and LiOTf as an additive (7.8 mg, 0.05 mmol). The crude mixture was purified by PTLC to give the title compound **2h** (5.2 mg, 19.1 μmol, 38%) as white solid.

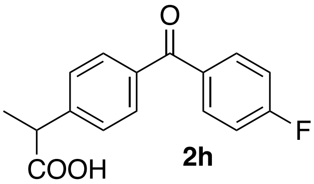 2h	**2h** New compound.
	^{1}H NMR (600 MHz, $CDCl_3$) δ 1.57 (d, J = 7.2 Hz, 3H), 3.85 (q, J = 7.2 Hz, 1H), 7.13-7.18 (m, 2H), 7.45 (d, J = 8.4 Hz, 2H), 7.75 (d, J = 8.4 Hz, 2H), 7.82-7.86 (m, 2H).<br>^{13}C NMR (151 MHz, $CDCl_3$) δ 18.1, 45.1, 115.5 (d, $J_{C\text{-}F}$ = 21.1 Hz), 127.7, 130.4, 132.6 (d, $J_{C\text{-}F}$ = 10.6 Hz), 133.7 (d, $J_{C\text{-}F}$ = 4.5 Hz), 136.6, 144.5, 165.4 (d, $J_{C\text{-}F}$ = 253.5 Hz), 177.9, 194.8.<br>^{19}F NMR (466 MHz, $CDCl_3$) δ −105.4.<br>HRMS (ESI-TOF): m/z calcd for $C_{16}H_{13}FO_3Na^+$ $[M+Na]^+$: 295.0741, found: 295.0753.<br>FT-IR (ATR): 2989, 2917, 2849, 1700, 1648, 1593, 1500, 1407, 1277, 1223, 1146, 1072, 1008, 930, 868, 852, 764, 744, 682 cm^{-1}.

2-(4-Acetylphenyl)propanoic acid (2i) [41387-29-5]:

Without additives: The reaction was conducted following the general procedure using 1-(4-vinylphenyl)ethan-1-one **1i** (7.3 mg, 0.05 mmol). The crude mixture was purified by PTLC to give the title compound **2i** (4.7 mg, 24.5 μmol, 49%) as off-white solid.

With LiOTf: The reaction was conducted following the general procedure using 1-(4-vinylphenyl)ethan-1-one **1i** (8.1 mg, 0.055 mmol) and LiOTf as additive (7.8 mg, 0.05 mmol). The crude mixture was purified with PTLC to give the title compound **2i** (4.7 mg, 24.5 μmol, 45%) as white solid.

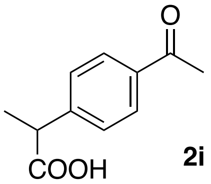 2i	The spectral data were in accordance with reference.[1]
	^{1}H NMR (600 MHz, $CDCl_3$) δ 1.55 (d, J = 7.2 Hz, 3H), 2.59 (s, 3H), 3.82 (q, J = 7.2 Hz, 1H), 7.42 (d, J = 7.2 Hz, 2H), 7.93 (d, J = 7.2 Hz, 2H).<br>^{13}C NMR (151 MHz, $CDCl_3$) δ 18.0, 26.6, 45.2, 127.9, 128.8, 136.3, 145.0, 178.5, 197.7.

2-(4-Isobutyrylphenyl)propanoic acid (2j) [65813-55-0]:

Without additives: The reaction was conducted following the general procedure using 2-methyl-1-(4-vinylphenyl)propan-1-one **1j** (8.7 mg, 0.05 mmol). The crude mixture was purified by PTLC to give the title compound **2j** (4.5 mg, 20.4 μmol, 41%) as off-white solid.

With LiOTf: The reaction was conducted following the general procedure using 2-methyl-1-(4-vinylphenyl)propan-1-one **1j** (8.7 mg, 0.05 mmol) and LiOTf as an additive (7.8 mg, 0.05 mmol). The crude mixture was purified by PTLC to give the title compound **2j** (5.4 mg, 24.5 μmol, 49%) as white solid.

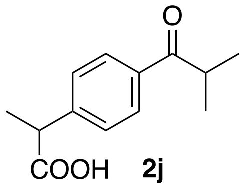 **2j**	The spectral data were in accordance with reference.[12]
	^{1}H NMR (600 MHz, $CDCl_3$) δ 1.20 (d, J = 6.6 Hz, 6H), 1.53 (d, J = 7.2 Hz, 3H), 3.53 (sept, J = 6.6 Hz, 1H), 3.80 (q, J = 6.6 Hz, 1H), 7.41 (d, J = 8.4 Hz, 2H), 7.93 (d, J = 8.4 Hz, 2H).<br>^{13}C NMR (151 MHz, $CDCl_3$) δ 18.0, 19.1, 35.3, 45.3, 127.9, 128.8, 135.3, 144.7, 179.6, 204.1.

2-(4-Cyanophenyl)propanoic acid (2k) [362052-00-4]:

<u>Without additives:</u> The reaction was conducted following the general procedure using 4-vinylbenzonitrile **1k** (9.2 mg, 0.07 mmol). The crude mixture was purified by PTLC to give the title compound **2k** (4.0 mg, 22.8 μmol, 32%) as off-white solid.

<u>With LiOTf:</u> The reaction was conducted following the general procedure using 4-vinylbenzonitrile **1k** (6.5 mg, 0.05 mmol) and LiOTf as an additive (7.8 mg, 0.05 mmol). The crude mixture was purified by PTLC to give the title compound **2k** (5.8 mg, 33.1 μmol, 66%) as off-white solid.

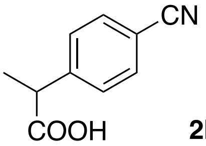 **2k**	The spectral data were in accordance with reference.[13]
	^{1}H NMR (500 MHz, $CDCl_3$) δ 1.54 (d, J = 7.2 Hz, 3H), 3.81 (q, J = 7.2 Hz, 1H), 7.44 (d, J = 8.2 Hz, 2H), 7.64 (d, J = 8.3 Hz, 2H), 10.70 (br, 1H).<br>^{13}C NMR (126 MHz, $CDCl_3$) δ 17.9, 45.3, 111.5, 118.5, 128.5, 132.5, 144.8, 179.2.

2-[6-(Methoxycarbonyl)naphthalen-2-yl]propanoic acid (2l)

<u>Without additives:</u> The reaction was conducted following the general procedure using methyl 6-vinyl-2-napthoate **1l** (10.8 mg, 0.05 mmol). The crude mixture was purified by PTLC to give the title compound **2l** (8.3 mg, 32.1 μmol, 63%) as off-white solid.

<u>With LiOTf:</u> The reaction was conducted following the general procedure using methyl 6-vinyl-2-napthoate **1l** (10.7 mg, 0.05 mmol) and LiOTf as an additive (7.8 mg, 0.05 mmol). The analysis of the crude by ^{1}H NMR showed the presence of 31.5 μmol (63%) of the title compound **2l** (1,1,2,2-tetrachloroethane was used as an internal standard).

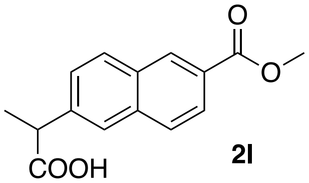 **2l**	**2l** New compound.
	^{1}H NMR (600 MHz, $CDCl_3$) δ 1.62 (d, J = 7.2 Hz, 3H), 3.94 (q, J = 7.2 Hz, 1H), 3.98 (s, 3H), 7.52 (dd, J = 8.4, 1.8 Hz, 1H), 7.80 (s, 1H), 7.84 (d, J = 8.4 Hz, 1H), 7.92 (d, J = 8.4 Hz, 1H), 8.05 (dd, J = 8.4, 1.8 Hz, 1H), 8.58 (d, J = 0.6 Hz, 1H).<br>^{13}C NMR (151 MHz, $CDCl_3$) δ 18.1, 45.4, 52.3, 125.7, 126.3, 126.6, 127.5, 128.1, 129.9, 130.8, 131.7, 135.6, 139.9, 167.2, 178.8.<br>HRMS (ESI-TOF): m/z calcd for $C_{15}H_{14}O_4Na^+$ $[M+Na]^+$: 281.0784, found: 281.0787.<br>FT-IR (ATR): 2976, 2932, 2850, 1713, 1633, 1435, 1288, 1237, 1198,1129, 1096, 990, 951, 913, 818, 770, 751 cm^{-1}.

2-(6-Benzoylnaphthalen-2-yl)propanoic acid (2m)

<u>Without additives:</u> The reaction was conducted following the general procedure using phenyl(6-vinylnaphthalen-2-yl)methanone **1m** (13.0 mg, 0.05 mmol). The crude mixture was purified by PTLC to give the title compound **2m** (8.0 mg, 26.3 μmol, 52%) as off-white solid.

With LiOTf: The reaction was conducted following the general procedure using phenyl(6-vinylnaphthalen-2-yl)methanone **1m** (13.0 mg, 0.05 mmol) and LiOTf as an additive (7.8 mg, 0.05 mmol). The crude mixture was purified by PTLC to give the title compound **2m** (8.2 mg, 26.9 μmol, 54%) as off-white solid.

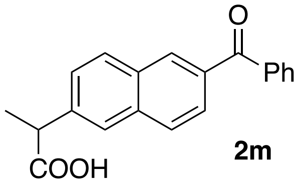	**2m** New compound.
	^{1}H NMR (600 MHz, $CDCl_3$) δ 1.64 (d, J = 7.2 Hz, 3H), 3.96 (q, J = 7.2 Hz, 1H), 7.49-7.55 (m, 3H), 7.60-7.64 (m, 1H), 7.82-7.85 (m, 3H), 7.88-7.92 (m, 2H), 7.94 (dd, J = 8.4, 1.8 Hz, 1H), 8.24 (s, 1H).<br>^{13}C NMR (151 MHz, $CDCl_3$) δ 18.1, 45.5, 126.25, 126.34, 126.8, 128.2, 128.4, 129.9, 130.1, 131.5, 131.6, 132.4, 134.9, 135.3, 137.9, 140.0, 179.2, 196.7.<br>HRMS (ESI-TOF): *m/z* calcd for $C_{20}H_{16}O_3Na^+$ $[M+Na]^+$: 327.0992, found: 327.1007.<br>FT-IR (ATR): 3055, 2973, 2919, 2850, 1733, 1702, 1648, 1625, 1595, 1577, 1474, 1446, 1284, 1239, 1176, 1120, 1072, 885, 820, 788, 714, 674 cm^{-1}.

2-(6-Acetylnaphthalen-2-yl)propanoic acid (2n)

Without additives: The reaction was conducted following the general procedure using 1-(6-vinylnapthalen-2-yl)ethan-1-one **1n** (9.8 mg, 0.05 mmol). The crude mixture was purified by PTLC to give the title compound **2n** (6.8 mg, 28.1 μmol, 56%) as off-white solid.

With LiOTf: The reaction was conducted following the general procedure using 1-(6-vinylnapthalen-2-yl)ethan-1-one **1n** (9.8 mg, 0.05 mmol) and LiOTf as an additive (7.8 mg, 0.05 mmol). The crude mixture was purified by PTLC to give the title compound **2n** (6.9 mg, 28.5 μmol, 57%) as off-white solid.

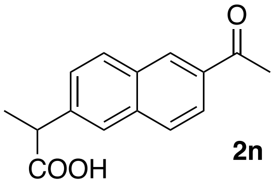	**2n** New compound.
	^{1}H NMR (600 MHz, $CDCl_3$) δ 1.63 (d, J = 7.2 Hz, 3H), 2.72 (s, 3H), 3.95 (q, J = 7.2 Hz, 1H), 7.54 (dd, J = 8.4, 1.8 Hz, 1H), 7.80 (s, 1H), 7.86 (d, J = 8.4 Hz, 1H), 7.94 (d, J = 8.4 Hz, 1H), 8.03 (dd, J = 8.4, 1.8 Hz, 1H), 8.44 (s, 1H).<br>^{13}C NMR (151 MHz, $CDCl_3$) δ 18.1, 26.7, 45.4, 124.4, 126.3, 126.7, 128.4, 129.9, 130.1, 131.8, 134.6, 135.6, 140.1, 178.8, 198.1.<br>HRMS (ESI-TOF): *m/z* calcd for $C_{15}H_{14}O_3Na^+$ $[M+Na]^+$: 265.0835, found: 265.0853.<br>FT-IR (ATR): 2954, 2916, 2850, 1675, 1625, 1465, 1407, 1359, 1262, 1221, 1128, 1064, 895, 829, 794, 727, 677 cm^{-1}.

2-(6-cyanonaphthalen-2-yl)propanoic acid (2o) [23981-78-4]

Without additives: The reaction was conducted following the general procedure using 6-vinyl-2-naphthonitrile **1o** (9.0 mg, 0.05 mmol). The crude mixture was purified by PTLC to give the title compound **2o** (6.5 mg, 28.9 μmol, 58%) as off-white solid.

With LiOTf: The reaction was conducted following the general procedure using 6-vinyl-2-naphthonitrile **1o** (9.0 mg, 0.05 mmol) and LiOTf as an additive (7.8 mg, 0.05 mmol). The crude mixture was purified by PTLC to give the title compound **2o** (5.3 mg, 23.5 μmol, 47%) as off-white solid.

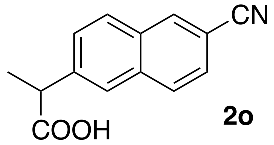 **2o**	**2o** New compound.
	^{1}H NMR (600 MHz, CDCl$_3$) δ 1.62 (d, J = 7.2 Hz, 3H), 3.95 (q, J = 7.2 Hz, 1H), 7.58 (dd, J = 8.4, 1.8 Hz, 1H), 7.61 (dd, J = 8.4, 1.8 Hz, 1H), 7.87 (s, 1H), 7.86-7.90 (m, 2H), 8.21 (s, 1H).<br>^{13}C NMR (151 MHz, CDCl$_3$) δ 18.1, 45.4, 109.5, 119.1, 126.5, 126.8, 127.6, 128.9, 129.1, 131.5, 133.8, 134.7, 140.7, 178.8.<br>HRMS (ESI-TOF): *m/z* calcd for $C_{14}H_{11}NO_2Na^+$ [M+Na]$^+$: 248.0688, found: 248.0697.<br>FT-IR (ATR): 3077, 3036, 2988, 2919, 2849, 2223, 1696, 1631, 1600, 1498, 1459, 1315, 1377, 1217, 1079, 891, 826, 673 cm^{-1}.

2-[3,5-Bis(trifluoromethyl)phenyl]propanoic acid (2p) [289686-73-3]

<u>**With LiOTf:**</u> The reaction was conducted following the general procedure using 1,3-bis(trifluoromethyl)-5-vinylbenzene **1p** (12.0 mg, 0.05 mmol) and LiOTf as an additive (7.8 mg, 0.05 mmol). 2,2,6,6-Tetramethylpiperidine-1-oxyl (TEMPO, 0.8 mg, 5.0 μmol, 10 mol%) was also added to the reaction mixture. The crude mixture was purified by PTLC to give 2-[3,5-bis(trifluoromethyl)phenyl]propanoic acid **2p** (4.8 mg, 16.8 μmol, 34%) as off-white solid.

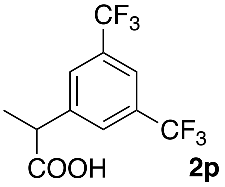 **2p**	The spectral data were in accordance with reference.[13]
	^{1}H NMR (500 MHz, CDCl$_3$) δ 1.60 (d, J = 7.2 Hz, 3H), 3.90 (q, J = 7.1 Hz, 1H), 7.79 (s, 2H), 7.81 (s, 1H).<br>^{13}C NMR (126 MHz, CDCl$_3$) δ 18.1, 44.9, 121.6–121.7 (m), 123.1 (q, J = 272.9 Hz), 128.0 (d, J = 2.7 Hz), 132.0 (q, J = 33.9 Hz), 141.9, 178.2.

2-[4-(Trifluoromethyl)phenyl]propanoic acid (2q) [134904-86-2]

<u>**With LiOTf:**</u> The reaction was conducted following the general procedure using 1-(trifluoromethyl)-4-vinylbenzene **1q** (8.6 mg, 0.05 mmol) and LiOTf (7.8 mg, 0.05 mmol) and phenothiazine (1.0 mg, 5.0 μmol, 10 mol%) as additives. The crude mixture was purified by PTLC to give 2-[4-(trifluoromethyl)phenyl]propanoic acid **2q** (2.1 mg, 9.6 μmol, 19%) as off-white solid.

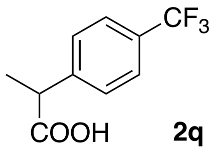 **2q**	The spectral data were in accordance with reference.[1]
	^{1}H NMR (500 MHz, CDCl$_3$) δ 1.54 (d, J = 7.2 Hz, 3H), 3.82 (q, J = 7.2 Hz, 1H), 7.44 (d, J = 8.1 Hz, 2H), 7.60 (d, J = 8.1 Hz, 2H), 10.95 (br, 1H).<br>^{13}C NMR (126 MHz, CDCl$_3$) δ 18.1, 45.1, 124.0 (q, J = 271.9 Hz), 125.65 (q, J = 3.7 Hz), 128.1, 129.8 (q, J = 32.5 Hz), 143.5, 179.2.

2-[4-(Methylsulfonyl)phenyl]propanoic acid (2r) [1250933-90-4]

<u>**With LiOTf:**</u> The reaction was conducted following the general procedure using 1-(methylsulfonyl)-4-vinylbenzene **1r** (9.1 mg, 0.05 mmol) and LiOTf (7.8 mg, 0.05 mmol) as an additive. The crude mixture was purified by PTLC to give 2-[4-(methylsulfonyl)phenyl]propanoic acid **2r** (3.1 mg, 13.6 μmol, 27%) as off-white solid.

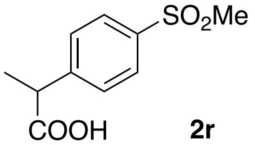 2r	**2r** New compound.
	^{1}H NMR (500 MHz, $CDCl_3$) δ 1.57 (d, J = 7.2 Hz, 3H), 3.05 (s, 3H), 3.87 (q, J = 7.1 Hz, 1H), 7.54 (d, J = 8.3 Hz, 2H), 7.92 (d, J = 8.3 Hz, 2H).<br>^{13}C NMR (126 MHz, $CDCl_3$) δ 18.2, 44.5, 44.9, 127.9, 128.7, 139.7, 145.9, 176.7.<br>HRMS (ESI-TOF): m/z calcd for $C_{10}H_{12}O_4SNa^+$: 251.0349, found: 251.0324.<br>FT-IR (ATR): 3055, 3001, 2917, 2846, 2619, 1702, 1595, 1560, 1489, 1457, 1407, 1292, 1258, 1226, 1183, 1142, 1090, 1066, 971, 906, 861, 808, 784, 739, 663 cm^{-1}.

2-[2-(Methoxycarbonyl)phenyl]propanoic acid (2s) [40570-20-5]

With LiOTf: The reaction was conducted following the general procedure using methyl 2-vinylbenzoate **1s** (8.1 mg, 0.05 mmol) and LiOTf (7.8 mg, 0.05 mmol) as an additive. The reaction was stirred under irradiation for 10 h. The crude mixture was purified by PTLC to give 2-[2-(methoxycarbonyl)phenyl]propanoic acid **2s** (2.4 mg, 11.5 μmol, 23%) as white solid.

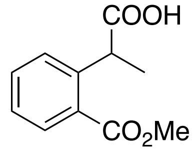 2s	The spectral data were in accordance with reference.[14]
	^{1}H NMR (500 MHz, $CDCl_3$) δ 1.57 (d, J = 7.0 Hz, 3H), 3.98 (s, 3H), 4.48 (q, J = 6.8 Hz, 1H), 7.34 (td, J = 7.5, 1.3 Hz, 1H), 7.52 (d, J = 7.7 Hz, 1H), 7.56 (td, J = 7.6, 1.2 Hz, 1H), 7.89 (dd, J = 8.0, 1.5 Hz, 1H), 10.46 (br, 1H).<br>^{13}C NMR (126 MHz, $CDCl_3$) δ 17.3, 41.5, 53.0, 127.3, 128.4, 129.2, 130.6, 133.0, 141.1, 169.8, 175.8.

4-Methoxybenzyl 2-carboxypropanoate (6) [1624597-29-0]

With LiOTf: The reaction was conducted following the general procedure using 4-methoxybenzyl acrylate **5** (9.6 mg, 0.05 mmol) and LiOTf (7.8 mg, 0.05 mmol) as an additive. The crude mixture was purified by PTLC to give the title compound **6** (2.4 mg, 10.1 μmol, 20%) as off-white solid.

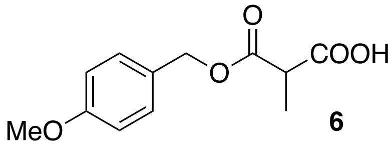 6	**6** New compound.
	^{1}H NMR (495 MHz, $CDCl_3$) δ 1.47 (d, J = 7.5 Hz, 3H), 3.50 (q, J = 7.0 Hz, 1H), 3.81 (s, 3H), 5.14 (s, 2H), 6.89 (d, J = 8.5 Hz, 2H), 7.29 (d, J = 9.0 Hz, 2H).<br>^{13}C NMR (125 MHz, $CDCl_3$) δ 13.7, 45.6, 55.3, 67.3, 114.0, 127.2, 130.1, 159.8, 170.2, 174.1.<br>HRMS (ESI-TOF): m/z calcd for $C_{12}H_{14}O_5Na^+$: 261.0733, found: 261.0747.<br>FT-IR (ATR): 2995, 2943, 2838, 1709, 1612, 1586, 1513, 1457, 1418, 1381, 1321, 1302, 1243, 1168, 1079, 1026, 949, 930, 850, 818, 756, 714 cm^{-1}.

Reactivity of Allyl 4-vinylbenzoate (1t) under standard conditions:

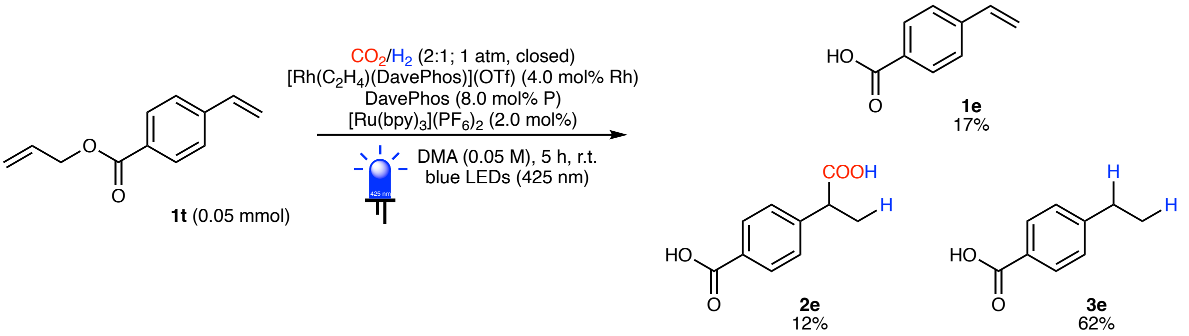

<u>Without additives:</u> The reaction was conducted following the general procedure using allyl 4-vinylbenzoate (**1t**, 9.7 mg, 0.05 mmol) for 8 h. The analysis of the crude by ^{1}H NMR showed the presence of 8.5 μmol (17%) of 4-vinylbenzoic acid (**1e**), 6 μmol (12%) of 4-(1-carboxyethyl)benzoic acid (**2e**), and 32 μmol (62%) of 4-ethylbenzoic acid (**3e**) (1,1,2,2-tetrachloroethane was used as an internal standard).

Supplementary Table 6. Summary of Substrate Scope and Unsuccessful Substrates

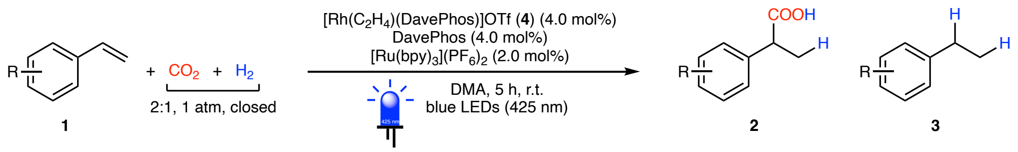

<table>
<tr><th>entry</th><th>1</th><th>LiOTf (100 mol%)</th><th>1 (%)</th><th>2 (%)</th><th>3 (%)</th><th>ratio (2 : 3)</th></tr>
<tr><td>1</td><td rowspan="2">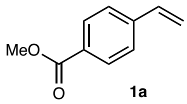 1a</td><td>w/</td><td>n.d.</td><td>84</td><td>16</td><td>5.3 : 1</td></tr>
<tr><td>2</td><td>w/o</td><td>trace</td><td>75</td><td>24</td><td>3.1 : 1</td></tr>
<tr><td>3</td><td rowspan="2">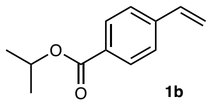 1b</td><td>w/</td><td>trace</td><td>71</td><td>22</td><td>3.2 : 1</td></tr>
<tr><td>4</td><td>w/o</td><td>7</td><td>52</td><td>23</td><td>2.3 : 1</td></tr>
<tr><td>5</td><td rowspan="2">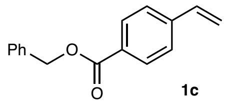 1c</td><td>w/</td><td>4</td><td>59</td><td>23</td><td>2.6 : 1</td></tr>
<tr><td>6</td><td>w/o</td><td>5</td><td>38</td><td>49</td><td>1 : 1.3</td></tr>
<tr><td>7</td><td>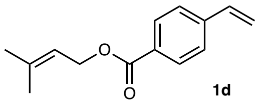 1d</td><td>w/o</td><td>6</td><td>55</td><td>28</td><td>2.0 : 1</td></tr>
<tr><td>8</td><td rowspan="2">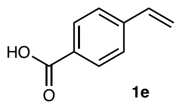 1e</td><td>w/</td><td>12</td><td>40</td><td>50</td><td>1 : 1.3</td></tr>
<tr><td>9</td><td>w/o</td><td>17</td><td>22</td><td>62</td><td>1 : 2.8</td></tr>
<tr><td>10</td><td rowspan="2">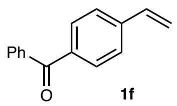 1f</td><td>w/</td><td>n.d.</td><td>54</td><td>46</td><td>1.2 : 1</td></tr>
<tr><td>11</td><td>w/o</td><td>3</td><td>52</td><td>48</td><td>1 : 1</td></tr>
<tr><td>12</td><td rowspan="2">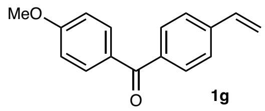 1g</td><td>w/</td><td>8</td><td>52</td><td>50</td><td>1 : 1</td></tr>
<tr><td>13</td><td>w/o</td><td>7</td><td>42</td><td>54</td><td>1 : 1.3</td></tr>
</table>

14	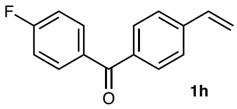 **1h**	w/	4	39	62	1 : 1.6
15		w/o	7	34	56	1 : 1.6
16	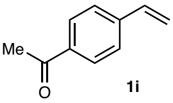 **1i**	w/o	2	48	10	4.8 : 1
17	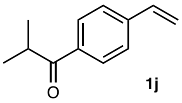 **1j**	w/	trace	50	23	2.2 : 1
18		w/o	trace	43	25	1.7 : 1
19	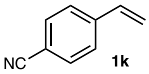 **1k**	w/	n.d.	78	22	3.5 : 1
20		w/o	20	39	37	1 : 1
21	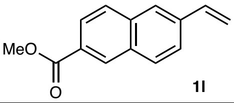 **1l**	w/o	trace	65	26	2.5 : 1
22	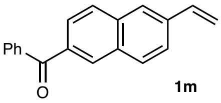 **1m**	w/o	6	55	28	2.0 : 1
23	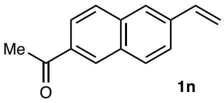 **1n**	w/o	trace	56	18	3.1 : 1
24	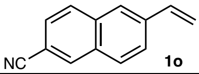 **1o**	w/o	trace	60	16	3.8 : 1
25	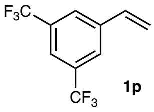 **1p**	w/	n.d.	40	n.d.	> 10 : 1
26	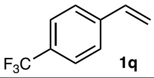 **1q**	w/	n.d.	24	n.d.	> 10 : 1
27	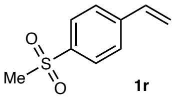 **1r**	w/	n.d.	29	39	1 : 1.3
28	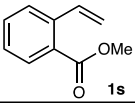 **1s**	w/	34	26	20	1.3 : 1
29	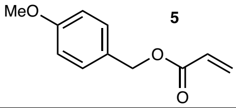 **5**	w/	n.d.	21	54	1 : 2.6
30	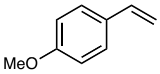	w/	58	n.d.	13	–
31	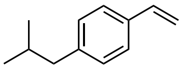	w/	4	3	16	–
32	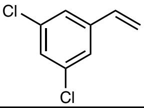	w/	44	16	16	–
33	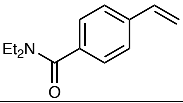	w/	39	6	38	–

34	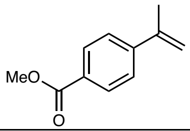	w/	92	n.d.	n.d.	–
35	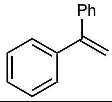	w/	n.d.	n.d.	n.d.	–

Synthesis of Ibuprofen from 2j

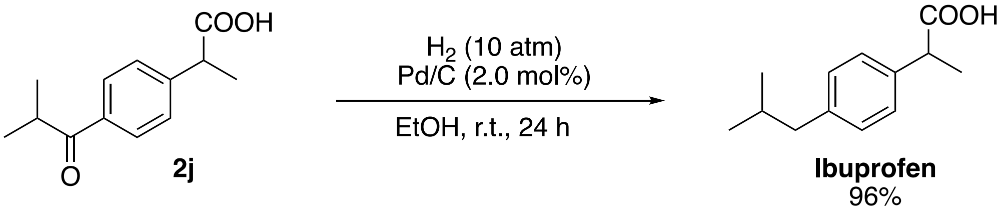

To an oven dried 10 mL test tube was added **2j** (10.8 mg, 49 μmol) and Pd/C [10 wt% Pd, 5 wt% H_2O] (1.1 mg, 2.0 mol%) in dry EtOH (1.0 mL) under N_2 atmosphere. The tube was placed into a nitrogen-purged stainless autoclave. After the autoclave was charged with 10 atm of H_2, the reaction mixture was stirred vigorously at room temperature for 24 h. The resulting mixture was diluted with Et_2O, and then carefully filtered through a Celite pad. The filtrate was concentrated, and dried under vacuum. The residue was purified by PTLC to give **Ibuprofen** (9.7 mg, 47 μmol, 96%) as an off-white solid.

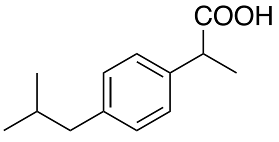 **Ibuprofen**	The spectral data were in accordance with reference.[11]
	^{1}H NMR (500 MHz, $CDCl_3$) δ 0.89 (d, J = 6.9 Hz, 6H), 1.50 (d, J = 6.9 Hz, 3H), 1.84 (sept, J = 6.9 Hz, 1H), 2.45 (d, J = 7.4 Hz, 2H), 3.72 (q, J = 7.3 Hz, 1H), 7.10 (d, J = 8.0 Hz, 2H), 7.21–7.23 (m, 2H). ^{13}C NMR (126 MHz, $CDCl_3$) δ 18.1, 22.4, 30.2, 44.9, 45.0, 127.3, 129.4, 137.0, 140.9, 180.4.

6. Experimental Mechanistic Studies

6.1 Thermal Background Reaction

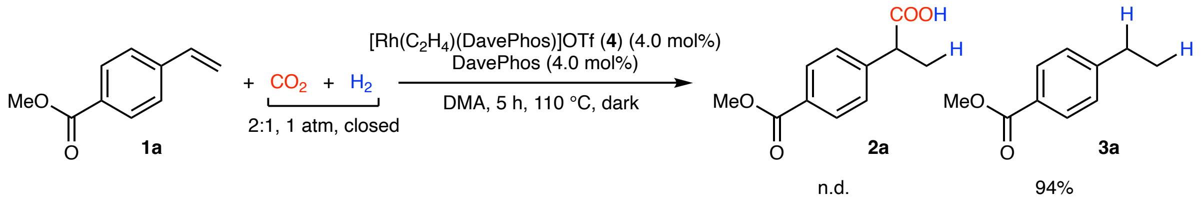

In an argon filled glove box, [Rh(C_2H_4)(DavePhos)](OTf) (1.4 mg, 2 μmol), DavePhos (0.8 mg, 2 μmol), and **1a** (8.1 mg, 0.05 mmol) were placed in an oven dried glass tube (ϕ = 1.7 cm, 18 cm). To the mixture was added anhydrous *N*,*N*-dimethylacetamide (DMA, 1.0 mL). The tube was sealed with a three-way cock and removed from the glove box. CO_2/H_2 (2:1, 1 atm) was charged into the glass tube through the three-way cock. The mixture was then heated at 110 ˚C with vigorous stirring. After 5 h, 1 N HCl aq. (1.0 mL) was added to the mixture, and the mixture was extracted with Et_2O three times. The crude mixture was analyzed by ^{1}H NMR using 1,1,2,2-tetrachloroethane as internal standard. **2a** was not observed and **3a** was obtained in 94% yield.

6.2 Benzylic C–H Bond Photocarboxylation

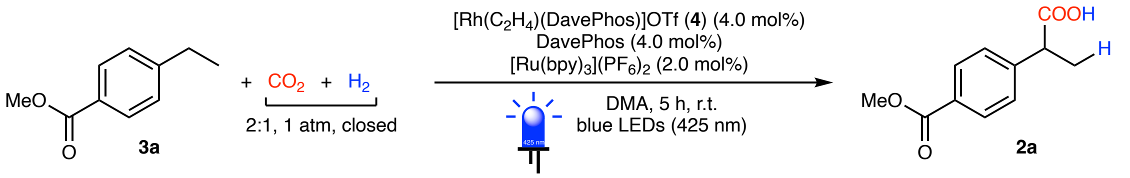

	2a	3a
w/ Rh complex **4**:	n.d.	> 99%
w/o Rh complex **4**:	n.d.	> 99%

In an argon filled glove box, [Rh(C_2H_4)(DavePhos)](OTf) (1.4 mg, 2 μmol) (if necessary), DavePhos (0.8 mg, 2 μmol), [Ru(bpy)$_3$](PF$_6$)$_2$ (0.9 mg, 1 μmol), and **3a** (8.2 mg, 0.05 mmol) were placed in an oven dried glass tube (ϕ = 1.7 cm, 18 cm). To the mixture was added anhydrous *N*,*N*-dimethylacetamide (DMA, 1.0 mL). The tube was sealed with a three-way cock and removed from the glove box. CO_2/H_2 (2:1, 1 atm) was charged into the glass tube through the three-way cock. The mixture was then subjected to a blue LED (425 nm) irradiation (two sockets) with vigorous stirring. After 5 h, 1 N HCl aq. (1.0 mL) was added to the mixture, and the mixture was extracted with Et_2O three times. The crude mixture was analyzed by ^{1}H NMR using 1,1,2,2-tetrachloroethane as internal standard. In both reactions (w/ and w/o Rh complex **4**), **2a** was not observed and **3a** was quantitatively recovered.

6.3 Synthesis of Rh Complexes 7–9 and Phosphonium Salt 10

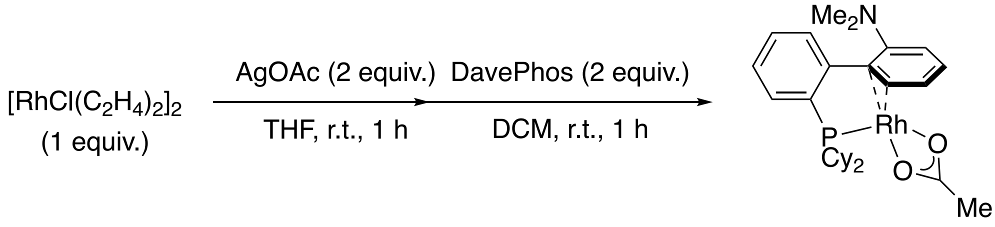

[Rh(OAc)(DavePhos)] (**7**)
59%

In an argon filled glove box, a THF solution (5.0 mL) of $[RhCl(C_2H_4)_2]_2$ (117 mg, 0.30 mmol) was stirred in a 50 mL flask at room temperature. AgOAc (102 mg, 0.61 mmol) was added to the solution in small portions. White precipitates appeared immediately, and the mixture was stirred for 1 h. After all the volatiles were removed in vacuo, DCM (5.0 mL) was added to the residue and the mixture was filtered through Celite. The organic filtrate was evaporated again under reduced pressure to remove all the volatiles. DavePhos (240 mg, 0.61 mmol) was added to the residue and the flask was charged with DCM (2.0 mL). The solution was stirred at room temperature for 1 h to ensure complete complexation. The resulting mixture was filtered through Celite again, and the filtrate was evaporated under reduced pressure to yield [Rh(OAc)(DavePhos)] (7) (198 mg, 0.36 mmol, 59 %) as red solid.

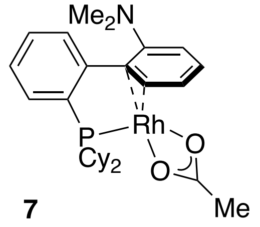 **7**	**7** New compound.
	^{1}H NMR (495 MHz, C_6D_6): δ 0.90-1.21 (m, 5H), 1.24-1.32 (m, 1H), 1.53-1.59 (m, 4H), 1.67 (s, 3H), 1.68-1.93 (m, 9H), 2.08-2.16 (m, 1H), 2.23 (d, J = 11.3 Hz, 1H), 2.60 (d, J = 9.4 Hz, 1H), 2.88 (s, 6H), 4.65 (d, J = 5.4 Hz, 1H), 6.23 (d, J = 7.4 Hz, 1H), 6.87 (t, J = 7.6 Hz, 1H), 6.94 (t, J = 7.6 Hz, 1H), 6.98-7.08 (m, 2H), 7.13 (t, J = 7.4 Hz, 1H), 7.53 (d, J = 7.4 Hz, 1H).<br>^{13}C NMR (125 MHz, C_6D_6): δ 24.1, 26.4 (d, J = 12.0 Hz), 27.3 (d, J = 12.0 Hz), 27.5 (d, J = 8.4 Hz), 27.6 (d, J = 11.9 Hz), 29.3 (d, J = 18.0 Hz), 29.9, 30.6, 36.4 (d, J = 22.3 Hz), 36.5 (d, J = 20.4 Hz), 44.3, 72.5 (d, J = 10.8 Hz), 80.0 (d, J = 12.7 Hz), 107.9, 122.0, 125.8 (d, J = 4.9 Hz), 129.2, 129.6, 129.7, 130.5, 132.4 (d, J = 38.3 Hz), 154.6, 157.1 (d, J = 20.4 Hz), 190.4.<br>$^{31}P\{^{1}H\}$ NMR (200 MHz, C_6D_6): δ 79.8 (d, J = 185.4 Hz).<br>HRMS (ESI): m/z calcd for $C_{28}H_{38}NO_2PRh^+$ $[M–H]^+$: 554.1690, found 554.1669.<br>IR (ATR): 2925, 2848, 2786, 1583, 1552, 1434, 1412, 1132 cm^{-1}.

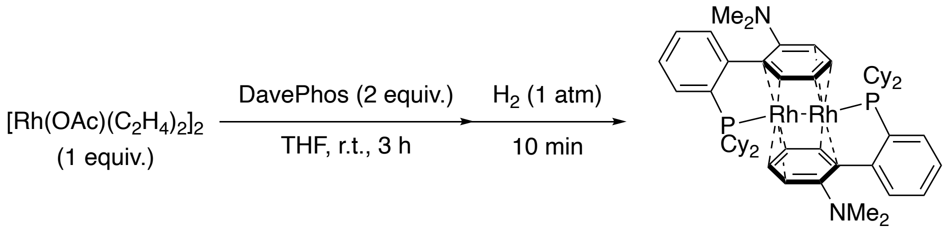

[Rh(DavePhos)]$_2$ (**8**)
31%

[Rh(OAc)(C_2H_4)$_2$]$_2$ (21.8 mg, 0.050 mmol) and DavePhos (39.4 mg, 0.10 mmol) were dissolved in THF (1 mL) in a 50 mL round bottom flask, and the mixture was stirred at room temperature for 3 h. The solvent was removed in vacuo. The residue was dissolved in DMA, and H_2 gas (1 atm) was charged into the system. After 10 min, brown solid was precipitated. The solid was collected by filtration and washed with THF and Et_2O to yield [Rh(DavePhos)]$_2$ **8** (15.4 mg, 16 µmol, 31%) as brown solid. **8** was observed as mixtures of two isomers (~5:4) in C_6D_6 by ^{1}H NMR spectroscopy. Block shaped brown crystals were grown by vapor diffusion of DMF into a benzene solution of **8** under an Ar atmosphere, which showed the structure of **8** by single crystal X-ray analysis.

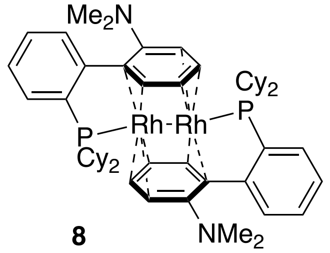 **8**	**8** New compound.
	^{1}H NMR (500 MHz, C_6D_6): δ 0.80-2.20 (m, 41H), 2.36-2.46 (m, 3H), 2.48 (s, 5.16H), 2.61 (s, 6.84H), 4.00 (t, J = 6.4 Hz, 0.86H), 4.50 (d, J = 5.4 Hz, 1.14H), 4.54 (d, J = 5.9 Hz, 1.14H), 4.73 (s, 0.86H), 4.99 (t, J = 5.9 Hz, 1.14H), 5.47 (d, J = 5.0 Hz, 0.86H), 6.46-6.52 (m, J = 5.9 Hz, 1.14H), 6.74 (d, J = 5.0 Hz, 1.14H), 7.08-7.20 (m, 3.72H), 7.31-7.38 (m, 1.14H), 7.42-7.46 (m, 0.86H), 7.58 (d, J = 6.9 Hz, 1.14H), 7.96 (d, J = 7.9 Hz, 0.86H) .<br>^{13}C NMR (151 MHz, C_6D_6): δ 21.3, 26.4-28.1 (m), 29.4, 29.7, 29.9, 30.9, 34.6, 36.9, 37.9, 40.1, 42.6, 43.9, 64.5 (d, J = 4.5 Hz), 66.1 (d, J = 10.6 Hz), 69.4, 69.8, 72.6, 72.7, 77.2, 81.7, 83.3, 125.6, 131.4, 134.5 (d, J = 22.7 Hz), 160.9, 161.0, 161.1, 164.4.<br>^{31}P{^{1}H} NMR (202 MHz, C_6D_6): δ 42.2-44.7 (m).<br>HRMS(ESI): m/z calcd for $C_{52}H_{71}N_2OP_2Rh_2^+$ [M+O–H]$^+$: 1007.3147, found 1007.3152. (The complex might be oxidized during measurement.)<br>IR (ATR): 3047, 2923, 2846, 1647, 1581, 1540, 1481, 1445, 1413, 1397,1336, 1319, 1234, 1206, 1192, 1067 cm^{-1}.

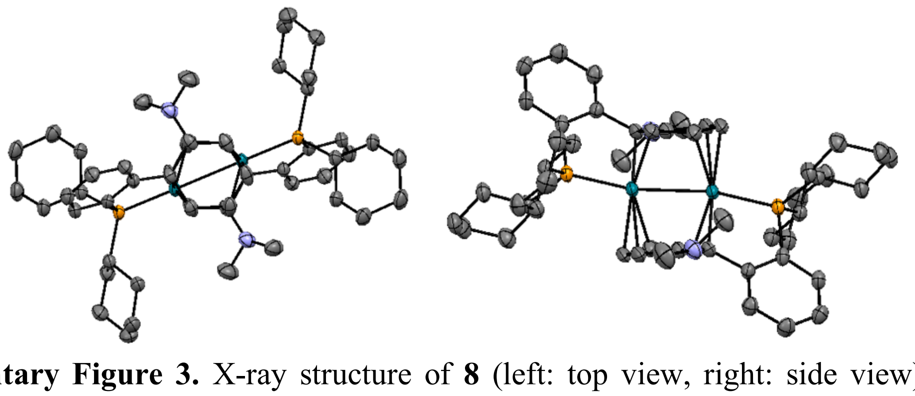

Supplementary Figure 3. X-ray structure of **8** (left: top view, right: side view). Thermal ellipsoids are set at 50% probability. H atoms are omitted for clarity.

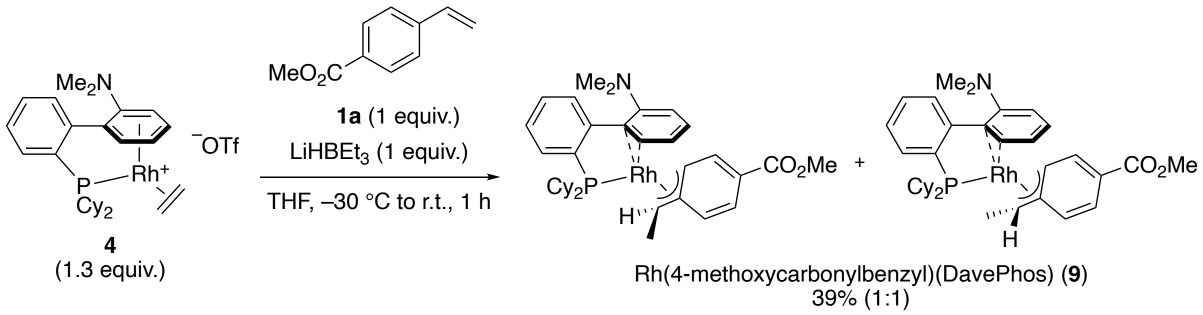

To a solution of **4** (88.8 mg, 0.13 mmol) and **1a** (16.2 mg, 0.10 mmol) in THF (10 mL), $LiHBEt_3$ (1.48 M in THF, 66 μL, 0.098 mmol) was slowly added at –30 °C and gradually warmed to room temperature over 1 h. The solution was evaporated under reduced pressure. The residue was dissolved in hexane and filtered through Celite. Evaporation of the solvent and trituration by cold hexane (–30 °C) gave **9** (25.2 mg, 0.038 mmol, 39%) as red solid.

9 was obtained as a 1:1 mixture of two isomers judging from ^{1}H and ^{31}P NMR. The large P–Rh coupling constants (J = 256.1 and 261.6 Hz) indicate that the P nucleus is located *trans* to the Ar ring and *cis* to the benzyl carbon for the both isomers.[15] The structures were additionally verified by ^{1}H-^{1}H COSY and NOESY NMR techniques (see Section 10 for the corresponding NMR spectra). Key NOESY correlations are shown below by red arrows.

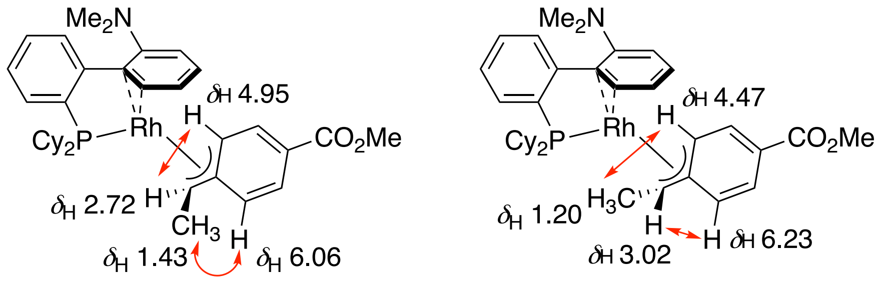

^{1}H NMR (500 MHz, THF-d_8, –78 °C): δ 0.40-1.70 (m, 16H), 1.78-3.09 (m, 16H), 3.81-3.90 (m, 3H), 4.47 (br, 0.5H), 4.95 (br, 0.5H), 5.28 (br, 0.5H), 5.88 (br, 0.5H), 6.06 (br, 0.5H), 6.16-6.34 (m, 1.5H), 6.49 (br, 0.5H), 6.58-6.74 (m, 1H), 6.88 (br, 0.5H), 7.03-7.20 (m, 3.5H), 7.20-7.35 (m, 1.5H), 7.56-7.67 (m, 1H).
^{13}C NMR (126 MHz, THF-d_8, –78 °C): δ 17.0, 18.0, 27.0-30.8 (m), 35.3, 35.5, 35.7, 35.9, 37.5 (d, J = 22.1 Hz), 38.5 (d, J = 24.4 Hz), 41.3, 43.1, 51.6, 84.1 (d, J = 11.5 Hz), 90.3, 91.8, 95.6, 97.5, 106.5, 106.7, 115.2, 118.1, 120.6, 121.3, 122.3, 123.5, 124.8, 126.2, 126.7, 127.6, 129.4, 129.6, 132.1, 132.4, 133.3, 133.5, 145.0, 154.2, 155.0, 155.5 (d, J = 21.5 Hz), 156.3 (d, J = 21.5 Hz), 167.5.
$^{31}P\{^{1}H\}$ NMR (202 MHz, THF-d_8, –78 °C): δ 57.4 (d, J = 256.1 Hz, 0.5P), 54.0 (d, J = 261.6 Hz, 0.5P).
HRMS (ESI): m/z calcd for $C_{36}H_{46}NO_2PRh^+$ $[M–H]^+$: 658.2316, found 658.2262.
IR (ATR): 3078, 2933, 2849, 1447, 1436, 1263, 1223, 1143, 1029 cm^{-1}.

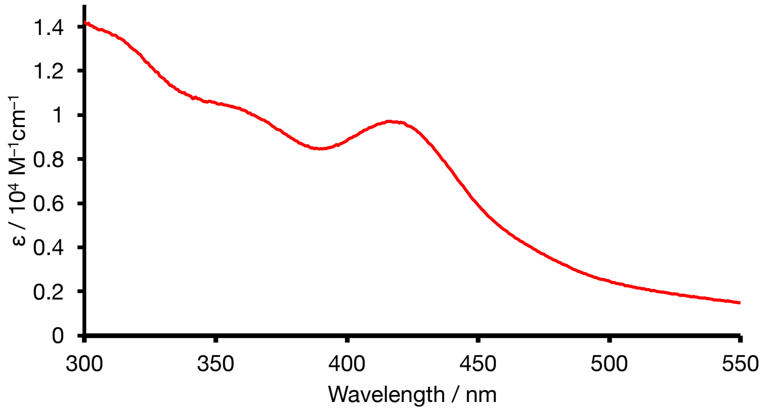

Supplementary Figure 4. Electronic absorption spectrum of **9** in DMA.

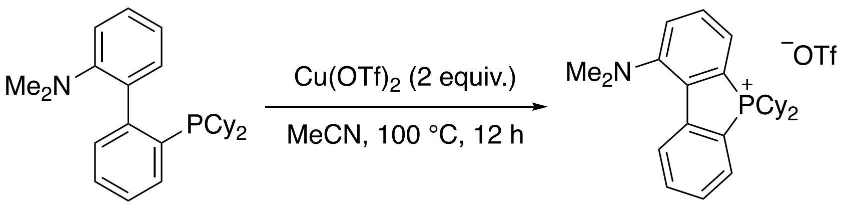

(1 eq.)

10

The phosphonium salt **10** was prepared according to the literature procedure using DavePhos and $Cu(OTf)_2$.[16]

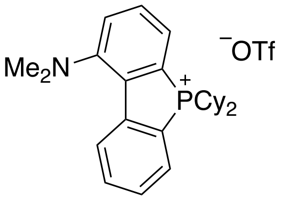<br>**10**	**10** New compound.
	^{1}H NMR (495 MHz, CD_3CN): δ 1.08-1.30 (m, 6H), 1.31-1.42 (m, 4H), 1.64-1.85 (m, 10H), 2.79 (s, 6H), 2.96-3.06 (m, 2H), 7.58-7.68 (m, 4H), 7.88 (td, J = 7.9, 1.0 Hz, 1H), 7.96 (t, J = 7.9 Hz, 1H), 8.68 (dd, J = 7.9, 3.0 Hz, 1H).<br>^{13}C NMR (125 MHz, CD_3CN): δ 25.7, 25.96, 25.99, 26.01, 26.04, 26.4 (d, J = 14.4 Hz), 30.7 (d, J = 40.8 Hz), 44.6, 117.5, 120.0, 120.7, 122.1 (q, J = 330.4 Hz), 126.8 (d, J = 9.5 Hz), 127.7, 128.4 (d, J = 9.6 Hz), 130.0 (d, J = 12.0 Hz), 132.0 (d, J = 13.1 Hz), 132.9 (d, J = 9.6 Hz), 136.8, 137.8 (d, J = 15.5 Hz), 146.5 (d, J = 14.4 Hz), 154.4 (d, J = 10.8 Hz).<br>^{31}P{^{1}H} NMR (200 MHz, CD_3CN): δ 40.0.<br>^{19}F NMR (466 MHz, CD_3CN): δ –79.4.<br>HRMS (ESI): m/z calcd for $C_{26}H_{35}NP^+$ $[M–OTf]^+$: 392.2502, found 392.2488.<br>IR (ATR): 3060, 2928, 2855, 2791, 1579, 1447, 1258, 1145, 1029 cm^{-1}.

6.4 Reactivity of Rh complex 4 and DavePhos under H_2 Atmosphere

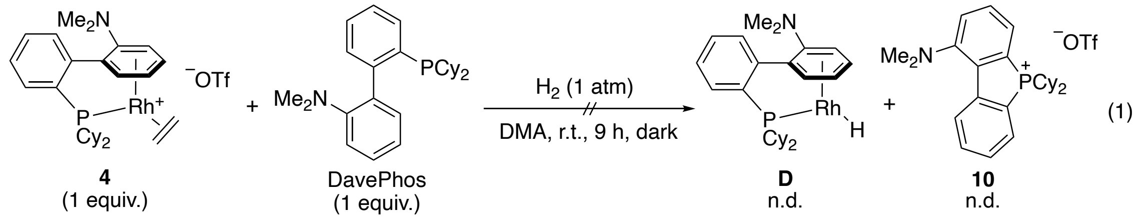

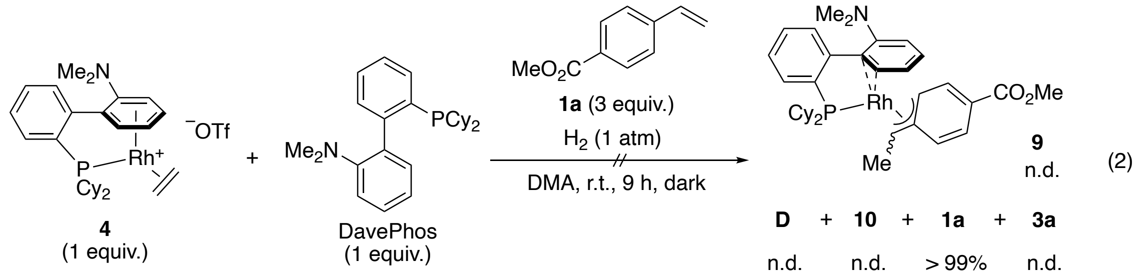

4 (6.7 mg, 10 μmol), DavePhos (3.9 mg, 10 μmol), **1a** (4.9 mg, 30 μmol) (if necessary), and DMA (0.75 mL) were added to a J. Young NMR tube under argon. H_2 gas was charged into the NMR tube. The mixture was left standing under dark for 9 h. The composition of the mixture was determined by ^{1}H NMR (no-D) and ^{31}P NMR.

In eq. 1, after 9 h, **D** and **10** were not observed at all. Rh complex **4** and DavePhos were detected as the major products, while very small amounts (approximately < 10%) of unidentified compounds (not complex **8**) were observed. We assumed that the ethylene ligand in **4** was gradually saturated by H_2, causing slow decomposition of **4**.
In eq. 2, after 9 h, **D** and **10** were not observed at all. Rh complex **4**, DavePhos, and **1a** remained completely unchanged. The addition of styrene **1a** may stabilize the cationic complex **4** from undesired decomposition.
These results indicated that both **4** and DavePhos were quite stable under a H_2 atmosphere under dark.

6.5 Generation of Phosphonium Salt 10 from DavePhos•HOTf Salt

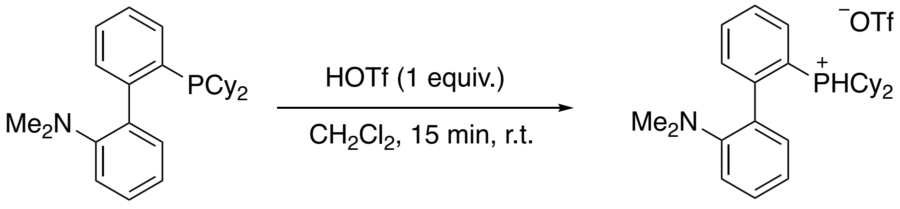

DavePhos

DavePhos•HOTf
99%

To an oven dried 30 mL Schlenk flask was added DavePhos (98.4 mg, 0.25 mmol) in dry CH_2Cl_2 (5 mL) under a N_2 atmosphere. Trifluoromethanesulfonic acid (22 μL, 0.25 mmol) was added to the solution. After stirring at room temperature for 15 min, all volatiles were removed under vacuum to give a white solid. Dry toluene (5 mL) was added and the white suspension was stirred for 30 min. The white solid was collected by filtration, washed with hexane, and dried under vacuum to give DavePhos•HOTf salt (134.5 mg, 0.25 mmol, 99%) as white solid.

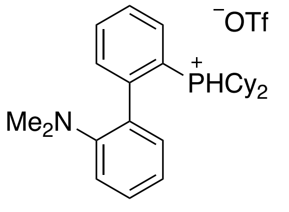 DavePhos•HOTf	**DavePhos•HOTf** New compound.
	^{1}H NMR (500 MHz, $CDCl_3$): δ 0.71-0.81 (m, 1H), 0.84-1.00 (m, 2H), 1.22-1.37 (m, 4H), 1.43-1.46 (m, 1H), 1.51-1.63 (m, 5H), 1.68-1.76 (m, 2H), 1.82-1.85 (m, 2H), 1.92-1.94 (m, 2H), 2.13-2.17 (m, 1H), 2.50 (s, 6H), 2.57-2.65 (m, 1H), 3.13-3.22 (m, 1H), 5.96 (ddd, J = 483.4, 9.8, 3.9 Hz, 1H), 7.10 (dd, J = 7.4, 1.7 Hz, 1H), 7.14-7.18 (m, 2H), 7.45-7.49 (m, 1H), 7.53 (dd, J = 7.4, 3.7 Hz, 1H), 7.69-7.72 (m, 1H), 7.84 (t, J = 7.4 Hz, 1H), 8.00 (dd, J = 11.7, 7.7 Hz, 1H).<br>^{13}C NMR (126 MHz, $CDCl_3$): δ 24.9, 25.37, 25.43 (d, J = 13.2 Hz), 25.6 (d, J = 14.4 Hz), 25.82, 25.84 (d, J = 10.8 Hz), 26.0 (d, J = 8.4 Hz), 27.0 (d, J = 3.6 Hz), 27.9 (d, J = 42.0 Hz), 28.2, 28.3, 30.3 (d, J = 42.0 Hz), 43.5, 112.4 (d, J = 79.2 Hz), 118.7, 120.8 (q, J = 320.3 Hz), 123.2, 128.7 (d, J = 12.0 Hz), 130.6 (d, J = 3.6 Hz), 130.9, 132.3 (d, J = 9.6 Hz), 132.8, 133.7 (d, J = 10.8 Hz), 135.3 (d, J = 2.4 Hz), 147.3 (d, J = 6.0 Hz), 149.4.<br>^{31}P{^{1}H} NMR (200 MHz, $CDCl_3$): δ 21.3.<br>^{19}F NMR (466 MHz, $CDCl_3$): δ –78.1.<br>HRMS (ESI): m/z calcd for $C_{26}H_{37}NP^+$ $[M–OTf]^+$: 394.2658, found 394.2662.

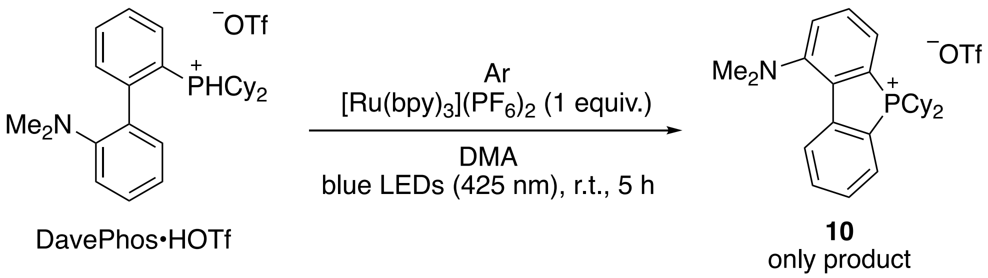

In an argon filled glovebox, DavePhos•HOTf (2.2 mg, 4 μmol) and $[Ru(bpy)_3](PF_6)_2$ (3.4 mg, 4 μmol) were placed in an oven dried test tube (ϕ = 1.7 cm, 18 cm). To the mixture was added anhydrous *N*,*N*-dimethylacetamide (DMA, 1.0 mL). The tube was sealed with a three-way cock and removed from the glovebox. The mixture was then subjected to a blue LED (425 nm) irradiation (two sockets) with vigorous stirring. After 5 h, the composition of the reaction mixture was confirmed by ^{31}P NMR analysis. The results showed full consumption of DavePhos•HOTf and **10** was observed as the only product.

6.6 Hydrogenolysis of [Rh(OAc)(DavePhos)] (7) (Fig. 3b)

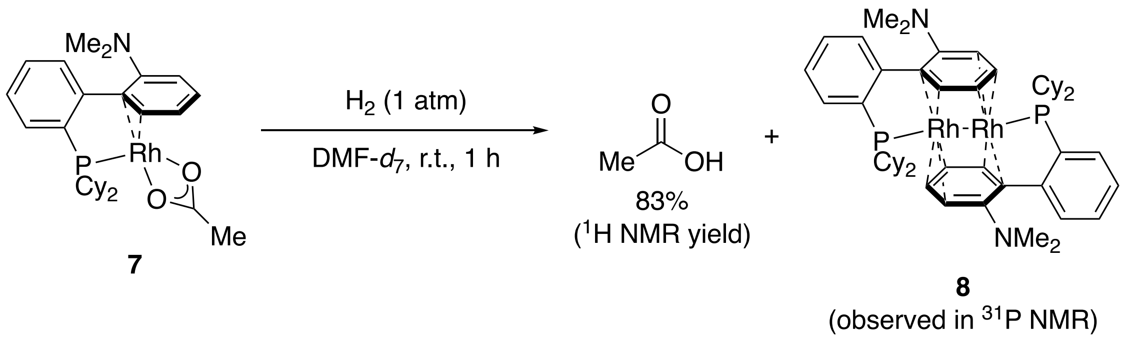

7 (16.6 mg, 30 μmol), 1,3,5-trimethoxybenzene (1.7 mg, 10 μmol), and DMF-d_7 (0.75 mL) were added in a J. Young NMR tube. H_2 gas was charged into the NMR tube by a freeze-pump-thaw method. After the mixture was left standing for 1 h, H_2 gas was exchanged to N_2 gas by a freeze-pump-thaw method. The yield of acetic acid (83%) was determined by ^{1}H NMR using 1,3,5-trimethoxybenzene as an internal standard. The generation of **8** was confirmed by ^{31}P NMR.

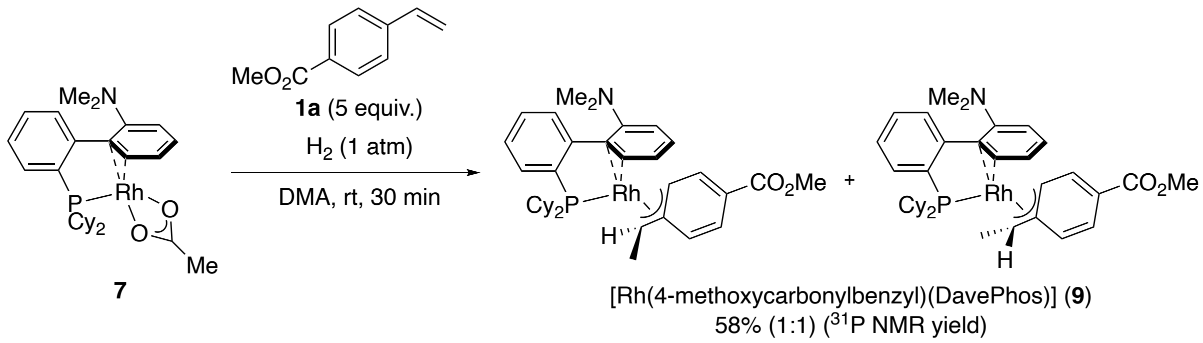

7 (5.5 mg, 10 μmol), **1a** (8.1 mg, 50 μmol), DMF-d_6 (0.75 mL), and a sealed capillary containing a toluene solution of $P(OPh)_3$ were added in a J. Young NMR tube. H_2 gas was charged into the NMR tube. After the mixture was left standing for 30 min, H_2 gas was exchanged to N_2 gas by a freeze-pump-thaw method. The yield of **9** (58%) was determined by ^{31}P NMR using $P(OPh)_3$ as an external standard.

6.7 Reactivity of Benzylrhodium Complex 9 (Fig. 3c)

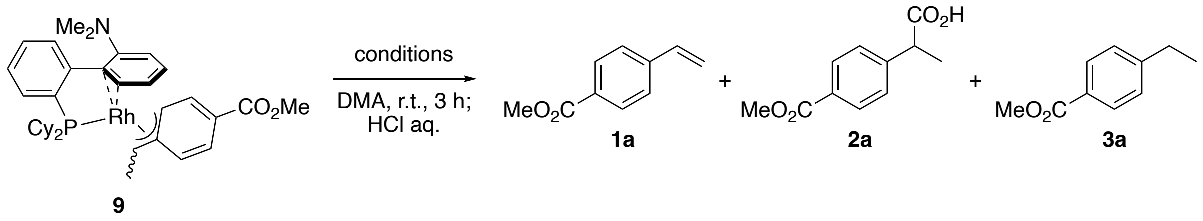

entry	conditions	^{1}H NMR yield (%) 1a	2a	3a
1	CO_2 (1 atm), dark	80	n.d.	7
2	CO_2 (1 atm), [Ru(bpy)$_3$](PF$_6$)$_2$ (30 mol%), blue LEDs (425 nm)	7	33	35
3	H_2 (1 atm), dark	trace	n.d.	95
4	CO_2 / H_2 (2:1, 1 atm), [Ru(bpy)$_3$](PF$_6$)$_2$ (30 mol%), blue LEDs (425 nm)	n.d.	23	55

General procedure: **9** (6.5 mg, 10 µmol), [Ru(bpy)$_3$](PF$_6$)$_2$ (2.6 mg, 3 µmol),) and DMA (0.6 mL) were added in a J. Young NMR tube. CO_2, H_2, or CO_2/H_2 (2:1) gas was charged into the NMR tube by a freeze-pump-thaw method. The mixture was irradiated with visible light (425 nm) or left in the dark at room temperature for 3 h. The mixture was diluted with Et_2O (5.0 mL), and 1 N HCl aq. (1.0 mL) was added to the mixture. The organic layer was extracted with Et_2O three times, dried over $MgSO_4$, and evaporated in vacuo to give the crude product. The yields of **1a**, **2a**, and **3a** were determined by ^{1}H NMR in $CDCl_3$ using CH_2Br_2 (3.5 µL) as an internal standard.

6.8 Time Course Analysis of Hydrocarboxylation and Hydrogenation of 1a

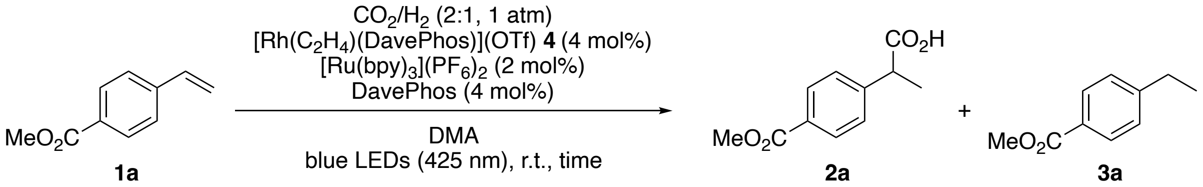

In an argon filled glovebox, [Rh(C_2H_4)(DavePhos)](OTf) **4** (1.4 mg, 2 µmol), DavePhos (0.8 mg, 2 µmol), [Ru(bpy)$_3$](PF$_6$)$_2$ (0.9 mg, 1 µmol), and **1a** (8.1 mg, 0.05 mmol) were placed in an oven dried test tube (ϕ = 1.7 cm, 18 cm). To the mixture was added anhydrous *N*,*N*-dimethylacetamide (DMA, 1.0 mL). The tube was sealed with a three-way cock and removed from the glovebox. A mix gas of CO_2/H_2 (2:1, 1 atm) was charged into the glass tube through the three-way cock. The mixture was then subjected to a blue LED (425 nm) irradiation (two sockets) with vigorous stirring. After X h, the reaction mixture was diluted with Et_2O (5.0 mL) and 1 N HCl aq. (1.0 mL) was added to the mixture. The organic layer was extracted with Et_2O three times. The organic layer was dried over $MgSO_4$. After removal of the solvent under reduced pressure, 1,1,2,2-tetrachloroethane (10 µL) was added to the crude product as an internal standard. The conversion of **1a** and the yields of **2a** and **3a** were calculated based on the integration of ^{1}H NMR spectra in $CDCl_3$.

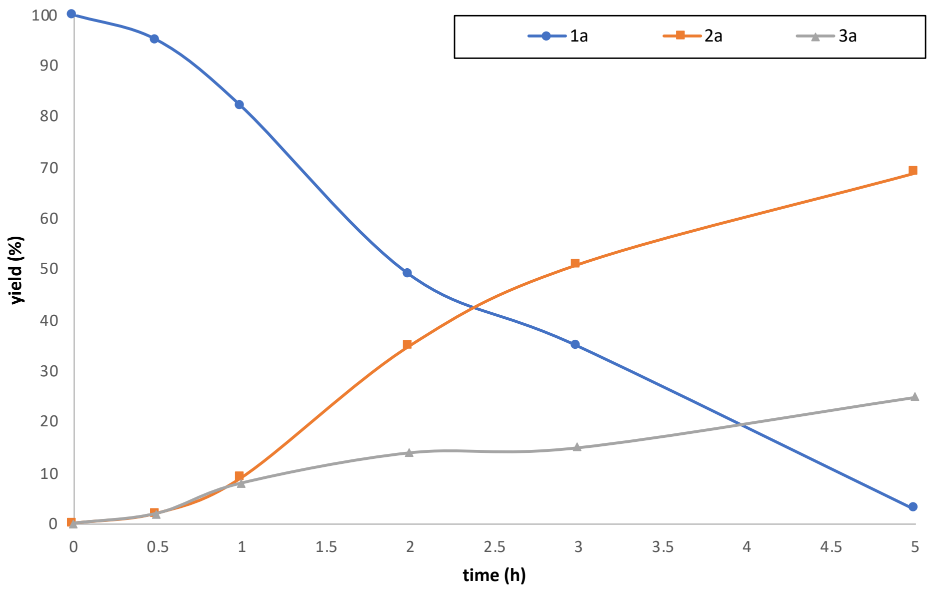

Supplementary Figure 5. Time course analysis of hydrocarboxylation and hydrogenation of **1a**.

6.9 Observation of Phosphonium Salt 10 under the Catalytic Reaction Conditions

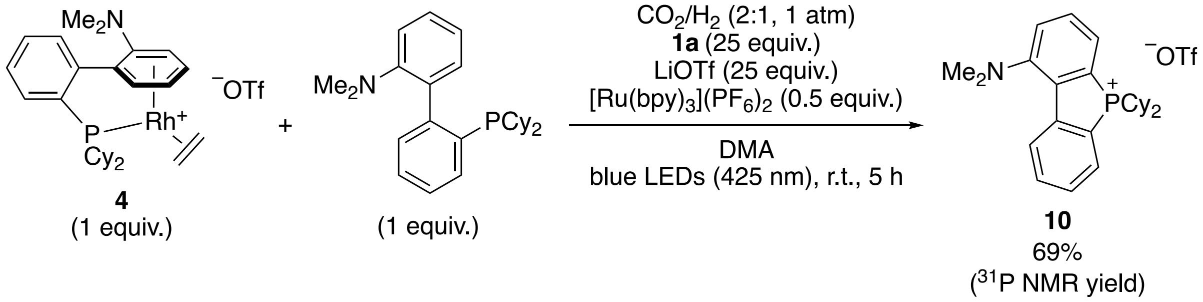

In an argon filled glovebox, [Rh(C_2H_4)(DavePhos)](OTf) **4** (1.4 mg, 2 μmol), DavePhos (0.8 mg, 2 μmol), [Ru(bpy)$_3$](PF_6)$_2$ (0.9 mg, 1 μmol), LiOTf (7.8 mg, 0.05 mmol), and **1a** (8.1 mg, 0.05 mmol) were placed in an oven dried test tube (ϕ = 1.7 cm, 18 cm). To the mixture was added anhydrous *N*,*N*-dimethylacetamide (DMA, 1.0 mL). The tube was sealed with a three-way cock and removed from the glovebox. A mix gas of CO_2/H_2 (1 atm) was charged into the glass tube through the three-way cock. The mixture was then subjected to a blue LED (425 nm) irradiation (two sockets) with vigorous stirring. After 5 h, triphenylphosphine oxide (2.8 mg, 10 μmol) was added to the reaction mixture, and the reaction mixture was transferred to a J. Young NMR tube under an Ar atmosphere. The yield of compound **10** (69%) was determined by ^{31}P NMR using triphenylphosphine oxide as an internal standard. The yield was based on the sum of the amounts of **4** and DavePhos.

6.10 Catalytic Reactivity of Rhodium Acetate 7 and Benzylrhodium 9

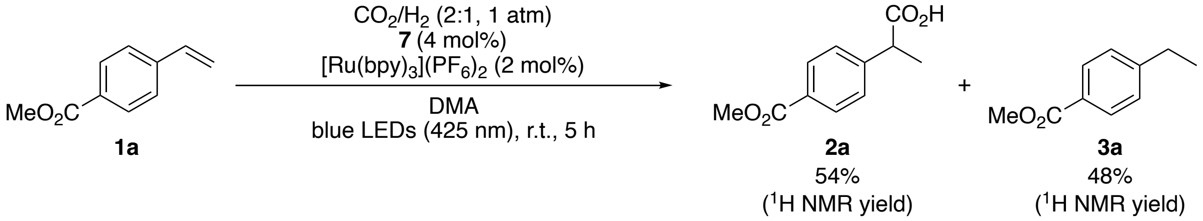

In an argon filled glovebox, **7** (1.1 mg, 2 μmol), [Ru(bpy)$_3$](PF$_6$)$_2$ (0.9 mg, 1 μmol), and **1a** (8.1 mg, 0.05 mmol) were placed in an oven dried test tube (ϕ = 1.7 cm, 18 cm). To the mixture was added anhydrous *N*,*N*-dimethylacetamide (DMA, 1.0 mL). The tube was sealed with a three-way cock and removed from the glovebox. A mix gas of CO_2/H_2 (2:1, 1 atm) was charged into the glass tube through the three-way cock. The mixture was then subjected to a blue LED (425 nm) irradiation (two sockets) with vigorous stirring. After 5 h, the reaction mixture was diluted with Et_2O (5.0 mL) and 1 N HCl aq. (1.0 mL) was added to the mixture. The organic layer was extracted with Et_2O three times. The organic layer was dried over $MgSO_4$. After removal of the solvent under reduced pressure, CH_2Br_2 (3.5 μL) was added to the crude product as an internal standard. The yields of **2a** and **3a** were calculated based on the integration of ^{1}H NMR spectra in $CDCl_3$.

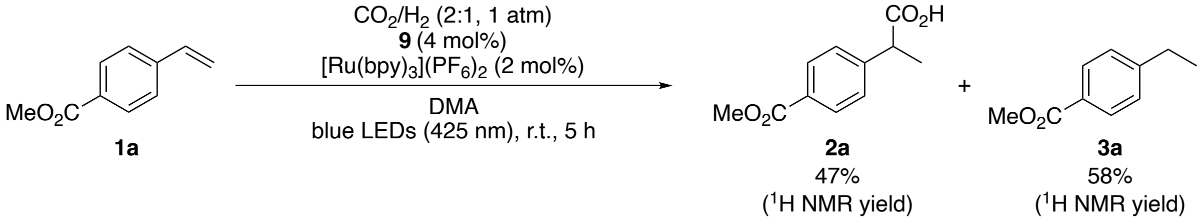

In an argon filled glovebox, **9** (1.3 mg, 2 μmol), [Ru(bpy)$_3$](PF$_6$)$_2$ (0.9 mg, 1 μmol), and **1a** (7.8 mg, 48 μmol) were placed in an oven dried test tube (ϕ = 1.7 cm, 18 cm). To the mixture was added anhydrous *N*,*N*-dimethylacetamide (DMA, 1.0 mL). The tube was sealed with a three-way cock and removed from the glovebox. A mix gas of CO_2/H_2 (2:1, 1 atm) was charged into the glass tube through the three-way cock. The mixture was then subjected to a blue LED (425 nm) irradiation (two sockets) with vigorous stirring. After 5 h, the reaction mixture was diluted with Et_2O (5.0 mL) and 1 N HCl aq. (1.0 mL) was added to the mixture. The organic layer was extracted with Et_2O three times. The organic layer was dried over $MgSO_4$. After removal of the solvent under reduced pressure, CH_2Br_2 (3.5 μL) was added to the crude product as an internal standard. The yields of **2a** and **3a** were calculated based on the integration of ^{1}H NMR spectra in $CDCl_3$. The yields were based on the sum of the amounts of **1a** and **9**.

6.11 Protonation of benzylrhodium 9

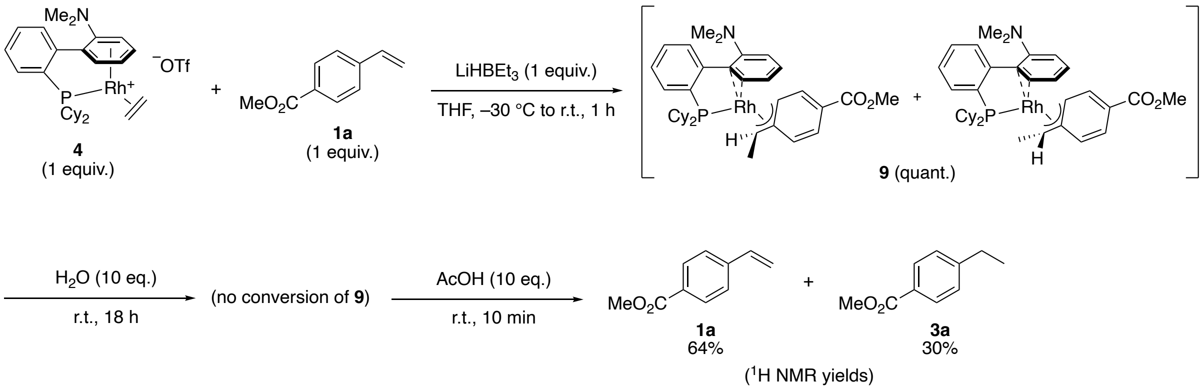

[Rh(C_2H_4)(DavePhos)](OTf) **4** (3.4 mg, 5 μmol), **1a** (0.8 mg, 5 μmol), and THF (0.5 mL) were added in a J. Young NMR tube under an Ar atmosphere. $LiHBEt_3$ (0.89 M in THF) was added to the solution at –30 °C, and the mixture was gradually warmed to room temperature over 1 h. Full conversion of **4** to **9** was confirmed by ^{31}P NMR. To the mixture was added H_2O (0.9 μL, 50 μmol), and the dark brown solution was left for 18 h at room temperature. No reaction of **9** was observed in ^{31}P NMR. Then, AcOH (2.9 μL, 50μmol) was added to the solution. The solution became pale brown immediately. After 10 min, the mixture was evaporated in vacuo to give the crude product. The yields of **1a** (64%) and **3a** (30%) were determined by ^{1}H NMR in $CDCl_3$ using 1,1,2,2-tetrachloroethane (1.0 μL) as an internal standard.

7. X-ray Diffraction Analysis.

A single crystal in immersion oil was mounted on a Rigaku XtaLAB Synergy-DW diffractometer with a HyPix-6000HE HPC detector. The diffraction data were collected using Cu *K*α radiation under a cold nitrogen stream at 123 K. The images were processed with the Rigaku CrysAlis^{PRO} software. The structure was solved by a direct method and refined on F^2 by a least-squares method by the programs SHELXT2015[17] and SHELXL2015,[18] respectively. All the non-hydrogen atoms were refined anisotropically. All the hydrogen atoms were put on the calculated geometry and refined by applying riding models.

**Compound**	**4**	**8**
Solvent for recrystallization	CH_2Cl_2/Et_2O (vapor diffusion)	C_6D_6/hexane (vapor diffusion)
Formula	$C_{29}H_{40}F_3NO_3PRhS$	$C_{26}H_{36}NPRh$
Formula weight	673.56	496.44
Crystal system	Triclinic	Monoclinic
Space group	$P\bar{1}$	$P\,2_1/n$
Crystal size (mm)	0.088×0.039×0.037	0.149×0.074×0.055
Crystal color and shape	Yellow block	Brown block
Wavelength (Å)	1.54184	1.54184
a (Å)	9.1982(2)	11.5636(8)
b (Å)	10.7195(2)	16.5707(10)
c (Å)	15.1783(6)	12.9433(9)
α (°)	97.970(3)	90
β (°)	94.176(3)	114.492(8)
γ (°)	95.477(2)	90
Volume (Å)	1469.73(7)	2257.0(3)
Z	2	4
$\rho_{calcd.}$ (g cm^{-3})	1.522	1.461
μ (mm^{-1})	6.309	6.861
θ_{min}, θ_{max} (°)	4.175, 75.000	2.631, 75.465
No. of reflection (unique)	17594 (5867)	16850 (4533)
R_{int}	0.0434	0.0452
Completeness to θ (%)	100.0	99.8
Goodness-of-fit on F^2	1.043	1.131
Final R_1 and wR_2 indices [$I > 2\sigma(I)$]	0.0453, 0.1067	0.0497, 0.1325
R_1 and wR_2 indices (all data)	0.0519, 0.1100	0.0567, 0.1365
CCDC number	2160650	2160651

Note: The crystallographic data for compound **4** contain one level B alert.

```
PLAT230_ALERT_2_B Hirshfeld Test Diff for    C00G     --C00O     . 14.0 s.u.
```

Author Response: The anomalous data for Hirshfeld test do not indicate an incorrect atom-type assignment, according to other characterizations.

8. Computational details

All DFT calculations were carried out with the Gaussian 16 program,[19] except for calculation of minimum energy crossing point (MECP). MECP was computed by GRRM11[20–24] based on the Gaussian 16 program. An ultrafine integral grid was employed for all the calculation. Geometry optimizations for the ground state S_0 and the excited state T_1 were performed at 298.15 K in DMA with M06[25]/6-31G(d) (C, H, N, O, P) & SDD (Rh). The vibrational frequencies were computed at the same level to check whether each optimized structure was an energy minimum (no imaginary frequency) or a transition state (single imaginary frequency). Intrinsic reaction coordinates (IRC) calculations were performed to track minimum energy paths from transition structures to the corresponding local minima. Single-point energies were calculated with M06/6-311+G(d,p) (C, H, N, O, P) & SDD (Rh) based on the optimized structure. The solvation with DMA was evaluated by the self-consistent reaction field (SCRF) method using the solvation model based on density (SMD). [26]

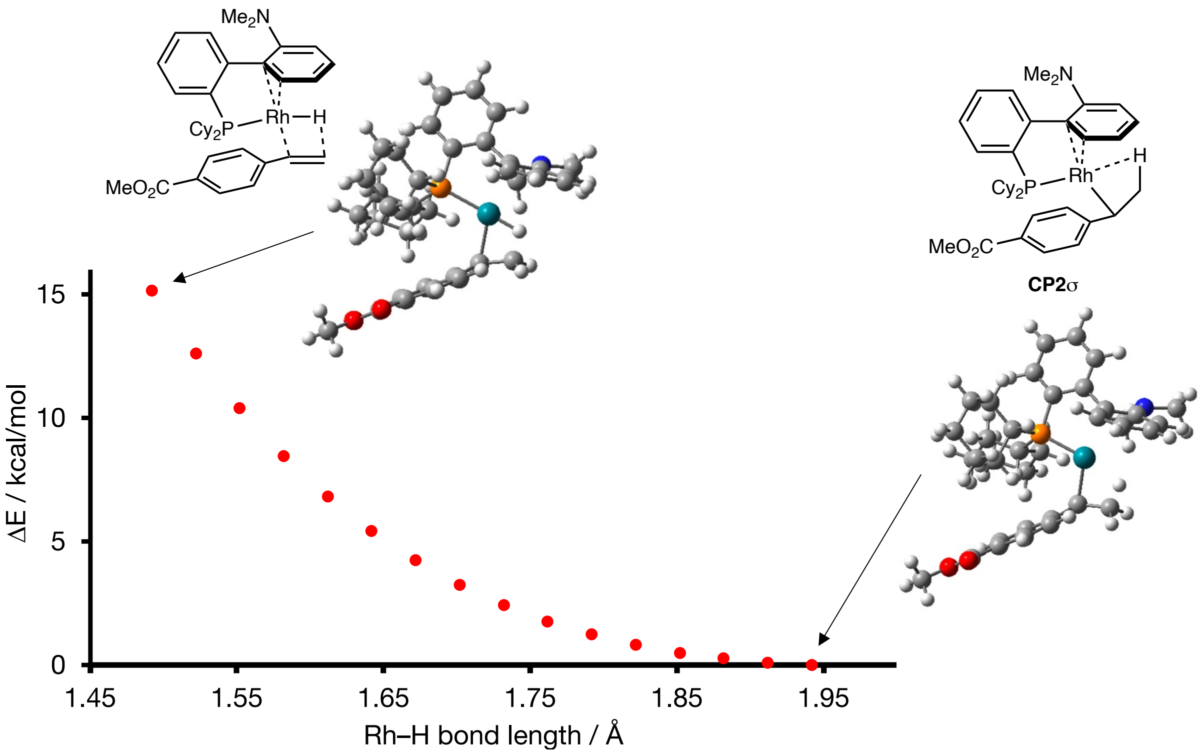

Supplementary Figure 6. Energy diagram of a reverse scan along Rh–H bond from **CP2 σ** at the M06/6-31G(d) (C, H, N, O, P) & SDD (Rh) level of theory.

9. Spectral Data

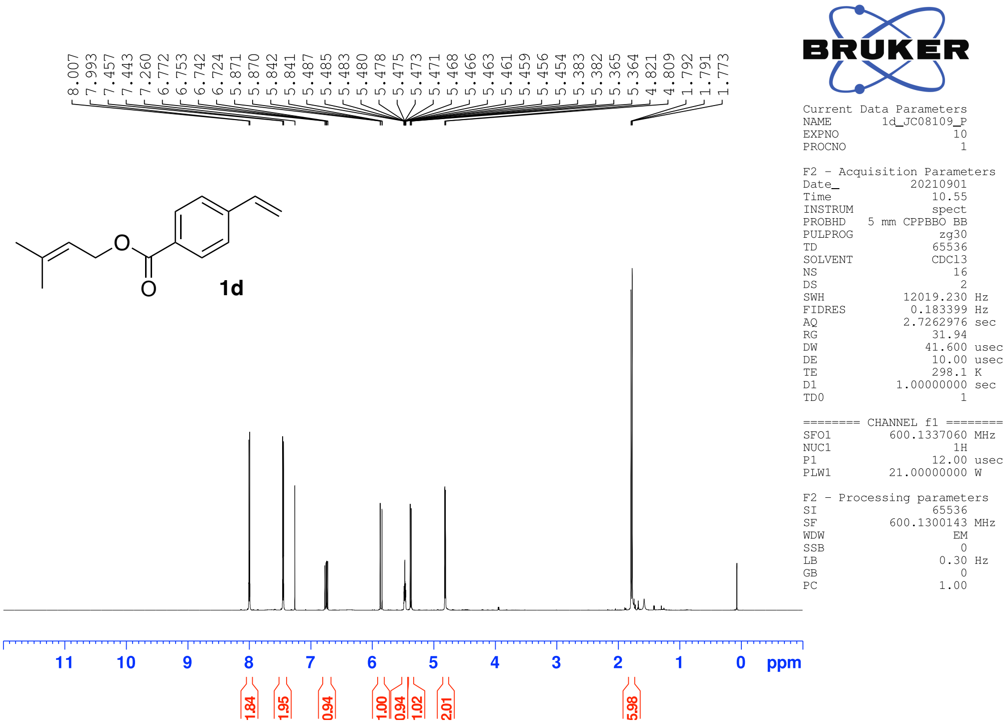

Supplementary Figure 7. ^{1}H NMR spectrum of **1d** (600 MHz, r.t., $CDCl_3$)

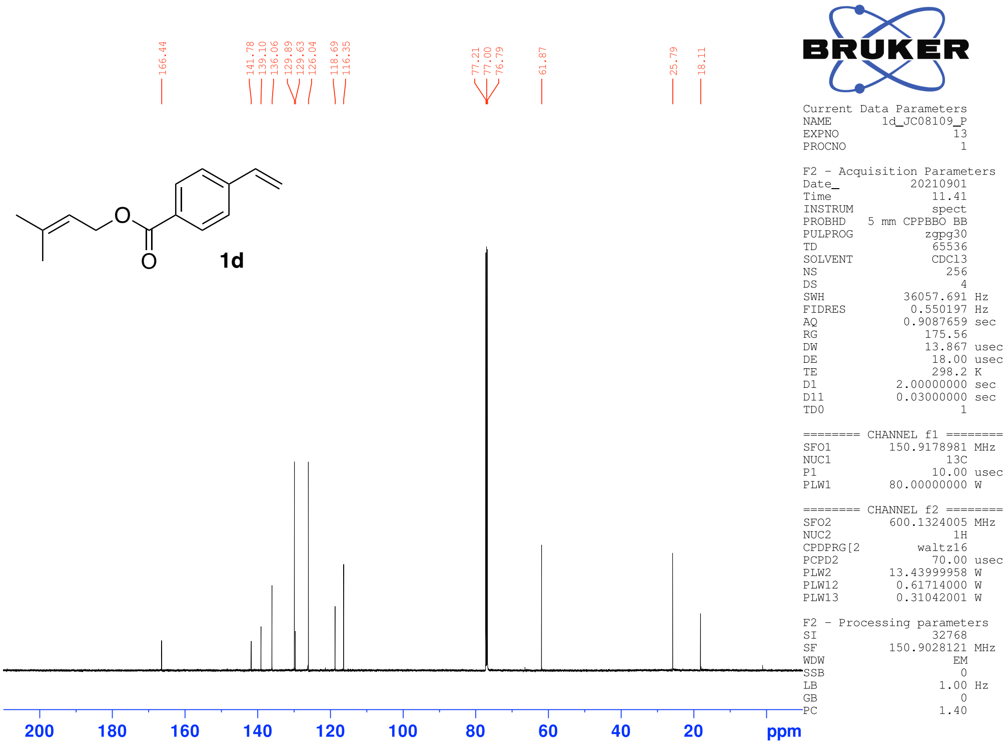

Supplementary Figure 8. ^{13}C NMR spectrum of **1d** (151 MHz, r.t., $CDCl_3$)

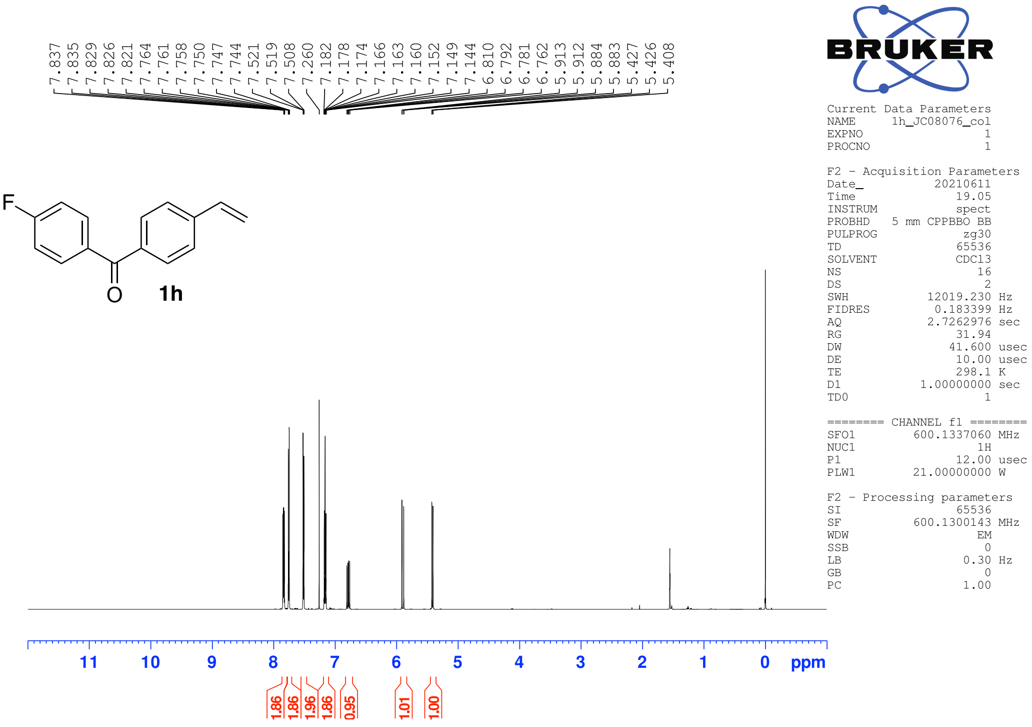

Supplementary Figure 9. ^{1}H NMR spectrum of **1h** (600 MHz, r.t., $CDCl_3$)

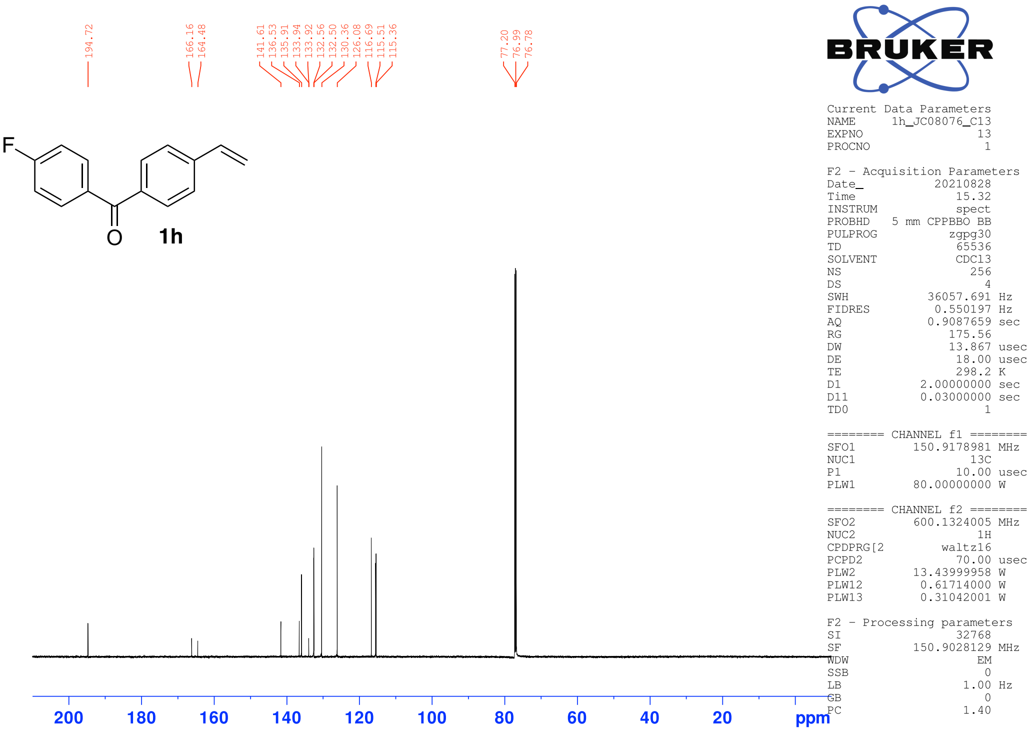

Supplementary Figure 10. ^{13}C NMR spectrum of **1h** (151 MHz, r.t., $CDCl_3$)

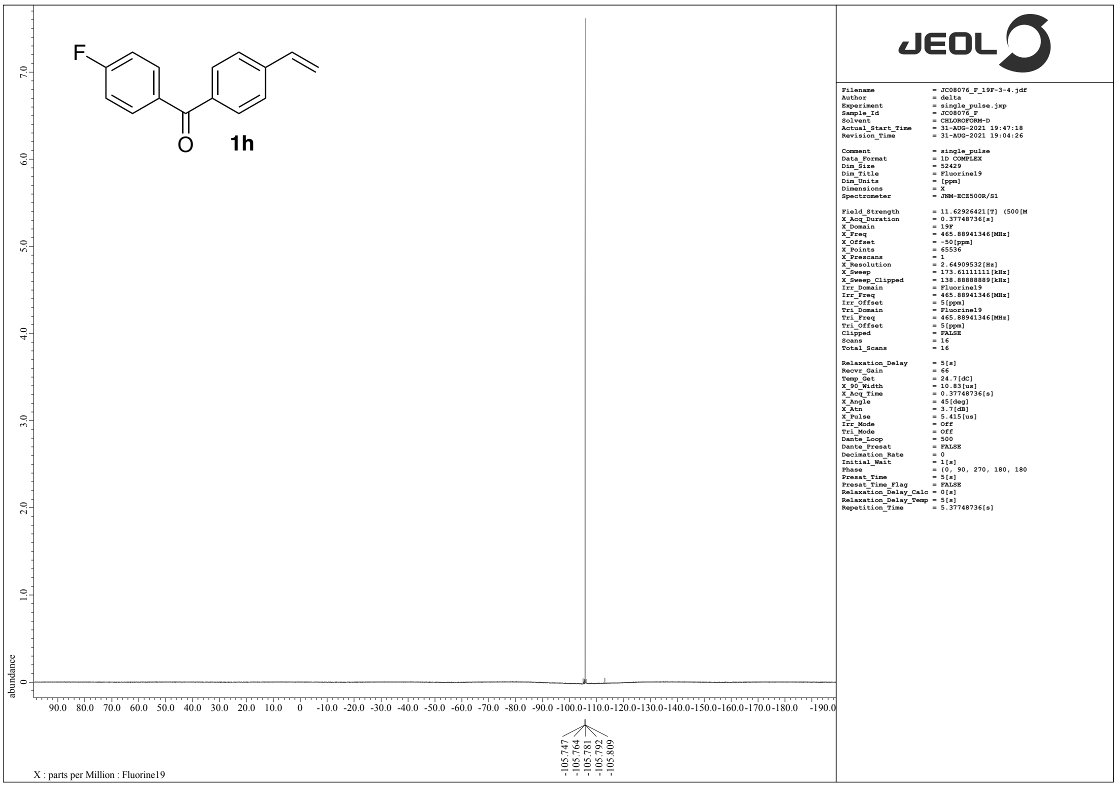

Supplementary Figure 11. ^{19}F NMR spectrum of **1h** (466 MHz, r.t., $CDCl_3$)

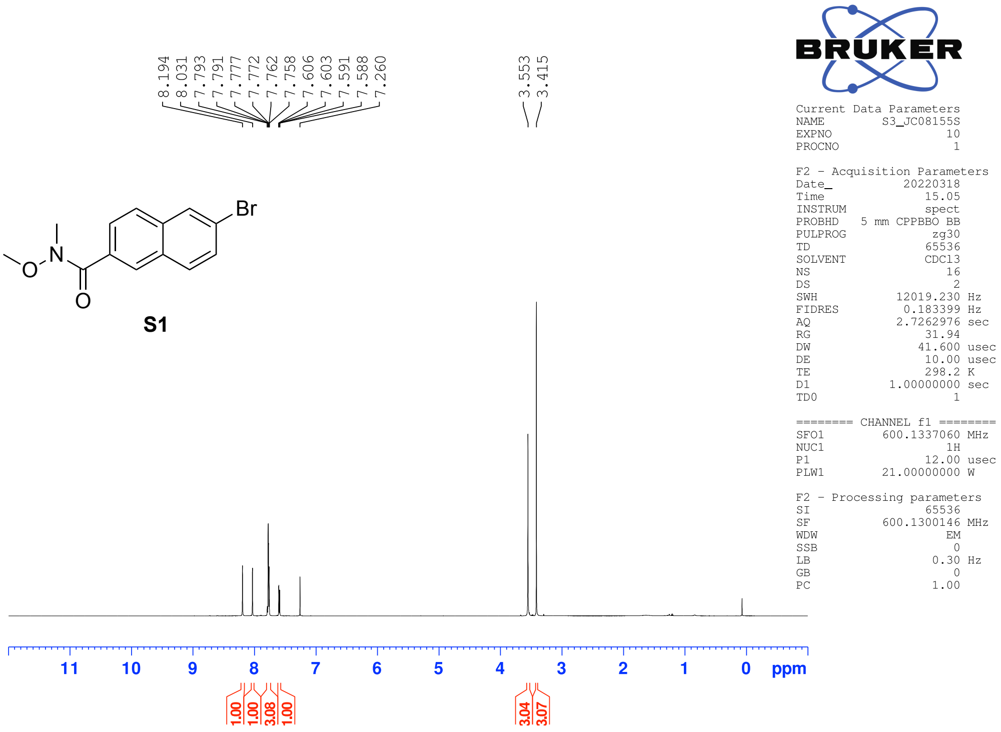

Supplementary Figure 12. ^{1}H NMR spectrum of **S3** (600 MHz, r.t., $CDCl_3$)

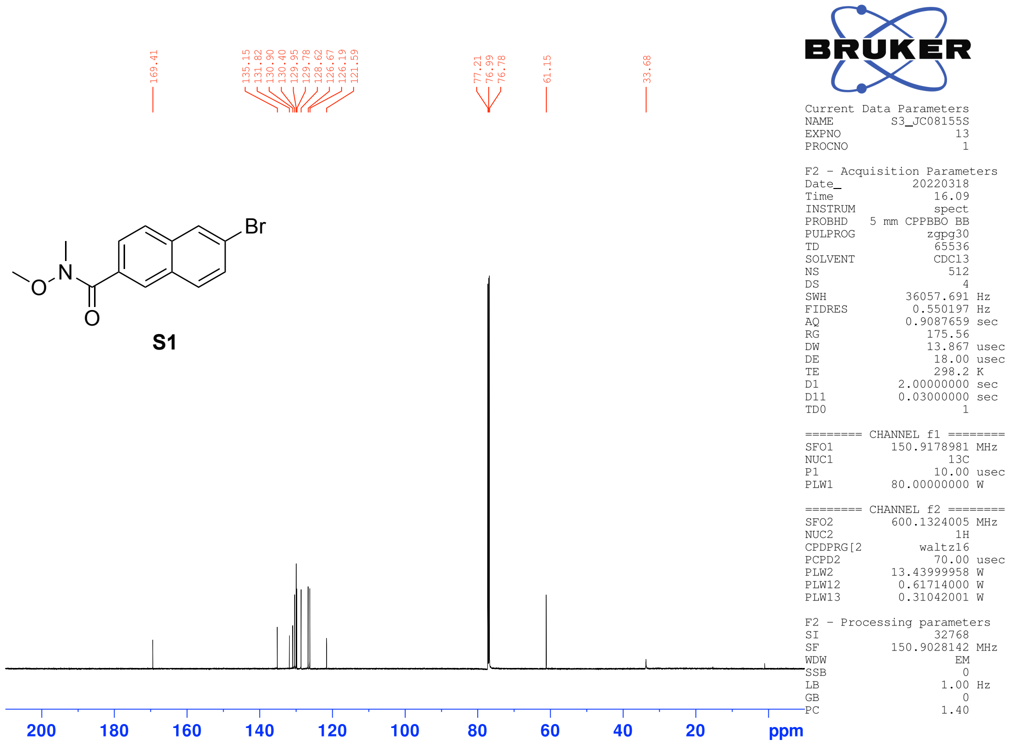

Supplementary Figure 13. ^{13}C NMR spectrum of **S3** (151 MHz, r.t., $CDCl_3$)

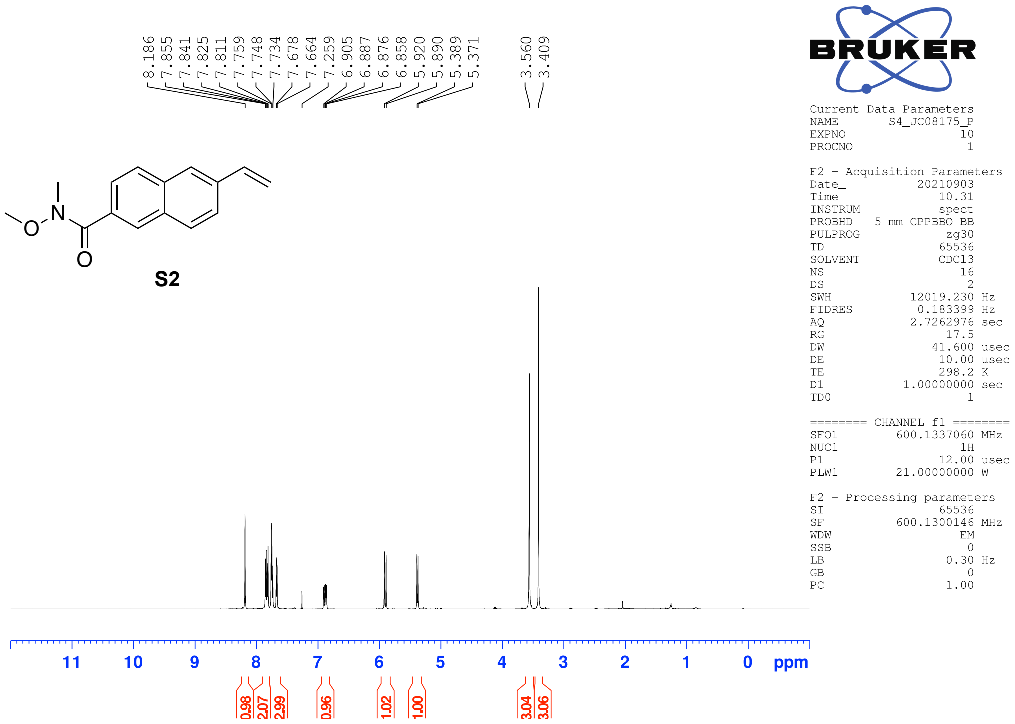

Supplementary Figure 14. ^{1}H NMR spectrum of **S4** (600 MHz, r.t., $CDCl_3$)

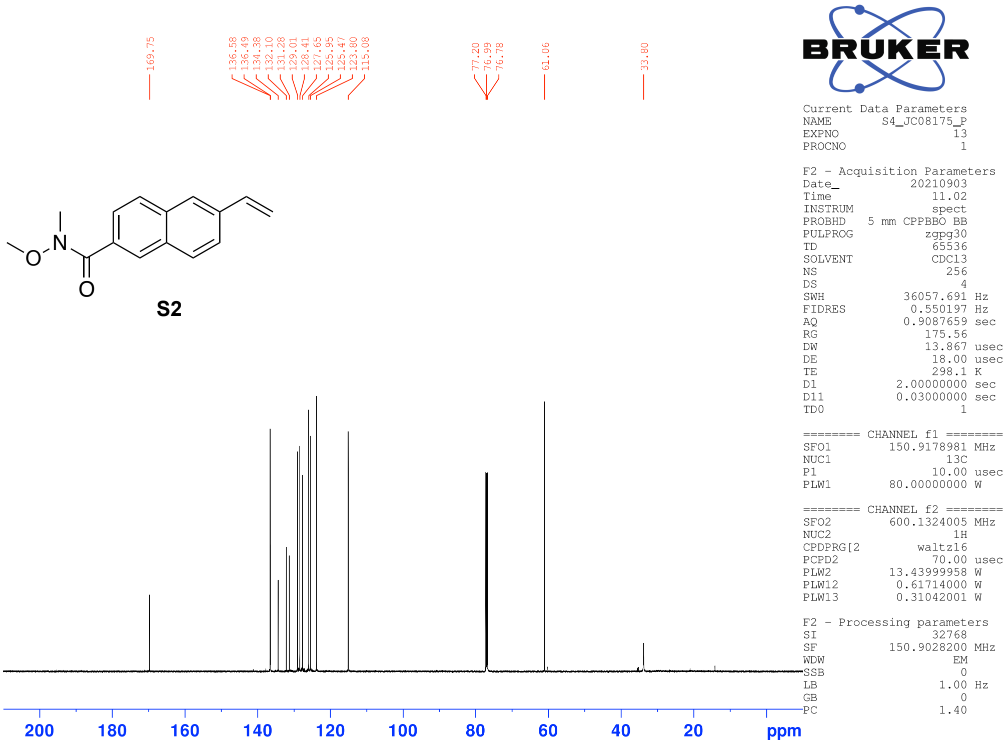

Supplementary Figure 15. ^{13}C NMR spectrum of **S4** (151 MHz, r.t., $CDCl_3$)

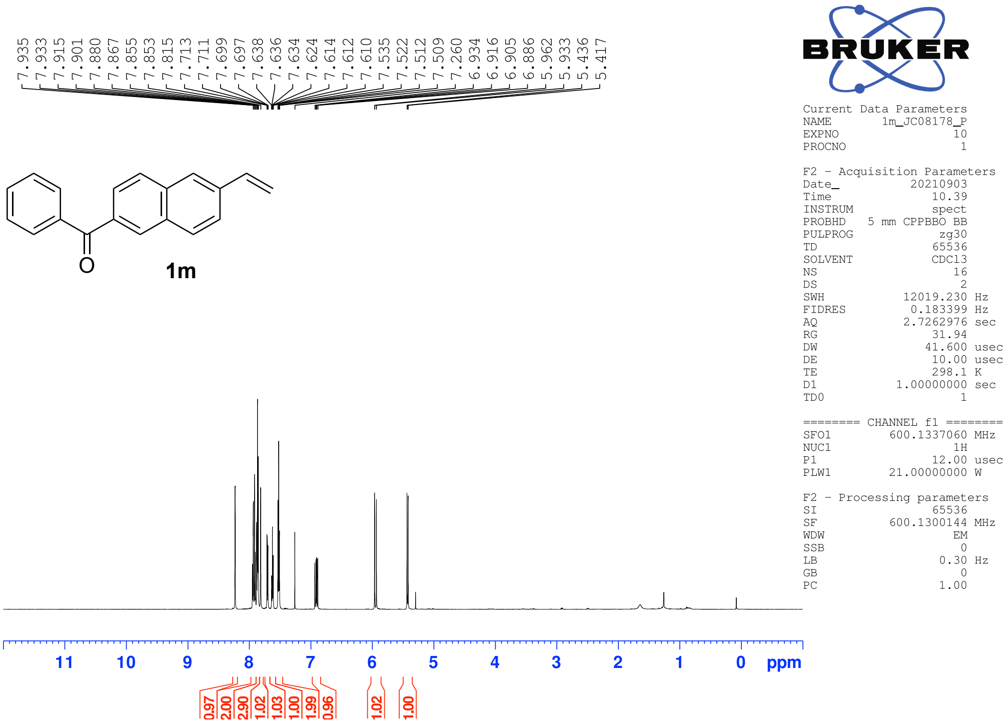

Supplementary Figure 16. ^{1}H NMR spectrum of **1m** (600 MHz, r.t., $CDCl_3$)

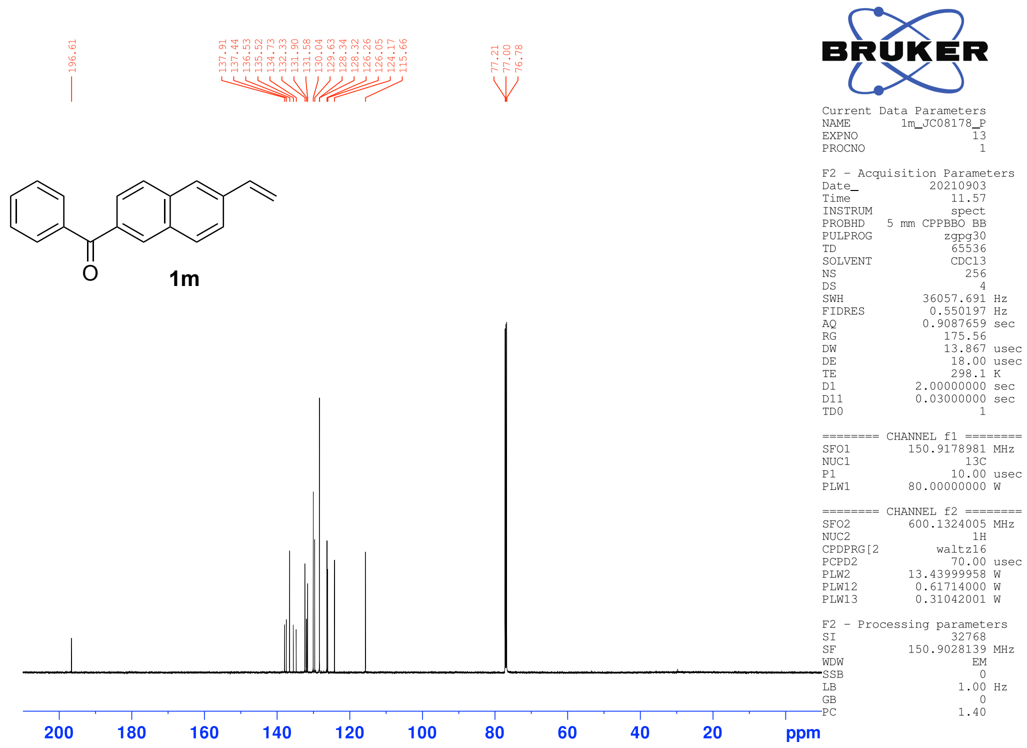

Supplementary Figure 17. ^{13}C NMR spectrum of **1m** (151 MHz, r.t., $CDCl_3$)

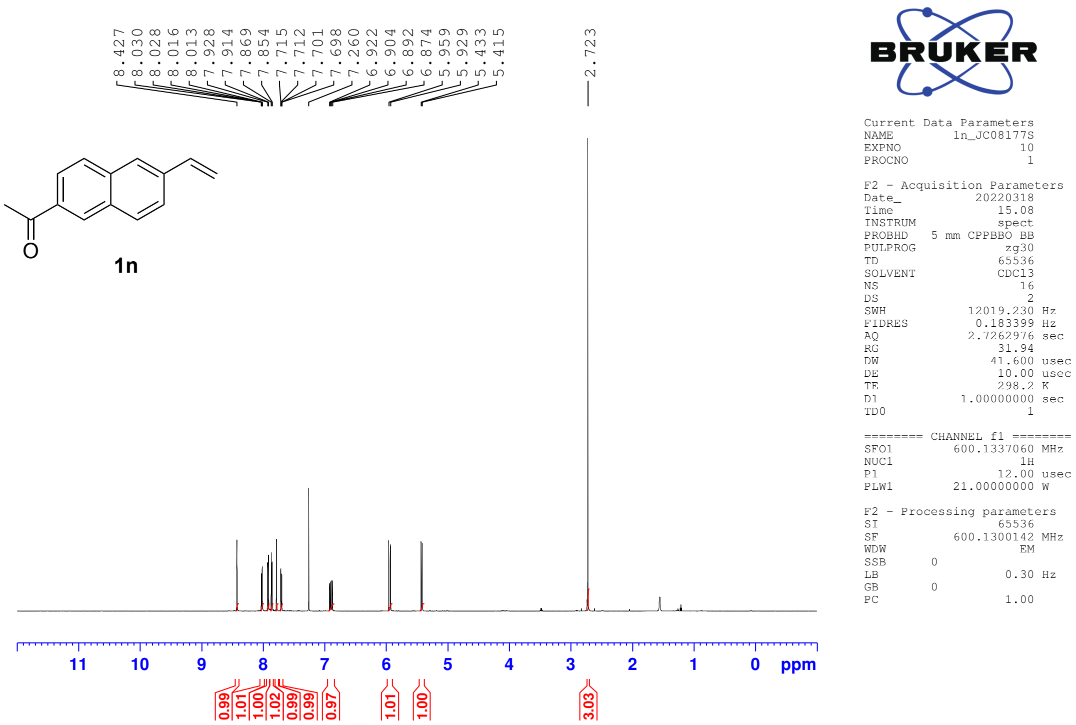

Supplementary Figure 18. ^{1}H NMR spectrum of **1n** (600 MHz, r.t., $CDCl_3$)

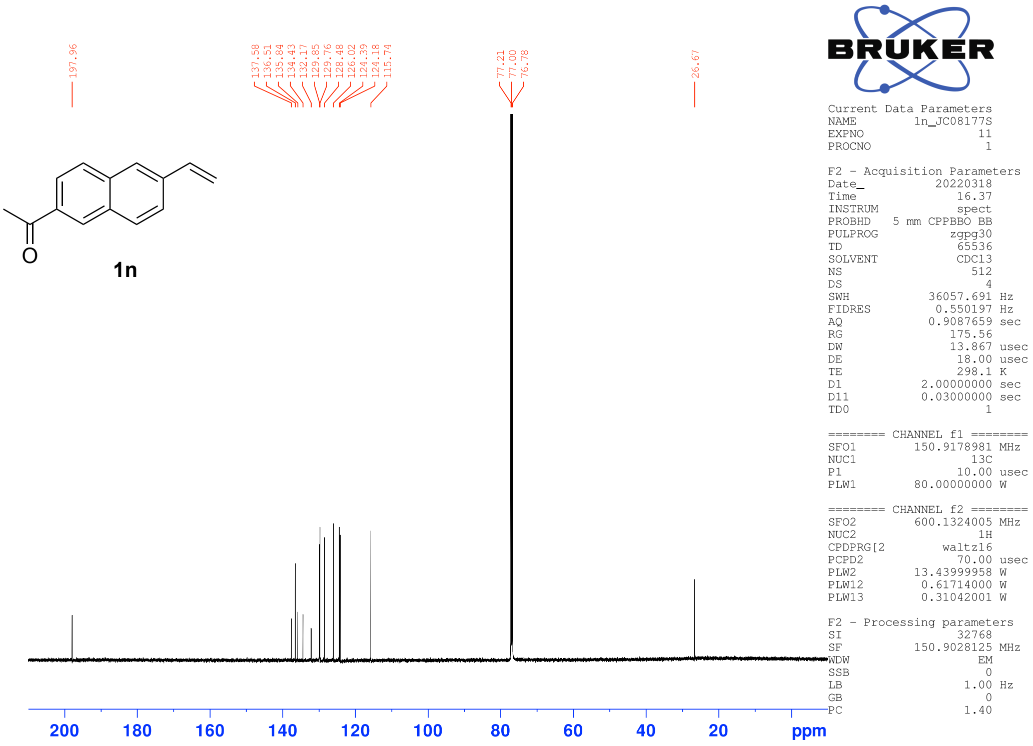

Supplementary Figure 19. ^{13}C NMR spectrum of **1n** (151 MHz, r.t., $CDCl_3$)

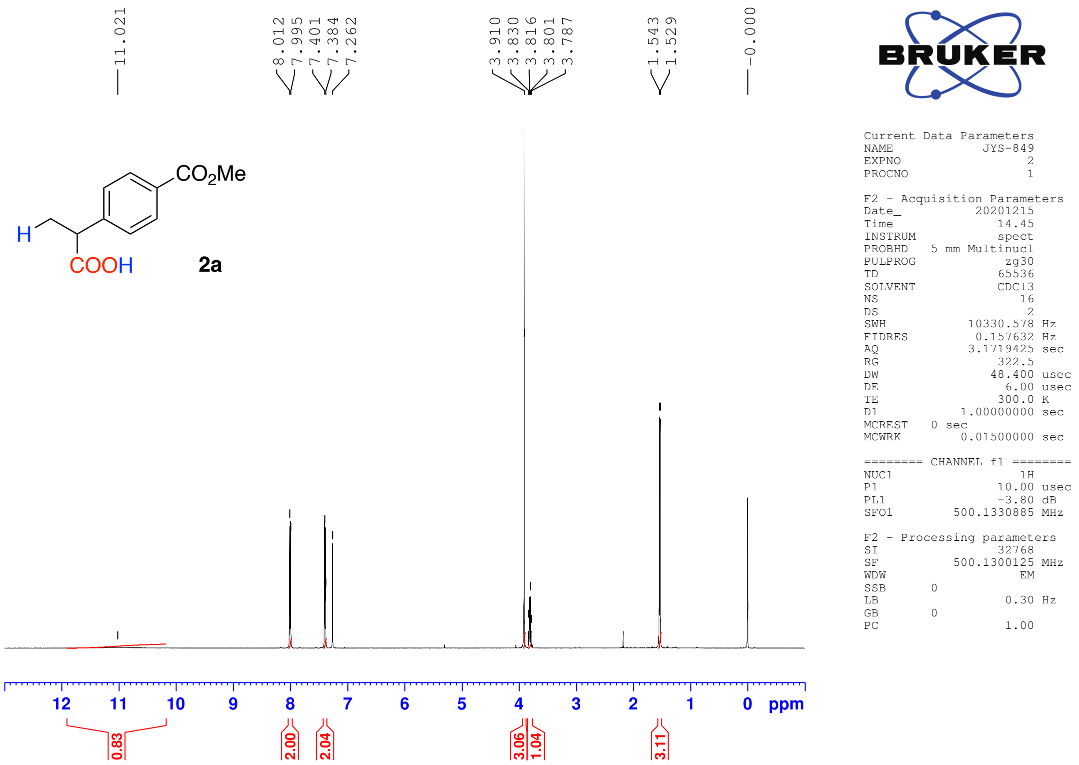

Supplementary Figure 20. ^{1}H NMR spectrum of **2a** (500 MHz, r.t., $CDCl_3$)

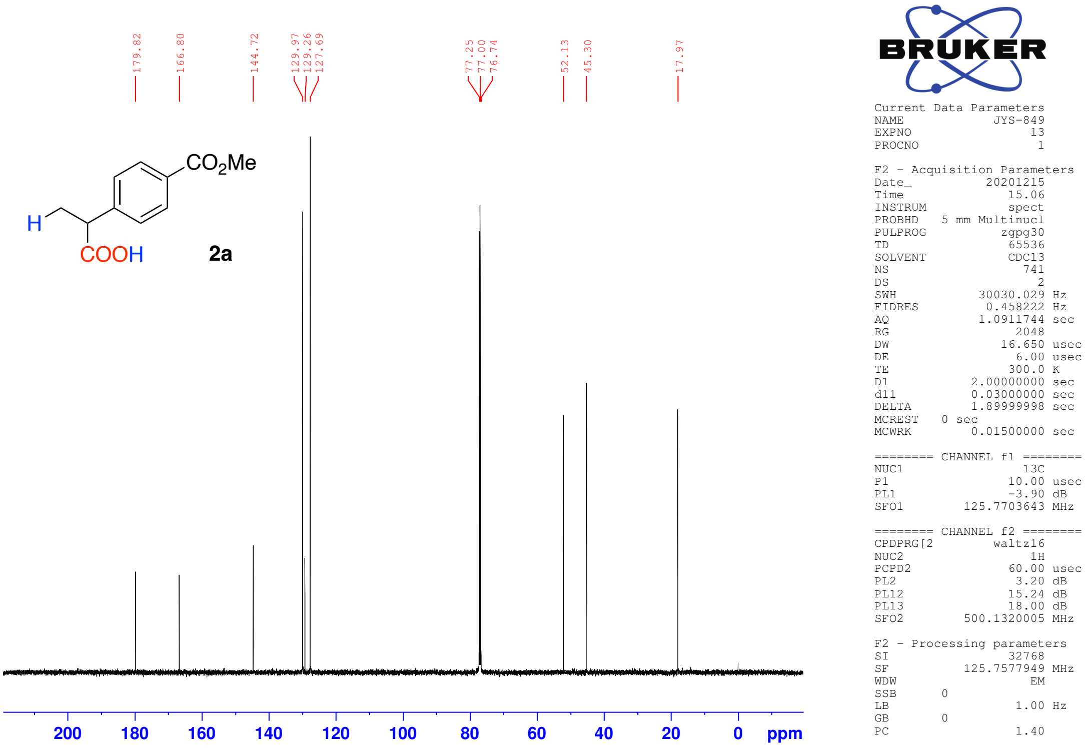

Supplementary Figure 21. ^{13}C NMR spectrum of **2a** (126 MHz, r.t., $CDCl_3$)

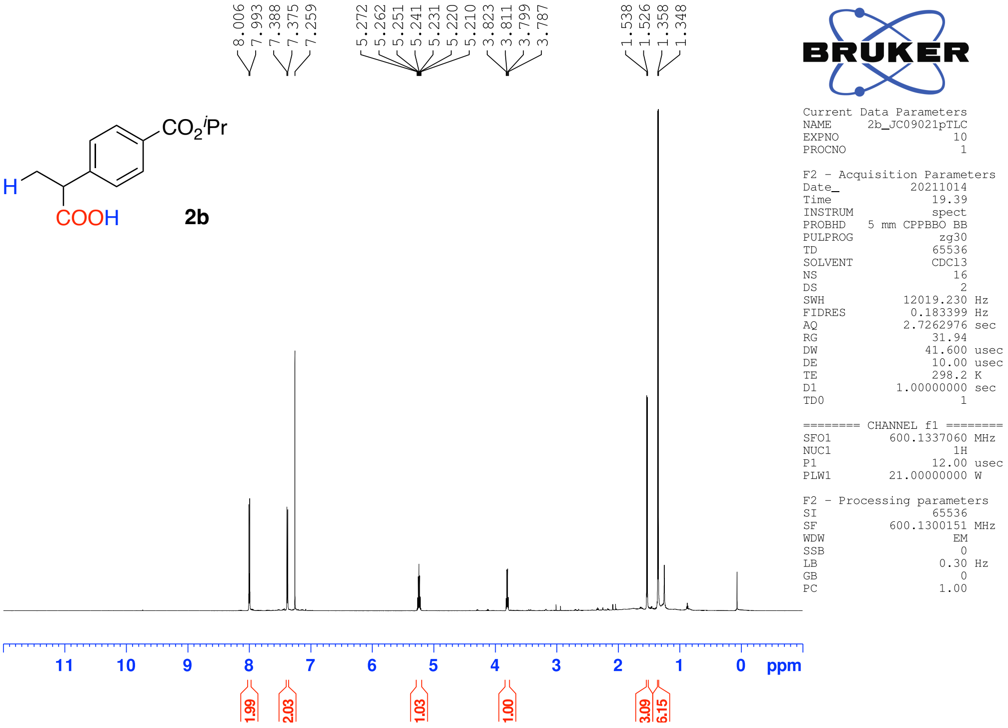

Supplementary Figure 22. ^{1}H NMR spectrum of **2b** (600 MHz, r.t., $CDCl_3$)

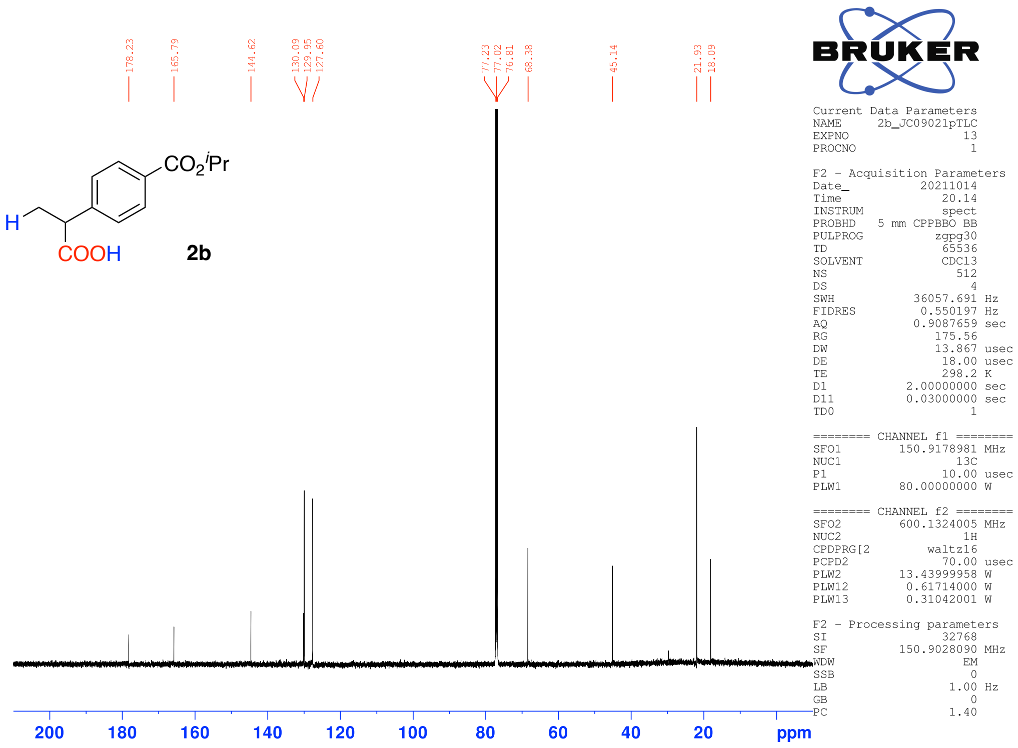

Supplementary Figure 23. ^{13}C NMR spectrum of **2b** (151 MHz, r.t., $CDCl_3$)

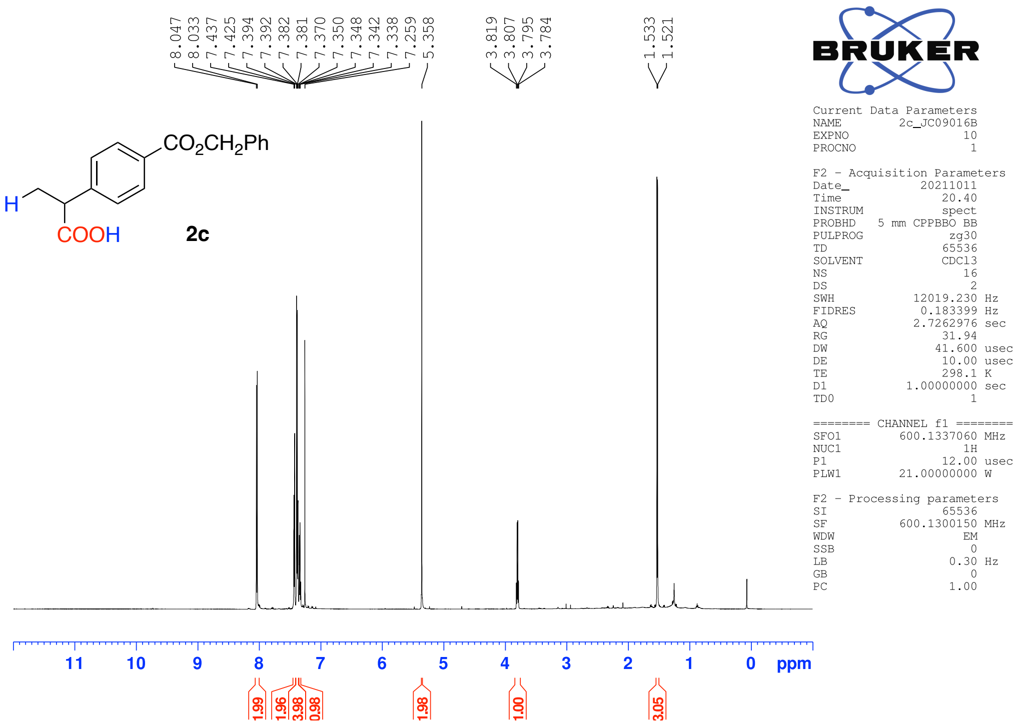

Supplementary Figure 24. ^{1}H NMR spectrum of **2c** (600 MHz, r.t., $CDCl_3$)

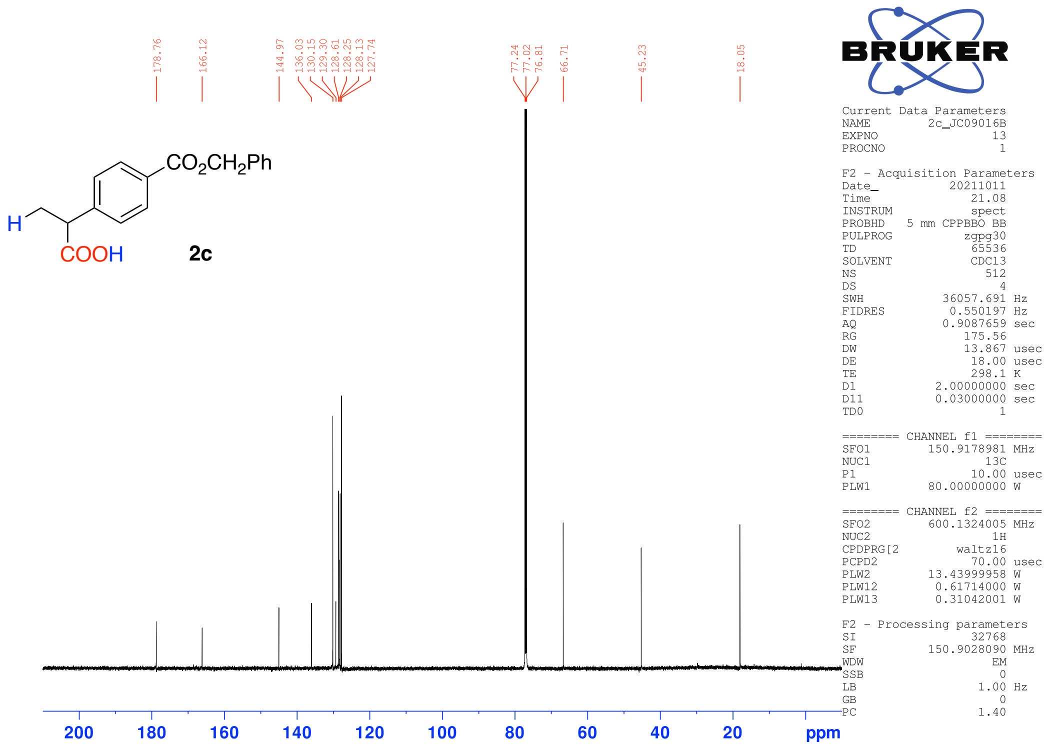

Supplementary Figure 25. ^{13}C NMR spectrum of **2c** (151 MHz, r.t., $CDCl_3$)

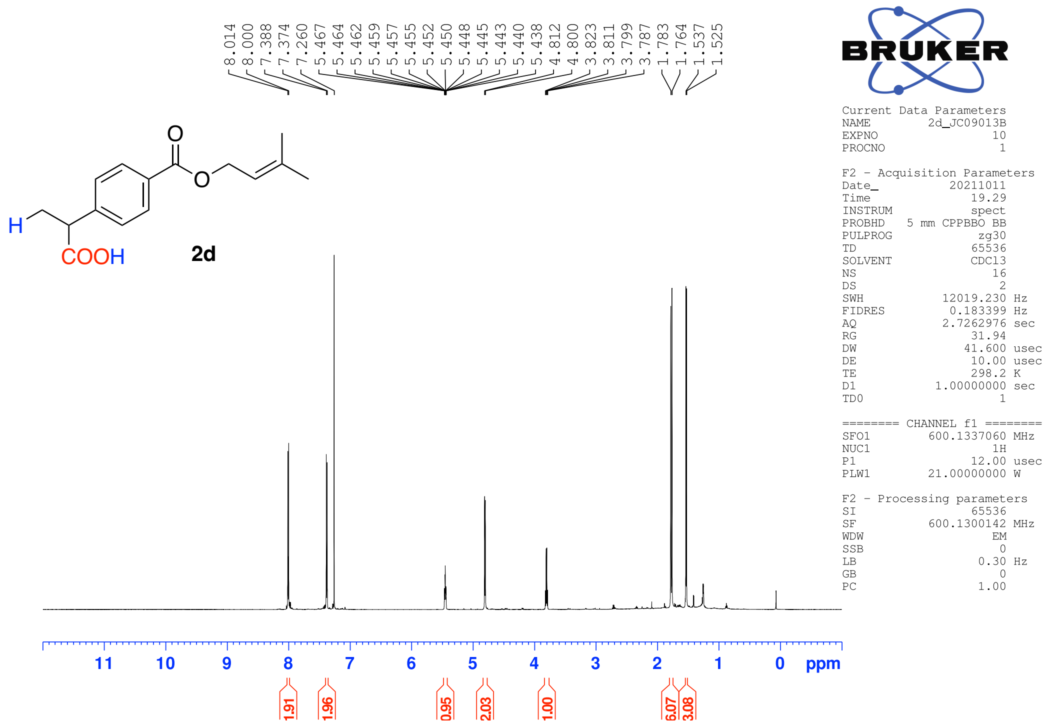

Supplementary Figure 26. ^{1}H NMR spectrum of **2d** (600 MHz, r.t., $CDCl_3$)

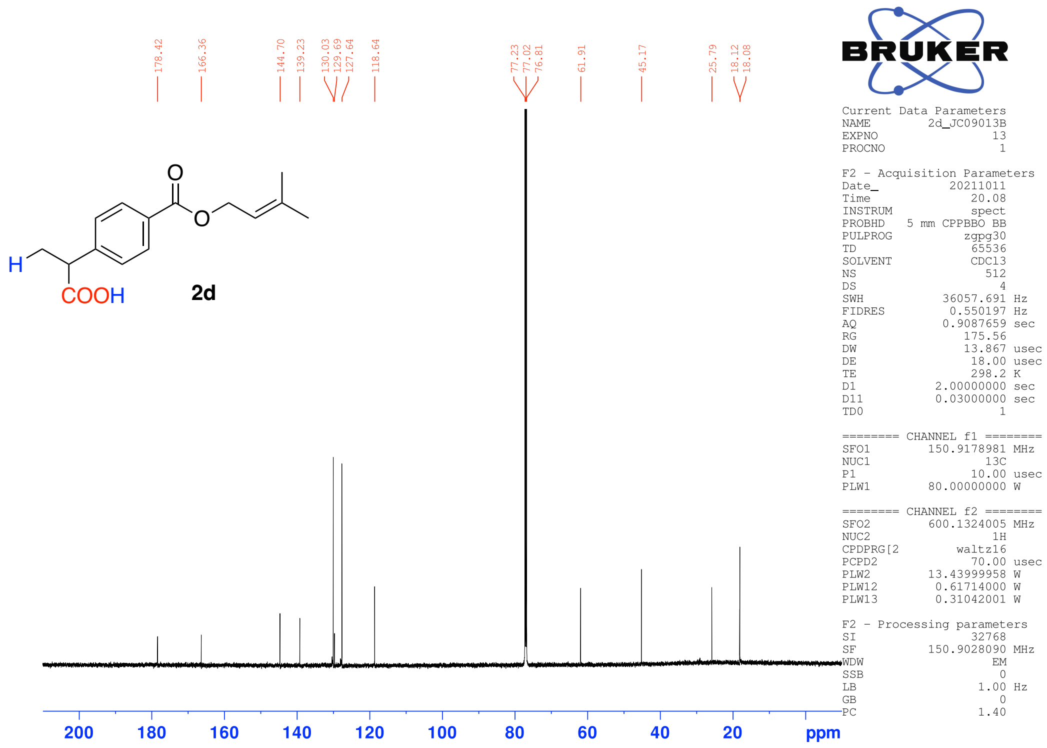

Supplementary Figure 27. ^{13}C NMR spectrum of **2d** (151 MHz, r.t., $CDCl_3$)

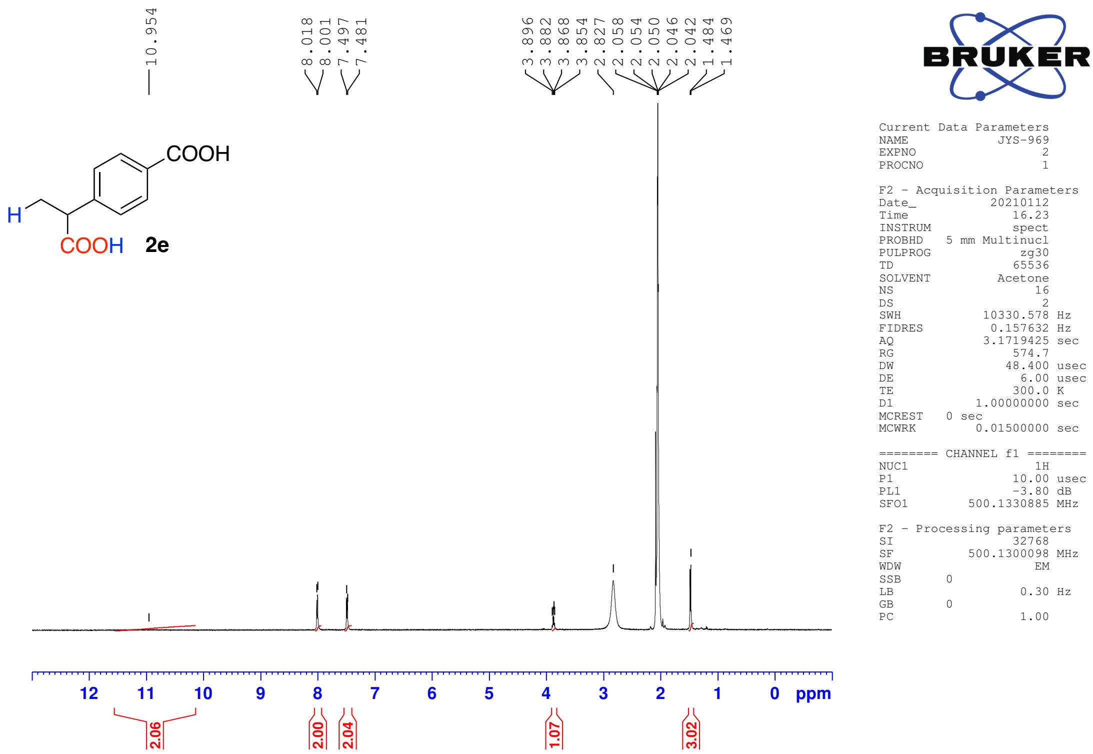

Supplementary Figure 28. ^{1}H NMR spectrum of **2e** (500 MHz, r.t., acetone-d_6)

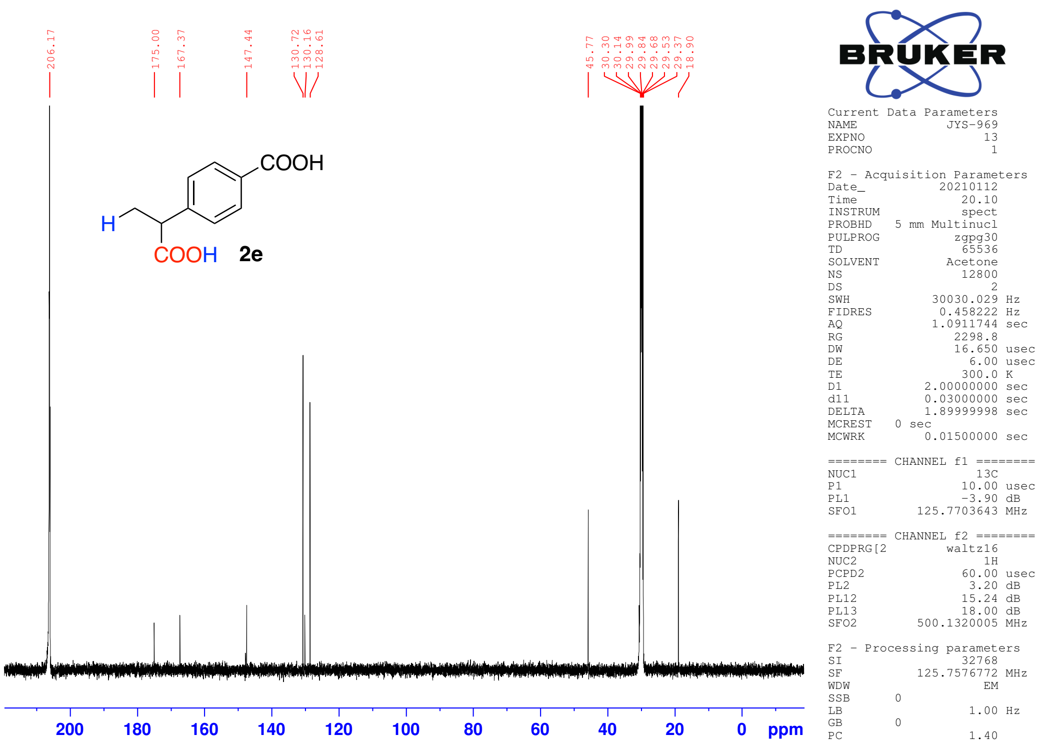

Supplementary Figure 29. ^{13}C NMR spectrum of **2e** (126 MHz, r.t., acetone-d_6)

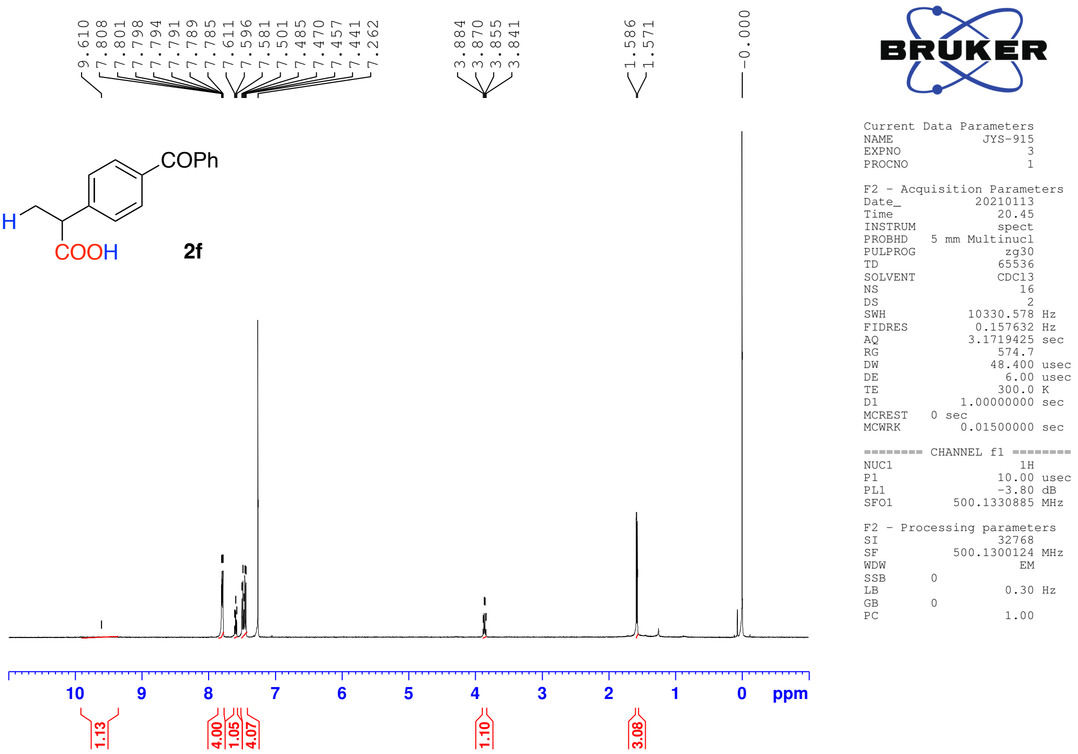

Supplementary Figure 30. ^{1}H NMR spectrum of **2f** (500 MHz, r.t., $CDCl_3$)

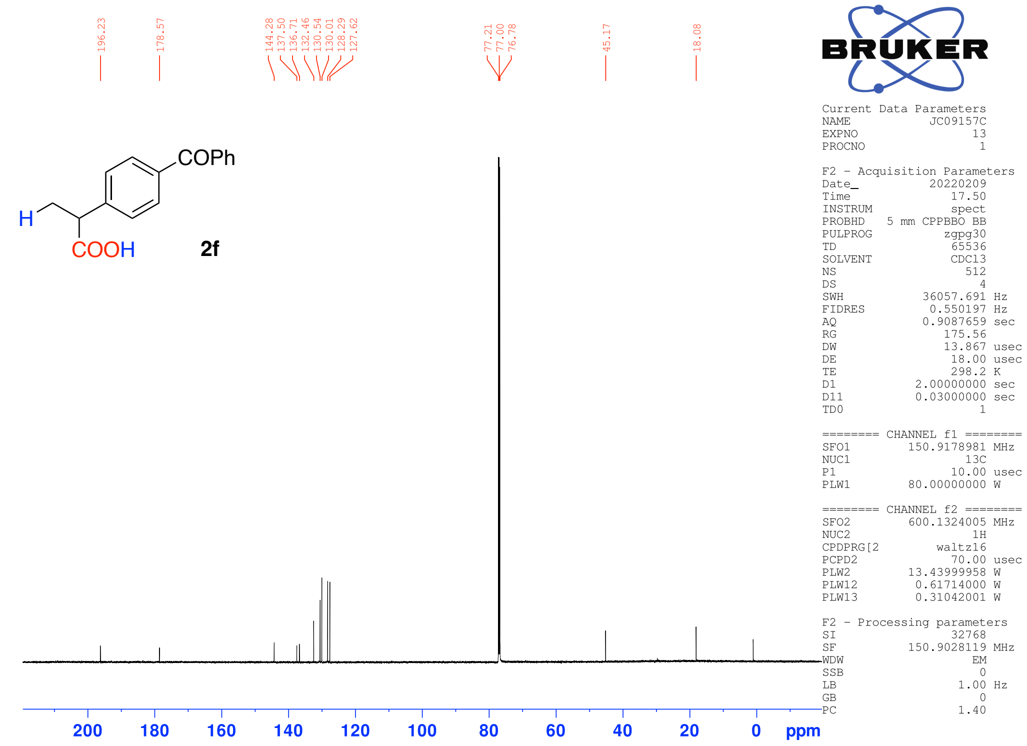

Supplementary Figure 31. ^{13}C NMR spectrum of **2f** (151 MHz, r.t., $CDCl_3$)

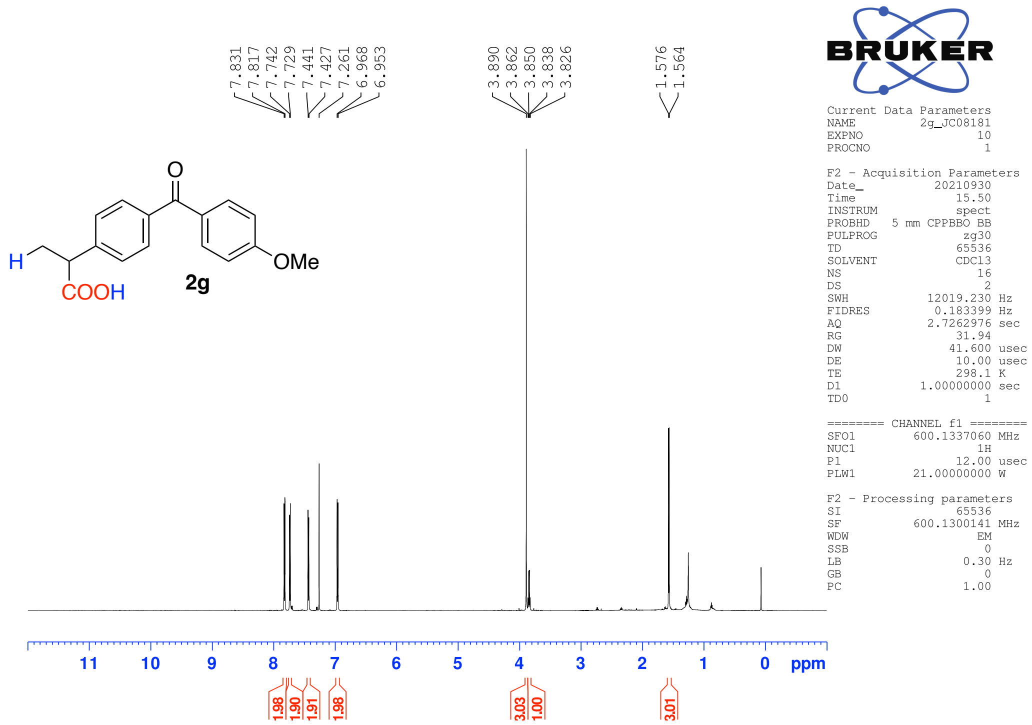

Supplementary Figure 32. ^{1}H NMR spectrum of **2g** (600 MHz, r.t., $CDCl_3$)

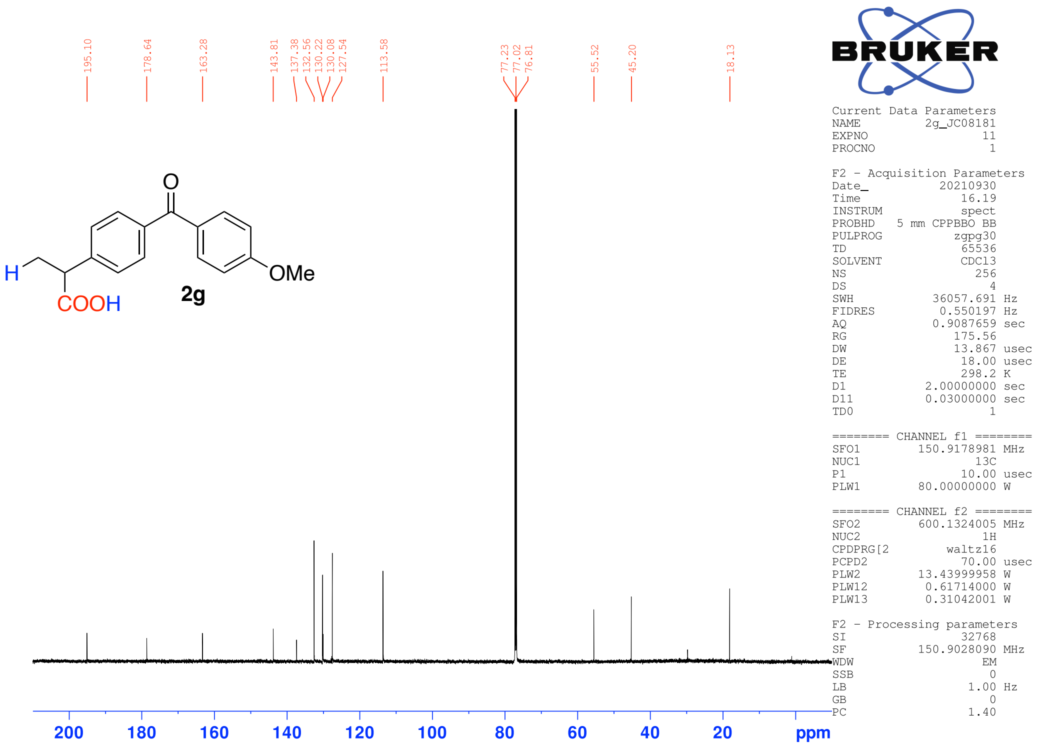

Supplementary Figure 33. ^{13}C NMR spectrum of **2g** (151 MHz, r.t., $CDCl_3$)

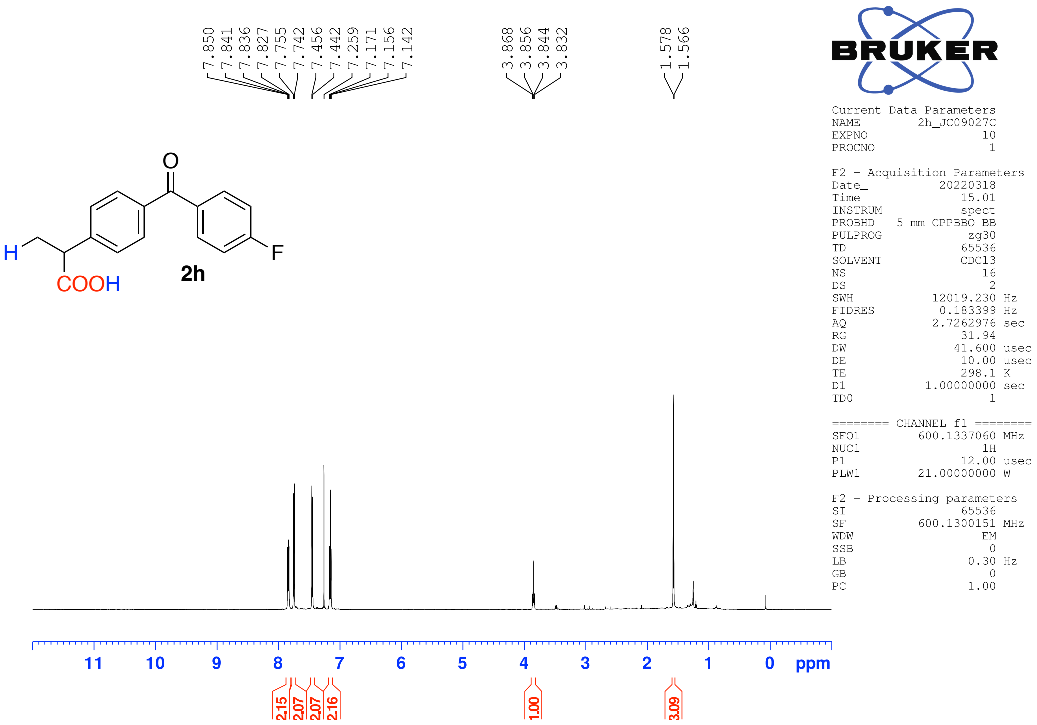

Supplementary Figure 34. ^{1}H NMR spectrum of **2h** (600 MHz, r.t., $CDCl_3$)

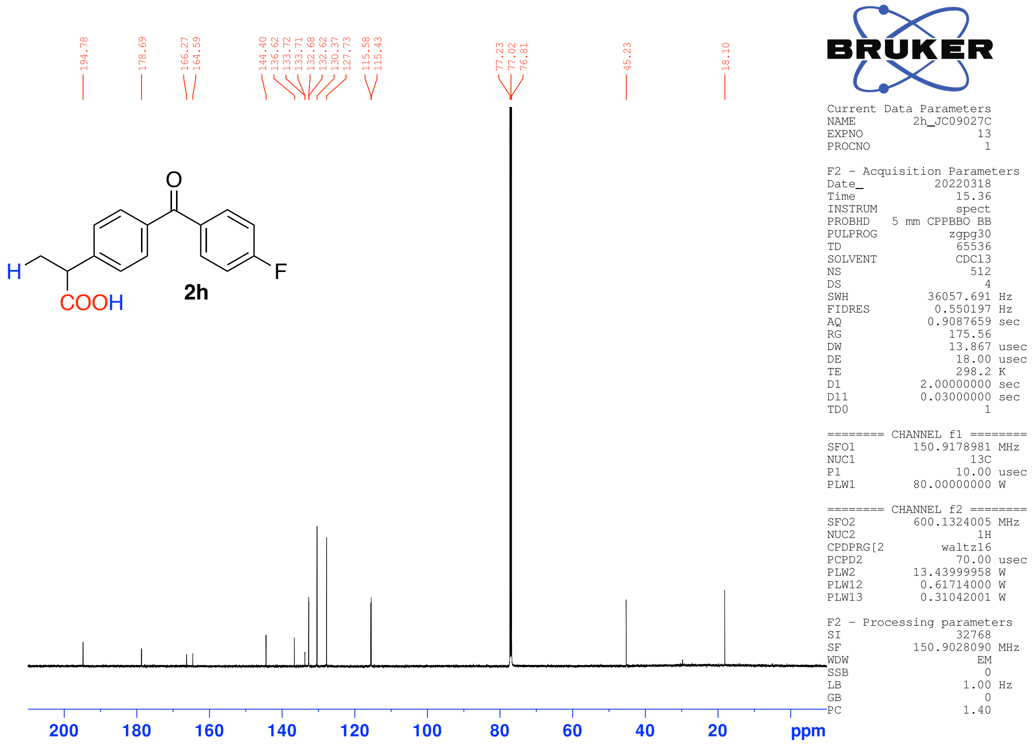

Supplementary Figure 35. ^{13}C NMR spectrum of **2h** (151 MHz, r.t., $CDCl_3$)

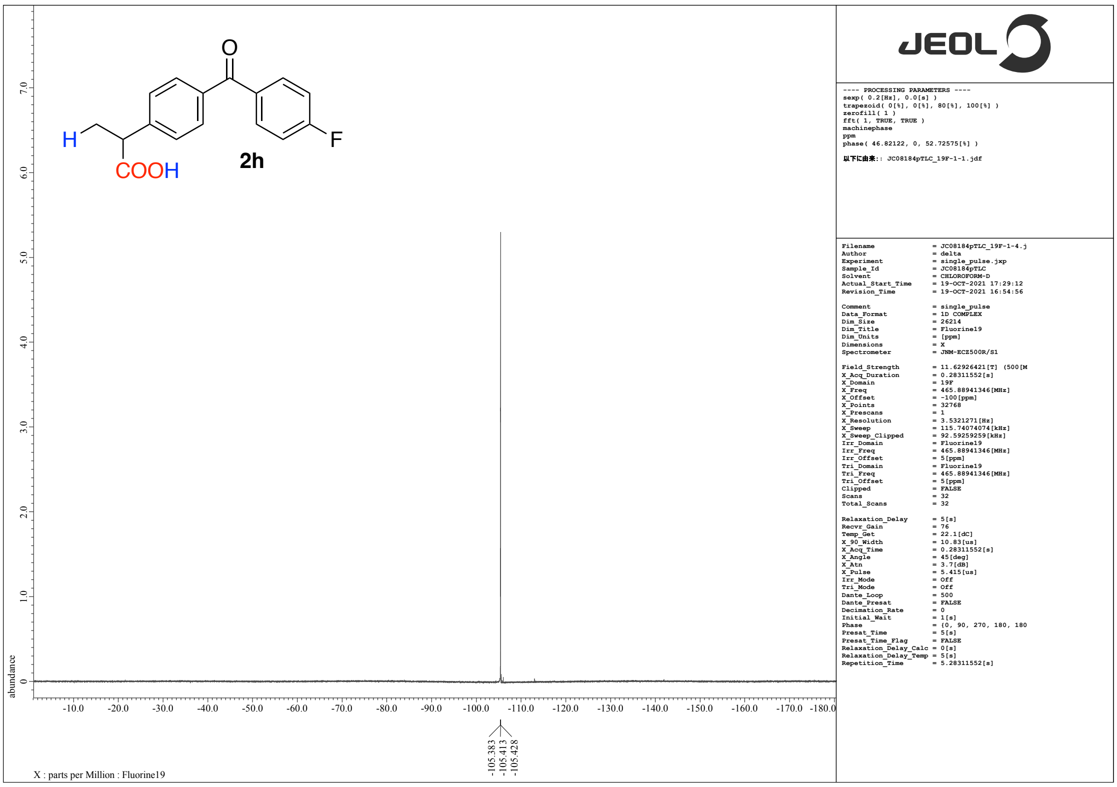

Supplementary Figure 36. ^{19}F NMR spectrum of **2h** (466 MHz, r.t., $CDCl_3$)

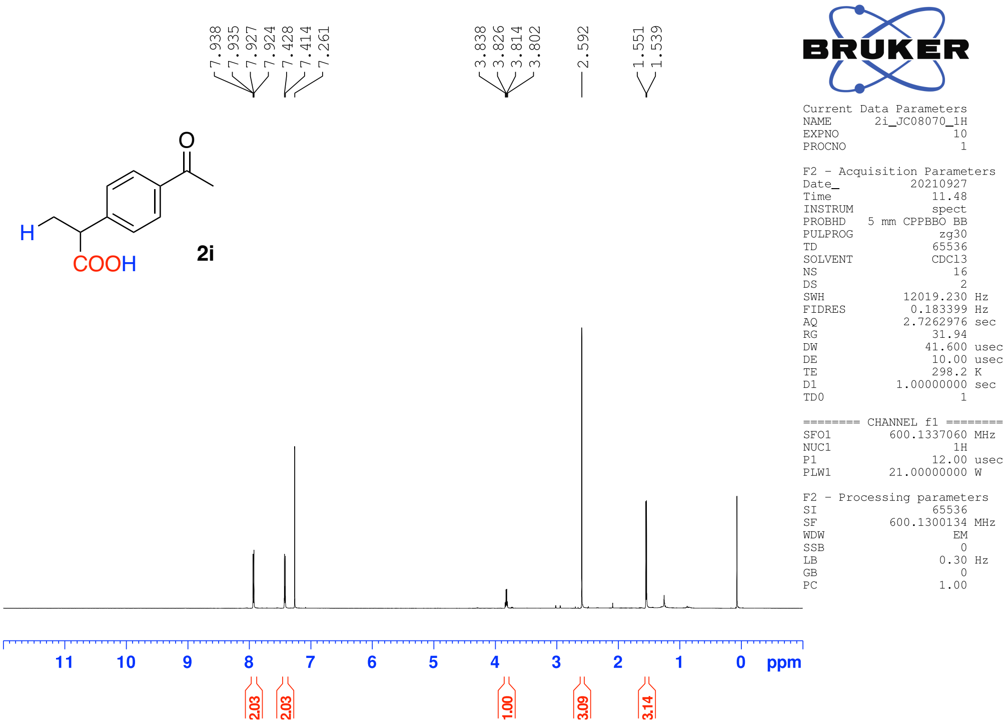

Supplementary Figure 37. ^{1}H NMR spectrum of **2i** (600 MHz, r.t., $CDCl_3$)

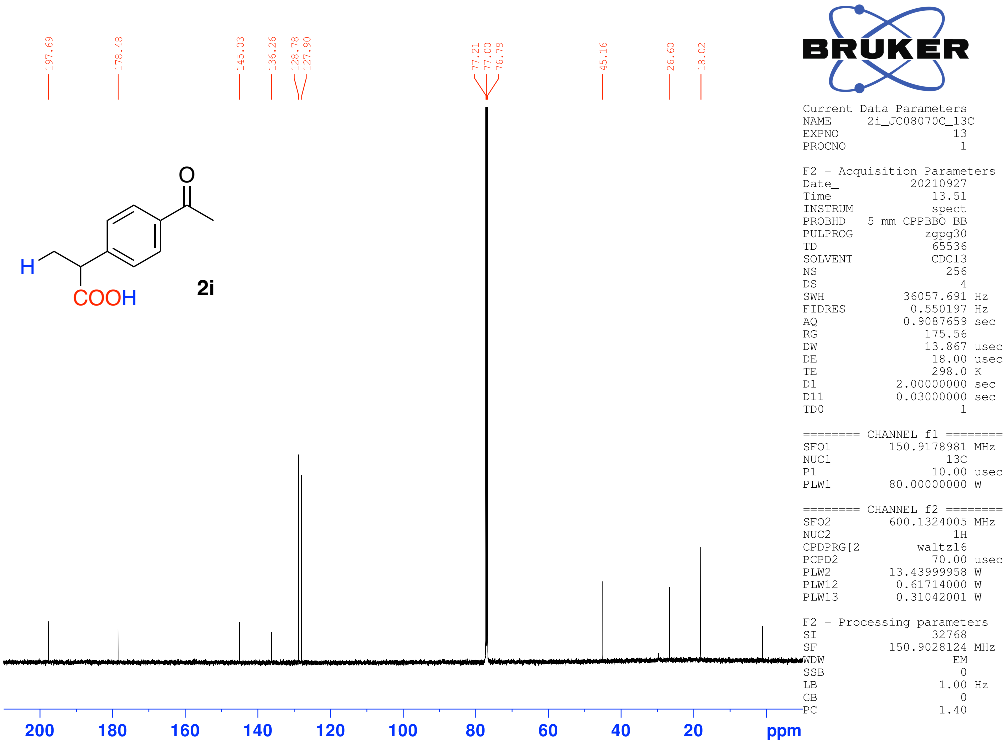

Supplementary Figure 38. ^{13}C NMR spectrum of **2i** (151 MHz, r.t., $CDCl_3$)

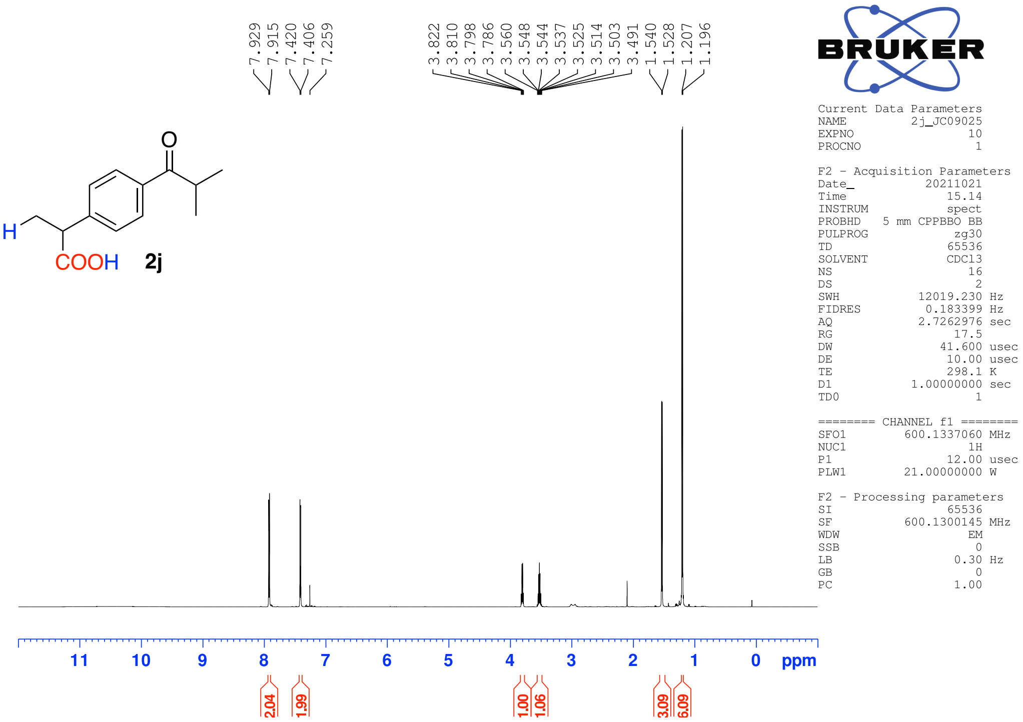

Supplementary Figure 39. ^{1}H NMR spectrum of **2j** (600 MHz, r.t., $CDCl_3$)

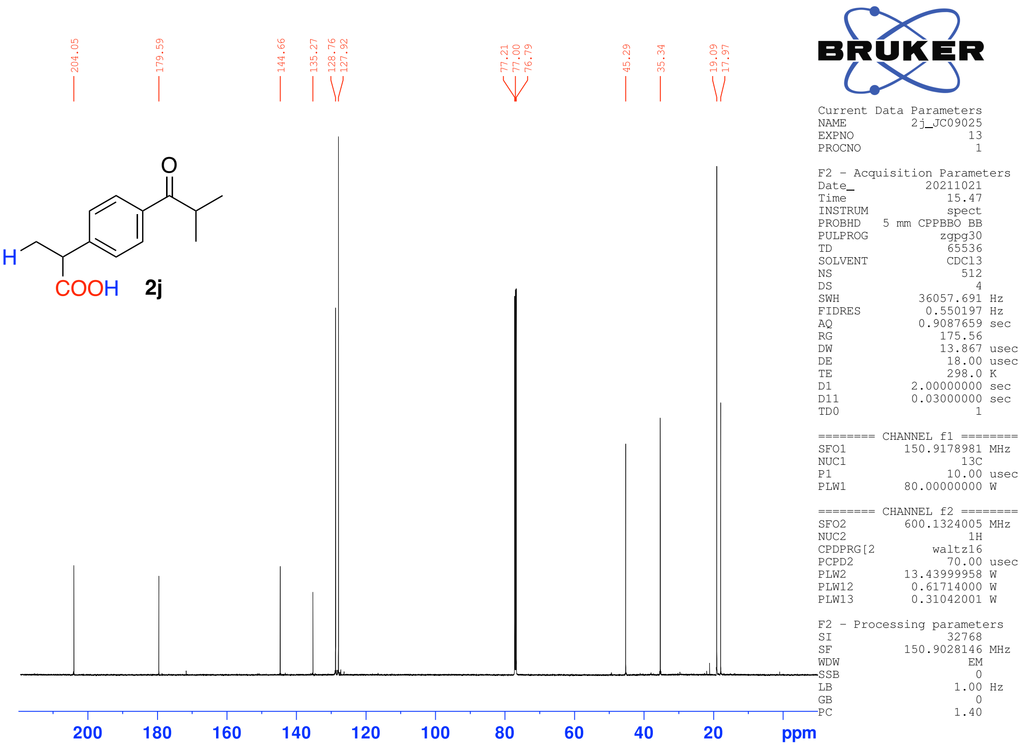

Supplementary Figure 40. ^{13}C NMR spectrum of **2j** (151 MHz, r.t., $CDCl_3$)

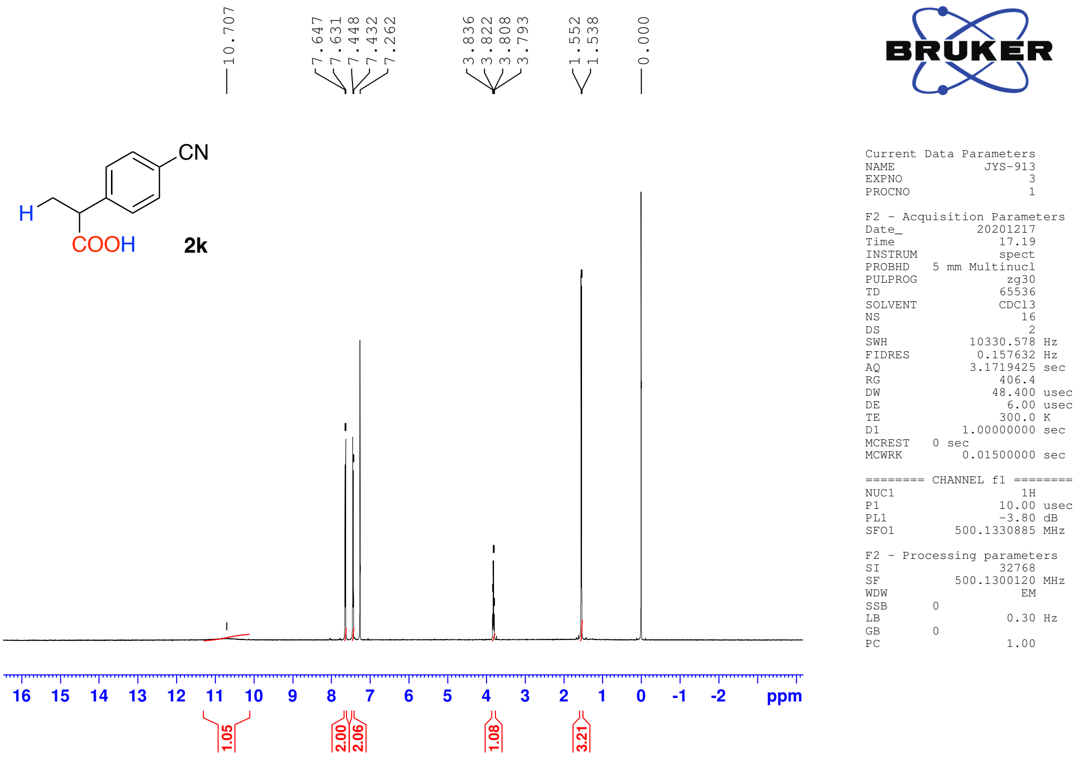

Supplementary Figure 41. ^{1}H NMR spectrum of **2k** (500 MHz, r.t., $CDCl_3$)

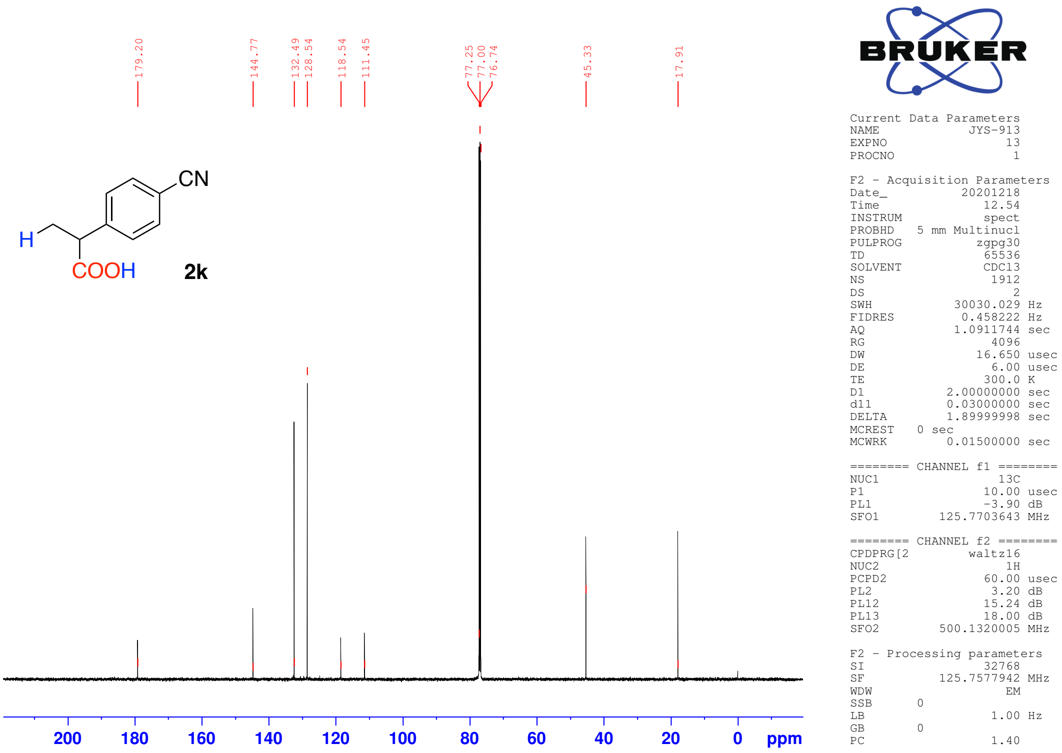

Supplementary Figure 42. ^{13}C NMR spectrum of **2k** (126 MHz, r.t., $CDCl_3$)

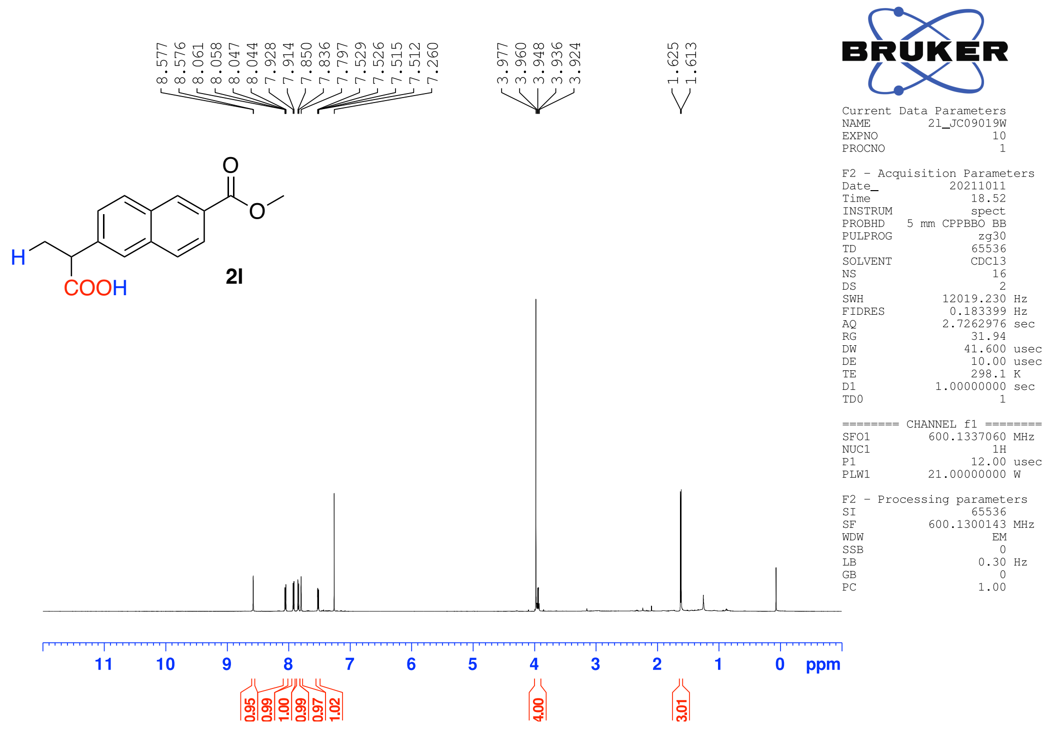

Supplementary Figure 43. ^{1}H NMR spectrum of **2l** (600 MHz, r.t., $CDCl_3$)

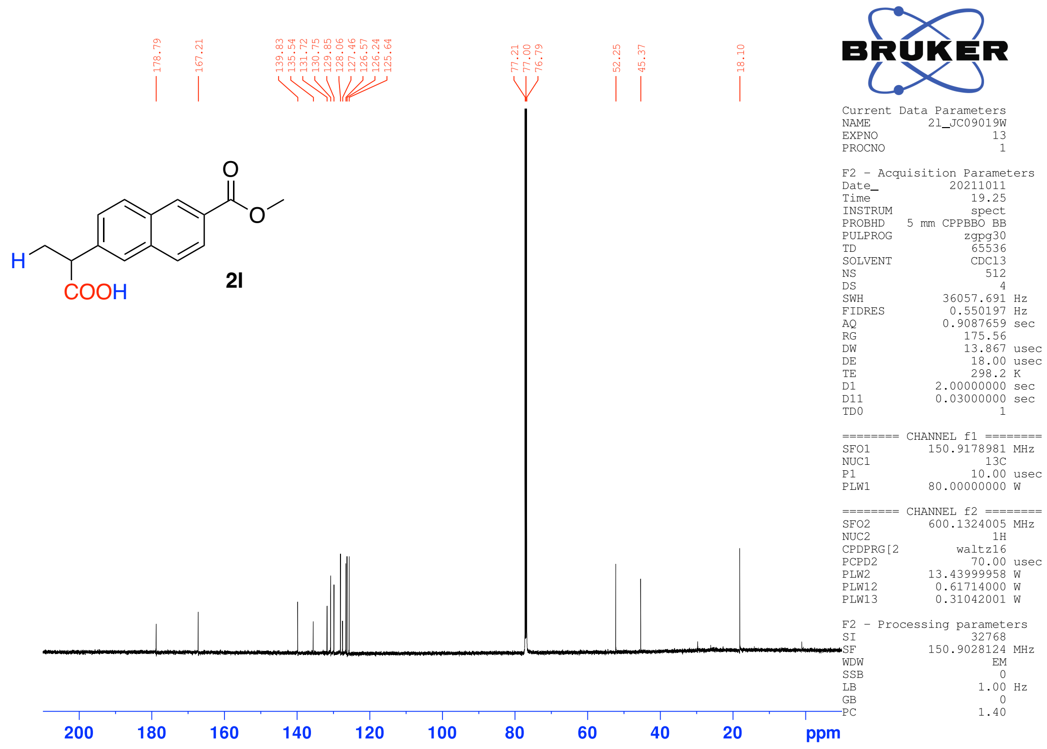

Supplementary Figure 44. ^{13}C NMR spectrum of **2l** (151 MHz, r.t., $CDCl_3$)

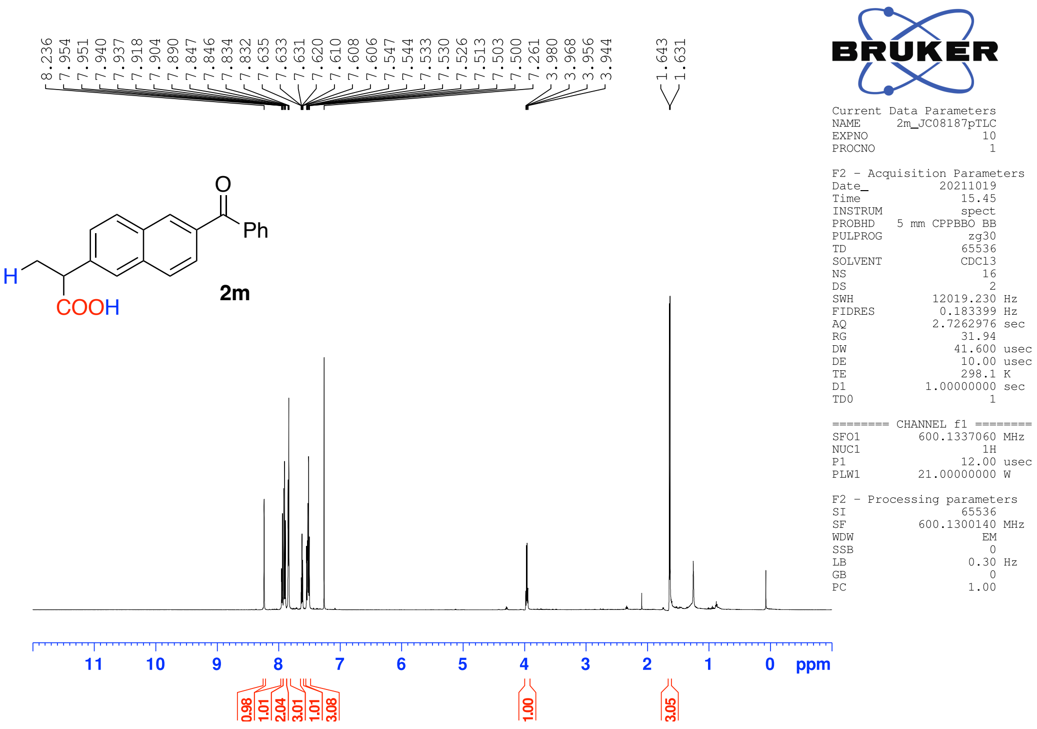

Supplementary Figure 45. ^{1}H NMR spectrum of **2m** (600 MHz, r.t., $CDCl_3$)

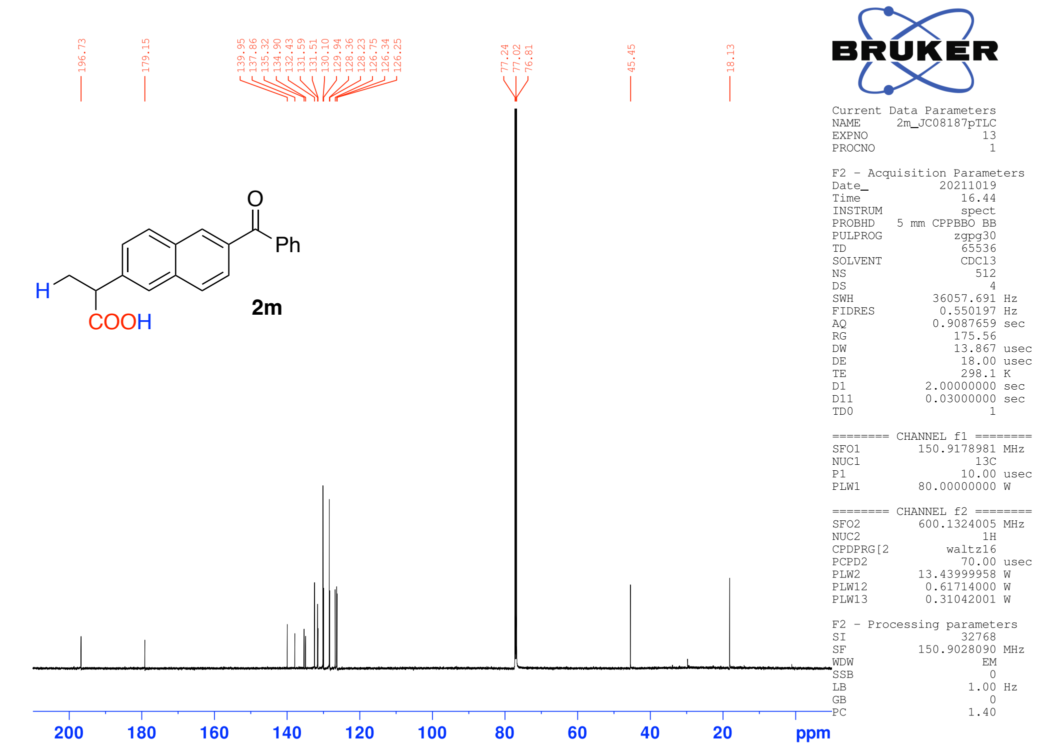

Supplementary Figure 46. ^{13}C NMR spectrum of **2m** (151 MHz, r.t., $CDCl_3$)

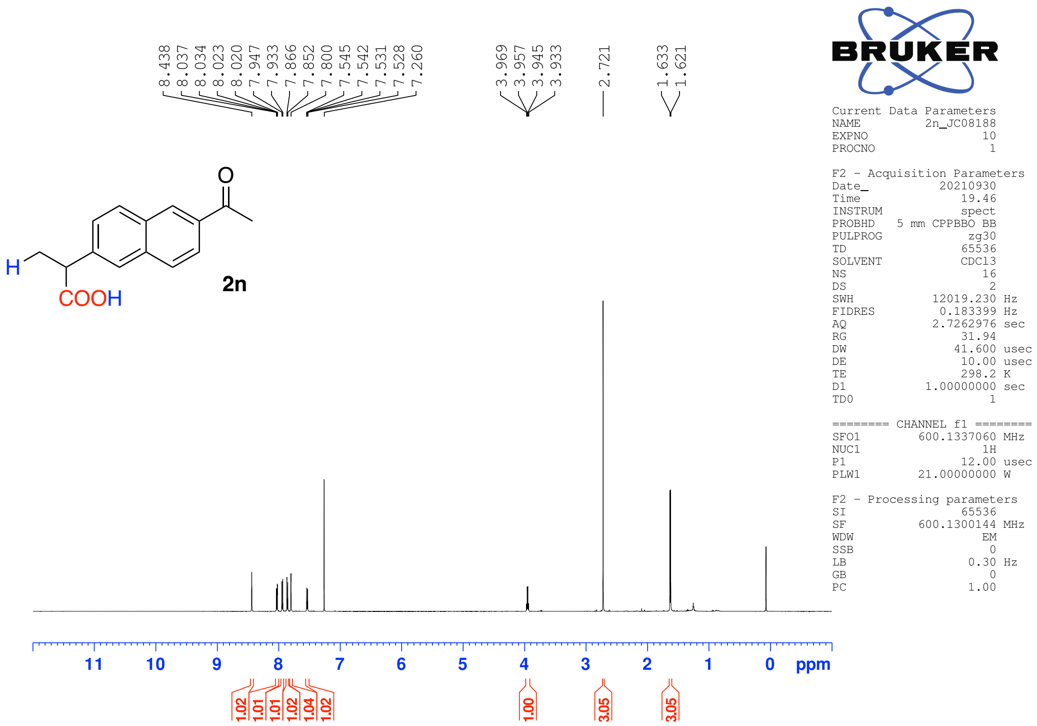

Supplementary Figure 47. ^{1}H NMR spectrum of **2n** (600 MHz, r.t., $CDCl_3$)

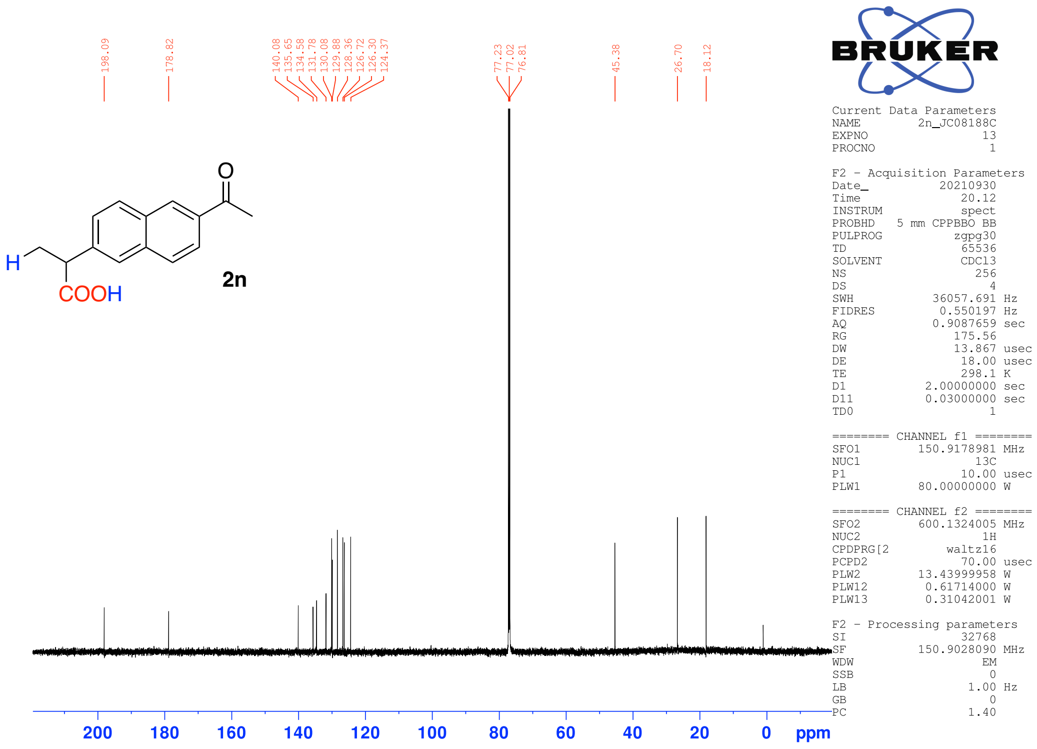

Supplementary Figure 48. ^{13}C NMR spectrum of **2n** (151 MHz, r.t., $CDCl_3$)

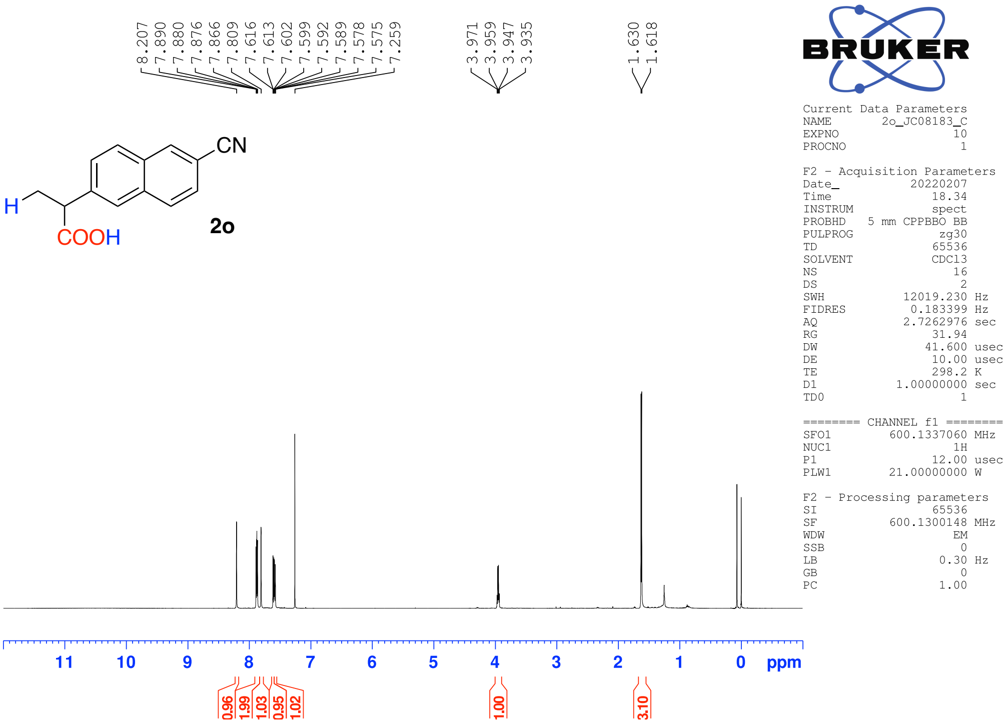

Supplementary Figure 49. ^{1}H NMR spectrum of **2o** (600 MHz, r.t., $CDCl_3$)

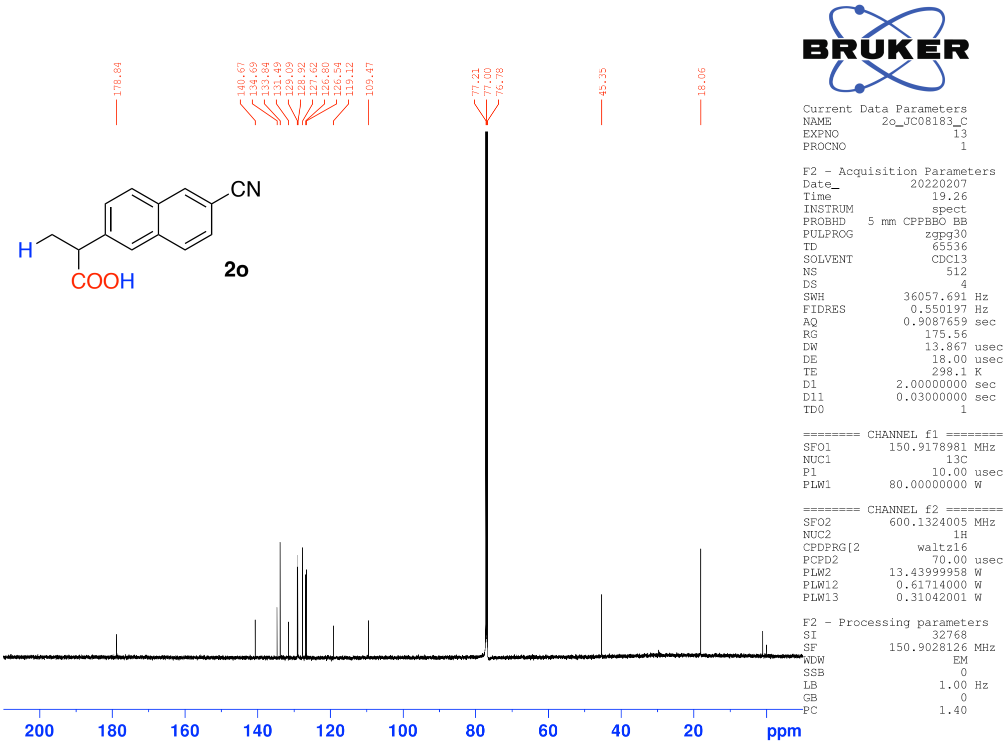

Supplementary Figure 50. ^{13}C NMR spectrum of **2o** (151 MHz, r.t., $CDCl_3$)

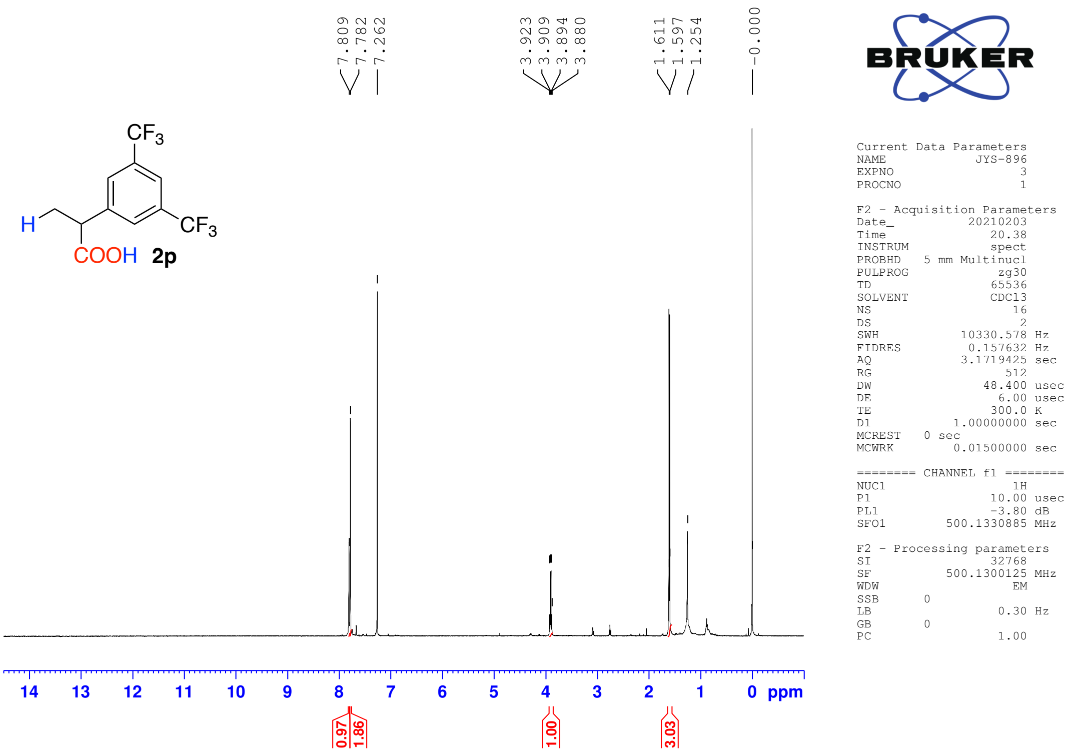

Supplementary Figure 51. ^{1}H NMR spectrum of **2p** (500 MHz, r.t., $CDCl_3$)

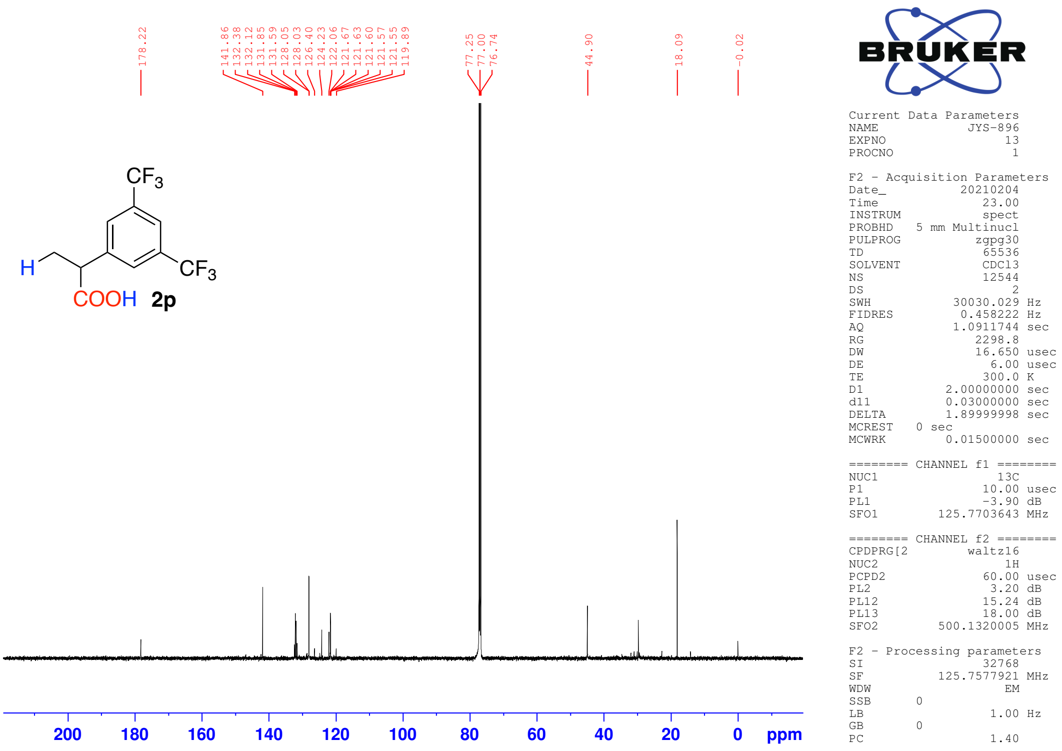

Supplementary Figure 52. ^{13}C NMR spectrum of **2p** (126 MHz, r.t., $CDCl_3$)

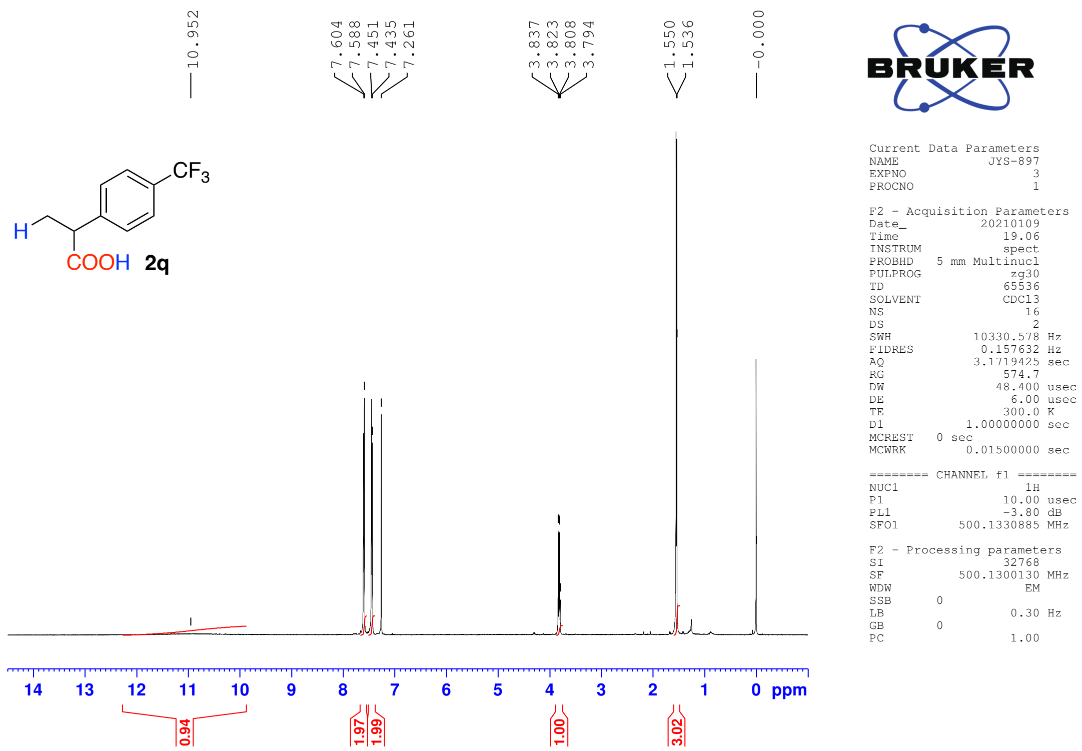

Supplementary Figure 53. ^{1}H NMR spectrum of **2q** (500 MHz, r.t., $CDCl_3$)

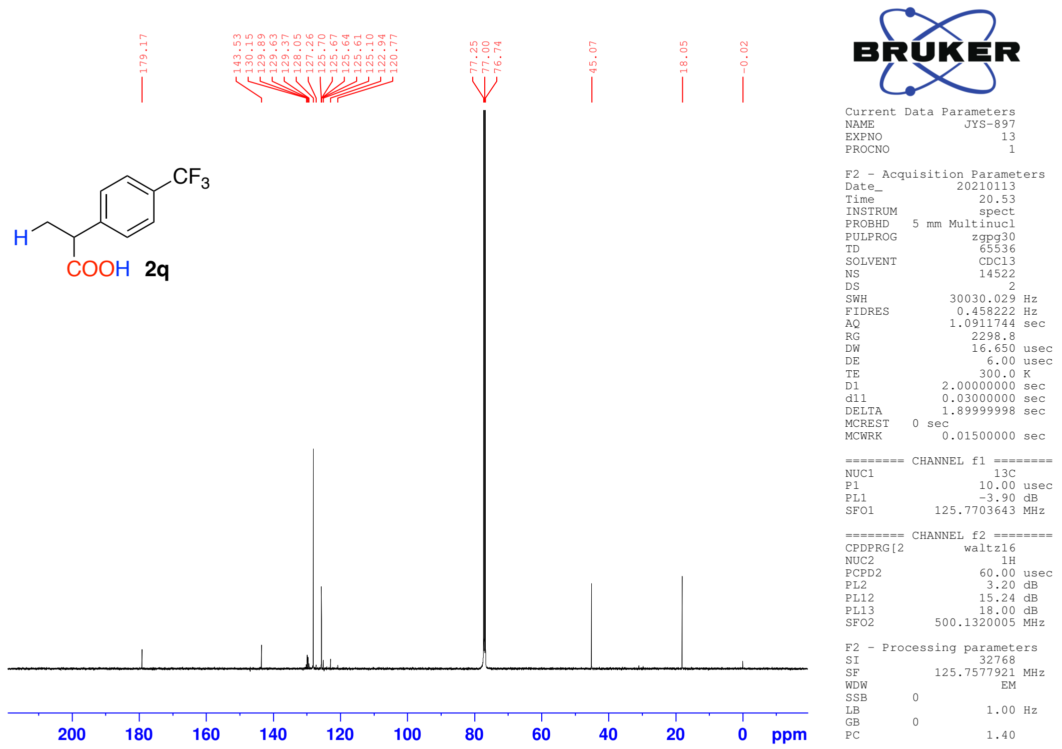

Supplementary Figure 54. ^{13}C NMR spectrum of **2q** (126 MHz, r.t., $CDCl_3$)

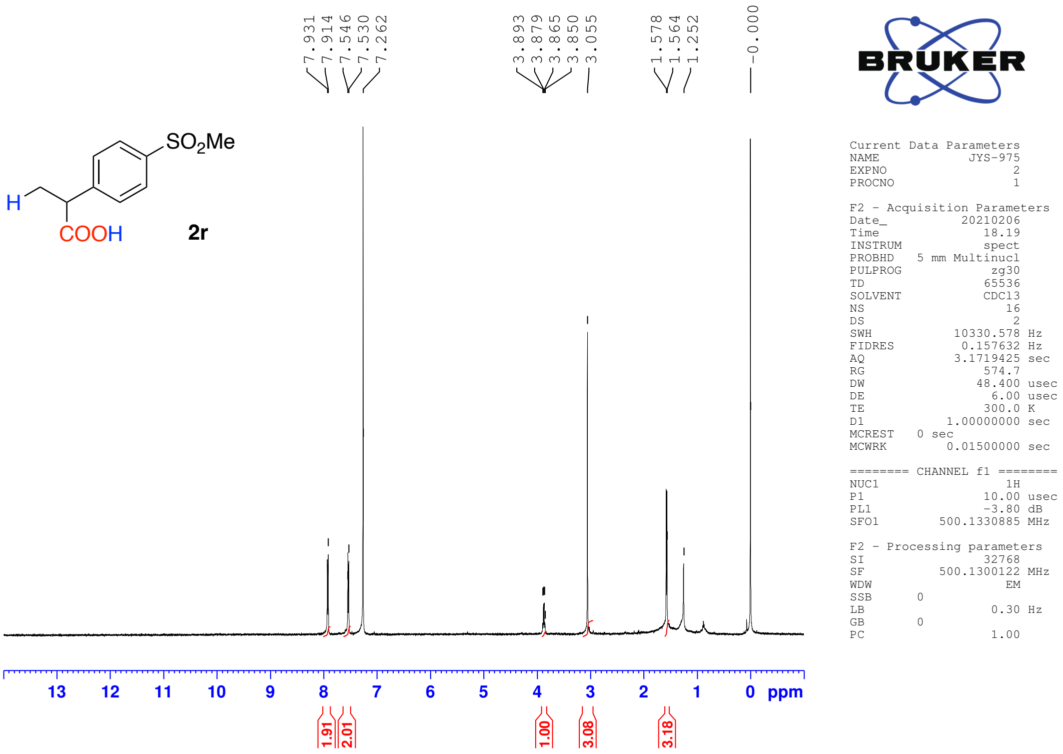

Supplementary Figure 55. ^{1}H NMR spectrum of **2r** (500 MHz, r.t., $CDCl_3$)

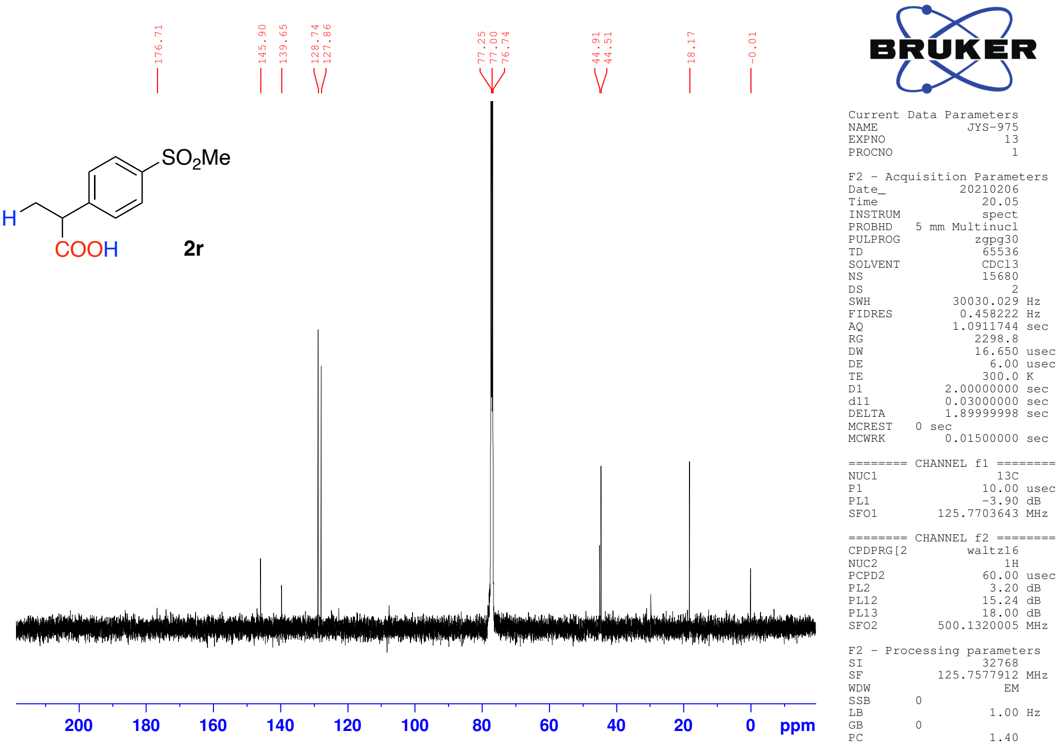

Supplementary Figure 56. ^{13}C NMR spectrum of **2r** (126 MHz, r.t., $CDCl_3$)

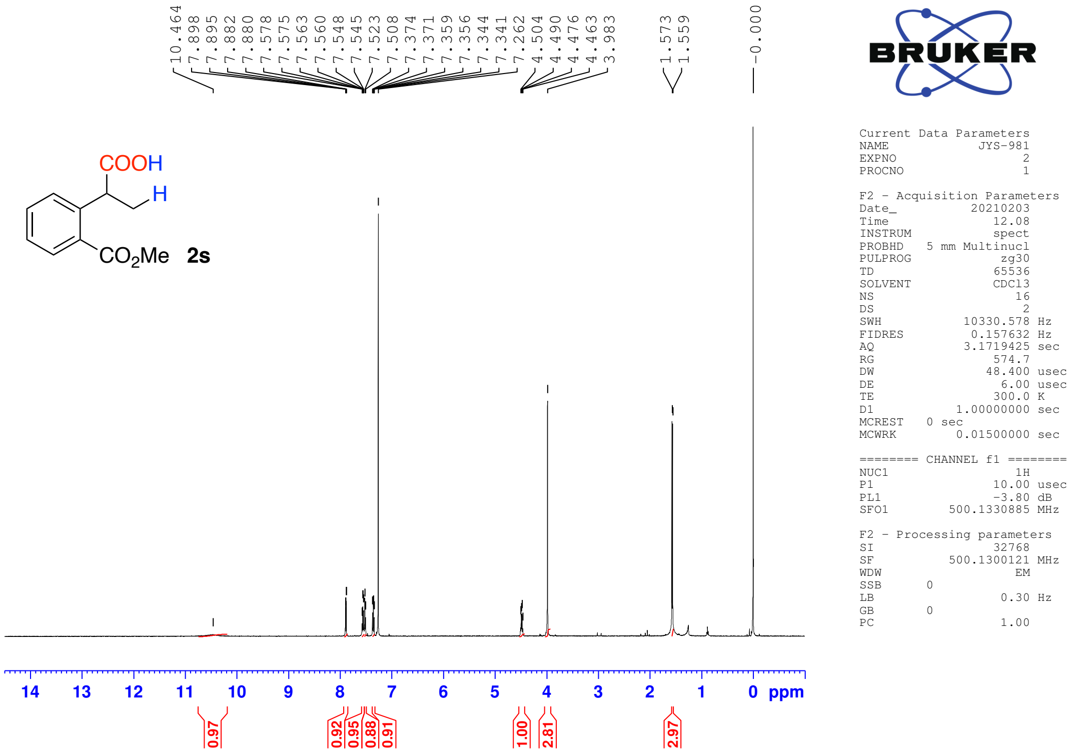

Supplementary Figure 57. ^{1}H NMR spectrum of **2s** (500 MHz, r.t., $CDCl_3$)

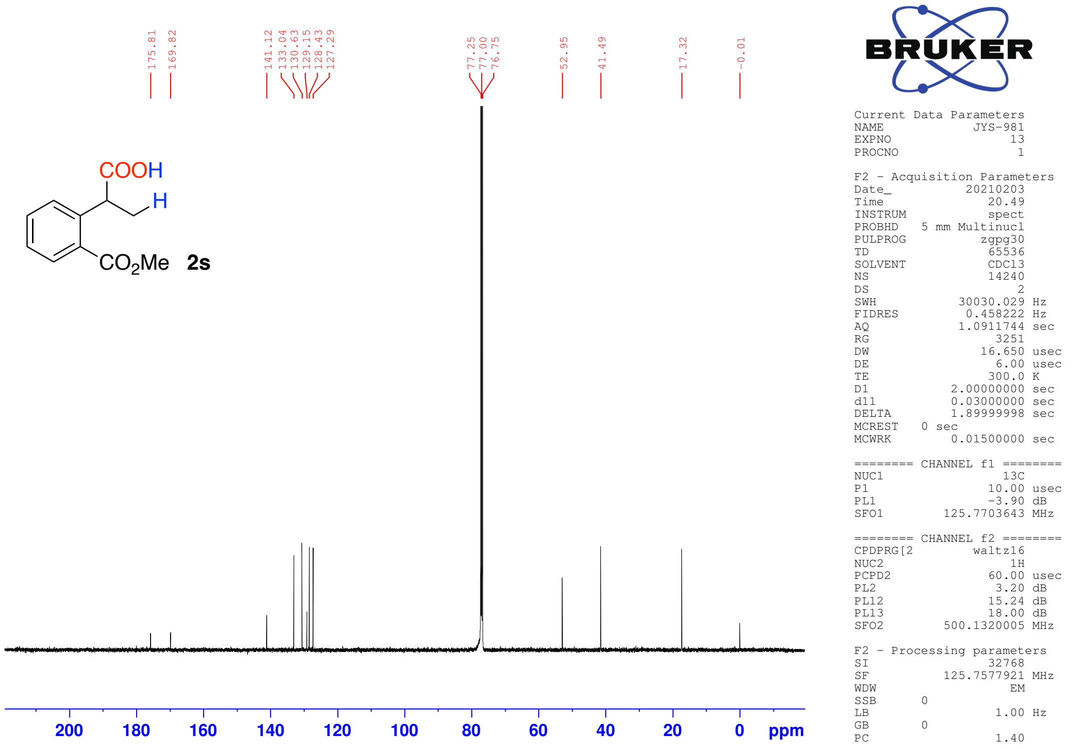

Supplementary Figure 58. ^{13}C NMR spectrum of **2s** (125 MHz, r.t., $CDCl_3$)

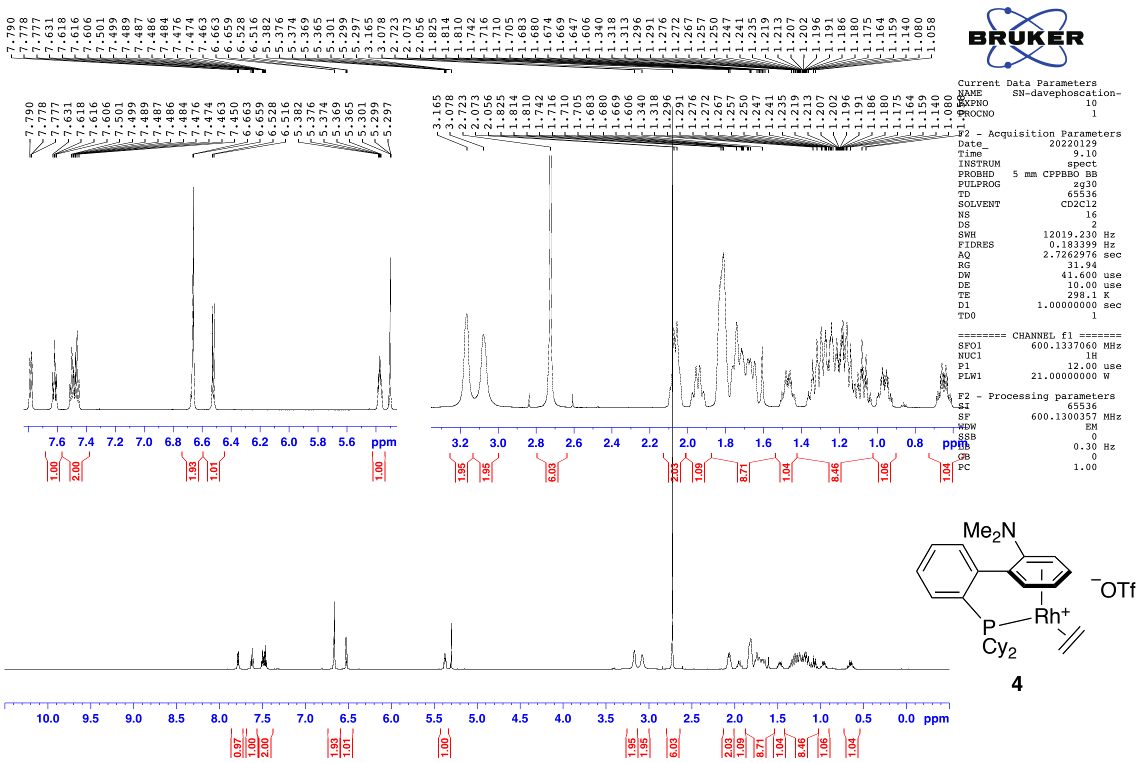

Supplementary Figure 59. ^{1}H NMR spectrum of **4** (600 MHz, r.t., CD_2Cl_2)

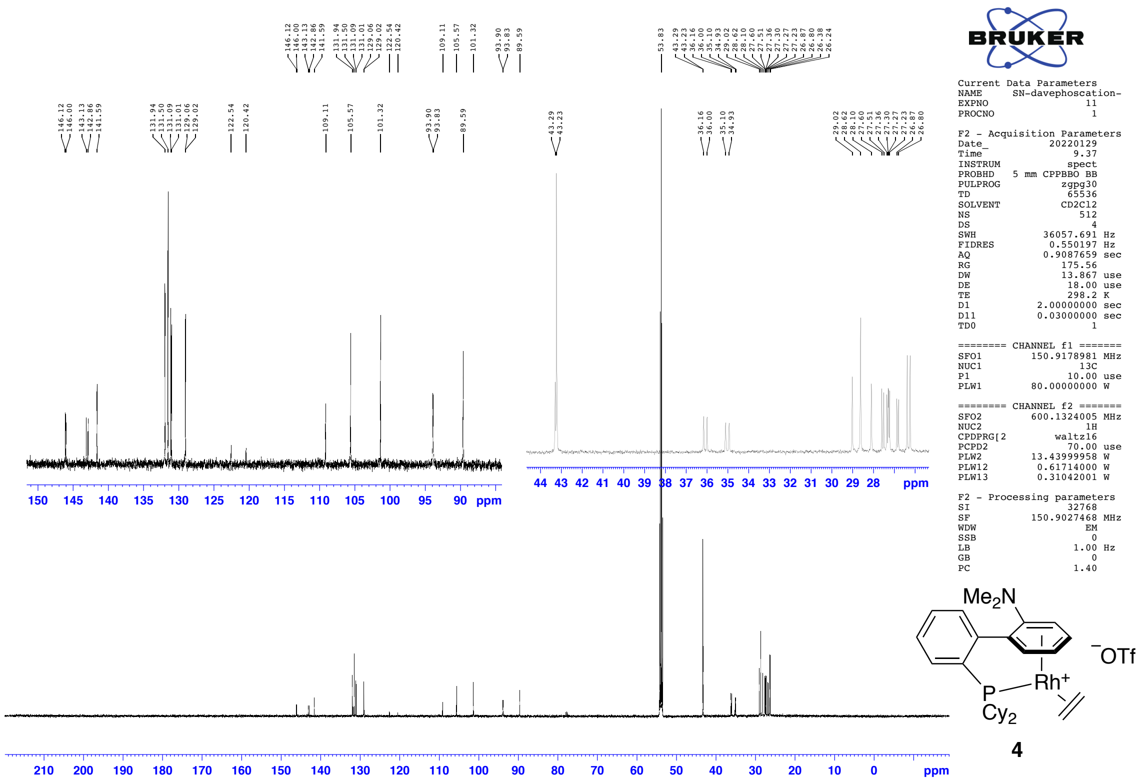

Supplementary Figure 60. ^{13}C NMR spectrum of **4** (151 MHz, r.t., CD_2Cl_2)

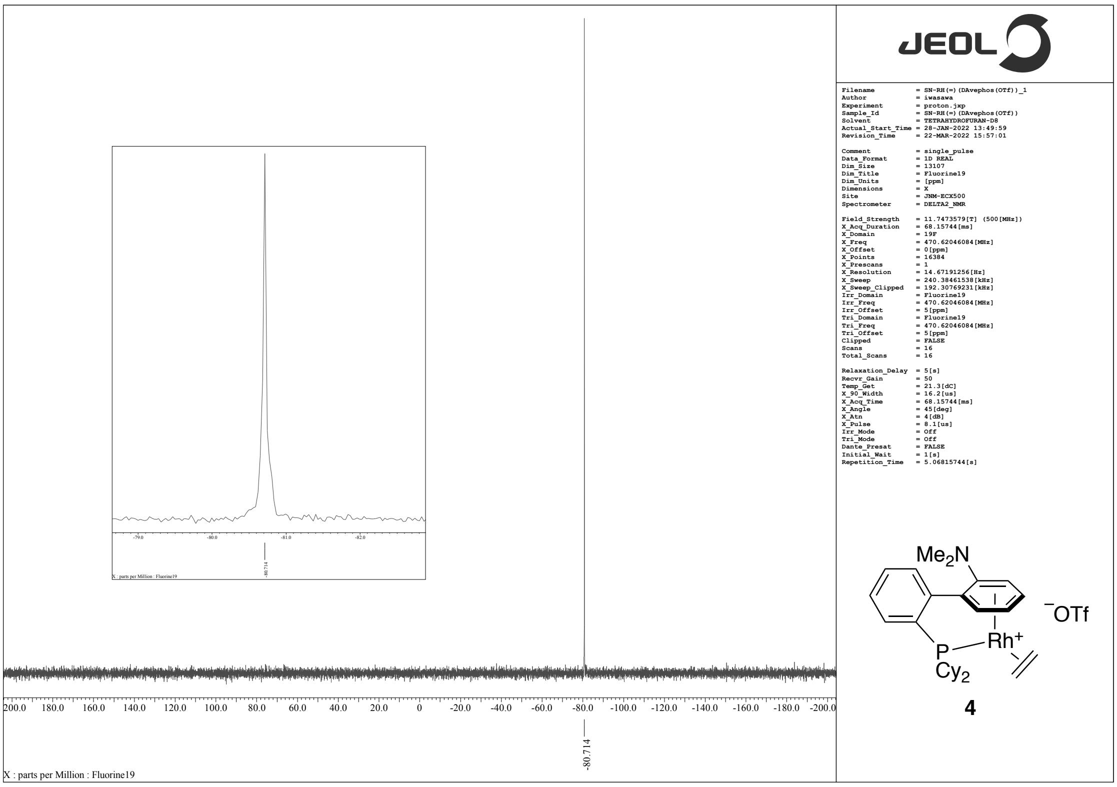

Supplementary Figure 61. ^{19}F NMR spectrum of **4** (471 MHz, r.t., THF-d_8)

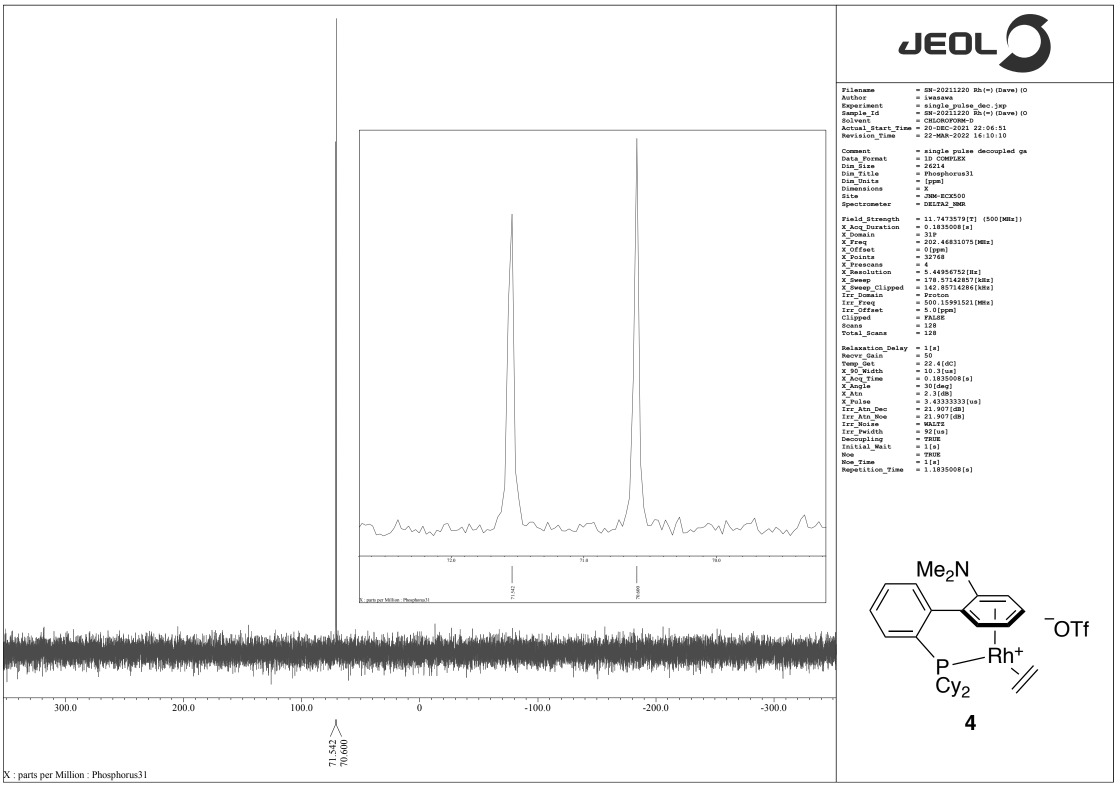

Supplementary Figure 62. ^{31}P NMR spectrum of **4** (202 MHz, r.t., $CDCl_3$)

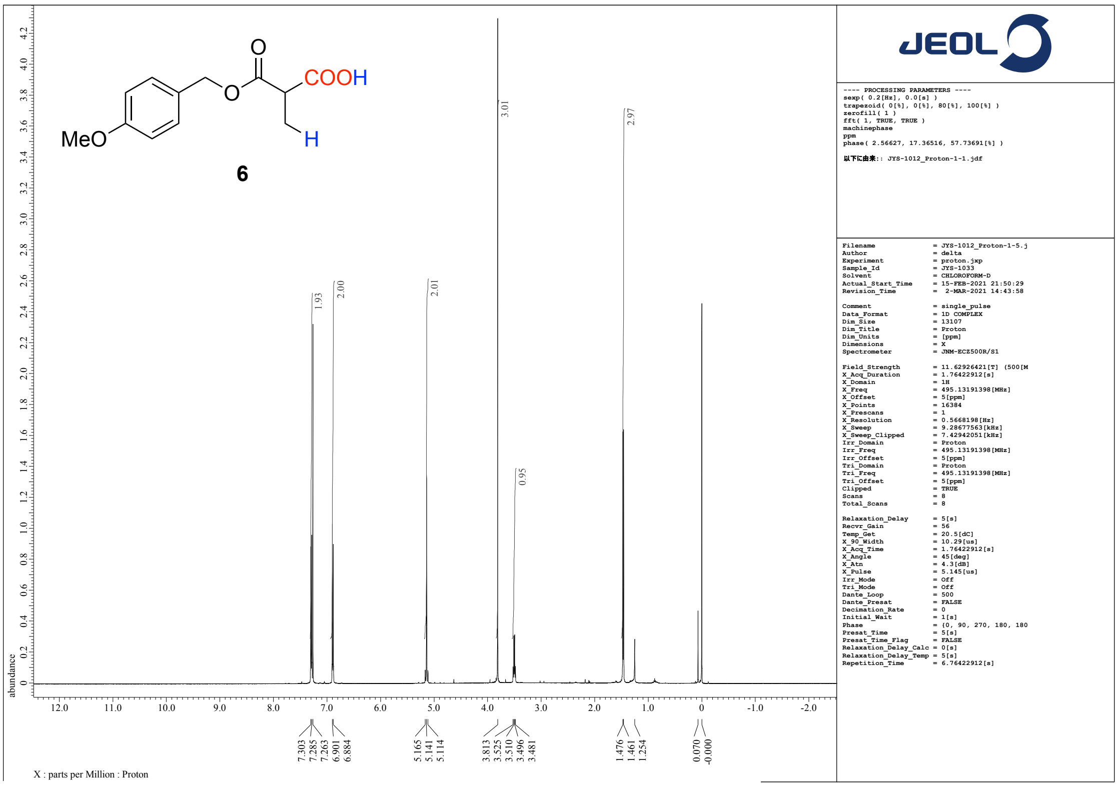

Supplementary Figure 63. ^{1}H NMR spectrum of **6** (495 MHz, r.t., $CDCl_3$)

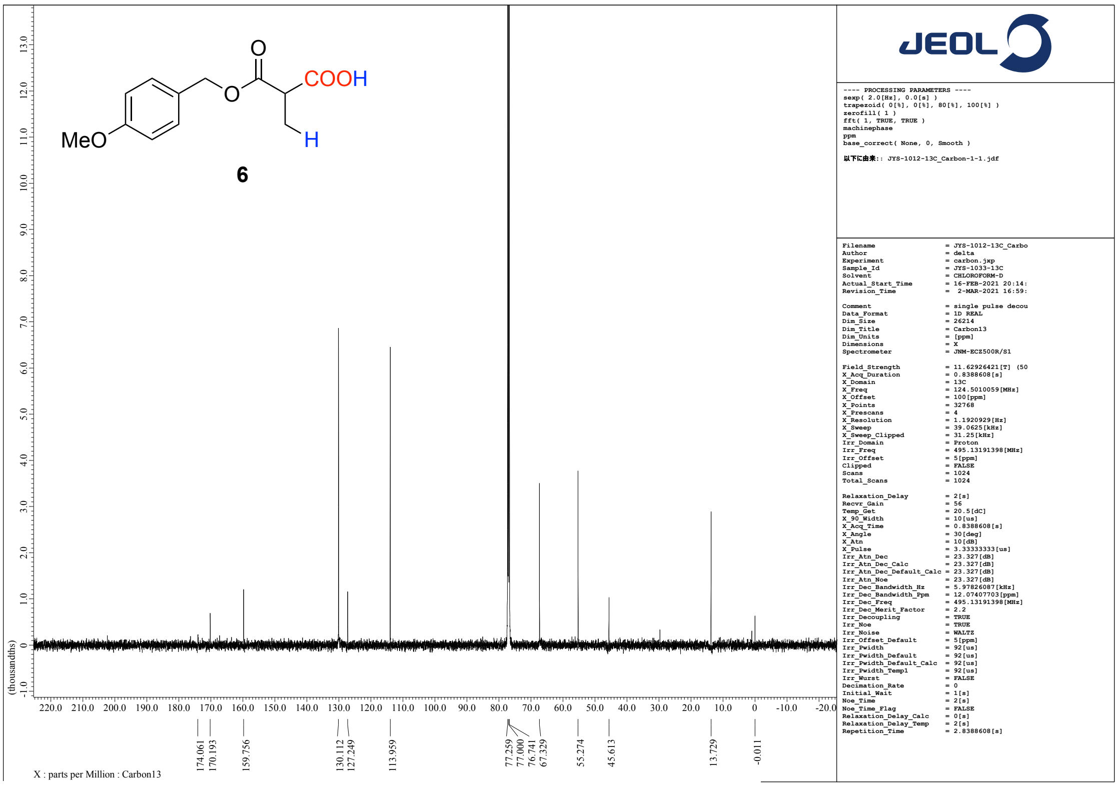

Supplementary Figure 64. ^{13}C NMR spectrum of **6** (125 MHz, r.t., $CDCl_3$)

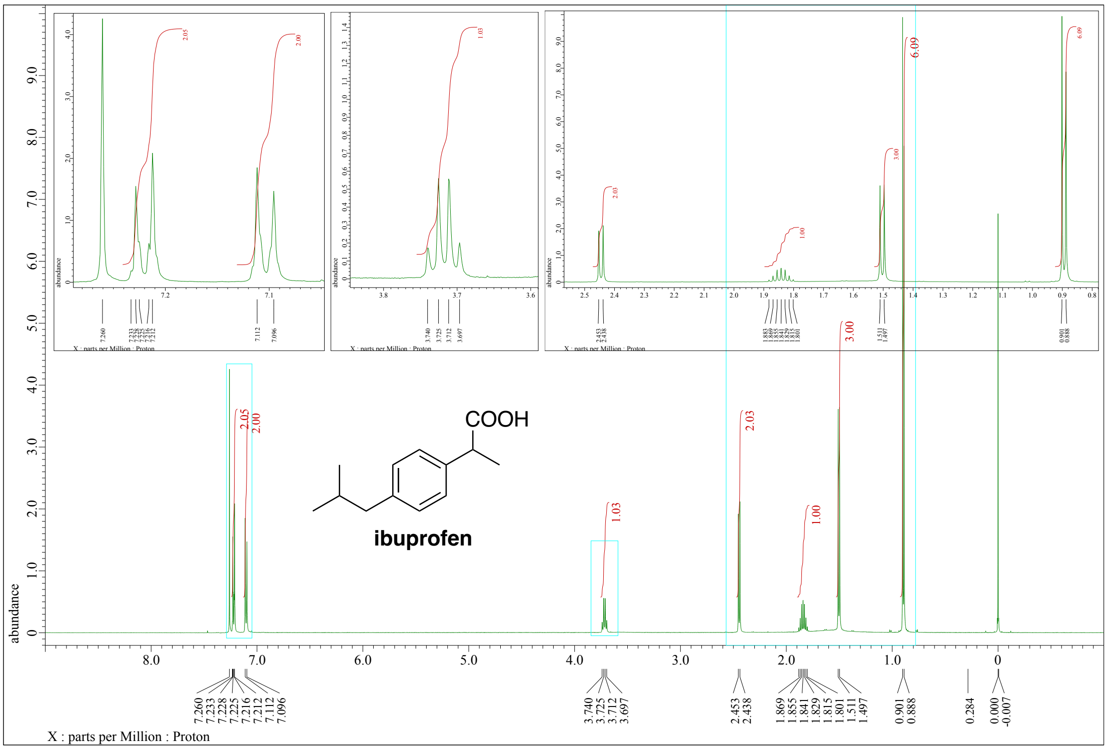

Supplementary Figure 65. ^{1}H NMR spectrum of **ibuprofen** (500 MHz, r.t., $CDCl_3$)

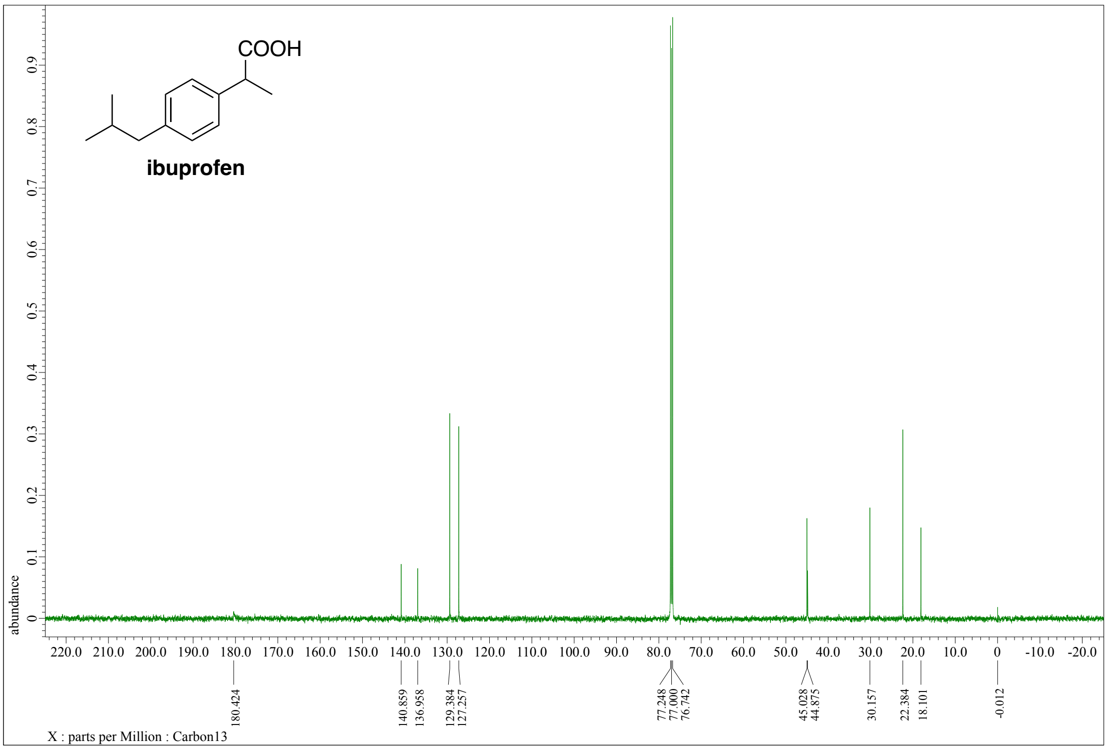

Supplementary Figure 66. ^{13}C NMR spectrum of **ibuprofen** (126 MHz, r.t., $CDCl_3$)

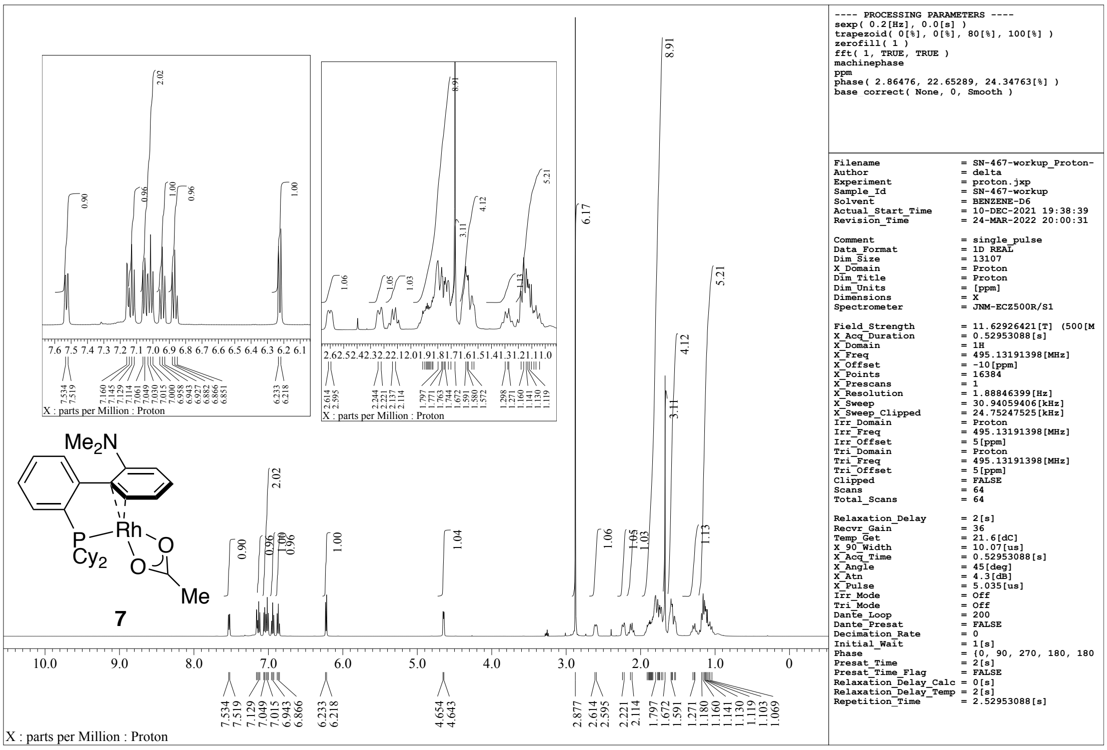

Supplementary Figure 67. ^{1}H NMR spectrum of **7** (495 MHz, r.t., C_6D_6)

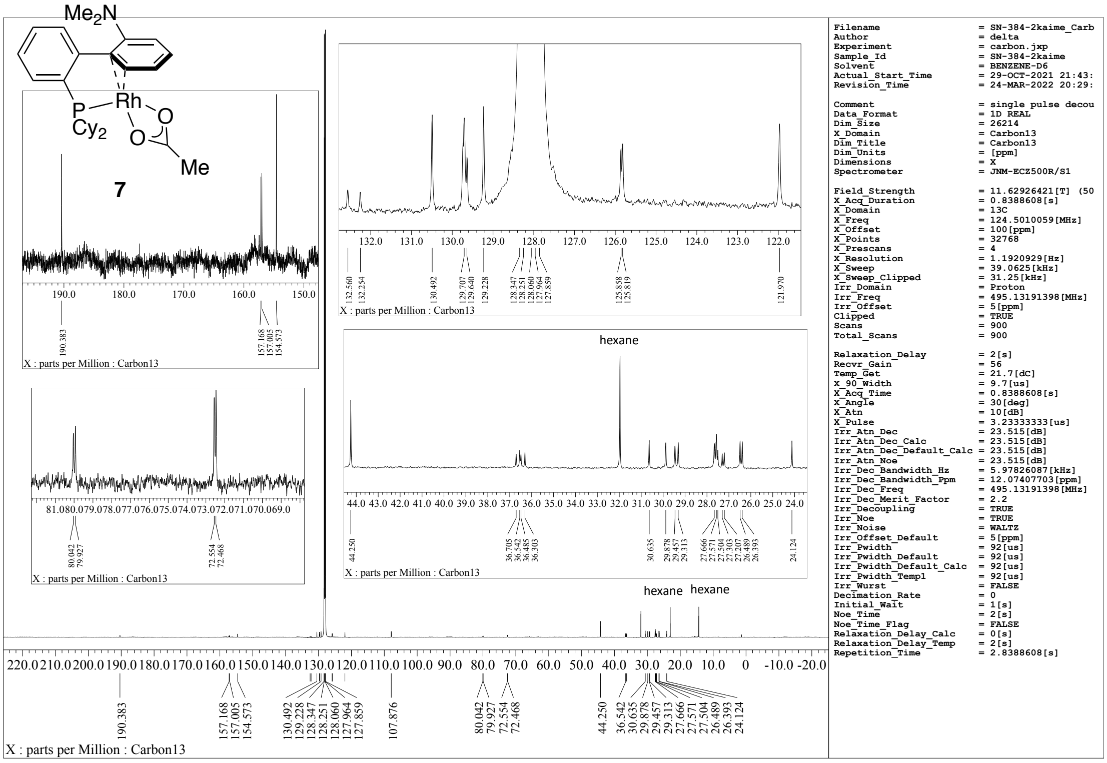

Supplementary Figure 68. ^{13}C NMR spectrum of **7** (125 MHz, r.t., C_6D_6)

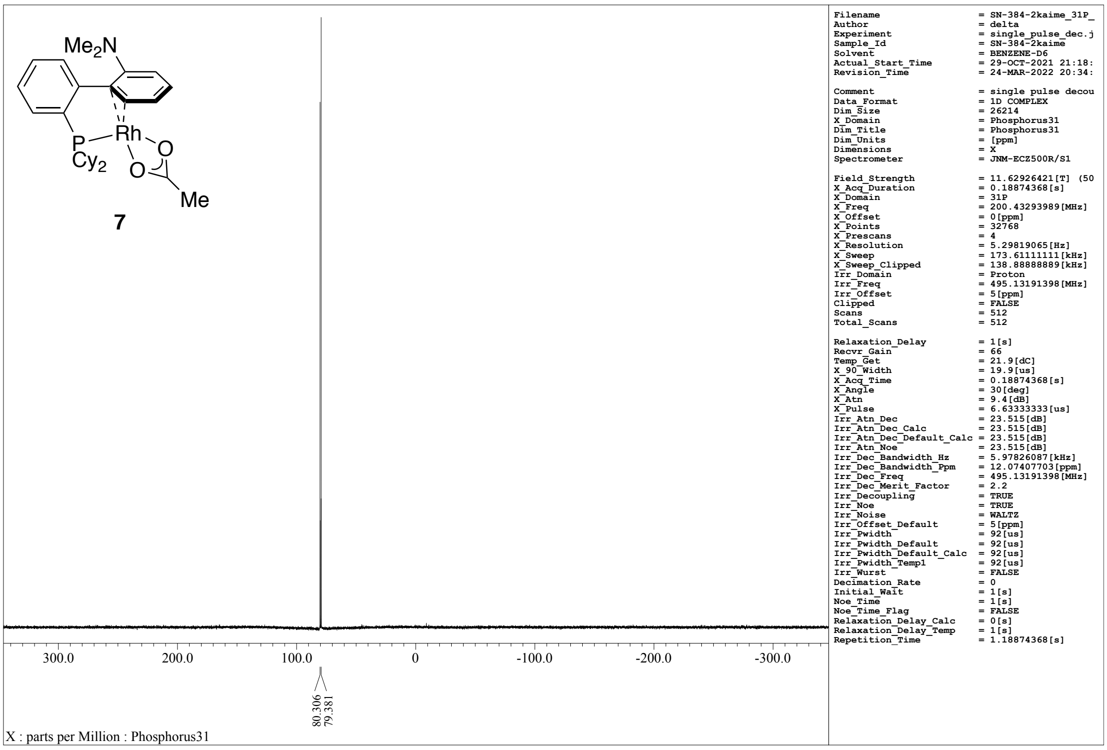

Supplementary Figure 69. ^{31}P NMR spectrum of **7** (200 MHz, r.t., C_6D_6)

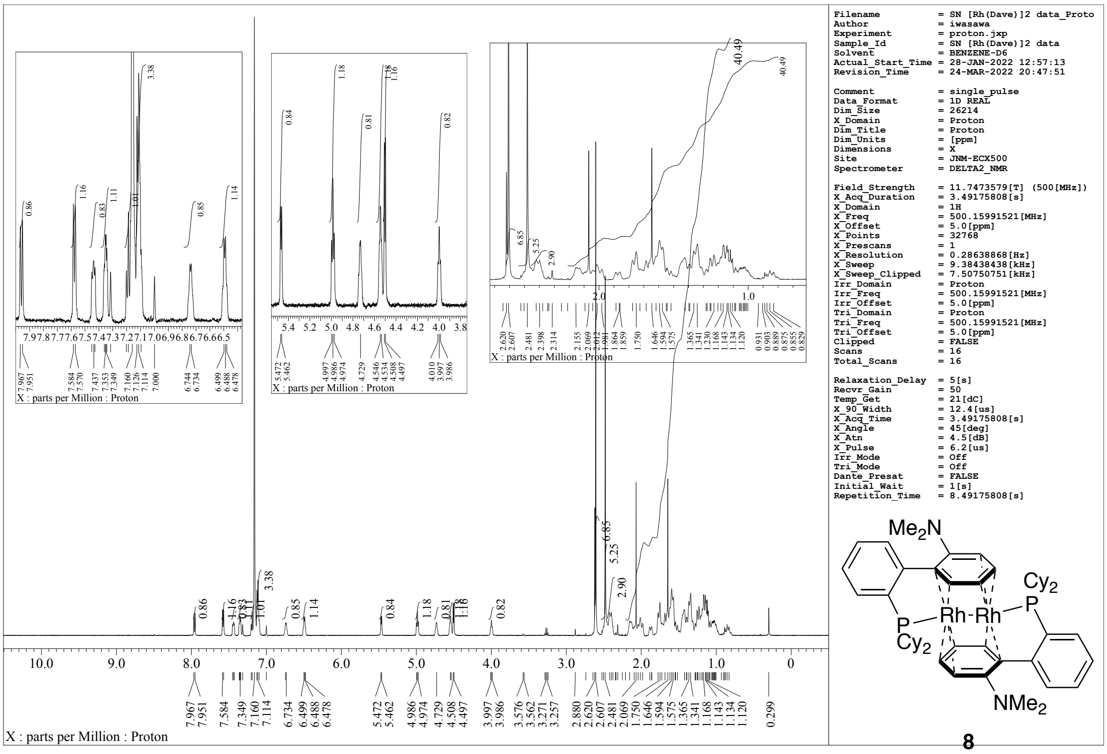

Supplementary Figure 70. ^{1}H NMR spectrum of **8** (500 MHz, r.t., C_6D_6)

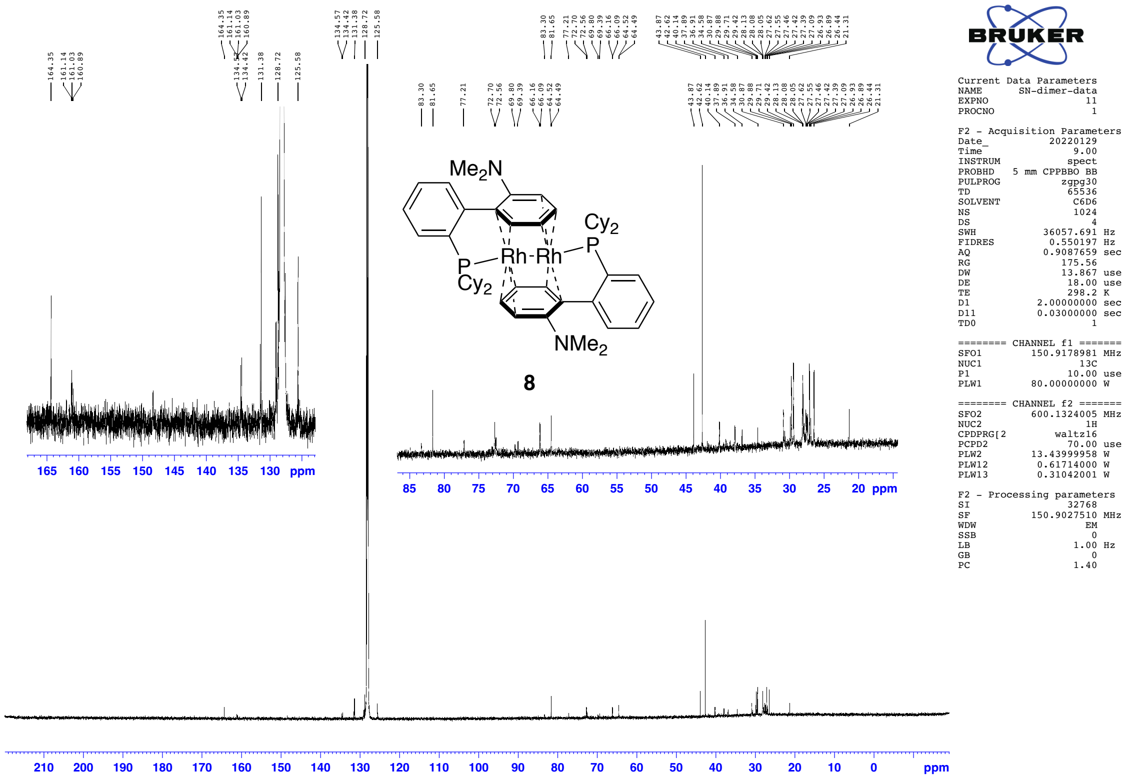

Supplementary Figure 71. ^{13}C NMR spectrum of **8** (151 MHz, r.t., C_6D_6)

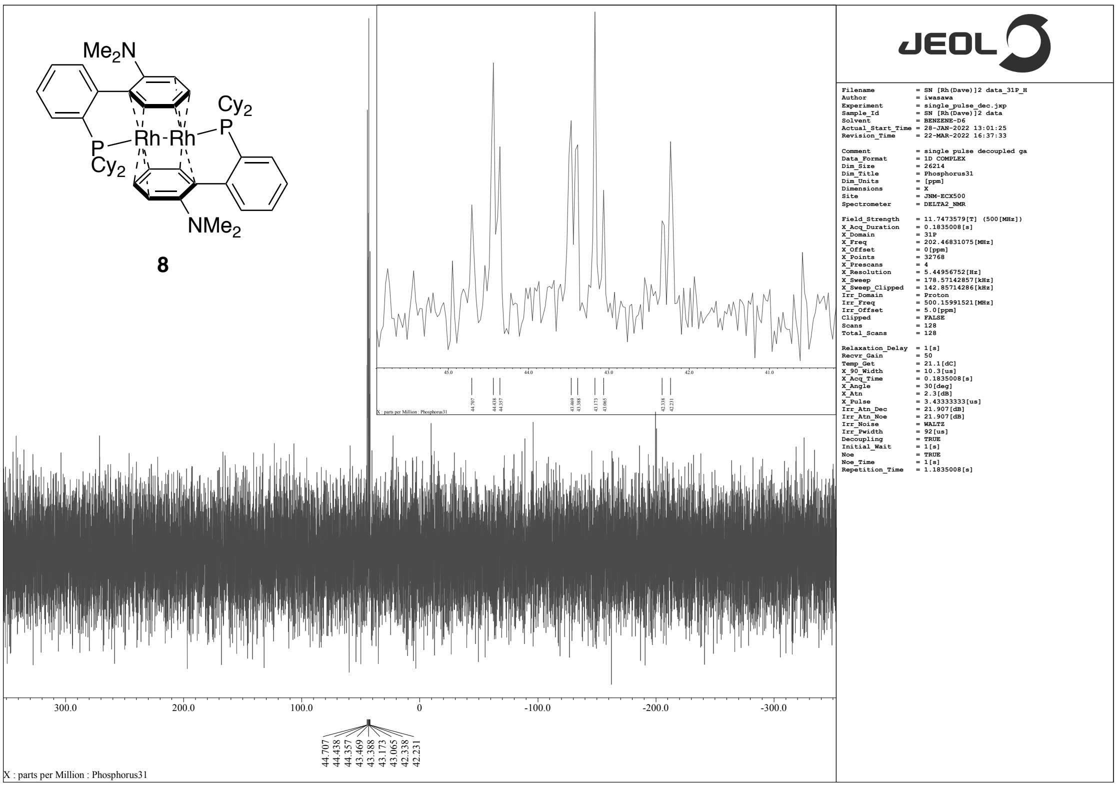

Supplementary Figure 72. ^{31}P NMR spectrum of **8** (202 MHz, r.t., C_6D_6)

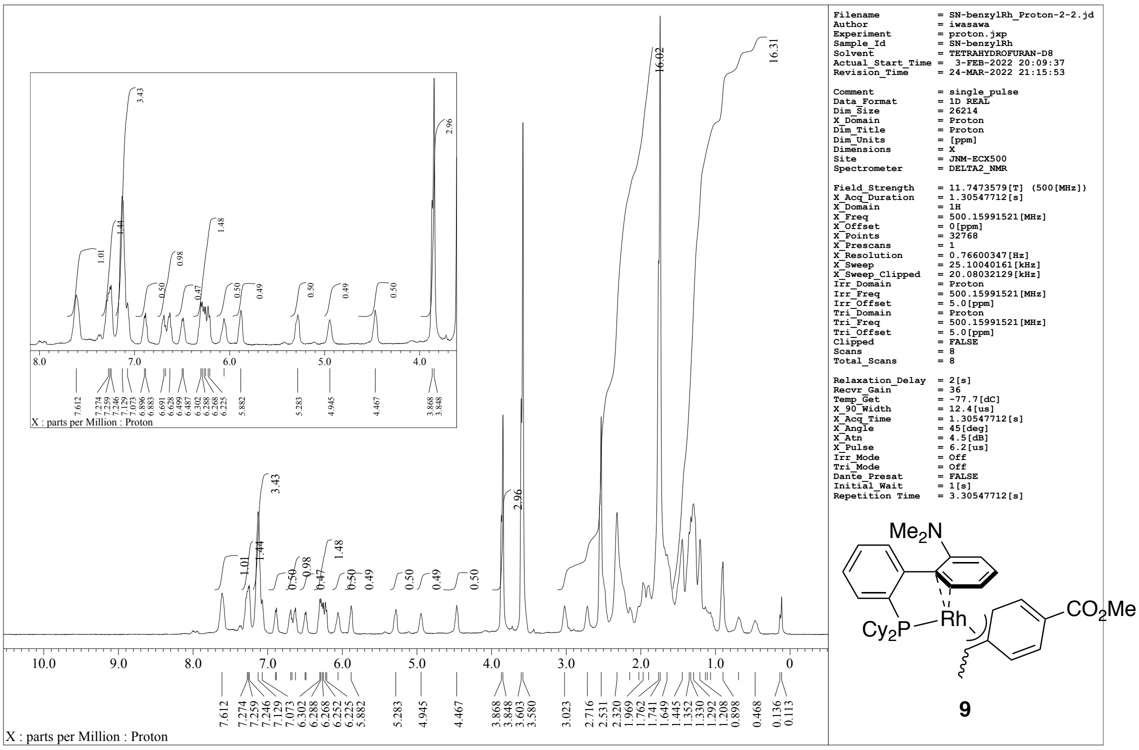

Supplementary Figure 73. ^{1}H NMR spectrum of **9** (500 MHz, –78 °C, THF-d_8)

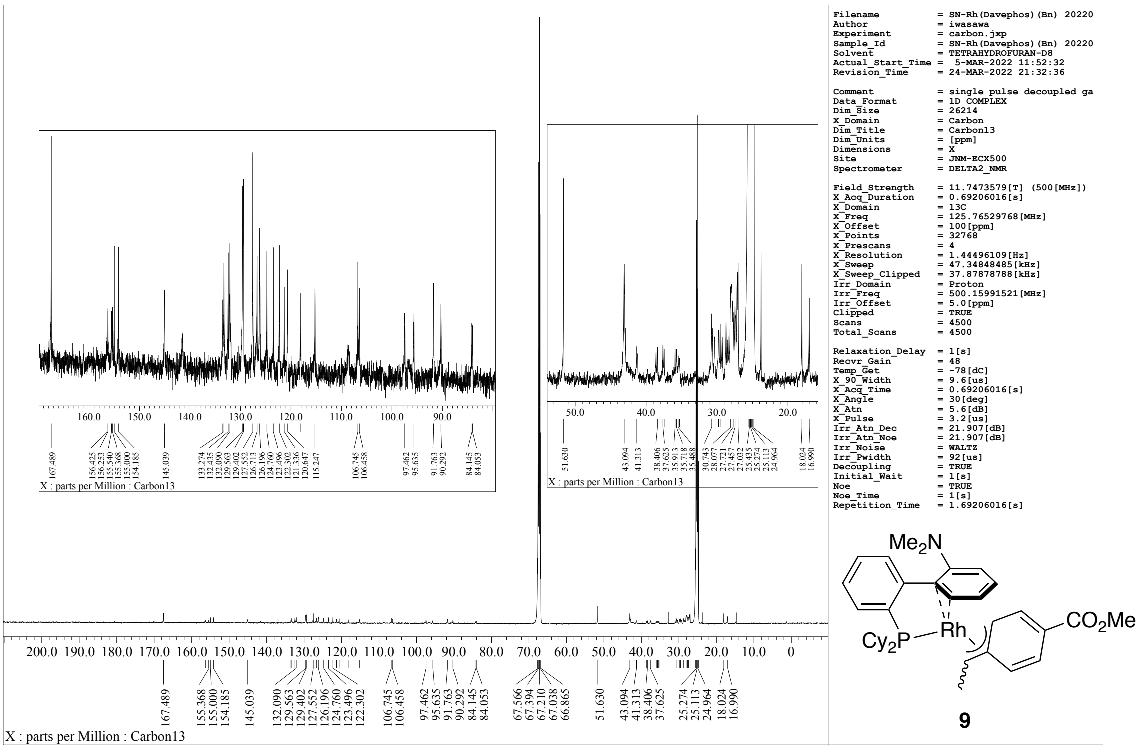

Supplementary Figure 74. ^{13}C NMR spectrum of **9** (126 MHz, –78 °C, THF-d_8)

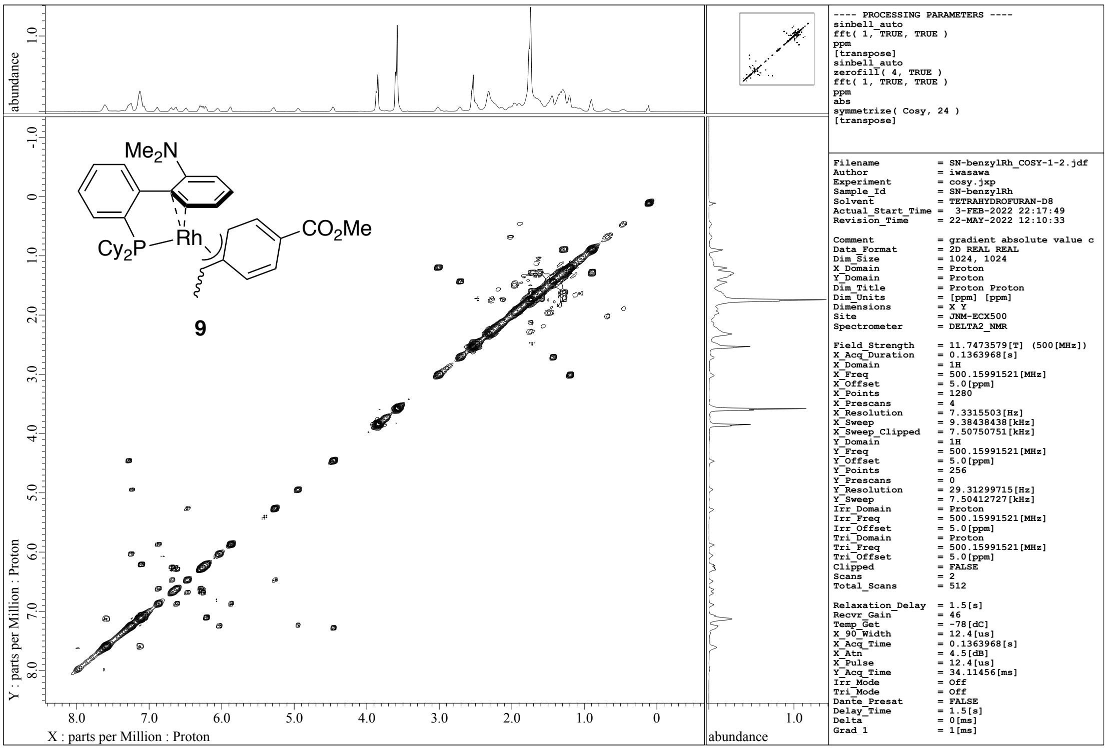

Supplementary Figure 75. ^{1}H-^{1}H COSY NMR spectrum of **9** (500 MHz, –78 °C, THF-d_8)

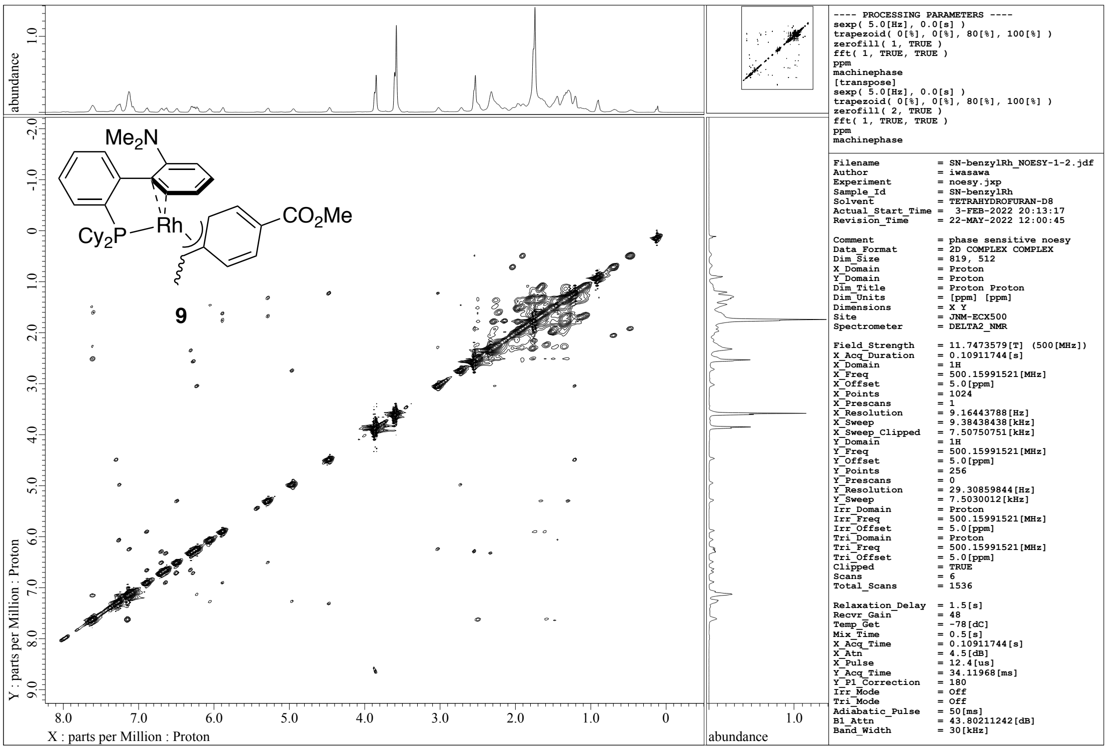

Supplementary Figure 76. NOESY NMR spectrum of **9** (500 MHz, –78 °C, THF-d_8)

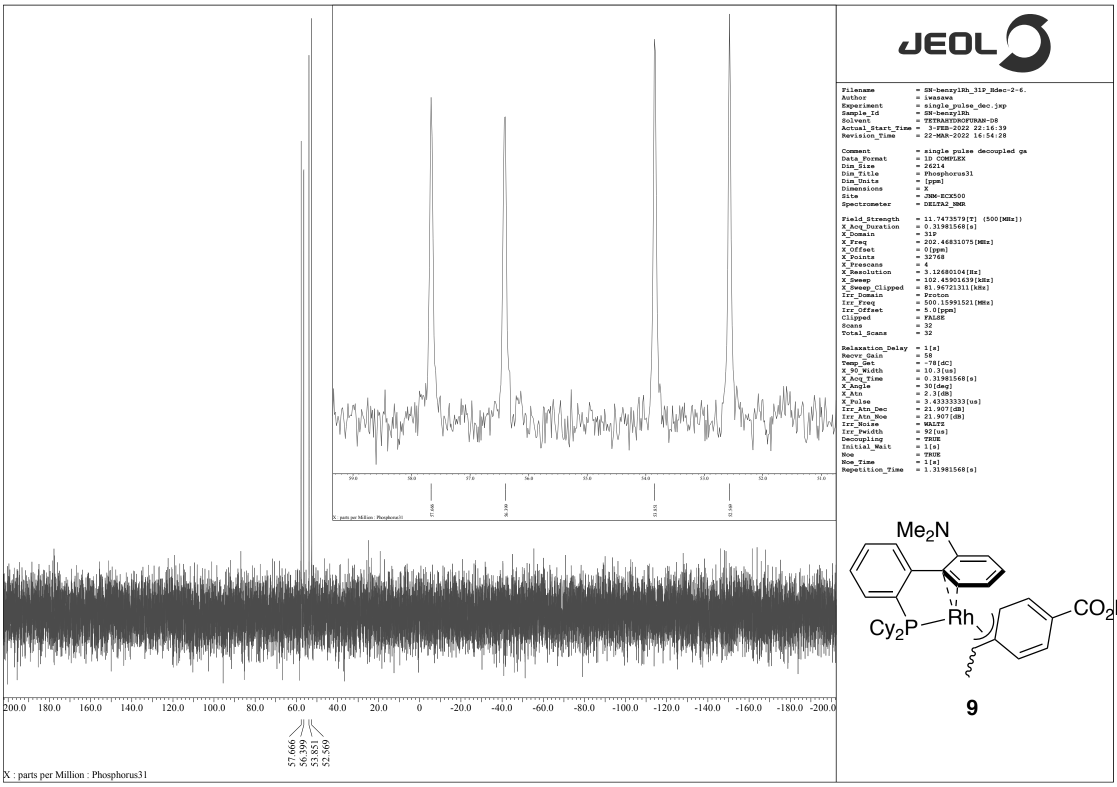

Supplementary Figure 77. ^{31}P NMR spectrum of **9** (202 MHz, –78 °C, THF-d_8)

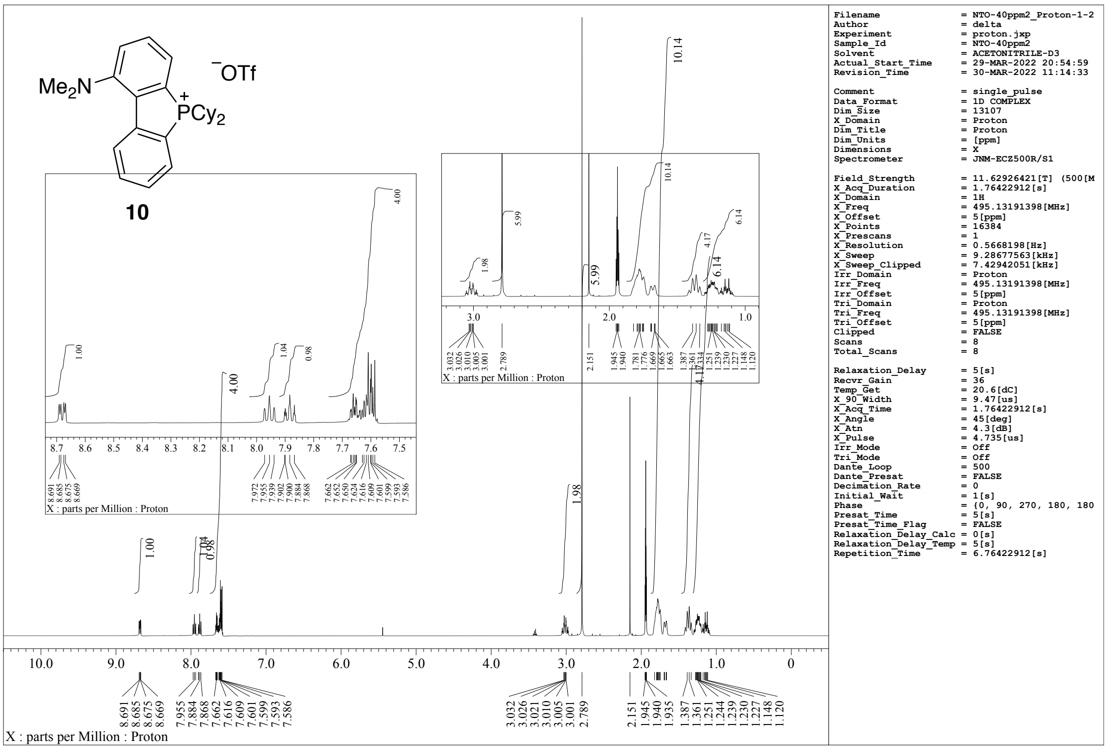

Supplementary Figure 78. ^{1}H NMR spectrum of **10** (495 MHz, r.t., CD_3CN)

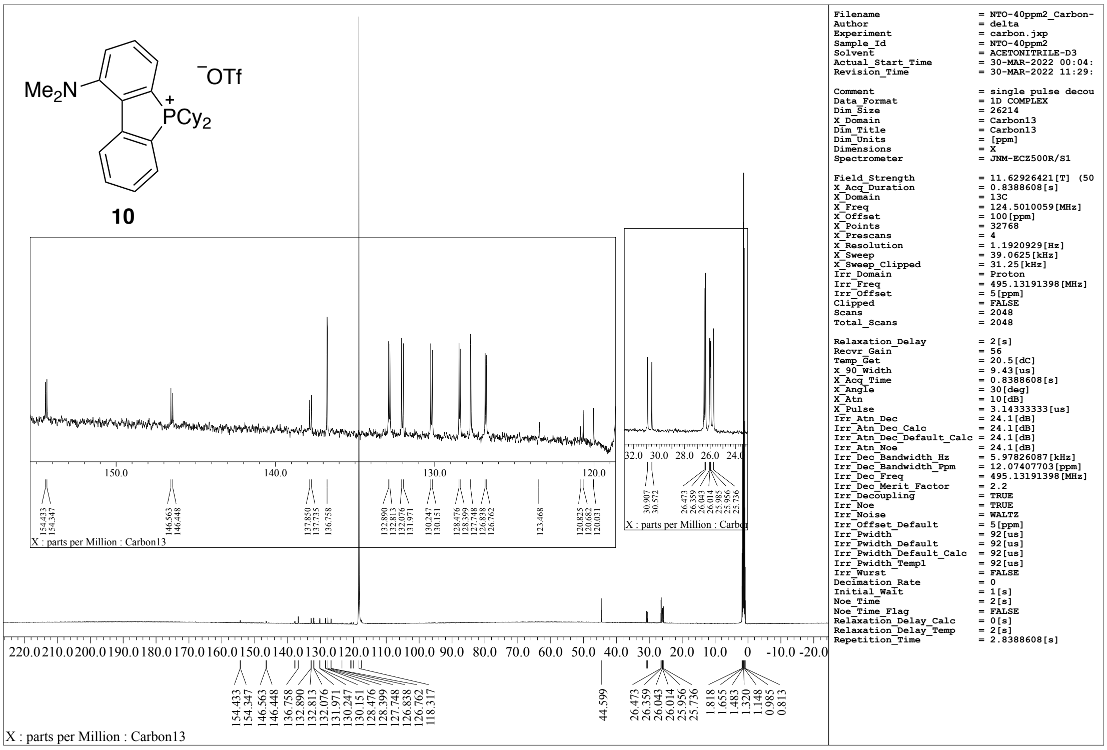

Supplementary Figure 79. ^{13}C NMR spectrum of **10** (125 MHz, r.t., CD_3CN)

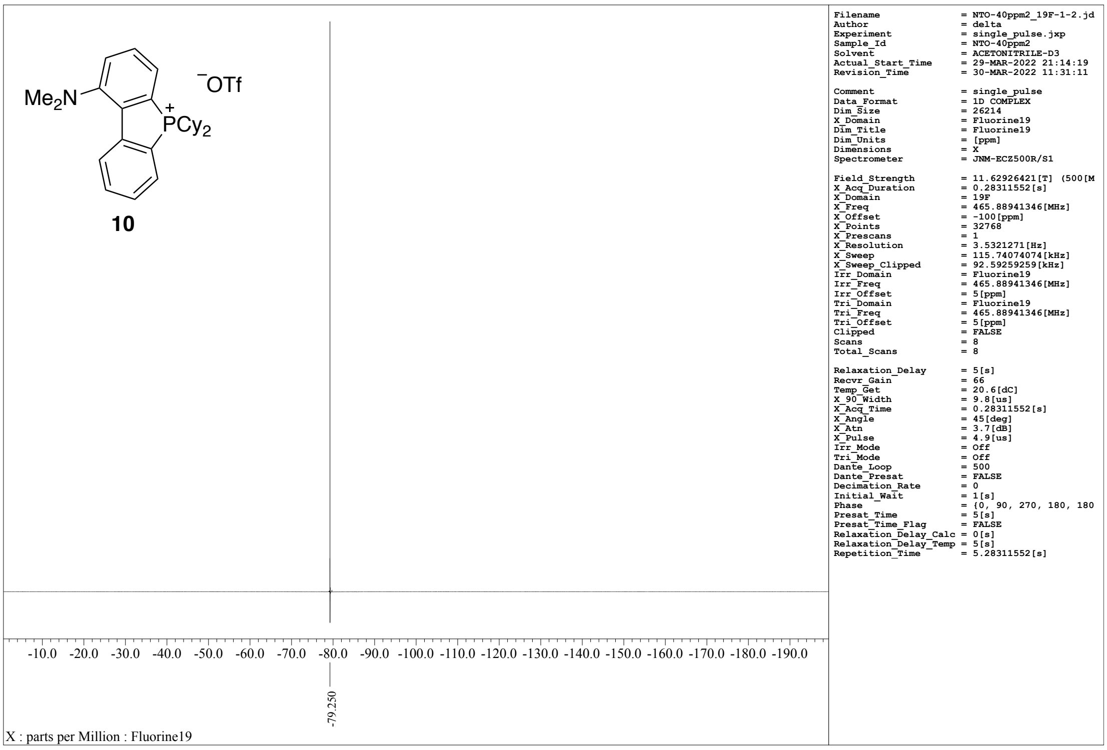

Supplementary Figure 80. ^{19}F NMR spectrum of **10** (466 MHz, r.t., CD_3CN)

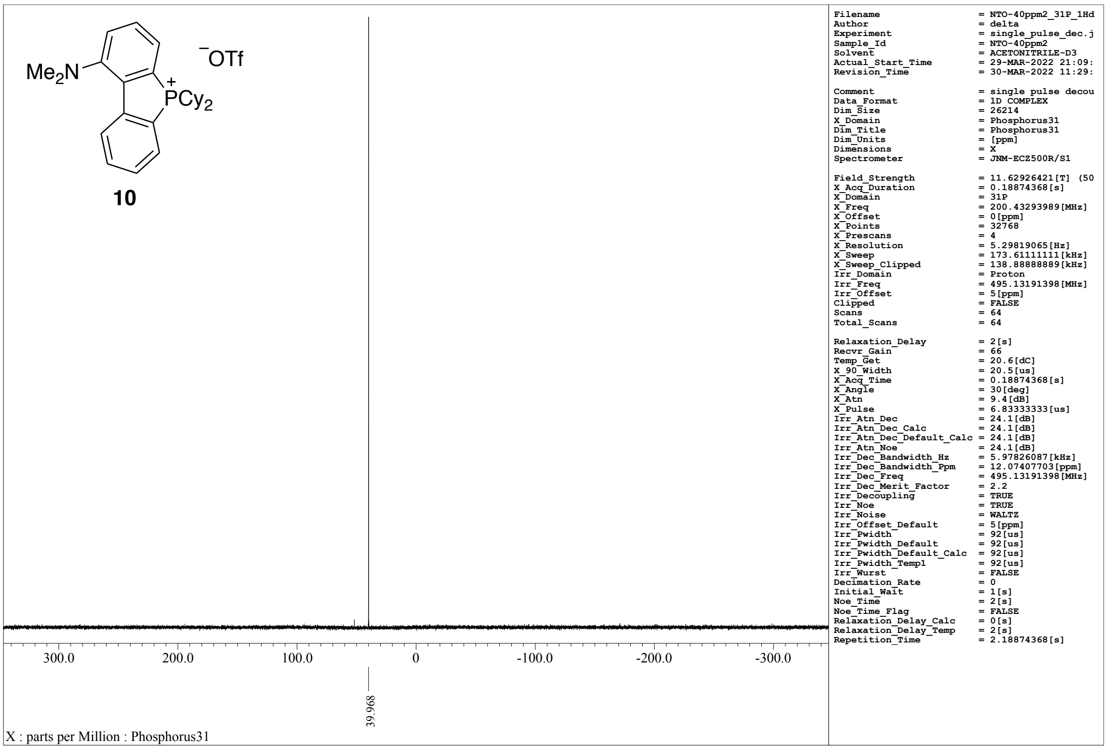

Supplementary Figure 81. ^{31}P NMR spectrum of **10** (200 MHz, r.t., CD_3CN)

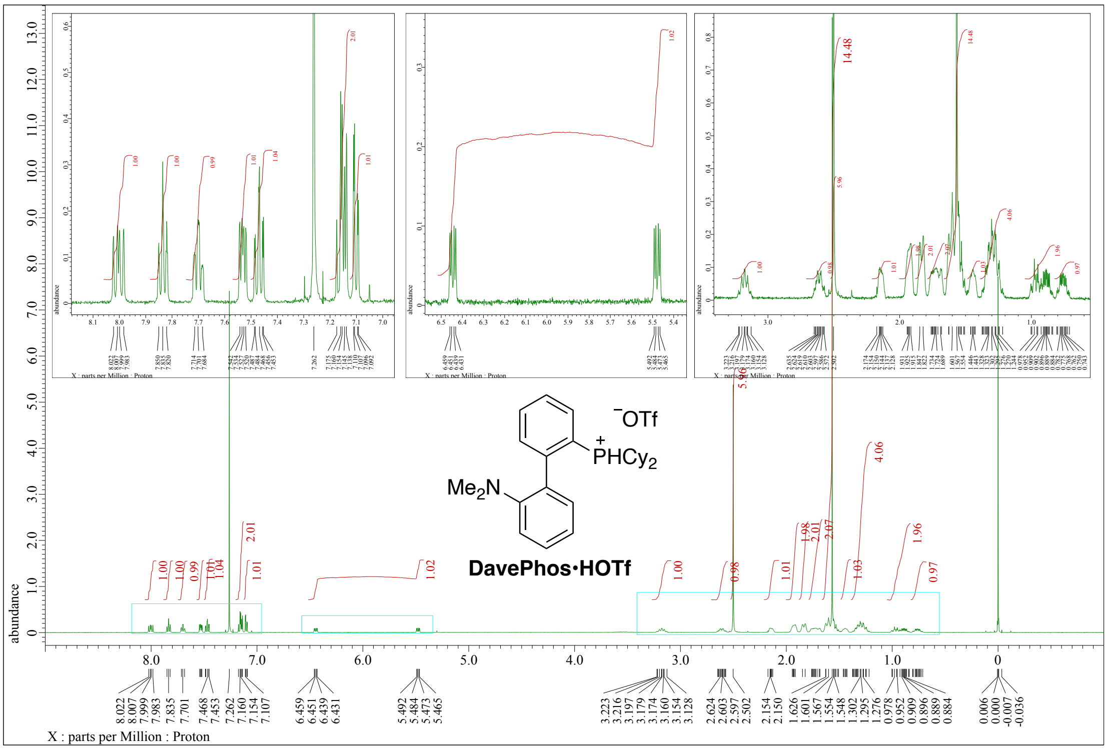

Supplementary Figure 82. ^{1}H NMR spectrum of **DavePhos•HOTf** (500 MHz, r.t., $CDCl_3$)

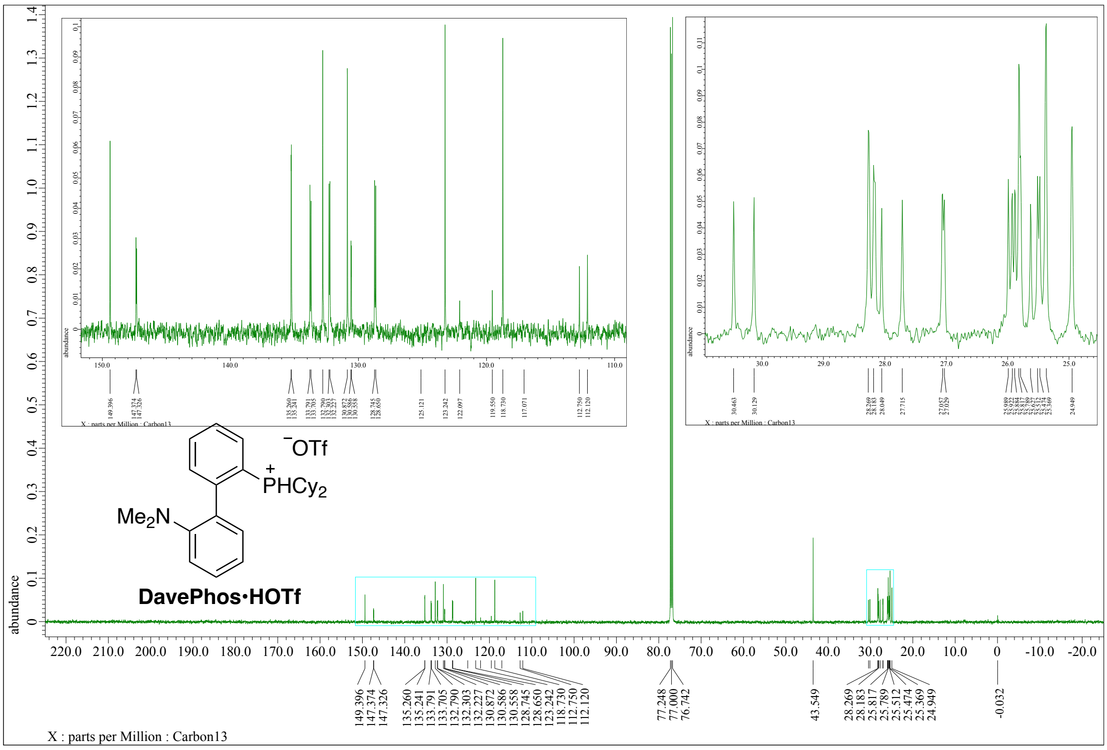

Supplementary Figure 83. ^{13}C NMR spectrum of **DavePhos•HOTf** (126 MHz, r.t., $CDCl_3$)

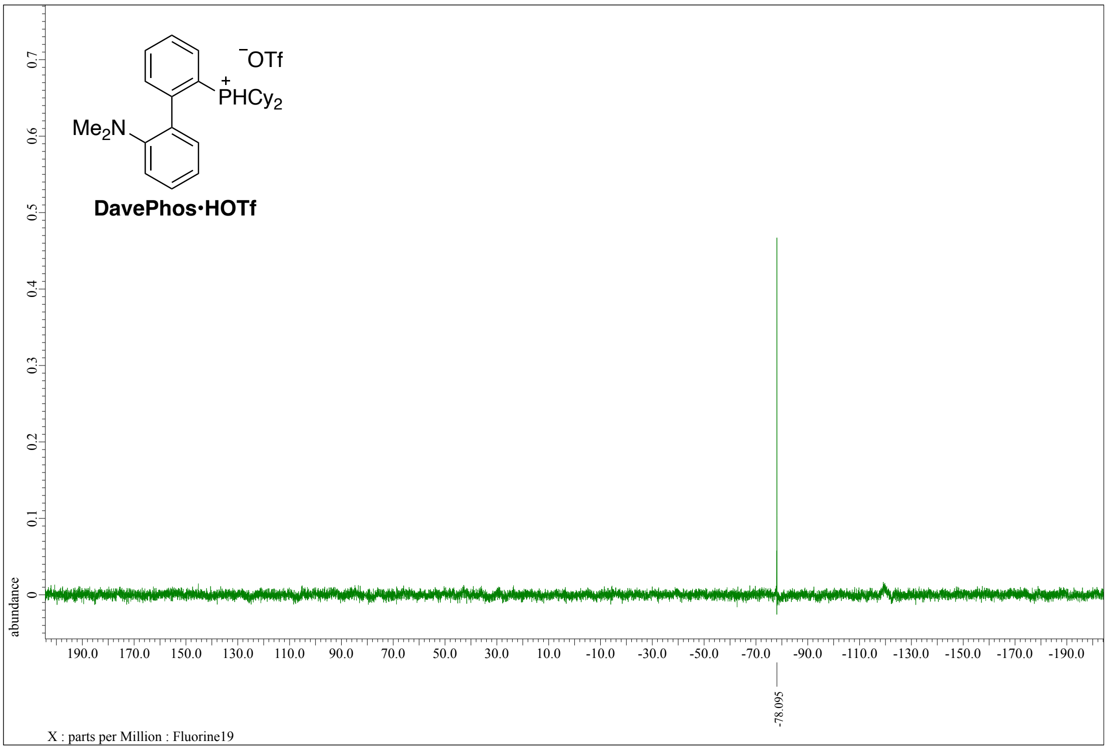

Supplementary Figure 84. ^{19}F NMR spectrum of **DavePhos•HOTf** (466 MHz, r.t., $CDCl_3$)

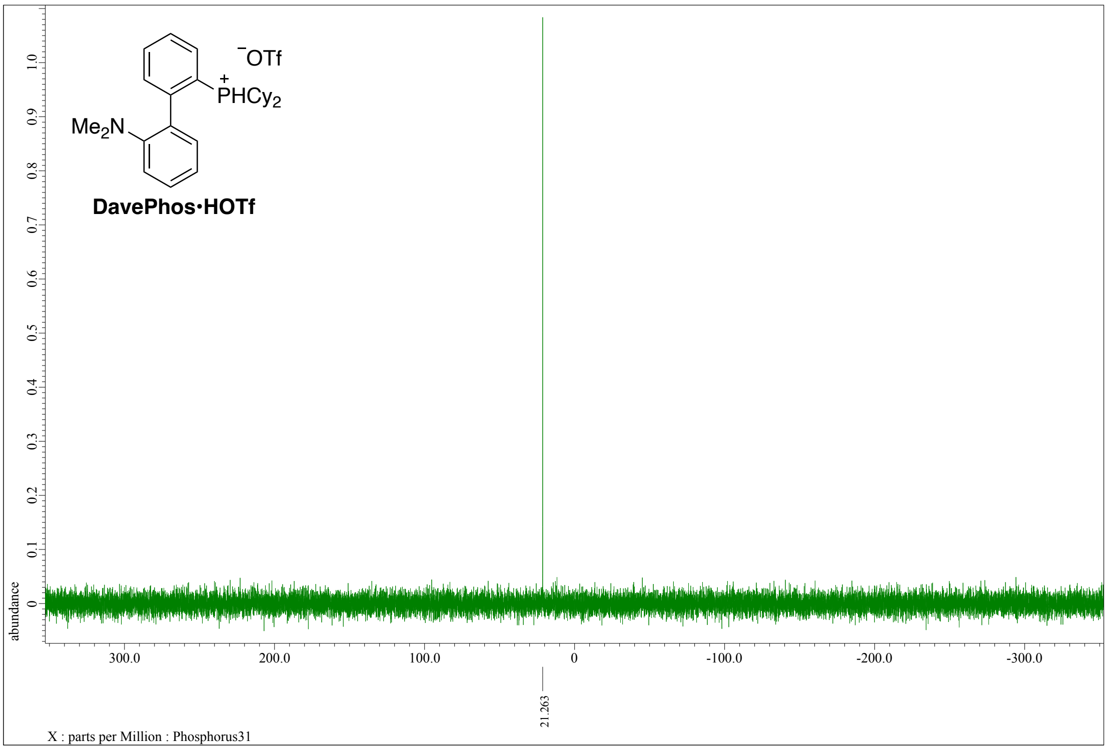

Supplementary Figure 85. ^{31}P NMR spectrum of **DavePhos•HOTf** (200 MHz, r.t., $CDCl_3$)

10. Supplementary References

[1] Kawashima, S.; Aikawa, K.; Mikami, K. *Eur. J. Org. Chem.* **2016**, 3166.
[2] Howe, R. K.; Schleppnik, F. M. *J. Heterocycl. Chem.* **1982**, *19*, 721.
[3] Hirao, A.; Imai, T.; Watanabe, K.; Hayashi, M.; Sugiyama, K. *Monatsh. Chem.* **2006**, *137*, 855.
[4] He, S.-J.; Wang, B.; Lu, X.; Gong, T.-G.; Yang, Y.-N.; Wang, X.-X.; Wang, Y.; Xiao, B.; Fu, Y. *Org. Lett.* **2018***, 20,* 5208.
[5] Deb, A.; Manna, S.; Modak, A.; Patra, T.; Maity, S.; Maiti, D. *Angew. Chem. Int. Ed.* **2013**, *52*, 9747.
[6] Rai, P; Maji, K.; Maji, B. *Org. Lett.* **2019**, 21, 3755.
[7] Neumann, H.; Brennführer, A.; Beller, M. *Chem. Eur. J.* **2008**, *14*, 3645.
[8] Xu, Z.; Xu, X.; O'Laoi, R.; Ma, H.; Zheng, J.; Chen, S.; Luo, L.; Hu, Z.; He, S.; Li, J.; Zhang, H.; Zhang, X. *Bioorg. Med. Chem.* **2016**, *24*, 5861.
[9] Ono, S.; Inoue, Y.; Yoshida, T.; Ashimori, A.; Kosaka, K.; Imada, T.; Fukaya, C.; Nakamura, N. *Chem. Pharm. Bull.* **1999**, *47*, 1685.
[10] Liu, Z.; Yamamichi, H.; Madrahimov, S. T.; Hartwig, J. F. *J. Am. Chem. Soc.* **2011**, *133*, 2772.
[11] Huang, Z.; Cheng, Y.; Chen, X.; Wang H-F.; Du, C-X.; Li, Y. *Chem. Commun.* **2018**, *54*, 3970.
[12] Das, S.; Incarvito, C. D.; Crabtree, R. H.; Brudvig, G. W. *Science*, **2006**, *312*, 1941.
[13] Murata, K.; Numasawa, N.; Shimomaki, K.; Takaya, J.; Iwasawa, N. *Chem. Commun.* **2017**, *53*, 3098.
[14] Meng, Q-Y.; Wang, S.; Huff G. S.; König. *J. Am. Chem. Soc.* **2018**, *140*, 3198.
[15] Scheetz, P. M.; Chachula, S. T.; Hughes, R. P.; Glueck, D. S.; Moore, C. E.; Gembicky, M.; Rheingold, A. L. *Organometallics* **2020**, *39*, 3802.
[16] Ge, Q.; Zong, J.; Li, B.; Wang, B. *Org. Lett.* **2017**, *19*, 6670.
[17] Sheldrick, G. M. *Acta Crystallogr., Sect. A* **2015**, *71*, 3.
[18] Sheldrick, G.M. *Acta Crystallogr., Sect. C* **2015**, *71*, 3.
[19] Gaussian 16, Revision B.01, Frisch, M. J.; Trucks, G. W.; Schlegel, H. B.; Scuseria, G. E.; Robb, M. A.; Cheeseman, J. R.; Scalmani, G.; Barone, V.; Petersson, G. A.; Nakatsuji, H.; Li, X.; Caricato, M.; Marenich, A. V.; Bloino, J.; Janesko, B. G.; Gomperts, R.; Mennucci, B.; Hratchian, H. P.; Ortiz, J. V.; Izmaylov, A. F.; Sonnenberg, J. L.; Williams-Young, D.; Ding, F.; Lipparini, F.; Egidi, F.; Goings, J.; Peng, B.; Petrone, A.; Henderson, T.; Ranasinghe, D.; Zakrzewski, V. G.; Gao, J.; Rega, N.; Zheng, G.; Liang, W.; Hada, M.; Ehara, M.; Toyota, K.; Fukuda, R.; Hasegawa, J.; Ishida, M.; Nakajima, T.; Honda, Y.; Kitao, O.; Nakai, H.; Vreven, T.; Throssell, K.; Montgomery, J. A., Jr.; Peralta, J. E.; Ogliaro, F.; Bearpark, M. J.; Heyd, J. J.; Brothers, E. N.; Kudin, K. N.; Staroverov, V. N.; Keith, T. A.; Kobayashi, R.; Normand, J.; Raghavachari, K.; Rendell, A. P.; Burant, J. C.; Iyengar, S. S.; Tomasi, J.; Cossi, M.; Millam, J. M.; Klene, M.; Adamo, C.; Cammi, R.; Ochterski, J. W.; Martin, R. L.; Morokuma, K.; Farkas, O.; Foresman, J. B.; Fox, D. J. Gaussian, Inc., Wallingford CT, **2016**.
[20] Maeda S.; Osada Y.; Morokuma K.; Ohno K. GRRM 11, Version 11.03, **2012**.
[21] Maeda, S.; Ohno, K.; Morokuma, K. *Phys. Chem. Chem. Phys.* **2013**, *15*, 3683.
[22] Ohno, K.; Maeda, S. *Chem. Phys. Lett.* **2004**, *384*, 277.
[23] Maeda, S.; Ohno, K. *J. Phys. Chem. A* **2005**, *109*, 5742
[24] Ohno, K.; Maeda, S. *J. Phys. Chem. A* **2006**, *110*, 8933.
[25] Zhao, Y.; Truhlar, D. G. *Theor. Chem. Acc.* **2008**, *120,* 215.
[26] (a) Cossi, M.; Barone, V.; Cammi, R.; Tomasi, J. *Chem. Phys. Lett.* **1996**, *255*, 327. (b) Cancès, E.; Mennucci, B.; Tomasi, J. *J. Chem. Phys.* **1997**, *107*, 3032.
