# Supplementary material for: Catalytic direct hydrocarboxylation of styrenes with CO2 and H2
Source: Nat Commun. 2022 Dec 8;13:7584. doi: 10.1038/s41467-022-35293-3 (PMC9732006; doi:10.1038/s41467-022-35293-3)
Supplement: Supplementary file 2 — Description of Additional Supplementary Files [file 41467_2022_35293_MOESM2_ESM.docx]

**Description of Additional Supplementary Files**

**Supplementary Data 1:**

Crystallographic data for compound **4** [Rh(C_2_H_4_)(DavePhos)](OTf)

**Supplementary Data 2:**

Crystallographic data for compound **8** [Rh(DavePhos)]_2_

**Supplementary Data 3:**

Cartesian coordinates of the optimized structure
