# Supplementary material for: Catalytic direct hydrocarboxylation of styrenes with CO2 and H2
Source: Nat Commun. 2022 Dec 8;13:7584. doi: 10.1038/s41467-022-35293-3 (PMC9732006; doi:10.1038/s41467-022-35293-3)
Supplement: Supplementary file 5 — Dataset 3 [file 41467_2022_35293_MOESM5_ESM.pdf]

Cartesian coordinates of the optimized structure

XYZ coordinates of structures (Å)

H_2

H	0.00000000	0.00000000	0.37106000
H	0.00000000	0.00000000	-0.37106000

CO_2

C	0.00000000	0.00000000	0.00000000
O	0.00000000	0.00000000	1.16406800
O	0.00000000	0.00000000	-1.16406800

1a

C	-0.60859500	0.12131600	-0.00027900
C	-0.08487800	-1.17132400	-0.00025400
C	1.28871600	-1.35807900	-0.00011300
C	2.17065500	-0.27044000	0.00002100
C	1.62927200	1.02334000	-0.00000100
C	0.26078200	1.21609300	-0.00014200
H	-0.75845900	-2.02324000	-0.00035700
H	1.69716300	-2.36755600	-0.00010200
H	2.29032700	1.88707800	0.00008600
H	-0.16851800	2.21472000	-0.00016600
C	3.61177800	-0.52994600	0.00017600
C	4.58919000	0.37786200	0.00040900
H	3.88470100	-1.58694200	0.00009500
H	5.63245000	0.07726200	0.00050900
H	4.39669000	1.44861000	0.00051300
C	-2.06354700	0.39575000	-0.00045600
O	-2.55487300	1.50211400	-0.00002500
O	-2.79728200	-0.73301200	-0.00003600
C	-4.20493600	-0.52862300	0.00038200
H	-4.65599100	-1.52159700	0.00068700
H	-4.51559100	0.03172000	0.88758700
H	-4.51615000	0.03143200	-0.88680900

2a

C	-1.55504600	0.09568800	0.09078600
C	-1.07564200	-1.19977900	-0.10400300
C	0.28806900	-1.44980700	-0.03700900
C	1.18800100	-0.41882900	0.22871900
C	0.70172100	0.87719200	0.42247000
C	-0.65798500	1.13288100	0.35236800
H	-1.77491000	-2.00482000	-0.30994900
H	0.66336100	-2.45994900	-0.19279700
H	1.40158000	1.68800300	0.62091300
H	-1.05375700	2.13488200	0.49680000
C	-2.99753300	0.43087400	0.02951000
O	-3.45236200	1.54055500	0.19142300
O	-3.76231400	-0.64568100	-0.22926500
C	-5.15865200	-0.38132300	-0.30027200
H	-5.63795900	-1.33730300	-0.51400500
H	-5.52484300	0.02601100	0.64706200
H	-5.37668900	0.34078300	-1.09303100
C	2.67246900	-0.69884600	0.34302000
H	2.85304400	-1.71773800	-0.02612600

C	3.16385300	-0.56079900	1.77791200
H	2.61073800	-1.24079900	2.43320100
H	4.23171200	-0.78978200	1.85317600
H	3.01859200	0.46279900	2.13662100
C	3.41674800	0.24462600	-0.57506500
O	3.98490900	1.25438900	-0.23642200
O	3.33967000	-0.15618900	-1.85606000
H	3.79386800	0.52205700	-2.37988900

3a

C	-0.70131100	0.11061100	-0.00006300
C	-0.20102600	-1.19465900	-0.00004500
C	1.16663000	-1.40747500	-0.00000800
C	2.07008900	-0.33800500	0.00001300
C	1.55844200	0.95993400	-0.00000400
C	0.18778800	1.18296900	-0.00004100
H	-0.89145000	-2.03322300	-0.00006000
H	1.55548200	-2.42569300	0.00000500
H	2.23443000	1.81159900	0.00001200
H	-0.21879100	2.19125100	-0.00005500
C	-2.15046100	0.41175200	-0.00010600
O	-2.62431500	1.52581100	-0.00004100
O	-2.90479700	-0.70448600	-0.00002400
C	-4.30814800	-0.47505000	0.00004200
H	-4.77699100	-1.45987400	0.00010900
H	-4.60942400	0.09062500	0.88713700
H	-4.60951900	0.09055000	-0.88707000
C	3.54744300	-0.63698400	0.00004500
H	3.76835600	-1.27060600	-0.87197600
H	3.76829600	-1.27071400	0.87200200
C	4.47301600	0.56546800	0.00014900
H	4.31884300	1.19336300	0.88551600
H	5.51995300	0.24730900	0.00019100
H	4.31894200	1.19344300	-0.88517900

CP1

P	0.77717900	-0.08256500	0.16800700
C	2.33335700	-1.10541100	-0.02682600
C	3.12804000	-1.07382300	1.28275100
C	3.25413000	-0.89835400	-1.23097500
H	1.89719800	-2.11603500	-0.11807400
C	4.25000900	-2.10629200	1.26883800
H	3.56997900	-0.07154400	1.41652800
H	2.45049800	-1.24435700	2.13092500
C	4.37311500	-1.93836000	-1.23103500
H	3.70435700	0.10699200	-1.20146500
H	2.68553100	-0.96454400	-2.16689500
C	5.16743100	-1.90634300	0.06879600
H	4.81989700	-2.05551500	2.20562400
H	3.80810900	-3.11454100	1.22588600
H	5.03293200	-1.77825300	-2.09393600
H	3.92944000	-2.93906200	-1.35835900
H	5.95962400	-2.66608800	0.05462400
H	5.67167600	-0.92989600	0.15876200
C	1.28192600	1.60305900	0.78479200
C	0.02373300	2.44378900	1.00894600
C	2.32626500	2.40000500	0.00236900
H	1.69857400	1.36367600	1.77989700

C	0.34636600	3.76608200	1.69413800
H	-0.46109200	2.63591100	0.03617200
H	-0.68877700	1.85738100	1.60449500
C	2.65379400	3.71065300	0.71561100
H	1.92859800	2.63714500	-0.99709600
H	3.24476200	1.81596500	-0.14496600
C	1.40052500	4.55187800	0.92388300
H	-0.57059400	4.35945400	1.81346100
H	0.71989800	3.56130100	2.71050900
H	3.40685000	4.27026800	0.14522700
H	3.10679400	3.48361500	1.69439100
H	1.64590900	5.48680300	1.44428200
H	0.99278200	4.83741600	-0.05963400
C	0.08203400	0.13851900	-1.54645200
C	-1.20986000	-0.37855500	-1.79015800
C	0.78078500	0.72230300	-2.61011400
C	-1.71899800	-0.36279800	-3.09027200
C	0.24179000	0.77083800	-3.89157100
H	1.77489800	1.13040500	-2.44419900
C	-1.00562500	0.21032100	-4.13641900
H	-2.68961500	-0.82048900	-3.28021600
H	0.80686300	1.22896600	-4.70003100
H	-1.42252100	0.21445700	-5.14093900
C	-1.99599000	-1.01264200	-0.68657600
C	-2.88832200	-0.26457600	0.15913400
C	-1.79158500	-2.40032600	-0.43699500
C	-3.41782000	-0.90095400	1.29585600
C	-2.35905600	-3.02940600	0.68912600
C	-3.08742200	-2.24342300	1.59310300
H	-4.06440800	-0.35123100	1.97287200
H	-2.17232000	-4.07968700	0.88780200
H	-3.47568400	-2.69132000	2.50401100
Rh	-0.86281600	-1.09818600	1.21387300
H	0.01603300	-0.91928100	2.55921300
H	-1.17454900	-2.96544600	-1.13305300
N	-3.17049000	1.08646500	-0.14874800
C	-4.10759800	1.26130700	-1.24675900
H	-4.13825200	2.31912600	-1.53329600
H	-5.13228600	0.94872000	-0.96834400
H	-3.79526400	0.68627500	-2.12130500
C	-3.53544500	1.92848400	0.97244300
H	-4.57164700	1.76216400	1.32468300
H	-3.45526300	2.97933100	0.66669600
H	-2.84993700	1.75764700	1.80878600

CP1'

P	-0.82575000	-0.10204400	-0.16482100
C	-2.21093800	-1.34888400	-0.00583900
C	-2.94351000	-1.44606200	-1.34894300
C	-3.19187700	-1.26711200	1.16482500
H	-1.63698900	-2.28651100	0.11925000
C	-3.92488500	-2.61312700	-1.36070100
H	-3.49975500	-0.51097700	-1.52991900
H	-2.21371200	-1.53986400	-2.16746800
C	-4.16568400	-2.44356200	1.13201100
H	-3.76493700	-0.32818000	1.11919700
H	-2.64549000	-1.25745500	2.11668000
C	-4.90569400	-2.51571900	-0.19830300

H	-4.45947300	-2.64688600	-2.31866000
H	-3.36205000	-3.55714600	-1.28255400
H	-4.87423100	-2.36808200	1.96719700
H	-3.60319800	-3.37923200	1.28308300
H	-5.60079200	-3.36513400	-0.20715400
H	-5.51934800	-1.60762200	-0.31924500
C	-1.54890600	1.52782700	-0.70837500
C	-0.43560500	2.57944300	-0.69008100
C	-2.81181800	2.06257000	-0.03158800
H	-1.78498300	1.32423800	-1.76945600
C	-0.89552400	3.88836200	-1.32039600
H	-0.12554800	2.76076400	0.35308800
H	0.44362000	2.18265300	-1.21157600
C	-3.26177200	3.37215800	-0.67748900
H	-2.60842600	2.24693000	1.03573400
H	-3.62674800	1.32857400	-0.08095500
C	-2.15434100	4.41789100	-0.64480400
H	-0.08714500	4.63048800	-1.27289500
H	-1.10153200	3.71961200	-2.38961600
H	-4.16518300	3.74529600	-0.17729100
H	-3.54265600	3.17523000	-1.72486200
H	-2.49017300	5.34831600	-1.12079400
H	-1.92506700	4.66691300	0.40434400
C	-0.15733800	0.11824700	1.53487700
C	1.16852000	-0.30660400	1.71127400
C	-0.86642300	0.61410100	2.63602900
C	1.73298700	-0.27797700	2.99057000
C	-0.28343500	0.67085700	3.89633100
H	-1.89390800	0.94930100	2.51074800
C	1.01742800	0.21119300	4.07599700
H	2.74939500	-0.64805400	3.12502000
H	-0.84882600	1.06070800	4.73965400
H	1.47385000	0.23078000	5.06304100
C	1.99552800	-0.85810600	0.59042200
C	2.89244400	-0.04276900	-0.19128100
C	2.14384200	-2.27310400	0.49375900
C	3.95188700	-0.67357600	-0.89839300
C	3.12284200	-2.85173900	-0.30284900
C	4.07277800	-2.04551100	-0.96156000
H	4.67321700	-0.03945300	-1.41121300
H	3.18453300	-3.93519700	-0.37125400
H	4.87684600	-2.50215700	-1.53202000
Rh	1.01456800	-0.89830300	-1.37946500
H	0.84267100	0.08195000	-2.71585400
H	1.47272900	-2.89772300	1.08189700
N	2.86168300	1.37518500	-0.03412400
C	3.70702100	1.84186900	1.05270700
H	3.55066100	2.91789900	1.19607700
H	4.78406200	1.67607500	0.85099200
H	3.44807000	1.33703100	1.98849900
C	3.12400500	2.12680500	-1.24835800
H	4.19257700	2.13674200	-1.53854200
H	2.81923600	3.17074500	-1.09275400
H	2.53840400	1.70258800	-2.07237000

CP2σ

Rh	1.00728600	-1.27492700	-0.07824500
P	0.47262900	0.87192000	0.21579900

C	-0.34022800	-1.96347200	-1.54184100
H	-0.13612300	-1.47592900	-2.50194900
C	-1.75299900	-1.86900500	-1.16749200
C	0.40864800	-3.25062400	-1.34882600
C	-2.30735500	-2.58048900	-0.08434400
C	-2.60988800	-1.00516000	-1.87704500
C	-3.92782700	-0.81270000	-1.49836000
C	-3.62321600	-2.39342400	0.29561300
C	-4.44872700	-1.49795500	-0.39653400
H	-2.21668600	-0.47711000	-2.74707900
H	-4.56651900	-0.13256100	-2.05628100
H	-1.67301100	-3.25441100	0.48998900
H	-4.04268500	-2.92419500	1.14748600
H	1.20359000	-3.19054900	-0.52539600
H	0.98152100	-3.54280400	-2.23451400
H	-0.21857700	-4.09030600	-1.02750300
C	-0.64553400	1.16701700	1.68620200
C	-2.09058300	0.79381500	1.34338900
C	-0.56606900	2.51648700	2.40291900
H	-0.28238500	0.40311800	2.39711300
C	-2.97032200	0.80823500	2.58798900
H	-2.50045000	1.50645800	0.60806500
H	-2.11886800	-0.19517500	0.87048700
C	-1.46064300	2.51506100	3.64141100
H	-0.88938100	3.32648700	1.73039000
H	0.47124300	2.73924200	2.68535600
C	-2.90136100	2.15756400	3.29247000
H	-4.00413500	0.55412600	2.31826000
H	-2.62897200	0.01849600	3.27701500
H	-1.41182800	3.49229700	4.13946900
H	-1.07142500	1.77794000	4.36245000
H	-3.52618300	2.15787900	4.19484900
H	-3.31346700	2.93361200	2.62594600
C	-0.26744800	1.76342600	-1.24064000
C	0.69255300	1.69908200	-2.43183500
C	-0.83002400	3.17839000	-1.09142700
H	-1.12047400	1.09871800	-1.46887400
C	0.00008500	2.13536400	-3.71879900
H	1.55291300	2.36142200	-2.23390600
H	1.09371500	0.68003100	-2.53136400
C	-1.54669100	3.59092100	-2.37602100
H	-0.01231000	3.89032100	-0.89466500
H	-1.52321800	3.24346000	-0.24376700
C	-0.61151500	3.52425900	-3.57730100
H	0.70713200	2.10830600	-4.55816000
H	-0.79615100	1.41109400	-3.95900200
H	-1.96721500	4.59912400	-2.26800300
H	-2.39999900	2.91032700	-2.53748100
H	-1.14178400	3.80824600	-4.49541100
H	0.19715000	4.26112200	-3.44247600
C	2.07313300	1.67230100	0.63371200
C	3.15676800	0.79797800	0.83600900
C	2.27304300	3.05480200	0.73522500
C	4.41318300	1.33710100	1.13688000
C	3.52877300	3.57536100	1.01706600
H	1.43459400	3.73344000	0.59037600
C	4.60034900	2.70935800	1.22011600
H	5.25010000	0.66137000	1.29736800

H	3.67124800	4.65161100	1.08169800
H	5.58655600	3.10647600	1.45046500
C	2.99504400	-0.69158400	0.80842800
C	3.95943700	-1.52654500	0.12170900
C	2.22394500	-1.29200300	1.85224500
C	4.11600400	-2.84684200	0.52353200
C	2.43813500	-2.63644400	2.25006600
C	3.37967800	-3.39069600	1.59454900
H	4.82375300	-3.48833100	0.00761800
H	1.88118500	-3.03888800	3.09250400
H	3.56053100	-4.42246800	1.88822800
C	-5.82926000	-1.31476000	0.08304200
O	-6.29821000	-1.83998200	1.07081400
O	-6.54169000	-0.47604900	-0.70038200
C	-7.87880300	-0.25974700	-0.27465700
H	-8.43856800	-1.20033200	-0.25092100
H	-8.32190900	0.42662700	-0.99816000
H	-7.90407500	0.17758900	0.72892600
H	1.69515600	-0.63232100	2.54057400
N	4.75018200	-0.96848700	-0.89320400
C	5.82380700	-1.78188700	-1.41676100
H	6.44869700	-1.16311700	-2.06954200
H	5.46636700	-2.64471100	-2.01038600
H	6.45228200	-2.15891000	-0.60346200
C	4.05446300	-0.25376100	-1.95130800
H	3.48510000	-0.94461100	-2.60223400
H	4.78716100	0.28325100	-2.56444700
H	3.35571000	0.47857300	-1.54289800

TS2σ-2

Rh	0.30267300	-1.06295600	-0.27289700
P	0.87041100	1.03640400	0.14536800
C	-0.74442800	-0.89162600	-2.08108900
H	-0.55924400	-0.03178800	-2.73420600
C	-2.11846600	-0.94497700	-1.58027200
C	-0.14223500	-2.18645700	-2.57876200
C	-2.63954100	-2.09927700	-0.95631400
C	-2.96862100	0.17614500	-1.68102300
C	-4.24312800	0.17003400	-1.14840500
C	-3.91400500	-2.10413800	-0.41602600
C	-4.72969400	-0.97191600	-0.49765800
H	-2.59697600	1.06634200	-2.19054200
H	-4.88036700	1.04644300	-1.23604500
H	-2.02806200	-2.99847100	-0.88955600
H	-4.31185100	-2.98954800	0.07461200
H	0.24524700	-2.82130900	-1.74286600
H	0.70816400	-2.00545900	-3.24324800
H	-0.87047000	-2.82967000	-3.09724500
C	-0.23187000	1.91929900	1.37438300
C	-1.53101300	2.35888100	0.69630300
C	0.36535900	3.03795600	2.23118400
H	-0.49585000	1.09442400	2.06024000
C	-2.53888200	2.87808000	1.71572100
H	-1.31860000	3.15827600	-0.03427100
H	-1.95758800	1.51785100	0.13487800
C	-0.66261700	3.54427000	3.24133500
H	0.68384500	3.87969000	1.59643400
H	1.26270300	2.68161900	2.75302300

C	-1.94321000	4.00272300	2.55353600
H	-3.45197000	3.21025000	1.20434700
H	-2.83782900	2.04904200	2.37689700
H	-0.23006900	4.35665300	3.83981800
H	-0.90300200	2.73162400	3.94583300
H	-2.66972200	4.36259200	3.29336000
H	-1.71333800	4.85913800	1.89773100
C	1.09462700	2.16564400	-1.32534600
C	2.11199700	1.54763500	-2.28853200
C	1.38803500	3.65481300	-1.12867600
H	0.09991800	2.08750000	-1.80380700
C	2.11771400	2.27727400	-3.62710400
H	3.11472500	1.60904100	-1.83166600
H	1.89148400	0.47839600	-2.42409500
C	1.38187400	4.38213600	-2.47249300
H	2.37858500	3.78111100	-0.66403700
H	0.65632800	4.11737100	-0.45417300
C	2.38226400	3.76694000	-3.44311900
H	2.86364700	1.83221700	-4.29824400
H	1.13770500	2.13848600	-4.11325900
H	1.59325900	5.44887900	-2.32251100
H	0.37012800	4.32201600	-2.90567600
H	2.35215200	4.28805900	-4.40858000
H	3.40070500	3.90462500	-3.04468000
C	2.49417100	0.86411300	0.99878800
C	2.88153100	-0.44681100	1.33482000
C	3.34154400	1.93345400	1.31691700
C	4.10448100	-0.64652800	1.98552200
C	4.56171200	1.71758300	1.94345800
H	3.04338600	2.95117700	1.07430000
C	4.94013800	0.42092300	2.28082600
H	4.39957700	-1.65991100	2.24729300
H	5.21243600	2.55826100	2.17286200
H	5.88908600	0.24177500	2.78153500
C	1.99570700	-1.62482100	1.06738300
C	2.53512200	-2.84717300	0.50022600
C	0.78144100	-1.71925200	1.82224600
C	1.83634600	-4.03259500	0.66721800
C	0.11461300	-2.96092700	1.98858300
C	0.63602100	-4.09067000	1.40860700
H	2.20935300	-4.94760300	0.21702400
H	-0.78895500	-3.00444200	2.59153200
H	0.13227600	-5.04831200	1.52094200
C	-6.07792300	-1.04475900	0.09053800
O	-6.54260400	-2.01380200	0.65285500
O	-6.76927300	0.10673900	-0.06011700
C	-8.07864700	0.08738400	0.48757200
H	-8.68697300	-0.69381900	0.02007400
H	-8.50738900	1.07102100	0.28824300
H	-8.04934200	-0.10292800	1.56548900
H	0.52577300	-0.89849400	2.49273900
N	3.76701000	-2.80535600	-0.16998900
C	4.37120700	-4.06968700	-0.52118900
H	5.39574500	-3.88752300	-0.86389300
H	3.83406300	-4.59690300	-1.33275800
H	4.41460600	-4.72994200	0.35091700
C	3.90364500	-1.83625500	-1.24514200
H	3.36831900	-2.16529400	-2.15600600

H	4.96506600	-1.71377500	-1.49126800
H	3.50719300	-0.86223200	-0.95372300

CP2

Rh	0.42908800	-0.01223200	-0.49194400
P	-1.69371500	-0.38797000	0.15122300
C	0.33341900	-1.05748100	-2.35465500
H	-0.14323700	-2.04394900	-2.31604800
C	1.72178500	-1.07813100	-1.95705900
C	-0.13848200	-0.23120100	-3.52539600
C	2.46890100	0.13643400	-1.91335800
C	2.32146500	-2.23135800	-1.36711600
C	3.57773700	-2.18911600	-0.81747500
C	3.74226000	0.16408300	-1.33843400
C	4.30302800	-0.97617400	-0.78563500
H	1.75730100	-3.16355100	-1.37861900
H	4.01970600	-3.08306700	-0.38600000
H	2.10466400	1.02154200	-2.43171900
H	4.31308800	1.08919200	-1.30460100
H	0.25347100	0.79230700	-3.51188000
H	-1.23290100	-0.15217800	-3.53299400
H	0.15742500	-0.68407200	-4.48438100
C	-1.87228400	-1.96369900	1.14765100
C	-1.79776600	-3.18573800	0.22695100
C	-3.02451300	-2.07357000	2.14868600
H	-0.93582800	-1.95516700	1.73404100
C	-1.73020800	-4.48026900	1.03090700
H	-2.68512400	-3.21757400	-0.42698900
H	-0.92226100	-3.09362700	-0.43125300
C	-2.93271500	-3.37868300	2.93609800
H	-3.99289700	-2.04196000	1.62647900
H	-3.00988000	-1.21653500	2.83433800
C	-2.89637800	-4.58514000	2.00589900
H	-1.71029900	-5.34403700	0.35372400
H	-0.78407600	-4.50263200	1.59524900
H	-3.77362500	-3.45340600	3.63777500
H	-2.01590000	-3.36698600	3.54751500
H	-2.83437700	-5.51642600	2.58339700
H	-3.84025800	-4.63127500	1.43729900
C	-3.03763600	-0.38311400	-1.15257300
C	-3.11293200	1.00983600	-1.78743200
C	-4.44317000	-0.89150100	-0.82016100
H	-2.61188800	-1.06664900	-1.91066400
C	-4.01075100	1.01855900	-3.01998000
H	-3.51231200	1.71915600	-1.04320900
H	-2.10106000	1.35652600	-2.04157700
C	-5.32820100	-0.88812600	-2.06561900
H	-4.90589300	-0.24502800	-0.05773100
H	-4.40692400	-1.90395300	-0.39852000
C	-5.40441100	0.49859900	-2.69086300
H	-4.06356600	2.03160300	-3.43931700
H	-3.56275300	0.38125300	-3.80040200
H	-6.33105000	-1.25602200	-1.81241000
H	-4.91247800	-1.59561600	-2.80151300
H	-6.03441000	0.48234600	-3.58944700
H	-5.88896900	1.18814200	-1.98042900
C	-2.07344900	0.98292700	1.31830300
C	-0.99900200	1.84214600	1.61252000

C	-3.33394800	1.24177700	1.87257600
C	-1.22400300	2.94280500	2.44875800
C	-3.54496800	2.34506900	2.68836500
H	-4.16586000	0.57402700	1.65791800
C	-2.48226800	3.19820900	2.97396900
H	-0.39219100	3.60462400	2.67901600
H	-4.53288500	2.53832400	3.10006600
H	-2.63358500	4.06268900	3.61679700
C	0.40115000	1.57504100	1.14465300
C	1.23501800	2.65436600	0.65881600
C	1.06252400	0.44551900	1.71649100
C	2.61336900	2.56741700	0.81895600
C	2.46237300	0.40081400	1.87106300
C	3.22187300	1.46126900	1.43524000
H	3.25423300	3.35939500	0.44370000
H	2.92357400	-0.46393200	2.34256800
H	4.30669900	1.43770400	1.52329700
C	5.62412700	-0.85411800	-0.14611400
O	6.23221000	0.18526700	0.01284300
O	6.10732600	-2.04441700	0.26898200
C	7.37665900	-1.98283700	0.90254800
H	8.13163900	-1.57090000	0.22516700
H	7.63128800	-3.00830400	1.17515900
H	7.33796000	-1.35058200	1.79569200
H	0.45746100	-0.26320400	2.28201300
N	0.63411500	3.78989000	0.09933200
C	1.50189900	4.88909600	-0.25809600
H	0.88498700	5.74778300	-0.54262000
H	2.16977300	4.65330900	-1.10824900
H	2.12117700	5.18539300	0.59454500
C	-0.39935800	3.57387500	-0.89844200
H	0.02152100	3.18569900	-1.84578000
H	-0.90523300	4.52416500	-1.10382900
H	-1.14942700	2.86389200	-0.54663700

CP2'

Rh	0.35935900	0.48487700	-0.71581500
P	-1.54641300	-0.50396000	0.13444700
C	0.38536200	0.12011000	-2.94071200
H	-0.32291600	-0.69447000	-3.11878400
C	1.65270100	-0.26477300	-2.42106900
C	0.18814900	1.33074800	-3.80475700
C	2.81720200	0.57938500	-2.44792800
C	1.77921200	-1.50617700	-1.70033200
C	2.92586700	-1.80959300	-0.98264500
C	3.94003600	0.25791200	-1.73674300
C	4.00560300	-0.92237600	-0.95316200
H	0.97006600	-2.23216000	-1.76498900
H	2.98228000	-2.73997100	-0.42146200
H	2.78976000	1.49431800	-3.03627100
H	4.80893600	0.91260900	-1.73894100
H	0.72496600	2.20690600	-3.41914700
H	-0.87418000	1.60136600	-3.85554400
H	0.52766600	1.16885100	-4.84090800
C	-1.31107300	-2.27844900	0.67739500
C	-1.51353500	-3.24556600	-0.49282500
C	-2.05279900	-2.77162100	1.91973500
H	-0.23132000	-2.28957500	0.91597500

C	-1.02680200	-4.64170200	-0.11945200
H	-2.58427800	-3.29683300	-0.75054200
H	-0.99491800	-2.88265700	-1.39189300
C	-1.58116100	-4.17588000	2.29246400
H	-3.13823800	-2.79317900	1.72633900
H	-1.89694600	-2.08064400	2.75885700
C	-1.74089200	-5.14845100	1.12905100
H	-1.17636000	-5.33388600	-0.95790200
H	0.05968200	-4.60238900	0.06572100
H	-2.12688300	-4.53609900	3.17407500
H	-0.51880800	-4.12857500	2.58100000
H	-1.37139700	-6.14358300	1.40725900
H	-2.81354100	-5.26592400	0.90296400
C	-3.06466500	-0.46900100	-0.94650700
C	-3.27751100	0.94188000	-1.50104000
C	-4.39338000	-1.03741900	-0.44515000
H	-2.73150400	-1.09565400	-1.79503500
C	-4.30503100	0.93063500	-2.62813300
H	-3.62836500	1.59813600	-0.68565700
H	-2.31897300	1.35085800	-1.85246100
C	-5.41214100	-1.07520700	-1.58274100
H	-4.78489400	-0.39124000	0.35541700
H	-4.26643900	-2.03995600	-0.01416900
C	-5.62400200	0.31376300	-2.17484300
H	-4.46613800	1.94867300	-3.00583600
H	-3.89759700	0.34646200	-3.47009600
H	-6.36337700	-1.49098000	-1.22587200
H	-5.04702700	-1.75771000	-2.36772800
H	-6.33693600	0.27221100	-3.00821900
H	-6.07766900	0.96234400	-1.40786800
C	-1.90350300	0.48942100	1.64453500
C	-0.93348300	1.44988200	1.99332300
C	-3.07673000	0.39543500	2.40582300
C	-1.18883300	2.31567800	3.06270500
C	-3.31713100	1.26321000	3.46305300
H	-3.81435800	-0.36802900	2.16919000
C	-2.37199600	2.23376600	3.78295900
H	-0.43882100	3.05898400	3.32378500
H	-4.23904200	1.18283500	4.03436800
H	-2.55092000	2.91955600	4.60816300
C	0.41758500	1.51305200	1.34296200
C	1.00266500	2.75384800	0.90443100
C	1.31023000	0.44652200	1.66076800
C	2.40087600	2.84266200	0.81226800
C	2.69530200	0.57968900	1.59595000
C	3.23494000	1.78649200	1.17264200
H	2.85743700	3.76043800	0.45576600
H	3.33393500	-0.25605900	1.87198500
H	4.31393600	1.89942100	1.08912500
C	5.18509500	-1.12279300	-0.11633500
O	6.12585200	-0.35534500	-0.02972900
O	5.12216200	-2.26672000	0.61342600
C	6.23417000	-2.48249000	1.46605400
H	7.16579700	-2.53979100	0.89328800
H	6.04780100	-3.42811200	1.97880700
H	6.33379400	-1.67032700	2.19483100
H	0.87781000	-0.44913800	2.10731300
N	0.18338400	3.86844500	0.65572600

C	0.84817100	5.11818800	0.35759500
H	0.10140000	5.91861800	0.33927800
H	1.36350100	5.11177200	-0.62171200
H	1.58129300	5.36046000	1.13320000
C	-0.96747400	3.66059400	-0.20547400
H	-0.66008100	3.42289900	-1.24369100
H	-1.57759500	4.57070000	-0.21878000
H	-1.59944700	2.84760900	0.15892500

TS2-3

Rh	0.19197900	-0.75817700	0.09942000
P	-1.88746500	0.15472500	0.10582000
C	0.26119100	-2.66324000	-1.61990600
H	0.40656000	-3.74425300	-1.49040000
C	1.55143800	-2.02145800	-1.27877500
C	-0.31117500	-2.39054300	-2.98901700
C	2.20539700	-1.08814000	-2.12767800
C	2.22278100	-2.41227600	-0.09184000
C	3.49833000	-1.93447900	0.19415000
C	3.46102100	-0.61063900	-1.82295400
C	4.12374800	-1.03082800	-0.65685500
H	1.75090000	-3.15306300	0.55023400
H	4.00586700	-2.26082000	1.09749300
H	1.71901200	-0.77567900	-3.04942000
H	3.96614800	0.09634000	-2.47821300
H	-0.44728700	-1.32219100	-3.18684000
H	-1.29180600	-2.86449800	-3.07828500
H	0.34320700	-2.79005700	-3.77635600
C	-3.02028700	-0.71362000	1.33325100
C	-4.30909200	-1.33714100	0.79188000
C	-3.33411800	0.09617000	2.59488400
H	-2.36924200	-1.55152500	1.63319800
C	-4.88203700	-2.28667200	1.84015700
H	-5.05011700	-0.54559200	0.59684300
H	-4.11779200	-1.87671100	-0.14142100
C	-3.93213900	-0.82342300	3.65825200
H	-4.05250000	0.89628600	2.35160700
H	-2.44352200	0.60098400	2.99336000
C	-5.16943400	-1.55105800	3.14417100
H	-5.79486300	-2.76114900	1.45785700
H	-4.15528800	-3.09637300	2.01226100
H	-4.17097400	-0.24637600	4.56117400
H	-3.17022100	-1.56348600	3.95011800
H	-5.54749400	-2.24412000	3.90670900
H	-5.97051500	-0.81407400	2.96856500
C	-2.65894200	0.27296200	-1.58527300
C	-1.68704600	0.95583900	-2.55333300
C	-4.02423300	0.95995100	-1.69782900
H	-2.75769200	-0.78785600	-1.87153300
C	-2.19767800	0.86691000	-3.98829000
H	-1.57986300	2.01578100	-2.26475500
H	-0.68705400	0.50897200	-2.46745900
C	-4.55453200	0.84440600	-3.12608100
H	-3.91100600	2.02775600	-1.45450900
H	-4.74999700	0.54287500	-0.99202600
C	-3.58882300	1.47466900	-4.12169700
H	-1.49272600	1.36061200	-4.67003700
H	-2.23540400	-0.19311400	-4.28786400

H	-5.54330100	1.31604400	-3.19584200
H	-4.69542500	-0.21984500	-3.37188700
H	-3.96202300	1.36114500	-5.14746000
H	-3.52979600	2.55794000	-3.92734500
C	-1.64513300	1.87711300	0.66354100
C	-0.32051600	2.22073700	0.96335200
C	-2.66352200	2.82846600	0.78329000
C	-0.03562900	3.54083300	1.33246600
C	-2.36721500	4.13550400	1.14562300
H	-3.69614800	2.54365900	0.58827400
C	-1.04576700	4.48994300	1.40788200
H	0.98980300	3.81586100	1.57194700
H	-3.16098500	4.87438100	1.22345800
H	-0.80351700	5.51103800	1.69412400
C	0.74508200	1.16968900	1.05707700
C	2.12322500	1.52819500	0.74353900
C	0.53541100	0.17793200	2.08409000
C	3.14506400	1.02157700	1.53770900
C	1.61967000	-0.37261900	2.81285200
C	2.88674500	0.09075700	2.56049500
H	4.17408000	1.31441800	1.34517200
H	1.42403100	-1.09330200	3.60159700
H	3.73193600	-0.29073700	3.13182900
C	5.43736600	-0.43885100	-0.35360300
O	5.97295900	0.43405000	-1.00870100
O	5.99130000	-0.94290400	0.76652200
C	7.23532500	-0.36069000	1.13119500
H	7.97485500	-0.48559200	0.33429000
H	7.55956300	-0.87749900	2.03555800
H	7.11965600	0.71132700	1.32692700
H	-0.44635600	0.12521600	2.55450400
N	2.38342300	2.39527400	-0.29029300
C	3.70673300	2.96399400	-0.44902700
H	3.64380300	3.80828300	-1.14217900
H	4.44364200	2.24351600	-0.83907500
H	4.07405300	3.35079400	0.50921900
C	1.49339800	2.51048200	-1.42561200
H	2.08441500	2.52208300	-2.35056500
H	0.87317400	3.41958000	-1.39517600
H	0.83229500	1.63704100	-1.46131700
C	-1.08878900	-2.90504300	-0.34246700
O	-2.17524700	-2.83244000	-0.89575900
O	-0.63776600	-3.29449800	0.72753800

TS2-3'

Rh	0.54205000	-0.66629500	-0.08611500
P	-1.25167500	0.83322900	0.05778600
C	-0.49950900	-4.54199800	-0.91041300
H	0.04166200	-5.44877600	-0.63694200
C	0.32895300	-3.45296300	-1.28135500
C	-1.83963500	-4.74625400	-1.55604100
C	-0.17960000	-2.30751700	-1.94786000
C	1.71222900	-3.42135900	-0.89478100
C	2.50153800	-2.32422700	-1.13688200
C	0.62408400	-1.19505700	-2.24202800
C	1.97963200	-1.16947900	-1.79821700
H	2.11516500	-4.27826700	-0.35785100
H	3.53848200	-2.31063600	-0.81269600

H	-1.21175400	-2.30996900	-2.28884200
H	0.27145800	-0.40039000	-2.89641600
H	-1.75421800	-4.90819800	-2.64041800
H	-2.49297600	-3.88062000	-1.39027700
H	-2.34889900	-5.61572700	-1.12860400
C	-2.83086700	-0.03648400	0.55240800
C	-3.47214100	-0.70973100	-0.66517900
C	-3.87054800	0.73686000	1.36433800
H	-2.44812000	-0.85566300	1.18685800
C	-4.61253700	-1.62772200	-0.23657500
H	-3.86285800	0.05761000	-1.35509200
H	-2.71310500	-1.28362600	-1.21347600
C	-5.00805500	-0.19179700	1.78499000
H	-4.28473500	1.56406300	0.76417400
H	-3.40677100	1.19116700	2.24953300
C	-5.65075400	-0.86617200	0.57865700
H	-5.07613000	-2.08602500	-1.12058700
H	-4.18925000	-2.44748500	0.36368100
H	-5.75578000	0.36843000	2.36168000
H	-4.60499700	-0.96519500	2.45780900
H	-6.45744500	-1.53768100	0.89942900
H	-6.11882900	-0.09590600	-0.05730500
C	-1.60138600	1.88625300	-1.43758700
C	-0.33469100	2.64850200	-1.83755500
C	-2.81105400	2.82225100	-1.46698300
H	-1.77009900	1.10598700	-2.20468700
C	-0.48799400	3.26610500	-3.22225200
H	-0.15523500	3.44642200	-1.09565500
H	0.54034700	1.98499200	-1.81870100
C	-2.97327400	3.42772600	-2.86073400
H	-2.66046200	3.63821400	-0.74283600
H	-3.73020200	2.29791300	-1.17388700
C	-1.71382900	4.16936100	-3.29515900
H	0.42173500	3.81858600	-3.48910500
H	-0.58032300	2.45329600	-3.96158200
H	-3.84294200	4.09746400	-2.88085700
H	-3.18622500	2.61801500	-3.57771400
H	-1.83813300	4.57500000	-4.30746900
H	-1.56165400	5.03432700	-2.62884600
C	-0.81515400	1.93638200	1.46175500
C	0.35667300	1.63396900	2.18171500
C	-1.56487600	3.06439700	1.82292500
C	0.73843100	2.46937600	3.23856400
C	-1.16462500	3.89305900	2.86203600
H	-2.48016800	3.29591000	1.28314000
C	-0.00683200	3.59069300	3.57278500
H	1.64310800	2.22987600	3.79340400
H	-1.75647300	4.76822800	3.11957800
H	0.31273400	4.22671600	4.39517200
C	1.20660900	0.42439200	1.92948000
C	2.62567900	0.53873600	1.73600500
C	0.68969800	-0.83375300	2.34405900
C	3.42019300	-0.59384000	1.96736800
C	1.50822100	-1.94483600	2.56548000
C	2.87635900	-1.80593700	2.38657300
H	4.49140400	-0.53968400	1.80082800
H	1.06531200	-2.89210300	2.85960200
H	3.53654600	-2.65481900	2.55100100

C	2.82112000	-0.03253400	-2.20871100
O	2.44830800	0.89786700	-2.89682700
O	4.08522600	-0.13097000	-1.74493800
C	4.97025800	0.88409600	-2.20025500
H	4.97698500	0.93316400	-3.29361600
H	5.95940200	0.61380000	-1.82532000
H	4.67790800	1.86727100	-1.81241700
H	-0.36644600	-0.88678200	2.61033700
N	3.20963500	1.76825300	1.41010600
C	4.65385900	1.84018700	1.42392400
H	4.95440100	2.88650300	1.30696700
H	5.12952700	1.25603800	0.61439100
H	5.04878100	1.48241400	2.38081700
C	2.62138700	2.57048700	0.34862400
H	2.76586900	2.12399800	-0.65059700
H	3.08575300	3.56290900	0.35809700
H	1.55008900	2.71182800	0.49900900
C	-0.90413300	-3.97055300	0.95743600
O	-1.83869100	-3.21214000	0.90537800
O	-0.10954200	-4.54091300	1.65645700

CP3

Rh	0.15219900	-0.12659900	0.81693400
P	-1.86062400	0.43968000	0.08799500
C	0.55128700	-3.66287000	0.47353500
H	0.75278800	-4.27977400	1.36686100
C	1.70230100	-2.69442300	0.38125400
C	0.40925200	-4.60424800	-0.71160900
C	2.47325500	-2.55710100	-0.77805100
C	2.09521700	-1.96555700	1.52437800
C	3.24615900	-1.19121500	1.52041600
C	3.62289500	-1.77822200	-0.78485200
C	4.03288000	-1.10845300	0.36680400
H	1.52296800	-2.07927600	2.44302500
H	3.54656000	-0.65795100	2.41804400
H	2.19398000	-3.09758900	-1.67931500
H	4.23913500	-1.69980500	-1.67872400
H	0.16030000	-4.05890200	-1.63203200
H	-0.41285900	-5.29722700	-0.52253900
H	1.32800900	-5.17495300	-0.88917800
C	-2.99011000	0.92152900	1.49074300
C	-3.59948400	-0.31880700	2.15601100
C	-4.05191700	1.98467700	1.19794100
H	-2.28121900	1.36028600	2.21484200
C	-4.34144200	0.07419100	3.42919700
H	-4.31371200	-0.79388400	1.46358500
H	-2.81782400	-1.06298400	2.35513500
C	-4.79212700	2.35652700	2.48070800
H	-4.77921800	1.60071400	0.46459700
H	-3.59321200	2.87710500	0.75151200
C	-5.40409300	1.12988800	3.14719900
H	-4.79114000	-0.81561600	3.88731900
H	-3.61951700	0.47079600	4.16129600
H	-5.56280700	3.10728500	2.26292700
H	-4.08214700	2.83086200	3.17755900
H	-5.92294900	1.41559900	4.07127100
H	-6.16886700	0.70127000	2.47868100
C	-2.69342100	-0.89144300	-0.89099300

C	-1.78835900	-1.38818600	-2.02194300
C	-4.08932500	-0.57618200	-1.43672200
H	-2.77860400	-1.71513100	-0.16154500
C	-2.39042200	-2.62178600	-2.68848500
H	-1.65491800	-0.58199800	-2.76524500
H	-0.78850500	-1.62211500	-1.62517300
C	-4.68978100	-1.82868000	-2.07246500
H	-4.02065600	0.21208200	-2.20360600
H	-4.75387000	-0.20233500	-0.64640000
C	-3.80140900	-2.34944200	-3.19539500
H	-1.74345100	-2.95590700	-3.51109400
H	-2.41282800	-3.43262500	-1.94427800
H	-5.69987300	-1.61198800	-2.44427500
H	-4.79298500	-2.60521900	-1.29810800
H	-4.23274000	-3.25739500	-3.63589900
H	-3.76317500	-1.59721500	-4.00093900
C	-1.59064800	1.88740300	-0.99188900
C	-0.25684200	2.31582500	-1.06433200
C	-2.57881700	2.53507300	-1.74342500
C	0.06003300	3.38657500	-1.90836500
C	-2.24620300	3.58592600	-2.58728800
H	-3.61561000	2.21354000	-1.67384000
C	-0.92077400	4.00646600	-2.66958100
H	1.09094400	3.73195300	-1.96121000
H	-3.01659000	4.07544300	-3.17797600
H	-0.65220200	4.83343600	-3.32327600
C	0.79352900	1.73451800	-0.16571500
C	2.16227700	1.64614400	-0.66188700
C	0.60816200	1.97934700	1.24261600
C	3.21114500	1.93898800	0.20075700
C	1.72004300	2.17305100	2.10593200
C	2.98286500	2.18982700	1.56749100
H	4.23174100	1.92946000	-0.17254200
H	1.55248000	2.39203200	3.15702100
H	3.84338200	2.38042500	2.20763200
C	5.29462500	-0.34602500	0.32020200
O	5.94243700	-0.13310000	-0.68555700
O	5.67137800	0.10345200	1.53156400
C	6.84773500	0.90077500	1.53663400
H	7.69382100	0.35405500	1.10970600
H	7.04145800	1.14987100	2.58098800
H	6.69772600	1.81554700	0.95107900
H	-0.34574400	2.40285300	1.56417800
N	2.38065700	1.32087600	-1.97768400
C	3.68003300	1.54604800	-2.57838300
H	3.57739700	1.49261600	-3.66655700
H	4.43946200	0.81675600	-2.25683500
H	4.04468400	2.55019000	-2.32864600
C	1.45417200	0.48820500	-2.71708800
H	2.01982100	-0.23869900	-3.31372200
H	0.80169000	1.06296000	-3.39288300
H	0.82403800	-0.07398800	-2.01620000
C	-0.81268400	-3.00748600	0.82285100
O	-1.83862200	-3.60869000	0.51578600
O	-0.78136800	-1.90592200	1.49063200

CP3'

Rh	-0.15076600	1.01730800	0.34628200

P	1.37722100	-0.71814200	0.01526100
C	-0.01504200	3.99675000	-0.50374700
H	-0.40364700	4.39641400	0.44788400
C	-0.90775200	2.84614000	-0.89206700
C	-0.03742900	5.09236000	-1.56018800
C	-0.54016500	1.96885100	-1.94696600
C	-2.18443900	2.66696500	-0.31469300
C	-3.06852300	1.70423000	-0.79154300
C	-1.44622500	1.02873900	-2.44648200
C	-2.71094200	0.88743400	-1.87532300
H	-2.47801600	3.31276200	0.51151200
H	-4.04011800	1.57685000	-0.32110600
H	0.42755200	2.10427300	-2.43032600
H	-1.17111600	0.37604700	-3.27303100
H	-1.05237400	5.47763000	-1.71566600
H	0.33977400	4.71274500	-2.51697500
H	0.62194800	5.91038000	-1.25881600
C	3.10320300	-0.05902300	0.20858500
C	3.53364300	0.67058700	-1.06900800
C	4.20772900	-0.98039300	0.72379500
H	2.93950600	0.73565200	0.95575300
C	4.80311500	1.47376100	-0.81232500
H	3.71519100	-0.05975200	-1.87658300
H	2.73774400	1.34963800	-1.40029800
C	5.47900400	-0.16945600	0.97218400
H	4.42467400	-1.77388200	-0.01052500
H	3.89404200	-1.48268900	1.64889700
C	5.91798900	0.58086400	-0.28065200
H	5.11712300	1.97866800	-1.73453100
H	4.56090400	2.27150100	-0.09436000
H	6.28037300	-0.82865300	1.33164100
H	5.28312200	0.55651500	1.77705600
H	6.82063600	1.17036200	-0.07444000
H	6.19540400	-0.15276700	-1.05671700
C	1.27992000	-1.65721400	-1.59493800
C	-0.11638000	-2.25475900	-1.79137300
C	2.35128800	-2.70345200	-1.91193100
H	1.39203700	-0.83467200	-2.32665800
C	-0.28986000	-2.80396100	-3.20381100
H	-0.25812100	-3.07074200	-1.06126900
H	-0.89184400	-1.50142600	-1.58698800
C	2.18358300	-3.22490600	-3.33762800
H	2.25224600	-3.55064500	-1.21533900
H	3.35883300	-2.28994500	-1.77572300
C	0.79518600	-3.81736900	-3.54592700
H	-1.28898800	-3.24234500	-3.31387700
H	-0.24827400	-1.96596200	-3.91879600
H	2.96055800	-3.96809500	-3.55893300
H	2.33681300	-2.39260700	-4.04370800
H	0.67859800	-4.17304500	-4.57758100
H	0.68240700	-4.70090700	-2.89629700
C	1.06140500	-1.90609800	1.38217400
C	0.04266600	-1.59964200	2.30740600
C	1.75232100	-3.11976300	1.50751700
C	-0.26413000	-2.53014400	3.30849300
C	1.43423200	-4.03228300	2.50344700
H	2.55616300	-3.35287600	0.81326100
C	0.41645200	-3.73483400	3.40446200

H	-1.05240100	-2.29254800	4.01909100
H	1.97948500	-4.97020100	2.57705100
H	0.15906200	-4.43883600	4.19251200
C	-0.68537200	-0.29139600	2.35422200
C	-2.11901400	-0.23015600	2.44234800
C	0.05752600	0.86384100	2.70651700
C	-2.70850400	0.94295500	2.91599100
C	-0.56998000	2.03396500	3.17080500
C	-1.94361200	2.05284200	3.29417300
H	-3.78973600	1.01183800	2.98687700
H	0.04019000	2.89267100	3.43654900
H	-2.44928100	2.94177500	3.66558500
C	-3.56975100	-0.20828100	-2.36036600
O	-3.25479100	-1.01332600	-3.21133200
O	-4.75245300	-0.25577500	-1.71227800
C	-5.58035600	-1.35762200	-2.06031500
H	-5.81023700	-1.35295400	-3.13003000
H	-6.49365100	-1.24893300	-1.47332500
H	-5.08284600	-2.30375900	-1.81787900
H	1.14119200	0.78277700	2.77769200
N	-2.89012800	-1.35625300	2.11417400
C	-4.30395700	-1.30034000	2.41053800
H	-4.73066400	-2.29951800	2.27151800
H	-4.85643500	-0.60394400	1.75029600
H	-4.47142900	-1.00369300	3.45077700
C	-2.65149600	-1.97065400	0.81813400
H	-3.06359800	-1.35336500	-0.00213100
H	-3.13552400	-2.95380500	0.78898200
H	-1.58604800	-2.11988400	0.63685500
C	1.42797000	3.52402300	-0.18721100
O	1.49996500	2.48758400	0.57643800
O	2.38352900	4.14597000	-0.63463300

CP3-MECP

Rh	-0.10678500	1.01593400	0.36561900
P	1.40687000	-0.69125500	0.06299900
C	0.01192400	3.98152300	-0.56403500
H	-0.34379800	4.36407800	0.40673900
C	-0.89479500	2.83881700	-0.94524400
C	-0.03255300	5.09646800	-1.59882200
C	-0.60167700	2.03732800	-2.07788100
C	-2.10712600	2.59195600	-0.26798500
C	-3.00600600	1.62470600	-0.72270200
C	-1.50790400	1.09076400	-2.53987900
C	-2.71854200	0.87692800	-1.86731300
H	-2.35251700	3.19116800	0.60754500
H	-3.92949300	1.44485100	-0.17781600
H	0.33350100	2.21040600	-2.61136000
H	-1.28678000	0.48499700	-3.41650200
H	-1.05098700	5.48253100	-1.72621800
H	0.32779000	4.73805800	-2.56967400
H	0.62939800	5.91194200	-1.29564400
C	3.14105700	-0.05380600	0.23968100
C	3.57608900	0.64744800	-1.05262200
C	4.23570600	-0.98195700	0.76395000
H	2.99119000	0.75407700	0.97532200
C	4.85332200	1.44209900	-0.81096500
H	3.75143100	-0.10135400	-1.84458100

H	2.78852900	1.32923300	-1.39741200
C	5.51666400	-0.18158300	0.99714100
H	4.44112400	-1.78668600	0.03831800
H	3.92025000	-1.46878100	1.69665400
C	5.96064400	0.54728100	-0.26670800
H	5.16961100	1.92965200	-1.74158000
H	4.61899800	2.25353800	-0.10604400
H	6.31173300	-0.84472500	1.36322600
H	5.33090500	0.55680200	1.79307600
H	6.86888200	1.13120900	-0.06932600
H	6.23094900	-0.19970200	-1.03239500
C	1.27998600	-1.59858600	-1.56139000
C	-0.13689800	-2.13211600	-1.78628100
C	2.30633200	-2.69179900	-1.86868900
H	1.43829400	-0.77294900	-2.28044300
C	-0.31046800	-2.64721900	-3.21110300
H	-0.32337900	-2.95567800	-1.07481600
H	-0.88105100	-1.35005000	-1.57715500
C	2.14040000	-3.19166900	-3.30262300
H	2.15139200	-3.53766700	-1.18036300
H	3.33051300	-2.32963400	-1.70997500
C	0.72795400	-3.71026300	-3.54675600
H	-1.33104400	-3.02399600	-3.34993400
H	-0.20546200	-1.80029600	-3.90918600
H	2.88284500	-3.97168100	-3.51563600
H	2.35096000	-2.36165400	-3.99652700
H	0.61557600	-4.04563500	-4.58563100
H	0.55716300	-4.59564200	-2.91244800
C	1.07150800	-1.89426300	1.40990700
C	0.02724200	-1.60150900	2.31037400
C	1.77092600	-3.10203900	1.54282100
C	-0.29305400	-2.53911300	3.30037500
C	1.43833100	-4.02183700	2.52739800
H	2.59051500	-3.32532700	0.86372400
C	0.39760100	-3.73736400	3.40636000
H	-1.10088700	-2.31309700	3.99261600
H	1.98940100	-4.95564200	2.60893000
H	0.12948500	-4.44744500	4.18536100
C	-0.71302300	-0.29970700	2.33603300
C	-2.14868000	-0.24795200	2.40426700
C	0.02094200	0.85989800	2.70511600
C	-2.75171800	0.92203100	2.87296500
C	-0.62123800	2.02529000	3.16130300
C	-1.99787800	2.03468600	3.26195100
H	-3.83386400	0.98597500	2.93015000
H	-0.02198600	2.88633000	3.44419300
H	-2.51417500	2.92011100	3.62767200
C	-3.58002400	-0.22736500	-2.32769900
O	-3.29936100	-1.00202600	-3.21798100
O	-4.71890400	-0.32311200	-1.60917900
C	-5.53757200	-1.43958500	-1.93158300
H	-5.83430200	-1.41744200	-2.98443900
H	-6.41469800	-1.36942400	-1.28632700
H	-5.00113700	-2.37681100	-1.74313600
H	1.10184300	0.77656900	2.81349300
N	-2.91283700	-1.37595300	2.07275200
C	-4.33552100	-1.31423700	2.32281100
H	-4.75949800	-2.31374700	2.17777000

H	-4.86345700	-0.62227100	1.63845300
H	-4.53762200	-1.00922400	3.35442400
C	-2.63333900	-2.04341200	0.81194100
H	-3.04361000	-1.47530100	-0.04277900
H	-3.09420200	-3.03827500	0.81969700
H	-1.56223200	-2.17606000	0.65715900
C	1.45798700	3.48421800	-0.30470800
O	1.54311800	2.48529600	0.50750100
O	2.40331100	4.04769900	-0.84219800

CP4

P	1.71959300	-0.88357000	0.17157800
C	-3.21952100	0.65406900	-2.14672900
C	-4.14444300	-0.37827800	-2.29715400
C	-3.18105100	1.34653500	-0.92807100
C	-4.02353300	0.99960900	0.11295800
C	-5.00876900	-0.71830800	-1.26194300
C	-4.94691000	-0.03853400	-0.04684100
H	-2.45760300	2.15464500	-0.80434900
H	-3.98440600	1.51633500	1.06983100
H	-4.19276100	-0.93351500	-3.23051900
H	-5.72613400	-1.52449800	-1.38847900
C	3.06351500	-1.92284200	-0.58879400
C	2.50146900	-2.69387200	-1.78847000
C	3.89931600	-2.82224600	0.32445800
H	3.74234900	-1.14960700	-0.99147400
C	3.62005400	-3.38122100	-2.56396800
H	1.79275800	-3.46043900	-1.43377400
H	1.93174500	-2.01355700	-2.43742100
C	5.00675800	-3.50742600	-0.47346900
H	3.26438300	-3.59329900	0.78826400
H	4.33162900	-2.23379400	1.14433400
C	4.44115100	-4.28773300	-1.65445600
H	3.20069700	-3.95044200	-3.40326000
H	4.27784200	-2.61446800	-3.00362500
H	5.58977600	-4.16528100	0.18392000
H	5.70506100	-2.74148500	-0.84775300
H	5.24963400	-4.77083500	-2.21781400
H	3.79618400	-5.09792000	-1.27565200
C	0.23815100	-1.95997200	0.48093400
C	-0.86971800	-1.13919300	1.14939200
C	0.44746800	-3.28236800	1.22077700
H	-0.09797800	-2.17641600	-0.54898200
C	-2.16946400	-1.93053700	1.22617500
H	-0.54599000	-0.86448900	2.16876700
H	-1.02911900	-0.20545200	0.59170600
C	-0.85930100	-4.07119400	1.28726800
H	0.79548200	-3.07702900	2.24671600
H	1.22134500	-3.89104500	0.73489200
C	-1.96870200	-3.25915600	1.94399100
H	-2.94087700	-1.32862000	1.72583900
H	-2.53189000	-2.10988600	0.19990400
H	-0.69855800	-5.01607300	1.82279900
H	-1.16308200	-4.33891600	0.26229500
H	-2.90348000	-3.83404300	1.96522600
H	-1.69766800	-3.06422600	2.99460700
C	2.34182600	-0.21174300	1.75381000
C	2.40730500	1.19192700	1.78864100

C	2.70259400	-0.95999300	2.88006600
C	2.84287300	1.81720500	2.96154900
C	3.11848300	-0.32403300	4.04235400
H	2.64882400	-2.04701600	2.85138000
C	3.18900200	1.06693900	4.07719800
H	2.89433300	2.90296600	2.99323200
H	3.38560100	-0.90940200	4.91892400
H	3.51965600	1.57089100	4.98274600
C	2.09003500	2.00969100	0.57167700
C	1.31757200	3.23935100	0.68094000
C	3.00281100	1.88797200	-0.53768100
C	1.47778100	4.21247100	-0.28811100
C	3.16193800	2.94155700	-1.47987800
C	2.39791500	4.06880500	-1.35126200
H	0.87475900	5.11392200	-0.25558600
H	3.89176900	2.84017600	-2.27847600
H	2.48302400	4.87409300	-2.07729800
C	-2.23495500	1.04541000	-3.23200300
C	-0.85936100	1.06130700	-2.58788000
O	-0.24887300	2.13847800	-2.39752000
O	-0.39371400	-0.05806600	-2.17935700
Rh	1.13456300	0.86524600	-1.01693800
H	3.80313900	1.15021000	-0.45752000
N	0.47182300	3.42598600	1.78199700
C	-0.15401900	4.72279600	1.91532900
H	-0.64999100	4.77617400	2.89013300
H	-0.91422400	4.91664000	1.13580300
H	0.59729400	5.51788000	1.87126200
C	-0.45259000	2.35166900	2.10572500
H	-1.26189200	2.26929600	1.35605700
H	-0.90207200	2.54662200	3.08583300
H	0.05745100	1.38873400	2.15814500
C	-5.79998700	-0.38859700	1.10948300
O	-5.68973900	0.09175600	2.21691800
O	-6.72403600	-1.32006400	0.80619600
C	-7.56011200	-1.70438000	1.88968200
H	-8.24777800	-2.45242700	1.49255800
H	-6.96774600	-2.12709600	2.70757600
H	-8.11514500	-0.84406500	2.27681100
H	-2.44345300	2.08263300	-3.53153500
C	-2.24385000	0.14465200	-4.45772000
H	-3.21693800	0.15978000	-4.96141000
H	-1.49090800	0.47901400	-5.17893300
H	-2.00649800	-0.88793500	-4.17891300

CP5

P	1.78767600	0.87363100	0.27249800
C	-3.80348800	-1.67456600	0.35548200
C	-3.79337700	-0.69626000	-0.64623600
C	-4.86681400	-1.69626300	1.25900200
C	-5.89694500	-0.77057500	1.16065800
C	-4.81767400	0.23059700	-0.74869400
C	-5.88166100	0.19709600	0.15751000
H	-4.89532700	-2.44369400	2.04781000
H	-6.73122100	-0.78038700	1.85779600
H	-2.95651800	-0.66472800	-1.34351000
H	-4.80399300	0.98194600	-1.53375400
C	0.82234800	1.19952400	-1.27876500

C	-0.52300100	1.87115700	-0.99058100
C	1.57308100	1.86147900	-2.43607600
H	0.60982500	0.16509600	-1.59168000
C	-1.37438800	1.90084700	-2.25579300
H	-0.37254300	2.90435800	-0.63664400
H	-1.04578500	1.32822400	-0.19124300
C	0.69337900	1.89892700	-3.68295100
H	1.86678900	2.88998600	-2.17511500
H	2.50135700	1.31226100	-2.64509900
C	-0.64019100	2.58277600	-3.40474400
H	-2.33193200	2.39900600	-2.05436000
H	-1.61292200	0.86273100	-2.54460600
H	1.22370500	2.39985400	-4.50356000
H	0.50647800	0.86429700	-4.01721300
H	-1.26199400	2.59549300	-4.30925000
H	-0.45462900	3.63676000	-3.13786600
C	2.07329400	2.49934100	1.16770500
C	3.17501300	2.38818300	2.22779100
C	2.22150700	3.80738200	0.38004500
H	1.11210900	2.57226200	1.70857300
C	3.14547300	3.58618900	3.17171800
H	4.15720300	2.34210800	1.73291400
H	3.07659400	1.45338300	2.79557600
C	2.18861400	5.00161000	1.33256800
H	3.17248200	3.82779300	-0.17444200
H	1.42427400	3.90838900	-0.36628000
C	3.26963700	4.89458500	2.40067200
H	3.94773000	3.49795400	3.91548200
H	2.19664400	3.58091300	3.73176400
H	2.29579800	5.93459700	0.76428400
H	1.20039100	5.04319600	1.81805700
H	3.22171500	5.75221600	3.08361900
H	4.25939900	4.93484300	1.91688100
C	3.43064200	0.26729800	-0.31214700
C	3.73274100	-1.10747400	-0.39043100
C	4.43311000	1.19091800	-0.64042100
C	5.04124500	-1.48933100	-0.71939300
C	5.71581400	0.79408700	-0.99336200
H	4.20880200	2.25408700	-0.61671800
C	6.02784500	-0.55951500	-1.01476600
H	5.26711300	-2.55438700	-0.75746300
H	6.46694600	1.54080300	-1.23943400
H	7.03176900	-0.89215900	-1.26806000
C	2.77929300	-2.22275100	-0.12236400
C	2.97519000	-2.95913000	1.05026000
C	1.77980800	-2.63179000	-1.03205500
C	2.18177000	-4.05163200	1.37286000
C	0.98759700	-3.73636200	-0.69453900
C	1.17792300	-4.43353000	0.49221500
H	2.34208400	-4.59134600	2.30230100
H	0.21190400	-4.06736400	-1.38031800
H	0.54586500	-5.29006600	0.71826800
H	3.76410200	-2.63678800	1.72947800
N	1.57147900	-1.91897100	-2.24252200
C	2.63512500	-2.03678200	-3.22902400
H	3.61101300	-1.81201300	-2.79126000
H	2.67483000	-3.04856100	-3.67406800
H	2.45607000	-1.31630300	-4.03729400

C	0.27576400	-2.09742600	-2.86894200
H	-0.51630700	-1.94844300	-2.12629800
H	0.16066700	-1.33956500	-3.65557900
H	0.15367700	-3.08686500	-3.34956700
C	-2.65102700	-2.65801600	0.44466800
C	-1.42807100	-1.89620200	0.92687700
O	-0.81925500	-1.15278000	0.08132000
O	-1.03830000	-1.96726500	2.11652700
Rh	0.60507200	-0.54953300	1.52968100
H	1.20067300	0.11482400	2.94861300
H	1.78653400	-0.50627800	2.72721600
C	-2.91298200	-3.88122000	1.30415300
H	-3.81356500	-4.41082400	0.97442300
H	-2.06310300	-4.56920400	1.24657000
H	-3.02565800	-3.60405200	2.35686600
H	-2.41302000	-2.96991000	-0.58454500
C	-7.01082800	1.15213900	0.10045700
O	-7.95365700	1.15467300	0.86061000
O	-6.88199600	2.03880400	-0.90615600
C	-7.94153400	2.98046700	-1.00833600
H	-7.69913000	3.62013800	-1.85831200
H	-8.89796100	2.47410700	-1.17322700
H	-8.02235700	3.57653200	-0.09372800

TS5-6

P	1.82447900	0.88715700	0.27636600
C	-3.84218100	-1.65194900	0.37916100
C	-3.79461300	-0.65912100	-0.60797000
C	-4.92778900	-1.67073600	1.25574100
C	-5.94074000	-0.72709600	1.14782300
C	-4.80032600	0.28654900	-0.71856600
C	-5.88596200	0.25683000	0.16214400
H	-4.98755800	-2.42883000	2.03247500
H	-6.79123900	-0.73454300	1.82515000
H	-2.93847000	-0.63168500	-1.28094700
H	-4.75375100	1.05128000	-1.48958600
C	0.87626300	1.20203800	-1.28602700
C	-0.45741100	1.90286800	-1.01446700
C	1.65090400	1.83499700	-2.44395500
H	0.64587000	0.16700900	-1.58768000
C	-1.29819700	1.93855300	-2.28661800
H	-0.28723100	2.93568000	-0.66719200
H	-0.99749400	1.37552900	-0.21555200
C	0.78108500	1.87914500	-3.69745700
H	1.96589400	2.85906400	-2.19088400
H	2.56787700	1.26214000	-2.63954800
C	-0.53992700	2.59364800	-3.43570500
H	-2.24628400	2.45906200	-2.09735300
H	-1.55754200	0.90323900	-2.56621200
H	1.32791100	2.36094100	-4.51860500
H	0.57524900	0.84549500	-4.02289100
H	-1.15400700	2.61089100	-4.34531300
H	-0.33407700	3.64602000	-3.17742900
C	2.12814000	2.50924200	1.15878000
C	3.17911000	2.36279700	2.26490100
C	2.35924000	3.79462800	0.35515500
H	1.14915900	2.62878300	1.65705300
C	3.17641000	3.58115900	3.18246900

H	4.17621300	2.25357000	1.81093500
H	3.00145500	1.44692300	2.84414200
C	2.35429800	5.00550400	1.28701000
H	3.32469700	3.76293400	-0.17282800
H	1.58707400	3.91771800	-0.41433100
C	3.39407900	4.86511600	2.39166900
H	3.94546600	3.46820700	3.95714800
H	2.20906400	3.63504700	3.70712300
H	2.52438100	5.92167300	0.70701500
H	1.35442700	5.10309900	1.73949700
H	3.36887100	5.73815100	3.05601000
H	4.39955100	4.84448000	1.93999200
C	3.44790400	0.21353100	-0.27482800
C	3.67675800	-1.17509300	-0.34474700
C	4.50141000	1.08496900	-0.58262200
C	4.96911000	-1.62586000	-0.64727200
C	5.76809400	0.61980700	-0.90901400
H	4.33150900	2.15827500	-0.56152500
C	6.00831600	-0.74852100	-0.92347000
H	5.14084900	-2.70123100	-0.67764200
H	6.56311600	1.32493500	-1.13908200
H	6.99858500	-1.13352500	-1.15524100
C	2.66003500	-2.23830700	-0.08876600
C	2.76831700	-2.94719200	1.11473500
C	1.69524000	-2.64202000	-1.03846500
C	1.93156700	-4.01157900	1.41881800
C	0.86604800	-3.72627600	-0.72134700
C	0.97366400	-4.39734400	0.48946300
H	2.02169800	-4.52477600	2.37216300
H	0.11976800	-4.05457900	-1.44006800
H	0.31169900	-5.23539200	0.69878700
H	3.52610300	-2.62604400	1.82846100
N	1.54789800	-1.94072400	-2.26465600
C	2.63387200	-2.10885100	-3.21886300
H	3.60378800	-1.90544900	-2.75790500
H	2.65459700	-3.12942000	-3.64478600
H	2.49869800	-1.39891700	-4.04474800
C	0.26297100	-2.08640300	-2.92387600
H	-0.54412300	-1.91451400	-2.20199400
H	0.18929000	-1.33012400	-3.71686900
H	0.12721700	-3.07423200	-3.40397200
C	-2.70765000	-2.65624300	0.47761000
C	-1.45442100	-1.90276900	0.89724300
O	-0.86944600	-1.18824200	0.01646800
O	-1.02754600	-1.96378000	2.07730400
Rh	0.61512900	-0.54493400	1.48896100
H	0.76299200	0.37973600	2.72443800
H	1.72463500	-0.47377100	2.59940300
C	-2.97538700	-3.84286200	1.38429700
H	-3.88368700	-4.37629300	1.08257700
H	-2.13315500	-4.54157000	1.34612600
H	-3.07637900	-3.52495000	2.42676800
H	-2.50083700	-3.00873700	-0.54584500
C	-6.99618400	1.23286000	0.09685100
O	-7.95635300	1.23952900	0.83508700
O	-6.82850500	2.13537000	-0.89019300
C	-7.86814300	3.09814400	-0.99778800
H	-7.59705100	3.74688600	-1.83211100

H	-8.83035200	2.61262000	-1.18960500
H	-7.95634700	3.68093000	-0.07527500

CP6

P	1.86675000	0.89845900	0.27651100
C	-3.83159600	-1.64299200	0.57143500
C	-3.91811400	-0.91678500	-0.62239000
C	-4.73935500	-1.35465000	1.59276800
C	-5.70425500	-0.37018500	1.42678300
C	-4.87736900	0.06710600	-0.79476300
C	-5.78138500	0.34772100	0.23415800
H	-4.69416300	-1.90384900	2.53020700
H	-6.41503500	-0.13943900	2.21659700
H	-3.20811000	-1.12968500	-1.41959400
H	-4.93576100	0.62199400	-1.72735200
C	0.79698500	1.29257800	-1.18218700
C	-0.50244400	1.98877500	-0.77182600
C	1.47414700	1.96359700	-2.37803900
H	0.52867000	0.27118300	-1.50067000
C	-1.45314100	2.04674400	-1.96240500
H	-0.29588900	3.01372300	-0.42017400
H	-0.97119100	1.44220000	0.05704100
C	0.49994500	2.04086000	-3.55141100
H	1.80921400	2.98015500	-2.11850300
H	2.37014700	1.39646700	-2.66777200
C	-0.80072900	2.73248500	-3.15743200
H	-2.38208100	2.55848100	-1.67987600
H	-1.73266300	1.01325300	-2.22885600
H	0.97142900	2.55388600	-4.39996800
H	0.27608000	1.01555400	-3.89075400
H	-1.49028300	2.76417900	-4.01092900
H	-0.58273300	3.78106800	-2.89374300
C	2.23527700	2.44803100	1.24035200
C	3.34240400	2.21203000	2.27265400
C	2.46642700	3.75420500	0.47299100
H	1.28915900	2.56618300	1.79843300
C	3.46001900	3.39588200	3.22681600
H	4.30265300	2.06853700	1.75206300
H	3.14469100	1.28606500	2.82908500
C	2.58233200	4.92719100	1.44446400
H	3.39121700	3.69777300	-0.12217800
H	1.64978600	3.93827100	-0.23623100
C	3.68771000	4.69685300	2.46721700
H	4.27139100	3.21841100	3.94398200
H	2.53325500	3.47607300	3.81685200
H	2.75463400	5.85660600	0.88671200
H	1.62175900	5.05114100	1.96978600
H	3.75132700	5.54450000	3.16118700
H	4.65748600	4.64612300	1.94524200
C	3.42905500	0.23530100	-0.41329800
C	3.54818500	-1.16016000	-0.51496200
C	4.51105500	1.04402100	-0.77931200
C	4.77409800	-1.70305300	-0.91871100
C	5.71237800	0.49092300	-1.20321300
H	4.41746400	2.12626300	-0.72568200
C	5.84923500	-0.89187100	-1.25714300
H	4.86660000	-2.78713300	-0.97614400
H	6.54164300	1.13821200	-1.47793900

H	6.79113200	-1.33934800	-1.56554500
C	2.45579700	-2.13349000	-0.19763500
C	2.53358200	-2.81765100	1.02946900
C	1.50615400	-2.56561500	-1.16366100
C	1.71914800	-3.91199800	1.31397000
C	0.71509200	-3.67727100	-0.86199200
C	0.81928900	-4.34574700	0.35427300
H	1.78936900	-4.40570500	2.27871600
H	-0.00774700	-4.02627800	-1.59497300
H	0.18434600	-5.20799800	0.54766600
H	3.29151900	-2.49950100	1.74299800
N	1.32317500	-1.84567700	-2.37220500
C	2.36456000	-1.98882200	-3.37796100
H	3.35509000	-1.79901000	-2.95767200
H	2.36281200	-2.99684100	-3.83347700
H	2.19209700	-1.25653700	-4.17690800
C	0.00685800	-1.96461600	-2.97438300
H	-0.76027700	-1.83184700	-2.20297400
H	-0.10760500	-1.17034200	-3.72413300
H	-0.15042000	-2.92822400	-3.49516100
C	-2.74295800	-2.68353100	0.73365900
C	-1.41701700	-1.96319400	0.97841700
O	-0.97933900	-1.20657400	0.06354000
O	-0.79256200	-2.13666600	2.05946500
Rh	0.81492100	-0.70603400	1.38900500
H	0.06942600	0.41140900	2.13716700
H	1.84136000	-0.43458300	2.52667700
C	-3.02017900	-3.74479900	1.78286100
H	-3.98581200	-4.23226400	1.60681200
H	-2.23345100	-4.50572500	1.76510600
H	-3.01553700	-3.31708600	2.79028700
H	-2.61382800	-3.16610700	-0.24973400
C	-6.83199600	1.38210800	0.11658100
O	-7.63829100	1.65212900	0.97934900
O	-6.80253100	2.01135300	-1.07571300
C	-7.79381800	3.01563200	-1.24120100
H	-7.64524200	3.42851400	-2.24021100
H	-8.79855600	2.59044000	-1.15076700
H	-7.68474100	3.79964500	-0.48491200

TS6-7

P	2.23409700	0.74209800	0.05628700
C	-4.59944700	-1.02012700	0.64866600
C	-5.60305200	-1.47362700	-0.20865600
C	-4.66305400	0.29157600	1.13110800
C	-5.70572700	1.12553400	0.76024000
C	-6.65180900	-0.64411700	-0.58061700
C	-6.70832200	0.66368500	-0.09596800
H	-3.87607100	0.65066600	1.79262000
H	-5.76706900	2.14884100	1.12263000
H	-5.55601000	-2.49328000	-0.58894900
H	-7.42961200	-1.00254200	-1.24905300
C	2.91134900	1.43350900	-1.53675300
C	2.01366000	2.55532800	-2.06665600
C	4.39334600	1.80312400	-1.62454500
H	2.76851900	0.55772100	-2.19204700
C	2.40358900	2.93447700	-3.49159100
H	2.10504800	3.44448200	-1.42046300

H	0.96101000	2.24183200	-2.02375100
C	4.75746300	2.17760700	-3.05983200
H	4.62304800	2.65296300	-0.96267100
H	5.01363700	0.96118300	-1.28921300
C	3.87820500	3.30800200	-3.58061300
H	1.77152400	3.75673600	-3.85038300
H	2.20572400	2.07608500	-4.15507900
H	5.81914800	2.44998800	-3.12082300
H	4.62422400	1.29185200	-3.70429300
H	4.15106000	3.56440100	-4.61232700
H	4.05844200	4.21188000	-2.97546400
C	2.10967700	2.10888000	1.32117500
C	1.87063600	1.50764000	2.71105600
C	3.19382700	3.18678000	1.38265500
H	1.17041800	2.59933200	1.00902900
C	1.50204400	2.59049000	3.71942600
H	2.79046900	0.99867000	3.04361300
H	1.08519800	0.73974300	2.66426900
C	2.81241600	4.26966000	2.39069900
H	4.15307300	2.74219200	1.69179700
H	3.35576700	3.63944600	0.39566600
C	2.56676400	3.67942500	3.77368000
H	1.35365800	2.14425900	4.71088900
H	0.53719800	3.03649300	3.43080900
H	3.59562600	5.03796300	2.42903400
H	1.89631700	4.77355700	2.04288200
H	2.27943300	4.46748600	4.48149200
H	3.50734800	3.24656000	4.15218500
C	3.51252100	-0.42934800	0.66823200
C	3.16517700	-1.78965800	0.72884000
C	4.77040600	-0.02752400	1.13347900
C	4.07968900	-2.69764900	1.27934600
C	5.67162200	-0.94047600	1.66714000
H	5.04935600	1.02288000	1.08887300
C	5.31970800	-2.28304700	1.74657600
H	3.80393500	-3.75036700	1.32532700
H	6.64026500	-0.60189800	2.02699000
H	6.01003100	-3.00860000	2.17049900
C	1.87283200	-2.36611900	0.24408200
C	1.07433200	-3.04089200	1.18838400
C	1.55332700	-2.52226300	-1.13710900
C	0.01222000	-3.84940200	0.81558700
C	0.47821800	-3.35421600	-1.49282100
C	-0.27932200	-4.00991800	-0.53554100
H	-0.58900200	-4.34255100	1.57584400
H	0.23902000	-3.48558900	-2.54477800
H	-1.10634800	-4.64280200	-0.84831800
H	1.32107600	-2.91157400	2.24136100
N	2.33634300	-1.87314200	-2.12803800
C	3.67042900	-2.40791400	-2.34696100
H	4.17953400	-2.59711400	-1.39811000
H	3.64865900	-3.34922100	-2.92690900
H	4.26539200	-1.67418200	-2.90822600
C	1.67126400	-1.58259800	-3.38251700
H	0.69029700	-1.12895000	-3.19564300
H	2.27967800	-0.86023900	-3.94468300
H	1.53858100	-2.47000600	-4.02907500
C	-3.49106900	-1.94911100	1.08818200

C	-2.14124600	-1.26412100	0.90695400
O	-1.62020300	-1.34963700	-0.24156700
O	-1.63931100	-0.65314300	1.88784200
Rh	0.32138000	-0.39298300	-0.21828200
H	-0.33538200	1.00761000	-0.38610000
H	-0.38626700	-0.08459800	1.17468700
C	-3.70927100	-2.41793800	2.52279700
H	-4.67592300	-2.92583700	2.61713500
H	-2.91760200	-3.10928000	2.83418800
H	-3.69215600	-1.56600500	3.20967900
H	-3.48658800	-2.81405400	0.40975800
C	-7.79605700	1.60021800	-0.45435200
O	-7.88635200	2.74124300	-0.05780300
O	-8.70005600	1.04426300	-1.28593600
C	-9.76743400	1.90298300	-1.66225900
H	-10.40801000	1.31979700	-2.32564100
H	-10.33008700	2.23377200	-0.78332200
H	-9.38965200	2.79013000	-2.18069400

CP7

P	2.35185000	0.71556300	0.06220500
C	-4.75979800	-0.92828100	0.73694900
C	-5.63810900	-1.36442200	-0.25605100
C	-4.97855500	0.31573900	1.33793500
C	-6.05507500	1.09924100	0.95365200
C	-6.71933600	-0.58400700	-0.64241300
C	-6.93346100	0.65401300	-0.03617500
H	-4.29091000	0.67235400	2.10202900
H	-6.23789800	2.06943800	1.40882700
H	-5.46911800	-2.32953000	-0.73113900
H	-7.40045400	-0.92852700	-1.41516800
C	2.83368800	1.49717300	-1.55813500
C	1.95514000	2.71065200	-1.86775200
C	4.31070300	1.77469000	-1.84116300
H	2.53954000	0.69013500	-2.24547500
C	2.15452700	3.16175800	-3.31106500
H	2.21266400	3.54401700	-1.19207200
H	0.90095100	2.46215300	-1.67905400
C	4.48764000	2.22751000	-3.28878900
H	4.70121900	2.55529500	-1.16867800
H	4.90470600	0.86895700	-1.65906100
C	3.62393700	3.44358300	-3.60275300
H	1.54076000	4.04718200	-3.52155100
H	1.79628900	2.36506100	-3.98419800
H	5.54549200	2.43936200	-3.49296800
H	4.20069100	1.39703800	-3.95619800
H	3.75915600	3.75280400	-4.64736900
H	3.95774100	4.29046600	-2.98028000
C	2.42485000	2.02664200	1.38924300
C	2.22833700	1.37322400	2.76029900
C	3.60372100	3.00064500	1.42758600
H	1.51376700	2.61034500	1.16793800
C	2.03045600	2.42213700	3.84883400
H	3.11627300	0.76286200	2.99621900
H	1.37501600	0.67993800	2.72020700
C	3.39019900	4.05559500	2.51198700
H	4.53457900	2.45515700	1.64883700
H	3.74274200	3.48998700	0.45454400

C	3.18872800	3.41255300	3.87865000
H	1.91301300	1.93591000	4.82604900
H	1.09235700	2.96719200	3.65562100
H	4.23929000	4.75141500	2.53227800
H	2.50073500	4.65443400	2.25742700
H	3.02218500	4.18103500	4.64463900
H	4.11077100	2.88014100	4.16432200
C	3.67818100	-0.49241400	0.45795600
C	3.25251400	-1.82732900	0.51485900
C	5.00897500	-0.18481900	0.76000900
C	4.16348800	-2.81763500	0.90066900
C	5.90589200	-1.17729000	1.13904300
H	5.35320900	0.84557600	0.70529800
C	5.47825500	-2.49846100	1.21609500
H	3.82554100	-3.85272100	0.94096100
H	6.93452600	-0.91751700	1.37846700
H	6.16974100	-3.28202400	1.51784800
C	1.86298300	-2.26264400	0.17261700
C	1.16883800	-3.03270200	1.14738800
C	1.39426500	-2.32496600	-1.17908100
C	0.10017600	-3.84209100	0.82294200
C	0.31498300	-3.19094000	-1.48473200
C	-0.32057800	-3.93198800	-0.51239200
H	-0.39928700	-4.41966700	1.59808100
H	-0.00479500	-3.27698400	-2.52115600
H	-1.14657400	-4.58608100	-0.78375700
H	1.53821900	-2.99311900	2.17169300
N	2.17676800	-1.74647100	-2.23110900
C	3.27486200	-2.59138700	-2.66892700
H	3.87938500	-2.90987900	-1.81320800
H	2.92457400	-3.49495200	-3.20586000
H	3.92220900	-2.01793300	-3.34605400
C	1.40745500	-1.26101100	-3.36055700
H	0.57983300	-0.63741700	-2.99818400
H	2.05745400	-0.64473400	-3.99827900
H	0.99886200	-2.06578100	-4.00114000
C	-3.61058100	-1.81475600	1.17199300
C	-2.29108900	-1.11290000	0.95368500
O	-1.60401000	-1.32507400	-0.03864700
O	-1.94682200	-0.26469200	1.90089200
Rh	0.45564700	-0.42393200	-0.02158200
H	-0.19289700	1.03590100	0.26538600
H	-1.10087400	0.16822600	1.57707700
C	-3.77497400	-2.28875900	2.61522900
H	-4.70670600	-2.85483100	2.71571700
H	-2.94162900	-2.93374800	2.91581100
H	-3.81163900	-1.44145200	3.30660700
H	-3.57625200	-2.68312700	0.50095900
C	-8.06665700	1.53638800	-0.40118500
O	-8.29072200	2.61314300	0.10484000
O	-8.83557300	1.00730300	-1.37071100
C	-9.93877800	1.81451600	-1.76323800
H	-10.45651700	1.26072100	-2.54759300
H	-10.60905400	1.99423500	-0.91675300
H	-9.59594500	2.78246500	-2.14207500

CP8

P	0.51842000	0.91046300	0.19275600

C	-0.47424500	-1.43050400	-1.82891400
H	-0.33287600	-0.52253500	-2.43463400
C	-1.88344600	-1.45354200	-1.35233700
C	-0.15984900	-2.62692100	-2.72783200
C	-2.34784200	-2.43552300	-0.45703800
C	-2.81566600	-0.50051200	-1.79735300
C	-4.12200800	-0.47975900	-1.32923700
C	-3.64923200	-2.42696800	0.01003400
C	-4.54900700	-1.43825600	-0.40832500
H	-2.50010700	0.24057100	-2.53403100
H	-4.82105100	0.27559600	-1.67899700
H	-1.65008200	-3.19292900	-0.10149900
H	-3.99987100	-3.17582300	0.71659100
H	-0.33643000	-3.58462000	-2.22243600
H	0.88643300	-2.61420300	-3.05915900
H	-0.79406600	-2.61762400	-3.62915900
C	-0.60667000	1.21472300	1.65870400
C	-2.06736100	0.95274000	1.28238100
C	-0.43828500	2.53048100	2.42198300
H	-0.30951000	0.40222700	2.34548700
C	-2.97941500	1.02668600	2.50123900
H	-2.40534300	1.69401900	0.53935100
H	-2.15596200	-0.02934900	0.80306300
C	-1.36455800	2.57101900	3.63566700
H	-0.67282000	3.38448500	1.76800800
H	0.60549500	2.65616300	2.73807900
C	-2.81848800	2.35224800	3.23394500
H	-4.02052700	0.86952600	2.18929700
H	-2.73207200	0.19990200	3.18654900
H	-1.24466600	3.52556300	4.16454000
H	-1.06385500	1.78187800	4.34390200
H	-3.47155700	2.39140400	4.11518700
H	-3.13686000	3.17397600	2.57072700
C	-0.06386600	1.96838000	-1.22389300
C	0.98587200	1.95619800	-2.34051400
C	-0.58153400	3.39047800	-0.99474200
H	-0.92430300	1.37587900	-1.57924400
C	0.40943300	2.52507900	-3.63284400
H	1.85277600	2.56053400	-2.02511400
H	1.35731500	0.93292100	-2.49916300
C	-1.16856800	3.94703600	-2.29093700
H	0.23857000	4.04819200	-0.66568800
H	-1.34361800	3.41184900	-0.20593700
C	-0.14452000	3.92876500	-3.41912900
H	1.17367900	2.52675400	-4.42063800
H	-0.39978200	1.86309100	-3.98437300
H	-1.54609000	4.96459700	-2.12650200
H	-2.03883600	3.33316000	-2.57770400
H	-0.58660800	4.31523300	-4.34633400
H	0.68506500	4.60741100	-3.16155700
C	2.16548200	1.53999000	0.70892100
C	3.16519100	0.57870600	0.94219100
C	2.47070000	2.89961300	0.85634300
C	4.44303300	1.01175300	1.31908700
C	3.74619500	3.31409500	1.21251100
H	1.69806800	3.64640500	0.68646000
C	4.73465000	2.36164000	1.44582200
H	5.21423200	0.26850100	1.50677100

H	3.96755600	4.37419500	1.31124400
H	5.73606300	2.67259100	1.73513400
C	2.90188400	-0.89518200	0.89486200
C	3.86384800	-1.79143500	0.28925800
C	2.03032400	-1.43184500	1.88431900
C	3.93756200	-3.10423900	0.74441100
C	2.14098900	-2.77099700	2.32397200
C	3.10183400	-3.58154600	1.76892700
H	4.64986100	-3.79161100	0.29906000
H	1.49596900	-3.12712200	3.12300900
H	3.21385900	-4.61051500	2.10346800
C	-5.91540000	-1.46486700	0.14672900
O	-6.33282600	-2.29212200	0.92842500
O	-6.67703200	-0.44637400	-0.30807900
C	-8.00574300	-0.43086700	0.19257300
H	-8.53558800	-1.35058400	-0.07596400
H	-8.49285800	0.43339500	-0.26202600
H	-8.01141300	-0.34013200	1.28375300
H	1.47246400	-0.73302900	2.50636100
N	4.73041400	-1.30601400	-0.69554600
C	5.76443900	-2.19878300	-1.16811100
H	6.44917800	-1.63625600	-1.81102200
H	5.36822400	-3.04828400	-1.75526100
H	6.34219300	-2.59835800	-0.32835600
C	4.14742900	-0.52840300	-1.77841300
H	3.54344200	-1.16072500	-2.45562900
H	4.95291000	-0.06183800	-2.35648800
H	3.50495100	0.26745400	-1.39727100
Rh	0.90529700	-1.31154300	-0.19580700
H	0.91014100	-3.12315900	-0.18467200
H	1.54065400	-2.94309700	-0.67671300

CP8'

P	0.12222200	0.84800400	-0.24196600
C	-1.10864900	-3.10718800	0.82102800
H	-1.52162200	-2.95031700	1.82259900
C	0.30427300	-2.92655000	0.72017400
C	-1.87294400	-4.07519900	-0.03891500
C	1.05606700	-3.30403500	-0.43555800
C	1.03824000	-2.34320000	1.80107200
C	2.37190900	-2.03518200	1.68098600
C	2.38926800	-2.98085800	-0.54937200
C	3.05880900	-2.29471600	0.47863300
H	0.50381100	-2.12149400	2.72567900
H	2.90377700	-1.56175200	2.50404900
H	0.55533900	-3.82415900	-1.24884200
H	2.94348600	-3.20676200	-1.45869600
H	-1.60973000	-3.99582700	-1.10168400
H	-2.95000300	-3.88575700	0.03410000
H	-1.69534800	-5.11992600	0.26500500
C	1.09957100	1.13147400	-1.81584500
C	2.41040900	0.34261200	-1.75699000
C	1.32359000	2.56634900	-2.29756100
H	0.46502400	0.63343800	-2.57212900
C	3.15029000	0.37815800	-3.08820500
H	3.06821700	0.76433700	-0.97701400
H	2.20814400	-0.69441100	-1.46144900
C	2.06395300	2.57306200	-3.63397600

H	1.91706200	3.12983400	-1.56085500
H	0.36361000	3.09078400	-2.39206900
C	3.38263000	1.81273100	-3.54577100
H	4.09580700	-0.17089700	-2.99078400
H	2.55201900	-0.15219700	-3.84727300
H	2.23118400	3.60706600	-3.96322200
H	1.42676900	2.10097000	-4.39953100
H	3.90417600	1.83344400	-4.51130600
H	4.04326600	2.31994100	-2.82242400
C	1.26449700	1.23449400	1.18485400
C	0.47693500	1.20369900	2.49752200
C	2.19599500	2.44850900	1.15310100
H	1.91050000	0.33855500	1.17486800
C	1.40939100	1.20590200	3.70503600
H	-0.18740200	2.08377700	2.53929800
H	-0.17314900	0.31672100	2.51843100
C	3.15211800	2.39459500	2.34335000
H	1.61214100	3.38091200	1.20924100
H	2.76982100	2.48417800	0.21895500
C	2.38501200	2.37573800	3.65973700
H	0.82429500	1.22613000	4.63387000
H	1.97944600	0.26259900	3.71553000
H	3.84478200	3.24584100	2.31097200
H	3.76491700	1.48065700	2.26011500
H	3.07660100	2.33248900	4.51099100
H	1.82359700	3.31924100	3.75999400
C	-1.20711300	2.12538800	-0.25759200
C	-2.53319200	1.67798300	-0.40857600
C	-0.96732700	3.49605200	-0.09107400
C	-3.57303400	2.61553500	-0.39615700
C	-2.01006300	4.41175100	-0.05253500
H	0.05439900	3.85362100	0.01317600
C	-3.31966700	3.96640500	-0.20851000
H	-4.59526500	2.26419500	-0.52124000
H	-1.80109800	5.46911700	0.09268500
H	-4.14490700	4.67487600	-0.19114600
C	-2.89220800	0.24388600	-0.64678600
C	-3.96751100	-0.37912700	0.09254200
C	-2.53453900	-0.33074200	-1.90635600
C	-4.64519400	-1.45590100	-0.48221000
C	-3.23871400	-1.42398000	-2.45101700
C	-4.29625900	-1.95855100	-1.74432300
H	-5.45371400	-1.93575800	0.06103900
H	-2.96047700	-1.80796200	-3.42923800
H	-4.85892000	-2.79638900	-2.15087300
C	4.39582300	-1.76821800	0.19652700
O	5.01369900	-1.93511500	-0.83691000
O	4.87002000	-0.99364700	1.20425700
C	6.12157200	-0.38011200	0.93676900
H	6.88929400	-1.12835000	0.71580000
H	6.38522600	0.18125200	1.83520200
H	6.04545800	0.29628200	0.07708700
H	-1.86439500	0.23683600	-2.55302600
N	-4.32595700	0.12734700	1.33890900
C	-5.51645600	-0.39311900	1.97115700
H	-5.77346000	0.24057300	2.82524900
H	-5.40251500	-1.43060000	2.33455500
H	-6.36201500	-0.36620900	1.27538600

C	-3.26521400	0.53283700	2.25184200
H	-3.58578000	0.34706300	3.28221600
H	-3.00943100	1.59759600	2.16128100
H	-2.35154000	-0.05574300	2.06019500
Rh	-1.18271000	-1.15265200	-0.20550800
H	-0.07505700	-1.82135900	-2.82271800
H	0.20062700	-1.88144800	-3.51787800

TS8-9

P	0.42587900	0.89010700	0.17860600
C	-0.27708700	-2.09504500	-1.43057900
H	-0.07284900	-1.39125400	-2.25853600
C	-1.70099200	-1.91939800	-1.02240700
C	-0.00779100	-3.51092600	-1.92786700
C	-2.26117700	-2.61776600	0.06128300
C	-2.55331500	-1.07433800	-1.74997000
C	-3.87596300	-0.87042100	-1.37948900
C	-3.57991200	-2.42937500	0.43374800
C	-4.39865400	-1.53843400	-0.27145300
H	-2.16180200	-0.56861100	-2.63374100
H	-4.51033600	-0.19610900	-1.94964300
H	-1.62528000	-3.28830600	0.63787800
H	-4.00438900	-2.95207100	1.28800100
H	-0.17044800	-4.25566300	-1.13997400
H	1.02776500	-3.61871700	-2.27094200
H	-0.67469800	-3.76385700	-2.76857200
C	-0.57371000	1.18291600	1.73256100
C	-2.02479200	0.74498500	1.51411700
C	-0.48601200	2.55609800	2.39875000
H	-0.12989400	0.45036900	2.43063500
C	-2.81003600	0.77947100	2.81994000
H	-2.51636900	1.41141000	0.78627000
H	-2.05026000	-0.26292100	1.08140700
C	-1.28879100	2.57238500	3.69850000
H	-0.88111200	3.33241800	1.72482500
H	0.56285800	2.81779200	2.59435700
C	-2.73775100	2.15773000	3.46604100
H	-3.85115300	0.48151500	2.63730800
H	-2.39080100	0.03084100	3.51157000
H	-1.23751900	3.56794200	4.15849200
H	-0.82549700	1.87385500	4.41424000
H	-3.29798600	2.17495700	4.40966300
H	-3.22071800	2.89362100	2.80145200
C	-0.47165900	1.73214500	-1.22409900
C	0.39672300	1.69517200	-2.48632000
C	-1.12761800	3.10663400	-1.07260500
H	-1.29410500	1.01316400	-1.37903200
C	-0.41914900	2.04178400	-3.72834100
H	1.22556800	2.41465100	-2.37602900
H	0.85730900	0.70226200	-2.60036400
C	-1.97522400	3.41086000	-2.30662100
H	-0.36099000	3.89061800	-0.97016400
H	-1.75243500	3.15084200	-0.17204200
C	-1.12926900	3.38143200	-3.57384300
H	0.22629300	2.04653200	-4.61635000
H	-1.17004800	1.25180100	-3.89489700
H	-2.47198400	4.38343100	-2.19576100
H	-2.77601300	2.65493800	-2.38039100

H	-1.74619200	3.59342200	-4.45647600
H	-0.37594300	4.18431800	-3.51864800
C	2.01032500	1.80044100	0.41816900
C	3.17637500	1.03054700	0.59446900
C	2.12387500	3.19517400	0.34557600
C	4.41613600	1.67968900	0.65723800
C	3.35959200	3.82542200	0.40278000
H	1.22673400	3.79955300	0.23429500
C	4.51187300	3.05945200	0.55032600
H	5.31380700	1.08023300	0.79444700
H	3.42267400	4.90873000	0.33150000
H	5.48711000	3.53887600	0.59676200
C	3.17365800	-0.44843800	0.83696000
C	4.10026100	-1.32410800	0.17108200
C	2.61028100	-0.89339300	2.05526500
C	4.51517500	-2.48578000	0.83145300
C	3.01681000	-2.07911300	2.67756500
C	4.00169900	-2.84063400	2.07830700
H	5.23495000	-3.14842500	0.36258200
H	2.58851800	-2.36330900	3.63467000
H	4.35836900	-3.75024700	2.55597800
C	-5.78379300	-1.34612000	0.20078000
O	-6.25759500	-1.86230300	1.18999400
O	-6.48865200	-0.51307700	-0.59327000
C	-7.82894300	-0.29100200	-0.17885000
H	-8.39009400	-1.23061100	-0.15229700
H	-8.26526600	0.39047500	-0.91091600
H	-7.86005800	0.15393200	0.82111400
H	1.97942300	-0.19589700	2.60477500
N	4.57555500	-0.99180800	-1.09909800
C	5.48847000	-1.92670500	-1.72418500
H	5.85074200	-1.49626100	-2.66194900
H	5.01806600	-2.90182700	-1.94633300
H	6.36023600	-2.09993700	-1.08511600
C	3.58979400	-0.44898600	-2.02230600
H	2.68022800	-1.08958100	-2.05643000
H	4.01989300	-0.40768600	-3.02718300
H	3.29160900	0.56835200	-1.75133000
Rh	1.14845700	-1.43563900	-0.02232700
H	0.49824700	-2.50632000	0.88898600
H	1.64973400	-2.94954800	0.04156900

TS8-9'

P	0.00221200	0.89131300	0.39292500
C	0.92331400	-3.25360300	-0.53834700
H	1.49437700	-2.84950800	-1.38523100
C	-0.49146200	-3.00605500	-0.63508500
C	1.50936500	-4.50799100	0.03967300
C	-1.41157500	-3.41117800	0.37913500
C	-1.03222300	-2.26729800	-1.72934500
C	-2.34863500	-1.87005400	-1.75162700
C	-2.72319500	-2.97823500	0.36501600
C	-3.20115000	-2.16735000	-0.67180400
H	-0.36641800	-2.00673400	-2.55344900
H	-2.73703300	-1.29519300	-2.58941100
H	-1.05341900	-4.01996700	1.20530800
H	-3.40110800	-3.22554900	1.17899900
H	0.94272900	-4.87349700	0.90285700

H	2.53855500	-4.33593800	0.37572400
H	1.53573900	-5.31711400	-0.70747000
C	-1.02526600	1.31537800	1.90385900
C	-2.35180100	0.54756500	1.81865400
C	-1.24503100	2.77113700	2.31733600
H	-0.43681400	0.84041900	2.70888900
C	-3.12568800	0.63590300	3.12823500
H	-2.97542000	0.96728200	1.01070900
H	-2.16367500	-0.50402800	1.55506000
C	-2.02265400	2.83513500	3.63144400
H	-1.80954500	3.31587600	1.54487000
H	-0.28152300	3.28643700	2.42552400
C	-3.34777000	2.08775700	3.53466000
H	-4.08053800	0.10336300	3.02955800
H	-2.55497300	0.11845900	3.91655600
H	-2.18671800	3.88214500	3.91834700
H	-1.41226800	2.38432700	4.43070800
H	-3.89207700	2.14594200	4.48604800
H	-3.98415200	2.57952300	2.77983700
C	-1.07725800	1.28044300	-1.09417600
C	-0.24344200	1.17090100	-2.37361600
C	-1.95614300	2.53263900	-1.14607600
H	-1.76211400	0.41457400	-1.07605500
C	-1.11102000	1.19390700	-3.62904400
H	0.46674700	2.01493400	-2.40887500
H	0.35897100	0.25040400	-2.34754900
C	-2.85867100	2.48410000	-2.37789000
H	-1.33160200	3.43767900	-1.20470800
H	-2.57071300	2.62275100	-0.24210500
C	-2.03138500	2.40757400	-3.65468400
H	-0.47498200	1.17076000	-4.52383600
H	-1.72435400	0.27961600	-3.66032000
H	-3.51822200	3.36177000	-2.39602500
H	-3.50925200	1.59574900	-2.30550400
H	-2.68106400	2.37777300	-4.53887800
H	-1.42332000	3.32290700	-3.74296700
C	1.38732000	2.11731500	0.31623100
C	2.73642600	1.69534800	0.30391000
C	1.11824800	3.48599800	0.15142200
C	3.74462300	2.64948300	0.10122000
C	2.12773400	4.41741100	-0.04118200
H	0.08831200	3.83107500	0.17452200
C	3.45182900	3.99213900	-0.07292300
H	4.77814000	2.31240500	0.08741000
H	1.88077800	5.46915100	-0.16625000
H	4.25723700	4.70778100	-0.22173000
C	3.19931000	0.30822900	0.58747600
C	4.09445000	-0.38847300	-0.26322100
C	2.88297500	-0.25221200	1.83490000
C	4.67540100	-1.57567000	0.19933300
C	3.47451800	-1.44062300	2.27847000
C	4.38609400	-2.08725000	1.46106700
H	5.36441100	-2.12031100	-0.43970300
H	3.21767300	-1.83413200	3.25811500
H	4.86383600	-3.00745900	1.78971000
C	-4.53970400	-1.58005700	-0.53304500
O	-5.31236000	-1.79989000	0.37654000
O	-4.81896400	-0.69881100	-1.52438700

C	-6.07789200	-0.05308900	-1.40353500
H	-6.89479500	-0.78176700	-1.40121900
H	-6.16372900	0.61153500	-2.26537700
H	-6.13062900	0.52351300	-0.47335400
H	2.26707300	0.32856100	2.52043500
N	4.41325100	0.13367900	-1.53569000
C	5.55441600	-0.44146600	-2.21147500
H	5.80227000	0.18290900	-3.07710600
H	5.37511500	-1.46918100	-2.58176600
H	6.42426800	-0.46008400	-1.54681900
C	3.28500700	0.29018900	-2.43689400
H	2.85444300	-0.68670800	-2.73376500
H	3.61244300	0.81296000	-3.34284200
H	2.49563400	0.88732000	-1.97492500
Rh	0.75237500	-1.44869300	0.79694200
H	0.16758900	-1.47193400	2.22878600
H	1.02750600	-2.63312100	1.82715400

CP9

P	0.38448000	0.88400600	0.14901800
C	-0.18791600	-2.35263500	-1.24513600
H	0.02991200	-1.78868100	-2.17131900
C	-1.61221600	-2.10353700	-0.88127000
C	0.07489400	-3.83121400	-1.50627100
C	-2.20928400	-2.69488800	0.24571100
C	-2.42547900	-1.29772100	-1.69085200
C	-3.74939100	-1.03167200	-1.36627400
C	-3.52841900	-2.44190500	0.57523600
C	-4.31032900	-1.59299200	-0.21819100
H	-1.99906200	-0.87102500	-2.59981000
H	-4.35417200	-0.38920500	-2.00212100
H	-1.60338800	-3.33332300	0.88760400
H	-3.98180000	-2.87938100	1.46203700
H	-0.05238000	-4.43434700	-0.60023700
H	1.09923300	-3.99113600	-1.86073600
H	-0.61858700	-4.22231100	-2.26899700
C	-0.54868000	1.18808100	1.74332700
C	-2.00505100	0.73972400	1.58987500
C	-0.44316700	2.56394600	2.40031000
H	-0.07289400	0.46278600	2.42776100
C	-2.73113100	0.76712100	2.92964000
H	-2.53446000	1.40471400	0.88719100
H	-2.04306200	-0.26808700	1.15728500
C	-1.18624300	2.57620300	3.73521400
H	-0.87472300	3.33575000	1.74356900
H	0.61134700	2.83600600	2.54723000
C	-2.64051100	2.14689400	3.57089100
H	-3.77745900	0.46115000	2.79428800
H	-2.27481400	0.02224200	3.60159900
H	-1.12422900	3.57324100	4.19066600
H	-0.68378800	1.88399600	4.43043300
H	-3.15613900	2.16006000	4.53967900
H	-3.16120900	2.87728300	2.92920100
C	-0.64240200	1.65406200	-1.21080300
C	0.08547000	1.52064400	-2.55225500
C	-1.25832500	3.04959900	-1.08007300
H	-1.48509800	0.94204000	-1.23497900
C	-0.84664000	1.82415800	-3.72271000

H	0.94133000	2.21686900	-2.57129600
H	0.50176500	0.50708500	-2.65512800
C	-2.22555100	3.30271900	-2.23456100
H	-0.47295100	3.81960800	-1.11491100
H	-1.77972500	3.16562300	-0.12179900
C	-1.51106400	3.18744000	-3.57539100
H	-0.29681600	1.76110200	-4.67081600
H	-1.63042600	1.04961600	-3.76520500
H	-2.69246300	4.29051400	-2.12826600
H	-3.04160300	2.56174300	-2.18629900
H	-2.20852900	3.36566100	-4.40387800
H	-0.74175100	3.97438800	-3.63904100
C	1.93880000	1.87442300	0.26996200
C	3.14833400	1.16630700	0.41495500
C	1.98873100	3.26881800	0.13763600
C	4.35988100	1.86748300	0.36716700
C	3.19648500	3.95227700	0.09118700
H	1.06256600	3.83268000	0.06749600
C	4.38894300	3.24313500	0.19232900
H	5.28926200	1.31265600	0.47857800
H	3.20555000	5.03361000	-0.02441400
H	5.34306200	3.76397500	0.15457600
C	3.22765700	-0.28986600	0.75624300
C	4.15858800	-1.17189800	0.10757600
C	2.74643500	-0.67045500	2.02920500
C	4.66751100	-2.25837400	0.82608600
C	3.23713600	-1.78881600	2.70976100
C	4.23250000	-2.54413000	2.11905600
H	5.39647700	-2.91866300	0.36801800
H	2.86646400	-2.02456800	3.70331600
H	4.65625000	-3.39831400	2.64193200
C	-5.69644400	-1.32414800	0.21139800
O	-6.19783400	-1.73689800	1.23508500
O	-6.36733800	-0.54873100	-0.66585900
C	-7.70755700	-0.25771000	-0.29603800
H	-8.29315600	-1.17729500	-0.19629600
H	-8.11492700	0.36651300	-1.09296000
H	-7.74222000	0.27475700	0.66003100
H	2.10335200	0.03265800	2.55732600
N	4.53256900	-0.92040700	-1.21570400
C	5.44616800	-1.86314600	-1.82825400
H	5.71885600	-1.49838800	-2.82260000
H	5.01059800	-2.87364600	-1.93369700
H	6.36809200	-1.94127000	-1.24342300
C	3.44142100	-0.53492800	-2.09727600
H	2.59543500	-1.25829900	-2.02684600
H	3.79263500	-0.53244300	-3.13337400
H	3.07075700	0.46951900	-1.87428200
Rh	1.21706600	-1.43471500	0.03101600
H	0.37818100	-2.08266500	1.14026400
H	1.79003100	-2.90280500	0.19335000

CP9'

P	0.34843900	1.10463300	0.20154200
C	0.13890500	-2.81327500	-0.80266300
H	0.57183200	-2.26955500	-1.65256400
C	-1.27497900	-2.56530200	-0.64462100
C	0.76617200	-4.15687900	-0.57612000

C	-2.09680500	-3.27863600	0.26910200
C	-1.89976700	-1.53692000	-1.39900800
C	-3.23086600	-1.21355800	-1.23454900
C	-3.42813500	-2.95656000	0.42930900
C	-4.01420800	-1.91810600	-0.30926300
H	-1.29758500	-1.00951500	-2.13744500
H	-3.68067700	-0.41945600	-1.82755400
H	-1.65773300	-4.07310100	0.86734700
H	-4.05296200	-3.49775300	1.13568700
H	0.40648000	-4.64053400	0.33815100
H	1.85514200	-4.05760600	-0.47837600
H	0.56967600	-4.83904800	-1.41926800
C	-0.41529000	1.96229300	1.68042700
C	-1.91545600	1.64732200	1.72791200
C	-0.14218600	3.44463200	1.93469500
H	0.04582400	1.40169800	2.51374400
C	-2.51721600	2.09212000	3.05618500
H	-2.43351900	2.17041600	0.90717100
H	-2.07694800	0.57034500	1.57033600
C	-0.75748000	3.87427700	3.26543700
H	-0.57029500	4.06119400	1.12902300
H	0.93857400	3.63847500	1.93813400
C	-2.25060500	3.57086900	3.31270000
H	-3.59449200	1.88325500	3.07106800
H	-2.07119400	1.49268600	3.86634700
H	-0.57256300	4.94270700	3.43760200
H	-0.25369300	3.33438100	4.08350500
H	-2.67495500	3.87944800	4.27681400
H	-2.76314900	4.16803200	2.53995700
C	-0.60141500	1.68393300	-1.31123400
C	0.12838800	1.23559200	-2.58178100
C	-1.08246400	3.12921100	-1.46796700
H	-1.50755300	1.05904600	-1.21857700
C	-0.75873600	1.38045300	-3.81520200
H	1.03579400	1.84936700	-2.70976900
H	0.47347000	0.19516900	-2.47786800
C	-1.99360600	3.25228700	-2.68833900
H	-0.22518500	3.80604100	-1.60451600
H	-1.61713900	3.46671100	-0.57174500
C	-1.27881300	2.80565800	-3.95734700
H	-0.20779800	1.07731900	-4.71499700
H	-1.61622100	0.69192200	-3.72817000
H	-2.35052300	4.28557200	-2.78836500
H	-2.88762700	2.62588100	-2.53232800
H	-1.94439400	2.88781900	-4.82603900
H	-0.42969200	3.48216400	-4.14795600
C	2.04772000	1.81294800	0.02382400
C	3.18793200	0.99256700	0.17223500
C	2.23530300	3.15239200	-0.35322400
C	4.45390600	1.53708700	-0.09168700
C	3.49407400	3.67647100	-0.60628100
H	1.37013600	3.80267600	-0.44943400
C	4.61146500	2.85727200	-0.48039100
H	5.32533700	0.89691000	0.02080500
H	3.60148300	4.71841400	-0.89878800
H	5.60730100	3.25020000	-0.67240200
C	3.17454900	-0.40236900	0.69757800
C	3.84981600	-1.46707500	0.04454500

C	2.66700600	-0.62143300	1.98958300
C	4.06486100	-2.65757400	0.74961400
C	2.88085300	-1.82553100	2.67092400
C	3.60432100	-2.82965600	2.05248300
H	4.59058000	-3.47846100	0.27040100
H	2.48317400	-1.95251700	3.67384800
H	3.79248300	-3.76889600	2.56685100
C	-5.43986500	-1.62538300	-0.08140400
O	-6.15813900	-2.22252300	0.69182500
O	-5.88217500	-0.59776800	-0.83912600
C	-7.25387200	-0.27795000	-0.65912500
H	-7.89081400	-1.13282900	-0.90865800
H	-7.46264100	0.55808000	-1.32876700
H	-7.45590800	0.00646100	0.37872500
H	2.22480400	0.22131700	2.51982500
N	4.33474000	-1.29440700	-1.26846700
C	5.25357700	-2.29416700	-1.76378800
H	5.68309900	-1.94062500	-2.70767800
H	4.77011400	-3.27075800	-1.96143100
H	6.07349500	-2.44968500	-1.05516500
C	3.33962800	-0.95726700	-2.27248900
H	2.69651600	-1.82489500	-2.51761300
H	3.84328600	-0.63284400	-3.19055000
H	2.70105600	-0.13761000	-1.93456500
Rh	0.40328600	-1.29805300	0.81481400
H	-0.43085500	-1.18109300	2.18252900
H	0.39103000	-2.63930500	1.66019700

TS9-10

P	0.33715700	0.81389300	0.22977100
C	-0.18117800	-2.70837700	-0.73407400
H	0.15913300	-2.36660800	-1.72806600
C	-1.61608900	-2.32888000	-0.56832100
C	0.03254300	-4.21557000	-0.63299200
C	-2.36012900	-2.74469600	0.54829000
C	-2.27725000	-1.56956900	-1.54165900
C	-3.60239300	-1.18439300	-1.38841100
C	-3.68179900	-2.36774200	0.71014300
C	-4.31249200	-1.56711000	-0.24833200
H	-1.72642100	-1.26525000	-2.43283600
H	-4.09044300	-0.57702700	-2.14765700
H	-1.87393100	-3.34352200	1.31775900
H	-4.24903700	-2.66135600	1.59073100
H	-0.11721800	-4.59353500	0.38522900
H	1.05443300	-4.47744400	-0.92154000
H	-0.66726000	-4.75093500	-1.29207300
C	-0.65803600	1.12690900	1.78886500
C	-2.10852700	0.69156300	1.56785500
C	-0.59251600	2.49300800	2.46958700
H	-0.21171500	0.39431800	2.48615200
C	-2.89002800	0.67513500	2.87541600
H	-2.60514500	1.38540000	0.86819000
H	-2.13656300	-0.29941000	1.10143500
C	-1.37862600	2.46866600	3.78019000
H	-1.02104700	3.26641200	1.81138400
H	0.45066400	2.78265100	2.65500800
C	-2.82487300	2.03417000	3.56223900
H	-3.92956500	0.37472700	2.68288300

H	-2.46204200	-0.09346600	3.53983500
H	-1.33914300	3.45523700	4.26036400
H	-0.89164200	1.76458800	4.47441600
H	-3.36726400	2.01524900	4.51620400
H	-3.33227700	2.78089400	2.92905700
C	-0.70006800	1.48156200	-1.17593200
C	-0.00958200	1.21292800	-2.51574800
C	-1.23809200	2.91357300	-1.15109300
H	-1.57119900	0.80636000	-1.12677200
C	-0.96427800	1.45178700	-3.68293700
H	0.86468500	1.88032100	-2.61094200
H	0.37795500	0.18303300	-2.54160700
C	-2.22917200	3.12162300	-2.29415000
H	-0.40684500	3.62347300	-1.27923900
H	-1.71504700	3.14556100	-0.18962200
C	-1.56695000	2.85114100	-3.63988000
H	-0.44878800	1.28211900	-4.63730600
H	-1.78001600	0.71041900	-3.63290700
H	-2.63685100	4.14050700	-2.26290500
H	-3.08327200	2.43689100	-2.15726500
H	-2.28427500	2.98558600	-4.45969700
H	-0.76744800	3.59311100	-3.79877400
C	1.82160500	1.90394400	0.32667000
C	3.07345700	1.26297200	0.36053000
C	1.77822500	3.30492000	0.30742800
C	4.23838600	2.04003600	0.31706000
C	2.93989900	4.06401900	0.26155100
H	0.81779800	3.81279400	0.32857000
C	4.17563100	3.42431800	0.25171100
H	5.20404300	1.53884800	0.34464400
H	2.88040000	5.14956400	0.23407000
H	5.09408100	4.00610400	0.21504600
C	3.23280000	-0.21200200	0.57982400
C	4.25035100	-0.96253600	-0.12564900
C	2.84801600	-0.69547500	1.86953100
C	4.92817900	-1.98231000	0.54988500
C	3.53510800	-1.73996100	2.50748100
C	4.59707600	-2.33652700	1.85890700
H	5.72018400	-2.53110000	0.05106300
H	3.25324600	-2.03451800	3.51468100
H	5.16174200	-3.12735500	2.34771800
C	-5.70059900	-1.13719100	0.01489800
O	-6.31297900	-1.36537000	1.03592800
O	-6.22745700	-0.43927400	-1.01115100
C	-7.55902800	0.01104800	-0.80209000
H	-8.23530800	-0.83437100	-0.64029300
H	-7.84155000	0.55524000	-1.70457300
H	-7.61579600	0.66733700	0.07231800
H	2.19424700	-0.06164600	2.46874300
N	4.55563600	-0.62948000	-1.43970400
C	5.54224900	-1.43885000	-2.12435700
H	5.73214600	-1.01007300	-3.11184200
H	5.22156100	-2.48759200	-2.25253700
H	6.49154300	-1.43333400	-1.57853800
C	3.45031200	-0.18768400	-2.27265700
H	2.57064500	-0.85612400	-2.15063400
H	3.75820400	-0.20175500	-3.32185000
H	3.13442800	0.83280300	-2.03145500

Rh	1.36101400	-1.41730600	0.12335800
H	0.29121200	-2.29023600	0.85839000
H	2.15302400	-2.78569700	0.18038800

TS9-10'

P	0.00556100	1.13555800	-0.12188500
C	-1.13213700	-3.02025600	-1.48987900
H	-1.75445600	-3.33612800	-0.64416700
C	0.25972400	-2.82359400	-1.12911100
C	-1.48232900	-3.74265800	-2.77021000
C	1.21439600	-2.35324800	-2.07986900
C	0.73191000	-3.10064100	0.18618400
C	2.04433800	-2.89872400	0.53604200
C	2.51779900	-2.08633700	-1.69657200
C	2.95435700	-2.34868200	-0.39402600
H	0.02525000	-3.50125000	0.91393600
H	2.38792500	-3.14124900	1.53863900
H	0.90404100	-2.18059900	-3.10779200
H	3.23199900	-1.67065200	-2.40428800
H	-0.95118500	-3.33250700	-3.63665600
H	-2.55599800	-3.66802100	-2.97893000
H	-1.22588500	-4.80939500	-2.69651900
C	0.31208400	2.45630200	-1.41156500
C	1.43569300	2.00875600	-2.35399900
C	0.47904600	3.91350600	-0.97887100
H	-0.62473900	2.39763700	-1.99478100
C	1.53402700	2.93758800	-3.55913700
H	2.40006800	2.00792100	-1.82110800
H	1.25225200	0.97427000	-2.67820400
C	0.58020300	4.82208300	-2.20254700
H	1.38724800	4.03564100	-0.36961600
H	-0.36676000	4.22259300	-0.35069800
C	1.71261500	4.38760800	-3.12533200
H	2.35943800	2.62286300	-4.21051600
H	0.61107200	2.84574100	-4.15483600
H	0.71505200	5.86452100	-1.88551200
H	-0.37139400	4.78465500	-2.75733100
H	1.77216000	5.04904100	-3.99936400
H	2.67190300	4.48809500	-2.59069700
C	1.60816700	0.88058300	0.83287400
C	1.31091200	0.14779100	2.14683400
C	2.56095600	2.05627300	1.07206100
H	2.13496100	0.16870500	0.17008000
C	2.58304700	-0.26063100	2.88555200
H	0.71208900	0.80631500	2.79794600
H	0.69779400	-0.74275100	1.93936500
C	3.84169200	1.58134700	1.75554300
H	2.08529600	2.81108900	1.71807300
H	2.81002200	2.55727600	0.12910200
C	3.52557100	0.91823600	3.08921000
H	2.31761400	-0.71617100	3.84911100
H	3.10894300	-1.03556900	2.30918200
H	4.52653700	2.42838200	1.89281300
H	4.35788700	0.86339000	1.09446800
H	4.44666400	0.58923800	3.58945500
H	3.05482500	1.65925800	3.75646500
C	-1.19218500	1.87789400	1.07273200
C	-2.48180100	1.32486200	1.22305200

C	-0.81119300	2.92714300	1.92396400
C	-3.32199000	1.82754100	2.22722200
C	-1.65436500	3.41340300	2.91224000
H	0.17641900	3.36840800	1.81519600
C	-2.91840700	2.85340600	3.06688800
H	-4.31292300	1.39341100	2.33768000
H	-1.32500500	4.22362900	3.55856400
H	-3.59407300	3.22214800	3.83527300
C	-3.08867600	0.29638600	0.32632900
C	-3.70286500	-0.88475900	0.83321700
C	-3.31389500	0.64626700	-1.01466000
C	-4.55150100	-1.61036000	-0.00891200
C	-4.17379400	-0.08991500	-1.83782200
C	-4.80112500	-1.20706700	-1.32106800
H	-5.02953700	-2.51432000	0.35614800
H	-4.33814600	0.22585300	-2.86439400
H	-5.48095200	-1.79138500	-1.93733300
C	4.32884700	-1.97880300	-0.03844800
O	5.07189900	-1.30271300	-0.72221900
O	4.69157800	-2.44488300	1.18058200
C	5.96412200	-2.00497900	1.63007200
H	6.75116500	-2.29026900	0.92524500
H	6.12726000	-2.48161800	2.59821900
H	5.98024100	-0.91312000	1.73935200
H	-2.90592000	1.58949900	-1.37400300
N	-3.49295100	-1.27202400	2.17038700
C	-4.32935500	-2.33330400	2.68366500
H	-4.18387300	-2.40390200	3.76699100
H	-4.09430300	-3.32354700	2.24773200
H	-5.38605500	-2.11539300	2.49782800
C	-2.10935600	-1.44109200	2.58138600
H	-1.63607100	-2.31298900	2.08839400
H	-2.07120200	-1.59400000	3.66577700
H	-1.51407400	-0.55545000	2.34925800
Rh	-0.99706400	-0.75381900	-1.25508500
H	-0.96166900	0.02551100	-2.67140100
H	-1.80180600	-1.57293500	-2.34292400

CP10

P	0.41730600	0.89425800	0.09931800
C	-0.42649200	-2.71612200	1.09429700
H	0.16527000	-2.63602500	0.14891200
C	-1.83884400	-2.28864100	0.82085800
C	-0.32503400	-4.15567800	1.58885800
C	-2.61829300	-1.71370600	1.82858000
C	-2.41030600	-2.48185600	-0.43974400
C	-3.70908900	-2.06908600	-0.70483500
C	-3.91329800	-1.29068200	1.57112500
C	-4.46186800	-1.45154000	0.29705600
H	-1.81394600	-2.94213100	-1.22878600
H	-4.14311100	-2.20673000	-1.69221600
H	-2.18797500	-1.58192700	2.82168500
H	-4.51991600	-0.81915900	2.34092600
H	-0.88910400	-4.28594900	2.52040900
H	0.72041100	-4.42059100	1.77305300
H	-0.74007200	-4.85021700	0.84833600
C	-0.07852900	2.09298900	1.45168900
C	-1.36560000	1.60293200	2.12059100

C	-0.12828600	3.59111500	1.14702800
H	0.73363400	1.94320600	2.18728600
C	-1.70760300	2.42835300	3.35599500
H	-2.20634200	1.65736800	1.40858100
H	-1.25711400	0.54151000	2.38456200
C	-0.46979100	4.38903800	2.40340800
H	-0.88625600	3.80128600	0.37728600
H	0.83443700	3.92358700	0.73796700
C	-1.77859300	3.91341700	3.02265000
H	-2.65441600	2.07978500	3.78956900
H	-0.93235000	2.26759100	4.12254400
H	-0.51826400	5.45985900	2.16630600
H	0.34183100	4.26871100	3.13935500
H	-2.01811300	4.49953100	3.91916400
H	-2.60021900	4.08450300	2.30724700
C	-1.00505400	0.79455200	-1.11902900
C	-0.51180500	0.08226900	-2.38434900
C	-1.82286800	2.03481600	-1.48641800
H	-1.69145700	0.10060000	-0.60110900
C	-1.65472400	-0.25169500	-3.33731600
H	0.21211600	0.73480400	-2.90104500
H	0.03724400	-0.83091800	-2.10616100
C	-2.98100300	1.65967400	-2.40991900
H	-1.18642400	2.77201600	-2.00177200
H	-2.21432700	2.52622300	-0.58651600
C	-2.47516800	0.98552300	-3.67867000
H	-1.25829100	-0.71835100	-4.24878900
H	-2.31314900	-0.99568200	-2.86200100
H	-3.57110000	2.55300700	-2.65313800
H	-3.65810200	0.97015400	-1.87732600
H	-3.31268500	0.72280600	-4.33784600
H	-1.84584500	1.69686400	-4.23847700
C	1.80929800	1.71937400	-0.77526200
C	3.06032300	1.08514300	-0.67185100
C	1.69408900	2.88694000	-1.54083000
C	4.16082500	1.64892400	-1.33058900
C	2.78730900	3.41997300	-2.21051700
H	0.72892300	3.38257600	-1.62273000
C	4.02665100	2.79577200	-2.09941800
H	5.13125600	1.16537600	-1.23894700
H	2.67398300	4.31867700	-2.81246200
H	4.89447100	3.20832000	-2.60957200
C	3.29344100	-0.12365200	0.18975900
C	4.22657800	-1.14833100	-0.26947500
C	3.19098700	0.07333700	1.61452800
C	5.09442000	-1.72979900	0.64825800
C	4.06495000	-0.58620700	2.51642200
C	5.02628000	-1.43080900	2.01949800
H	5.81911300	-2.46775300	0.31966700
H	4.00287000	-0.36471600	3.57881700
H	5.72757300	-1.91764700	2.69425700
C	-5.83509200	-0.94646500	0.06788200
O	-6.54011600	-0.45686900	0.92175400
O	-6.22128000	-1.08026600	-1.21498800
C	-7.53577700	-0.61298400	-1.49313200
H	-8.27503100	-1.15455400	-0.89468300
H	-7.70270200	-0.79101000	-2.55627900
H	-7.62417900	0.45411000	-1.26633900

H	2.72550300	0.99753000	1.96128000
N	4.26374300	-1.46907100	-1.62092400
C	5.19862000	-2.49001500	-2.03993200
H	5.17861900	-2.56914500	-3.13024500
H	4.97324500	-3.48267000	-1.61180200
H	6.21941100	-2.21819000	-1.74896500
C	3.01159600	-1.43614200	-2.35838200
H	2.20145200	-1.92531000	-1.78359500
H	3.14391300	-1.95404500	-3.31255900
H	2.69037300	-0.41146400	-2.57784700
Rh	1.48731600	-1.12571700	0.83442600
H	0.00062700	-2.05324100	1.89555400
H	2.36688000	-2.39299000	1.25894900

CP10'

P	0.94273200	0.84078000	-0.06285900
C	-3.82216400	-2.56187800	-0.14015800
H	-4.12928400	-2.43786000	0.90955400
C	-2.32911800	-2.69444800	-0.21131200
C	-4.52342900	-3.77846200	-0.73906400
C	-1.64857800	-2.37178500	-1.40226500
C	-1.60297600	-3.23732400	0.85305200
C	-0.23010300	-3.43881200	0.76023700
C	-0.25992300	-2.54365200	-1.49659400
C	0.45109900	-3.08373400	-0.40470200
H	-2.13093100	-3.50791200	1.76778200
H	0.32075200	-3.86512700	1.59480900
H	-2.22098000	-2.06460600	-2.28164400
H	0.26555300	-2.36936500	-2.43136300
H	-4.24671800	-3.90897300	-1.79217600
H	-5.61305400	-3.67971800	-0.68606600
H	-4.23704400	-4.69434600	-0.20808900
C	2.15796300	1.49858300	-1.31937900
C	3.00869300	0.34665800	-1.86391000
C	3.00748800	2.72246000	-0.97313000
H	1.47320200	1.79496200	-2.13391600
C	3.84026200	0.80258400	-3.05753000
H	3.68841900	-0.02592100	-1.07811600
H	2.36638800	-0.49979900	-2.14316700
C	3.83283000	3.15532400	-2.18347600
H	3.69202600	2.49802100	-0.14008200
H	2.36650700	3.54901500	-0.63933700
C	4.69643600	2.01276300	-2.70451400
H	4.46366700	-0.02592000	-3.41682900
H	3.16098200	1.06577500	-3.88437300
H	4.45137300	4.02471400	-1.92418000
H	3.14994700	3.48445100	-2.98332900
H	5.28362600	2.33965900	-3.57257400
H	5.42264900	1.72641300	-1.92515000
C	1.93507800	-0.10945700	1.21524300
C	1.01481100	-0.56021300	2.35304600
C	3.22506800	0.48864500	1.78420000
H	2.21756100	-1.01262900	0.64286200
C	1.71991000	-1.54169700	3.28478800
H	0.70020100	0.32837100	2.92757700
H	0.10218500	-1.01428400	1.93981800
C	3.92570200	-0.50345100	2.71098000
H	2.98897300	1.39544600	2.36210300

H	3.90833600	0.78838700	0.97974300
C	3.00803500	-0.94911600	3.84202200
H	1.04479400	-1.83826700	4.09867400
H	1.95964900	-2.45717900	2.71956600
H	4.84620400	-0.05637900	3.10902500
H	4.23460100	-1.38541700	2.12541400
H	3.51879000	-1.67270000	4.49077200
H	2.76604300	-0.07833900	4.47324400
C	0.22907800	2.30775700	0.79615300
C	-1.15668300	2.51260500	0.65553200
C	0.96833400	3.18183300	1.60571400
C	-1.75991000	3.57179100	1.34550000
C	0.35483700	4.22127300	2.29177100
H	2.04302000	3.04808100	1.70262400
C	-1.01767400	4.41200200	2.16230700
H	-2.83068900	3.72626100	1.23293700
H	0.94590300	4.88040600	2.92349500
H	-1.50980400	5.22526500	2.69127600
C	-2.01402400	1.70613400	-0.27147700
C	-3.30246200	1.20353900	0.15858800
C	-1.80893100	1.90265300	-1.67695700
C	-4.28797300	0.96894800	-0.79150600
C	-2.85483300	1.66701800	-2.61061000
C	-4.06940900	1.21601000	-2.16568100
H	-5.25503700	0.58188800	-0.48441000
H	-2.67594200	1.86672200	-3.66390200
H	-4.88086000	1.03747600	-2.86801200
C	1.90726500	-3.24061400	-0.55791200
O	2.53651500	-2.96896900	-1.55910100
O	2.50178500	-3.70147100	0.56605100
C	3.91675500	-3.79760500	0.49109800
H	4.22157500	-4.53110400	-0.26226500
H	4.25325600	-4.10656700	1.48299400
H	4.35729500	-2.82899800	0.22347700
H	-0.96937200	2.52392300	-1.98633400
N	-3.55187800	1.03452000	1.52991900
C	-4.90733800	0.73218100	1.92619200
H	-4.98251700	0.81162200	3.01608500
H	-5.23069200	-0.28700700	1.63928100
H	-5.60572100	1.45070700	1.48470000
C	-2.57629100	0.29053100	2.30739400
H	-2.58212900	-0.78871500	2.06046400
H	-2.80411500	0.40678000	3.37325500
H	-1.56524900	0.66695400	2.14010400
Rh	-0.89183300	-0.09028800	-1.15422400
H	-0.22881000	0.08100200	-2.66465900
H	-4.13324000	-1.64629000	-0.66554000
